# Stromal and endothelial transcriptional changes during progression from MGUS to myeloma and after treatment response

**DOI:** 10.1038/s41467-026-74389-y

**Published:** 2026-06-23

**Authors:** Itziar Cenzano, Miguel Cócera, Marta Larrayoz, Lorea Campos-Dopazo, Sonia Sanz, Azari Bantan, Amaia Vilas-Zornoza, Patxi San-Martin, Paula Aguirre-Ruiz, Diego Alignani, Aitziber Lopez, Ignacio Sancho González, Javier Ruiz, Purificacion Ripalda-Cemborain, Marta Abengozar-Muela, Emma Muiños-López, Vincenzo Lagani, Jesper Tegner, Mikel Hernáez, Xabier Agirre, Benjamin Ebert, Bruno Paiva, Paula Rodriguez-Otero, Luis-Esteban Tamariz-Amador, Jesús San-Miguel, Borja Saez, José A. Martinez-Climent, Isabel A. Calvo, David Gomez-Cabrero, Felipe Prosper

**Affiliations:** 1 Hematology and Oncology Program, Centre for Applied Medical Research (CIMA), Instituto de Investigaciones Sanitarias de Navarra (IdiSNA), Cancer Center Clinica Universidad de Navarra (CCUN), Pamplona, Spain; 2 Division of Biomedical Sciences, King Abdullah University of Science and Technology (KAUST), Thuwal, Saudi Arabia; 3 Centro de Investigacion Biomedica en Red de Cancer (CIBERONC), Madrid, Spain; 4 Hospital Reina Sofía de Tudela, Tudela, Spain; 5 Hospital Universitario de Navarra, Instituto de Investigaciones Sanitarias de Navarra (IdiSNA), Pamplona, Spain; 6 Departament of Pathology, Clinica Universidad de Navarra, Pamplona, Spain; 7 SDAIA-KAUST Center of Excellence in Data Science and Artificial Intelligence, Thuwal, Saudi Arabia; 8 Institute of Chemical Biology, Ilia State University, Tbilisi, Georgia; 9 Computer Science Program, Electrical and Mathematical Sciences and Engineering Division, King Abdullah University of Science and Technology (KAUST), Thuwal, Saudi Arabia; 10 Unit of Computational Medicine, Department of Medicine, Center for Molecular Medicine, Karolinska Institutet, Karolinska University Hospital, Stockholm, Sweden; 11 Science for Life Laboratory, Solna, Sweden; 12 Computational Biology Program. Cima Universidad de Navarra, Data Science and Artificial Intelligence Institute (DATAI) Universidad de Navarra, Instituto de Investigaciones Sanitarias de Navarra (IdiSNA), Pamplona, Spain; 13 Department of Medical Oncology, Dana-Farber Cancer Institute, Harvard Medical School, Boston, MA USA; 14 Hematology and Cell Therapy Department. Cancer Center Clinica Universidad de Navarra (CCUN), Instituto de Investigaciones Sanitarias de Navarra (IdiSNA), Pamplona, Spain

**Keywords:** Myeloma, Transcriptomics, Stem-cell niche

## Abstract

Progression from monoclonal gammopathy of undetermined significance (MGUS) to multiple myeloma (MM) is accompanied by profound remodeling of the bone marrow microenvironment (BME), yet the contribution of its non-immune compartment remains unclear. Using single-cell RNA sequencing in genetically engineered mouse models that recapitulate disease evolution, we transcriptionally profile endothelial cells (EC) and mesenchymal stem cells (MSC). EC adopt a stress-associated program at MGUS that precedes angiogenesis in MM, while MSC undergo early and sustained loss of differentiation capacity. We identify a coordinated interferon (IFN)-driven program across EC and MSC that defines MM in the BIcγ1 model but is absent in the more aggressive MI_cγ1_ model. Treatment with bortezomib, lenalidomide, and dexamethasone suppresses this IFN signature, promotes endothelial adaptation, and restores osteogenic potential in MSC. Validation in patient samples reveals enrichment of this IFN-signature across disease stages. These findings define dynamic and targetable alterations in the non-immune BME during myeloma progression.

## Abstract (web summary)

Progression from MGUS to multiple myeloma involves changes in the bone marrow mi croenvironment beyond malignant plasma cells. Here, authors use single-cell tran scriptomics to show that during the transition from MGUS to active myeloma, endothe lial and stromal cells undergo early stress and differentiation changes and acquire an interferon-related signature in myeloma, features that are partially reversed after stand ard therapy.

## Introduction

Multiple myeloma (MM) is a B-cell malignancy characterized by the clonal expansion of plasma cells (PC) in the bone marrow (BM), preceded by asymptomatic precursor stages: monoclonal gammopathy of undetermined significance (MGUS) and smoldering multiple myeloma (SMM)^1^. Understanding the molecular mechanisms driving disease progression is crucial for developing therapies to prevent symptomatic MM.

The BM microenvironment (BME) comprises a complex network of hematopoietic and non-hematopoietic cells, extracellular matrix components, and soluble factors. This microenvironment creates an immunosuppressive and proinflammatory milieu that supports the growth and survival of malignant PC^2^. Therefore, the intricate interactions between myeloma cells and their surrounding microenvironment are crucial for developing effective therapies that can disrupt supportive signals and overcome drug resistance. While most studies have focused on the immune component^3–6^, growing evidence suggests that endothelial cells (EC) and mesenchymal stem cells (MSC) promote myeloma PC^7–10^. Furthermore, an enhanced inflammatory signature has been identified in MSC from MM patients^11–13^. However, the role of the non-hematopoietic BME in MGUS-to-MM progression remains poorly understood^14^.

In this study, we leveraged our recently described mouse models of MM, that recapitulate the clinical, genetic, molecular, and immunological characteristics of MM patients^15^. These models include a precursor stage MGUS-like that progresses into symptomatic MM. Using single-cell RNA sequencing (scRNA-seq) data from fluorescence-activated cell-sorted (FACS) non-hematopoietic BME cells and PC, we analyzed transcriptional changes during disease transition and in response to current therapy. Our results in the BI_cγ1_ model revealed distinct transcriptional changes between EC and MSC at the different stages of the disease. EC exhibited an altered stress profile at MGUS, followed by the upregulation of angiogenesis pathways during MM. In contrast, MSC exhibited early downregulation of pathways associated with adhesion, osteogenic, and myogenic differentiation at the MGUS stage. Interestingly, we identified an interferon (IFN)-related myeloma signature in both EC and MSC that was reversed after treatment with bortezomib (Velcade®), lenalidomide (Revlimid®), and dexamethasone (VRd). Changes were validated in MSC from patients with MGUS, SMM, and MM, highlighting their potential role in disease progression.

## Results

### Single-cell profiling of murine BM in control, MGUS, and MM

**Fig. 1:**
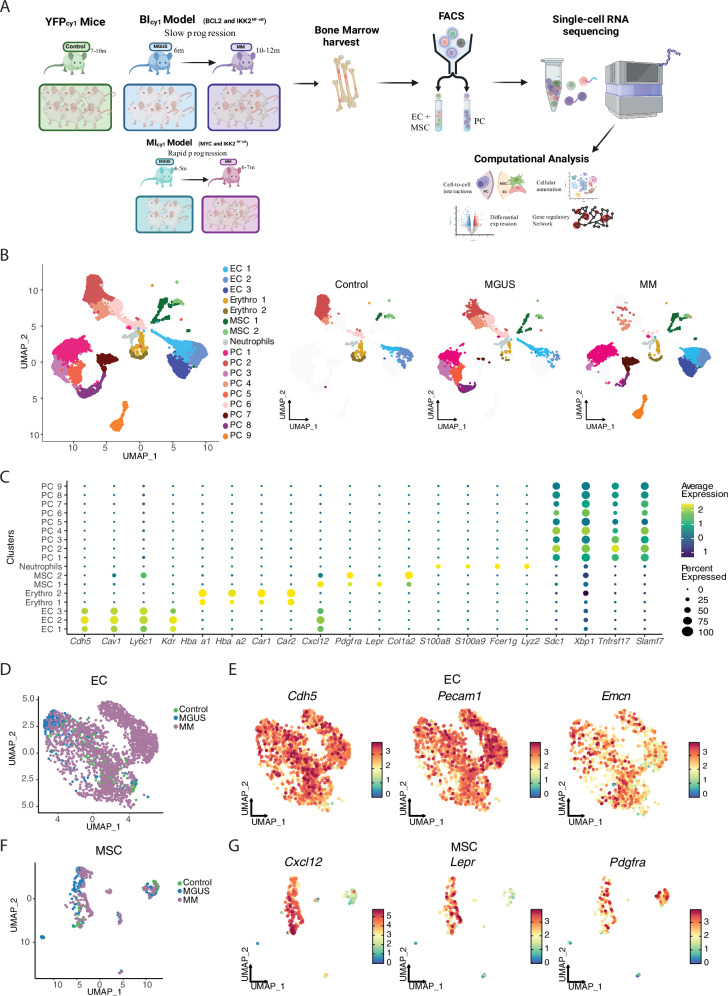
Single-cell profiling of murine BM in control, MGUS, and MM. **A** Experimental workflow for isolation and scRNA-seq analysis of PC, MSC and EC from control (YFPcγ1; pool of six mice mice), MGUS BIcγ1 (*n* = pool of five mice), MM BIcγ1 (*n* = pool of five mice), MGUS MIcγ1 (*n* = pool of four mice) and MM MIcγ1 (*n* = pool of five mice). Created with BioRender.com. **B** UMAP representation of murine BM microenvironment from control, MGUS, and MM cells colored by cell clustering (left) and split by condition (right). **C** Dot plot of canonical markers used to define PC, EC, MSC, erythroblasts, and neutrophils. Dot size indicates percentage of expressing cells; color indicates average expression. **D** UMAP projection of EC from control (*n* = 315 cells), MGUS (*n* = 262 cells), and MM (*n* = 2060 cells). **E** UMAP visualization of representative EC markers. **F** UMAP projection showing of MSC from control (*n* = 132 cells), MGUS (*n* = 143 cells), and MM (*n* = 229 cells). **G** UMAP visualization of representative MSC markers.

Using the BI_cγ1_ myeloma model^15^, we aimed to characterize transcriptional changes in BM stromal and EC during the transition from normal and precursor conditions to MM. This model, which carries BCL2 and IKK2^NF-κB^ genetic lesions triggered in germinal center B cells by a cre-cγ1 allele, develops signs of MGUS-like around 6 months of age that progressively evolve into MM within 4–6 months, overcoming limitations of previous models. Considering that BI_cγ1_ mice received T-cell-driven immunization, we included similarly immunized YFP_cγ1_ mice, carrying a yellow-fluorescence reporter activated from germinal-center B cells, as controls (Fig. 1A). We performed scRNA-seq on FACS-sorted non-hematopoietic BME cells (Lin^-^, TER119^-^, CD45^-^, Vybrant dye orange (VDO)^+^, and 7-aminoactinomycin D (7AAD)^-^) and PC (VDO^+^, 7AAD^-^ and GFP^+^), yielding 12437 cells (control=2416, MGUS = 4169, MM = 5852) (Supplementary Fig. 1). Using canonical markers, we identified 17 clusters comprising EC, MSC, PC, and other immune cells from the BME (Fig. 1B, Supplementary Fig. 2A, B). As a result, eight clusters were labeled as PC (clusters PC1-9; *n* = 7924; markers: *Sdc1*, *Xbp1, Tnfrsf17*, and *Slamf7*); two clusters as erythroblasts (Erythro1-2; *n* = 461; *Hba-a1*, *Hba-a2, Car1*, and *Car2*); one cluster as neutrophils (*n* = 176; *S100a8*, *S100a9, Fcer1g*, and *Lyz2*); three clusters as EC (EC1-3; *n* = 2637; *Cdh5*, *Cav1*, *Ly6c1*, and *Kdr*); and two clusters as MSC (MSC1-2; *n* = 504; mesenchymal and osteolineage-primed markers *Cxcl12*, *Pdgfra*, *Lepr*, and *Col1a2*) (Fig. 1C, Supplementary Fig. 2C–F).

To better understand the transcriptional shifts of the non-hematopoietic BME during MM progression, we focus our analysis on EC and MSC (Fig. 1D–G, Supplementary Fig. 2G–J). Overall, these results support our cell enrichment strategy for isolating EC and MSC within the BME.

### Identification of a MM-specific IFN signature in the BME

**Fig. 2:**
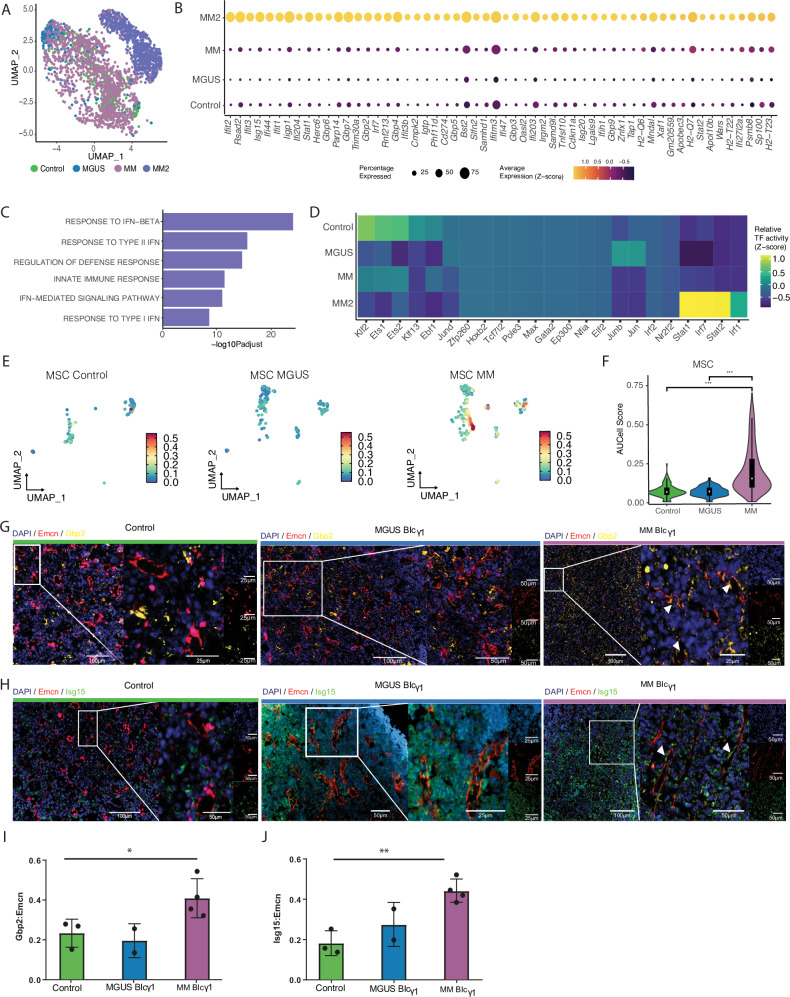
Identification of a MM-specific IFN signature in the BME. **A** UMAP projection of EC revealing a transcriptionally distinct population in MM. **B** Dot plot of the markers defining EC MM2 cluster. Dot size indicates the percentage of cells expressing each gene; color indicates average expression. **C** Bar plot of significant enriched GO terms identified by ORA using MM2 cluster markers. The x axis shows −log10(adjusted *P* value). **D** Heatmap representing z-scaled mean regulon activity score of inferred top specific regulons for each stage. **E** UMAP plot illustrating the distribution of the IFN-related signature in MSC across control, MGUS, and MM. **F** Violin plots showing AUC-score for the IFN-related signature within MSC from control (*n* = 132 cells), MGUS (*n* = 143 cells), and MM (*n* = 229 cells). Box plots are overlaid; center line, median; box limits, first and third quartiles; whiskers, 1.5 × interquartile range. Pairwise comparisons were performed using two-sided Wilcoxon rank-sum tests. **G**, **H** IF staining of EC (Emcn, red), IFN response genes markers of the MM2 cluster (Gbp2, yellow (**G**); Isg15, green (**H**)), and nucleus (DAPI) (blue) in FFPE femurs from Control (YFPcγ1), MGUS BIcγ1 and MM BIcγ1. White arrows point EC Emcn+ IFN+ (Gbp2 (**G**)/Isg15 (**H**)) in MM BIcγ1. Representative images from biologically independent mice with similar results. Scale bars, 50 μm. **I**, **J** Quantification of the percentage of endothelial IFN-enriched cells, based on the coexpression of Emcn with IFN+ cells (Gbp2, (**I**); Isg15, (**J**)) in femurs from Control (YFPcγ1; *n* = 3 mice), MGUS BIcγ1 (*n* = 2 mice) and MM BIcγ1 mice (*n* = 4 mice). Data are presented as mean ± SD. Statistical significance was determined using an unpaired two-tailed Student’s t-test. *P < 0.05; **P < 0.01; ***P < 0.001. Source data are provided as a Source Data file.

Delving into the composition of EC, our analysis revealed a cluster of cells shared across all stages alongside a second cluster (MM2) that was nearly unique to the MM stage (Fig. 2A). Cells within the MM2 cluster originated from both male and female mice, indicating that its emergence is not driven by a single animal (Supplementary Fig. 3). The MM2 cluster was characterized by the expression of IFN-inducible genes such as *Ifit2*, *Rsad2*, *Ifit3*, *Isg15*, *Ifi44, Ifit*, and *Oasl2*, indicating an upregulation of an IFN response, as supported by gene ontology (GO) over-representation analysis (ORA) (Fig. 2B, C).

To uncover the putative transcription factors (TFs) that might underlie the IFN-like signature, we performed gene regulatory network (GRN) inference using SCENIC^16^. This approach reconstructs TF–centered regulatory programs (regulons), defined as sets of co-expressed genes predicted to be regulated by the same TF, and quantifies their activity at the single-cell level. Using this framework, we identified Irf7, Stat1, and Stat2 as candidate regulators with increased regulon activity in MM2 EC (Fig. 2D, Supplementary Fig. 4A–C). These TFs are known positive regulators of type I and type II IFN responses, which are associated with T-cell immunosuppressive function in MM patients^17^.

To determine whether the IFN-like signature was unique to EC, we evaluated the signature score related for MM2 EC across other BM populations. This signature was also significantly upregulated in a subset of MSC at the MM stage as well as PC, neutrophils, and erythroblasts, suggesting a shared immunomodulatory response of the BME to the disease (Fig. 2E, F, Supplementary Fig. 4D). To further validate the MM-specific IFN signature, we performed quantitative PCR (qPCR) analysis of the IFN regulon (gene *Stat1)* and the associated gene (*Gbp2)* in MSC derived from mesenspheres^18^ of control and MM BIcγ1 mice (Supplementary Fig. 4E). We observed an enrichment of both the IFN *Stat1* and *Gbp2* expression in MSC from MM BIcγ1 mice compared with controls, confirming activation of the IFN signaling.

As the signature was not present in control YFP_cγ1_ mice or at precursor stages, we termed it the MM-IFN signature to emphasize its association with the myeloma stage. Immunofluorescence (IF) staining of BM samples from control YFP_cγ1_ and BI_cγ1_ mice at the MGUS and MM stages validated the increased proportion of IFN^+^ EC and MSC in the MM stage (Fig. 2G–J, Supplementary Fig. 4F–I).

Interestingly, when the MM-IFN signature was analyzed in the MI_cγ1_ model^15^, which carries MYC and IKK2^NF-κB^ from germinal-center B cells, no significant enrichment was observed at any stage in MSC and EC (Supplementary Fig. 5A). We confirm these scRNA-seq results by IF staining of EC and MSC IFN^+^ in BM biopsies from MM MI_cγ1_ mice (Supplementary Fig. 5B-I). This finding prompted us to investigate this MM-IFN signature in patients. The analysis of the only publicly available dataset from MM patients^12^ that contains non-immune BME uncovered an enrichment of the murine MM-IFN-related genes in certain EC and MSC from a subset of MM patients (Supplementary Fig. 5J, K). Likewise, previous studies have described a similar IFN-inducible signature with highly overlapping marker genes in PC and immune microenvironment cells from certain MM patients^3,4^. Collectively, our findings suggest that IFN-signaling is a coordinated response within the BME that could define a subgroup of MM patients.

### A stress state characterizes EC during the MGUS stage

**Fig. 3:**
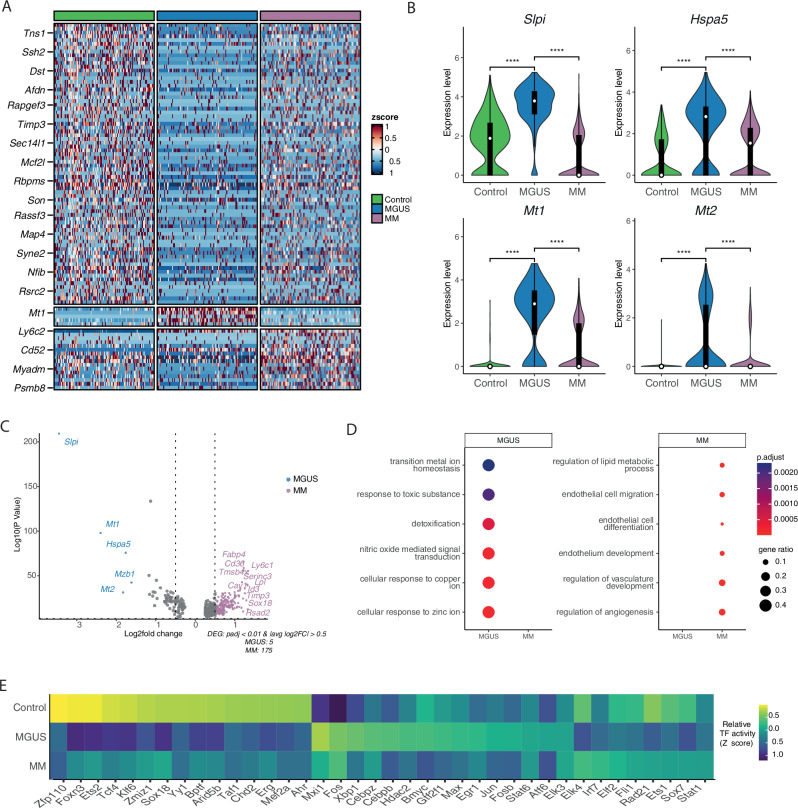
A stress state characterizes EC during the MGUS stage. **A** Heatmap of the DEGs between control, MGUS, and MM cells. Color represents the z-score scaled expression values. **B** Violin plots showing the distribution of expression of genes upregulated in MGUS EC. Control, *n* = 315 cells; MGUS, *n* = 262 cells; MM, *n* = 2060 cells. Box plots are overlaid; center line, median; box limits, first and third quartiles; whiskers, 1.5 × interquartile range. Pairwise comparisons were performed using two-sided Wilcoxon rank-sum tests. **C** Volcano plot of the DEGs in MGUS vs. MM EC. The y-axis represents the -log10(*p*-value), and the x-axis represents the log2(fold change). The dot’s color denotes the disease stage for which DEG was detected, with gray dots representing non-significant genes. DE was assessed using two-sided MAST test with Bonferroni correction. **D** Significant terms derived from ORA between EC from MGUS and MM. **E** Heatmap representing z-scaled mean regulon activity score of inferred top specific regulons for EC in each stage. *P < 0.05; **P < 0.01; ***P < 0.001. Source data are provided as a Source Data file.

To investigate the molecular mechanisms of myeloma progression, we analyzed the transcriptional changes in EC across different stages of the disease. An initial analysis uncovered a distinct transcriptome profile of EC at the MGUS stage (Fig. 3A). MGUS EC overexpressed *Slpi*, *Hspa5*, *Mt1*, and *Mt2*, indicating a stress state at the onset of the disease related to protein folding, reticulum stress, and ion homeostasis (Fig. 3B, Supplementary Fig. 6A, B, Supplementary Data 1). Moreover, *Cd74*, *Tmem176a*, and *Nme2* were also overexpressed in MGUS EC (Supplementary Fig. 6C). To assess whether non-IFN MM EC (*n* = 1193 cells) also exhibit disease-associated transcriptional remodeling, we performed a differential expression analysis (DEA) between MGUS and non-IFN MM EC. This analysis identified 180 differentially expressed genes (DEGs) most of them upregulated (175 upregulated and five downregulated) (Fig. 3C; Supplementary Data 2), with MM EC showing a pro-vascular and lipid metabolism profile (Fig. 3D). *Sox18*, a well described gene involved in tumor vascular development^19^, as well as *Fabp4* and *Cav1* were upregulated, suggesting their role in malignant transformation (Supplementary Fig. 6D). When we extend the transcriptional analysis to the MIcγ1 model, we observed a stress-related transitional phenotype in MGUS MIcγ1 EC like that seen in BIcγ1 (Supplementary Fig. 7). However, MGUS MIcγ1 EC overexpressed genes of MM EC, indicating a stronger vascular phenotype and faster progression.

Next, we examined changes in regulons of the BI_cγ1_ model between MGUS and non-IFN MM EC. The analysis revealed a decline in the activity of regulons during disease progression (Fig. 3E, Supplementary Fig. 8A, B). Xbp1, which promotes endothelial proliferation via VEGF^20^, showed the highest regulon activity in MGUS EC, while the Fos regulon activity was maintained at both stages. Moreover, the role of Fos has been previously described in PC^21^, and our findings point to a potential regulatory role in EC along with MM development (Supplementary Fig. 8C, D). Globally, these findings suggest that a stress-related regulatory state characterizes MGUS EC, prior to the angiogenic phenotype observed in MM and may be associated with disease progression.

### Early MSC transcriptional changes in MGUS persist in MM

**Fig. 4:**
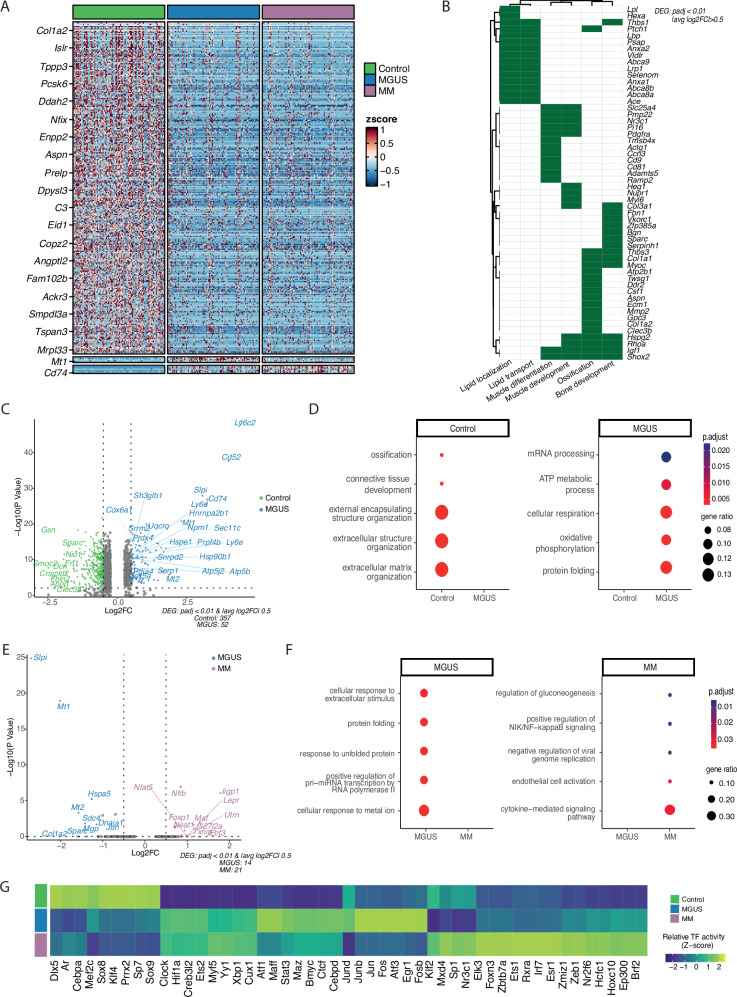
Early MSC transcriptional changes in MGUS persist in MM. **A** Heatmap depicting DEGs between MSC in control (*n* = 132 cells), MGUS (*n* = 143 cells), and MM (*n* = 229 cells). Color represents the z-score scaled expression values. **B** Heatmap of significantly downregulated GO terms (p. adjusted <0.05) in MGUS and MM cells compared to control MSC with the associated genes. The green color represents the presence of the DEGs in each GO term. **C** Volcano plot of the DEGs from the comparison between MSC from control and MGUS. The y-axis represents the -log10(*p*-value), and the x-axis represents the gene’s log2(fold change). The dot’s color denotes the disease stage for which DEG was detected, with gray dots representing non-significant genes. DE was assessed using two-sided MAST test with Bonferroni correction. **D** Significant terms derived from ORA of MSC from control vs MGUS. **E** Volcano plot of the DEGs in MSC from MGUS vs. MM. **F** Significant terms derived from ORA between MSC from MGUS and MM. DE was assessed using two-sided MAST test with Bonferroni correction. **G** Heatmap of mean regulon activity score of significantly enriched regulons in each stage. The color scale represents the z-score of the activity scaled by row. Source data are provided as a Source Data file.

Unlike EC, DEA indicated that MSC deregulation begins as early as the premalignant MGUS stage (Fig. 4A). GO overrepresentation analysis revealed impaired osteogenic and adipogenic differentiation capacity in MSC during MM development, consistent with prior studies^8,11,22^ (Fig. 4B, Supplementary Data 3). Additionally, we identified downregulation of muscle development genes, including *Pi16*, *Myl6*, *Enpp2, Nupr1*, and *Shox2*, that have not previously been linked to MM.

Given early changes at the MGUS stage, we compared MGUS and control MSC, identifying 409 DEGs (52 upregulated and 357 downregulated) (Supplementary Data 4). MGUS MSC exhibited enhanced expression of genes and pathways related to oxidative phosphorylation, ATP metabolism, protein folding, and RNA processing (Fig. 4C, D). Upregulation of *Cd74* in MGUS MSC and EC suggests its potential role in MM progression. Only 35 DEGs were identified between MM and MGUS MSC (21 upregulated and 14 downregulated), further indicating the high level of transcriptional similarity between the two stages. Upregulated genes in MM MSC were primarily associated with the immune response, consistent with the inflammatory profile in human MM MSC^12^ (Fig. 4E, F, Supplementary Data 5). Similar transcriptional changes were observed in MIcγ1 mice, supporting the impairment of MSC differentiation early in the MGUS-like stage (Supplementary Fig. 9).

Finally, GRN analysis identified regulons associated with MSC alterations during MM progression (Fig. 4G, Supplementary Fig. 10A). In control MSC Sp7, Dlx5, Sox8, Sox9, and Cebpa, well-known TFs mediating osteochondrogenic and adipogenic differentiation, were identified. Reduced Klf4 activity supported impaired myogenic potential in MGUS and MM MSC. Moreover, TFs from the AP-1 family and Egr1 showed the highest regulon activity in MGUS MSC, consistent with their involvement in MM development^23,24^. Yy1 and Xbp1 regulons remained active from MGUS to MM, while Elk3 was specific to MM, suggesting its role in cancer cell invasion^25–27^ (Supplementary Fig. 10B, C). We next validated the activation of these regulons in MM MSC derived from mesenspheres^18^ of control and MM BIcγ1 mice by qPCR analysis (Supplementary Fig. 10D). Consistent with our previous findings, we observed increased expression of these regulons in the MM stage compared with controls. Overall, our results suggest that MSC undergo a TF-mediated transcriptional shift at the MGUS stage that persists through disease progression.

### Altered crosstalk between EC, MSC, and PC during MM development

**Fig. 5:**
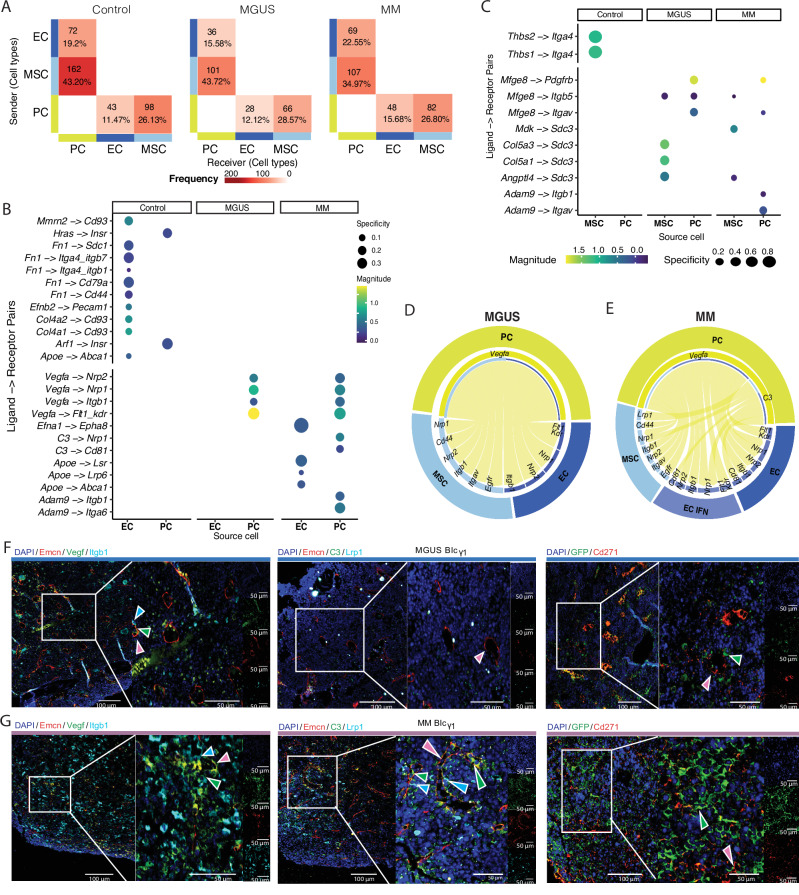
Altered crosstalk between EC, MSC, and PC during MM development. **A** Heatmap showing the total number and directionality of predicted L–R interactions between PC and niche populations (EC and MSC) across disease stages. Rows represent sender cell types and columns represent receiver cell types. Colors are proportional to the number of ligand-receptor pairs. **B**, **C** Dot plots of representative ligand-receptor interactions between PC and EC (**B**) or MSC (**C**) during disease progression. Dot size and color indicate the interaction’s specificity and strength, respectively. **D**, **E** Chord diagrams illustrating L-R pairs shared between PC–EC and PC–MSC communication within MGUS (**D**) and MM (**E**) stages. Colors and width represent signal senders and strength, respectively. PC-derived Vegfa engages multiple receptors in both stages (**D**, **E**), while C3-mediated interactions emerge in MM (**E**). **F**, **G** IF staining of PC–EC/MSC interactions on FFPE femur sections from MGUS BIcγ1 (**F**) and MM BIcγ1 mice (**G**). PC-derived ligands Vegf (left panel, green) and C3 (middle panel, green) are shown in PC (GFP⁺, green, right panel). The corresponding receptors Itgb1 (blue, left panel) and Lrp1 (blue, middle panel) are detected in EC (Emcn⁺, red, left and middle panels) and MSC (Cd271⁺, red, right panel). Representative IF images from *n* = 2 biologically independent mice for MGUS group (**F**) and 3 mice for MM (G) with similar results. Scale bars, 50 μm. Source data are provided as a Source Data file.

To further understand the mechanisms underlying BME-PC communication and how they may change upon disease progression, we analyzed cellular communication using LIANA ligand-receptor (L-R) analysis^28^ (Supplementary Fig. 11, Supplementary Data 6). We observed a decrease in the number of outgoing interactions from EC in the MGUS stage, reflecting the stress-associated phenotype of the cells (Fig. 5A and Supplementary Fig. 11A, B). Interactionsmediated by the *Fn1* ligand and the *Insr* receptor in EC and by the*Cd93* PC receptor were identified only in controls. In contrast, *Adam9* and *C3* PC ligands were only found in MM (Fig. 5B). Our results support the role of angiogenesis in myeloma via *Vegfa* secretion from PC, which interacts with *Kdr*, *Nrp1*, and *Nrp2* in EC. High expression of Nrps has been associated with tumor angiogenesis^29^.

Interestingly, analysis of MSC-PC communication revealed interactions associated with cell motility and adhesion in MM that may be related to malignant PC extravasation (Supplementary Fig. 11C and D). We observed a decrease in *Thbs1-Itga4* and *Thbs2-Itga4* interactions. In contrast, MGUS and MM PC participate in outgoing interactions involving *Mfge8* and *Sdc3*-mediated incoming interactions (Fig. 5C). Likewise, *Adam9*, which promotes cancer egress and invasion^30^, interacts with MSC through *Itgav* and *Itgb1* in MM.

We uncovered L-R pairs shared between EC-PC and MSC-PC communication across disease stages (Fig. 5D, E). Consistent with previous findings in human MM patients^12^, malignant PC engaged multiple receptors in EC and MSC through *Vegfa* secretion, supporting a pro-*angiogenic signaling* in MM. Additionally, shared *C3*-mediated interactions between PC and the non-hematopoietic BME emerged at the MM stage, warranting further investigation into whether *C3* crosstalk contributes to the maintenance of an immunosuppressive environment.

To further confirm the interactions identified in MGUS and MM BIcγ1 mice, we performed IF analysis on BM FFPE biopsies (Supplementary Fig. 11E and Fig. 5F, G). We observed that the PC-derived ligand *Vegfa* and the EC/MSC receptor *Itgb1* are expressed in close spatial proximity, suggesting functional crosstalk between these cell populations. In contrast, the spatial co-localization of the PC-derived ligand *C3* and its corresponding MSC receptor *Lrp1* was detected exclusively at the MM stage, thereby validating our previous findings from the scRNA-seq analysis.

In addition, we investigated cell–cell interactions in the MIcγ1 mouse model (Supplementary Data 6). Consistent with our previous findings, we detected pro-angiogenic signaling in the MM stage of this model, characterized by *Vegfa* secretion from PC and its interaction with multiple receptors expressed by EC and MSC (Supplementary Fig. 12A). To functionally validate the MM-associated interactions identified in MM MIcγ1 mice, we performed co-culture experiments using MM-PC (MM5080^15^) and MSC-mesenspheres^18^ derived from MM MIcγ1 mice. We specifically assessed the interaction between the PC-derived ligand *Vegfa* and its MSC-expressed receptors *Itgb1* and *Nrp1* using a proximity ligation assay (Navinci Kit) (Supplementary Fig. 12B, C), as well as conventional IF (Supplementary Fig. 12D, E). These experiments are consistent with the interactions predicted by our scRNA-seq analysis.

Taken together, despite the limitations associated with the sparsity of single-cell resolution data and the limited number of cells analyzed, the BME-PC interactions identified in the L-R-based analysis are consistent with known pathways associated with MM, further identifying potential targets in the disease.

### VRd induces transcriptional remodeling of EC and MSC in MM

**Fig. 6:**
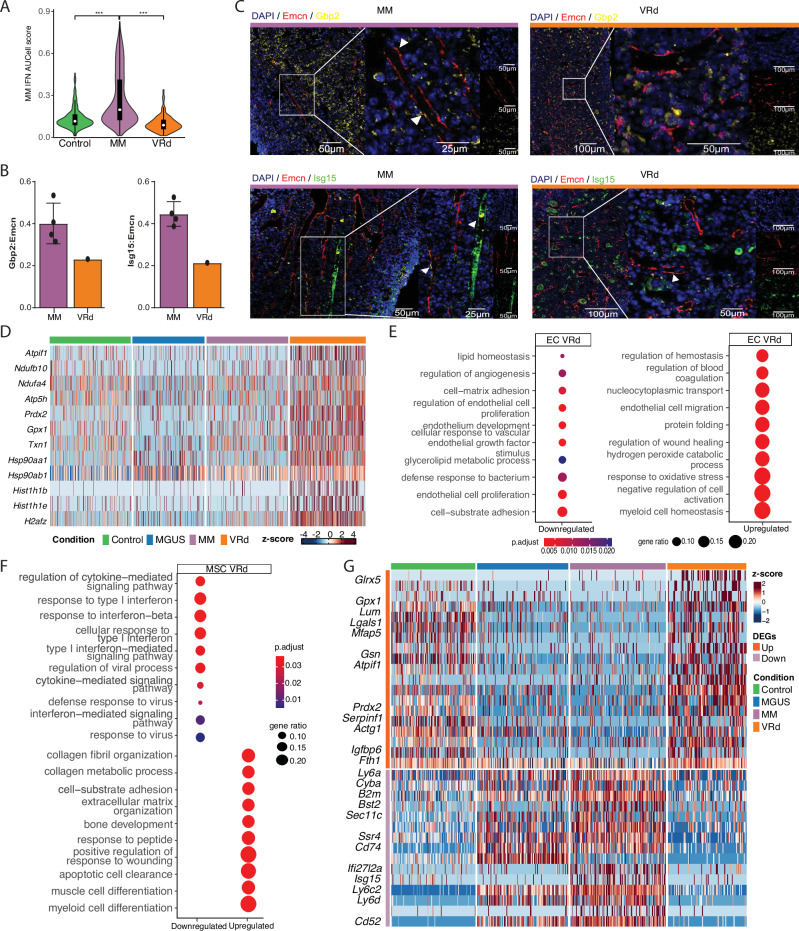
VRd induces transcriptional remodeling of EC and MSC in MM. **A** Violin plot showing AUC-score for IFN-related signature in EC from Control (YFPcγ1; *n* = 315 cells), MM BIcγ1 (MM; *n* = 2060 cells), and MM BIcγ1-CrbnI391V VRd-treated (VRd; *n* = 277 cells) mice. Box plots are overlaid: center line, median; box limits, first and third quartiles; whiskers, 1.5 × interquartile range. Pairwise comparisons were performed using two-sided Wilcoxon rank-sum tests. **B** Quantification of the percentage of endothelial IFN+ cells, determined by the coexpression of Emcn with IFN response genes (Gbp2 (left plot) and Isg15 (right plot) in femurs from MM BIcγ1 (MM; *n* = 4 biologically independent mice) and MM BIcγ1-CrbnI391V VRd-treated (VRd; *n* = 1 mouse). Data are mean ± SD. **C** IF staining of EC (Emcn, red), IFN response genes (Gbp2 (yellow, upper plot)); Isg15 (green, (bottom plot)), and nucleus (DAPI) (blue) in FFPE femurs from MM BIcγ1 (MM) and VRd-treated mice. White arrows point EC (Ecmn + ) IFN+ (Gbp2/Isg15). Representative images from biologically independent mice with similar results. Scale bars, 50 μm. **D** Heatmap showing the expression of stress-related DEGs identified in the comparison between treated and non-treated non-IFN MM EC at different stages of the disease. Color represents the z-score scaled expression values. **E** Significant terms derived from ORA between VRd-treated and untreated non-IFN MM EC. **F** Significant terms derived from ORA of MSC from MM mice before and after VRd treatment. **G** Heatmap projecting the expression of DEGs identified in the comparison between non-treated and treated MSC at different stages of the disease. Color represents the z-score scaled expression values. *P < 0.05; **P < 0.01; ***P < 0.001. Source data are provided as a Source Data file.

To assess the impact of MM therapy on EC and MSC molecular signatures, we performed scRNA-seq on a pool of BI_cγ1_ mice carrying humanized cereblon CRBN^I391V^ (BI_cγ1_-*Crbn*^I391V^)^31^ after 20 weeks of treatment with VRd, a current standard of care for patients with MM (Supplementary Fig. 13A–D). As expected, VRd treatment significantly reduced the tumor burden and improved survival outcomes (Supplementary Fig. 13E, F). Given the role of IFN signaling as a myeloma-specific response, we first sought to determine the presence of the IFN signature after treatment. Interestingly, VRd therapy downregulated IFN-driven MM signaling in EC and other BM populations (Fig. 6A, Supplementary Fig. 13G). As a result, EC from treated mice exhibited decreased expression of IFN-induced genes, including *Ifit1, Ifit2, Gbp2*, and *Isg15*, and of key related regulons previously upregulated during myeloma development (Supplementary Fig. 13H, Supplementary Data 7). IF analysis further confirmed the decrease in IFN^+^ EC and MSC following VRd treatment, supporting a potential link between supression of IFN signaling and the anti-myeloma drug response (Fig. 6B, C, Supplementary Fig. 13I–K). To further explore the significance of our findings, we analyzed publicly available bulk RNA-sequencing (bulk RNA-seq) data of MSC from MM patient samples before and after induction therapy with bortezomib-thalidomide-dexamethasone, with or without daratumumab^12^. This revealed a reduction of IFN- signature post-treatment in high-IFN MM patients (Supplementary Fig. 13L, M). Similarly, Chen et al^6^ reported a widespread downregulation of IFN-response genes in immune cells following bortezomib-cyclophosphamide-dexamethasone therapy, supporting the relevance of our results to human disease.

Beyond dampening the IFN signature, VRd treatment induced broader changes in gene expression in both EC and MSC. In EC, DEA revealed the upregulation of genes involved in mitochondrial metabolism, stress response, detoxification, and chromatin remodeling—indicative of an adaptive response (Fig. 6D, E). Consistent with its known tumor-suppressive role and previous studies in MM cells after bortezomib exposure, *Klf2* was upregulated in VRd-treated EC^32,33^ (Supplementary Fig. 13N). Furthermore, GRN analysis revealed enhanced activity of Zbtb7a and Rarg regulons, indicating activation of TF programs related to adaptive response^34,35^ (Supplementary Fig. 14A). Treated EC were enriched in thrombotic regulation pathways, including upregulation of *Hmgb1*, a gene linked to MM drug resistance^36^ (Supplementary Fig. 14B). Consistent with lenalidomide’s anti-angiogenic effects^37,38^, vascular and adhesion-related genes, including *Nrp1*, were downregulated, and Xbp1 regulon activity was reduced (Supplementary Fig. 14A and B).

In MSC, VRd treatment downregulated immune and IFN signaling pathways, indicating reduced inflammation in the BME (Fig. 6F). Likewise, antigen presentation genes, such as *Cd74*, *B2m*, and *Psmb8*, were downregulated (Fig. 6G, Supplementary Data 8). Interestingly, decreased expression of *Mdm4* and *Cyba* in treated MSC suggests that VRd may impair the proliferative supportive capacity of MSC^39,40^ (Supplementary Fig. 14C). On the contrary, genes linked to ECM organization, wound healing, and bone development were upregulated, aligning with bortezomib’s anabolic effects^41,42^ (Supplementary Fig. 14D). GRN analysis confirmed osteogenic reprogramming via higher regulon activity of Cebpa, Nfib, Nfic, Churc1 and Sox4 regulons (Supplementary Fig. 14E). Finally, MSC-PC communication analysis identified interactions previously observed in control MSC such as *Adam10–Tspan5*, *Adam17–Il6ra* and *Wnt10a–Fzd1/Lrp6* which may contribute to anti-inflammatory and osteogenic adaptations following VRd treatment^43–46^ (Supplementary Fig. 14F). Overall, our results suggest that VRd therapy is associated with suppression of IFN-driven MM signaling and transcriptional remodeling of the BM niche by triggering an adaptive response in EC and restoring MSC osteogenic potential.

### Transcriptional dynamics of human MSC during MM progression

**Fig. 7:**
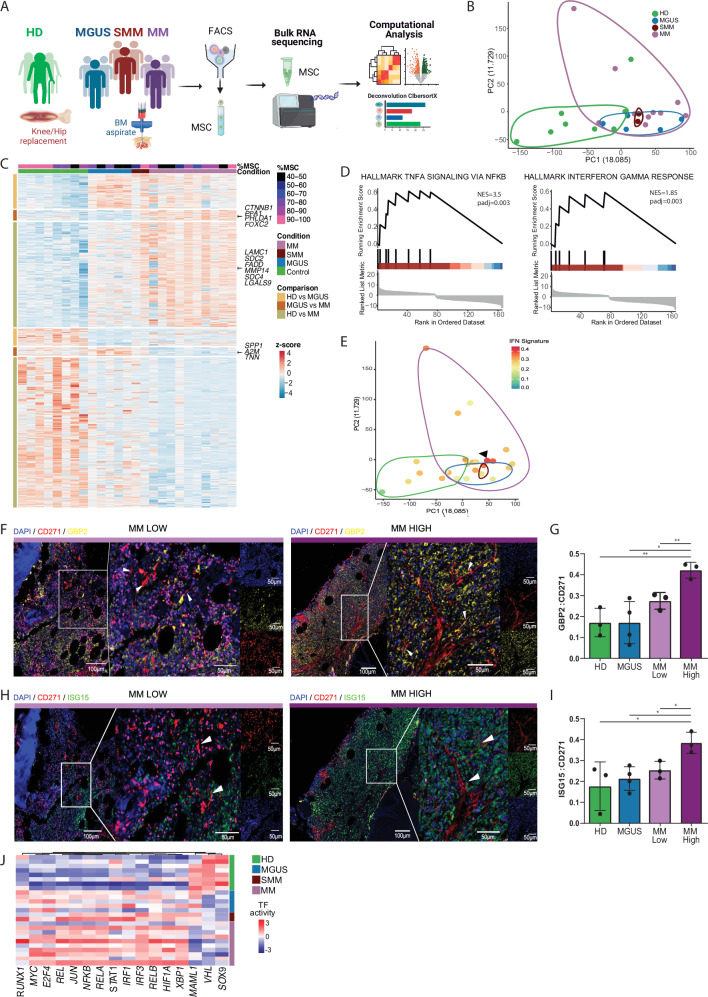
Transcriptional dynamics of human MSC during MM progression. **A** Experimental workflow for isolation and bulk RNA-seq analysis of human BM MSC. Created with BioRender.com. **B** PCA of MSC expression profiles shows clustering by disease stage. Each dot represents one sample. **C** Heatmap of DEGs identified in pairwise comparisons across disease progression. **D** GSEA plots for ‘TNFα signaling via NFκB’ (left) and ‘Interferon gamma response’ (right) comparing HD and MM MSC. **E** PCA showing the MM-IFN signature score projected onto each human sample. **F** IF staining of MSC (CD271, red), IFN (GBP2, yellow), and nucleus (DAPI) (blue) in human BM biopsies of MM patients with low and high IFN signal. White arrows point to MSC (CD271 + ) in MM LOW IFN samples, and MSC (CD271 + ) IFN+ (GBP2) in MM HIGH IFN samples. Representative images from independent patient biopsies with similar results. Scale bars, 50 μm. **G** Quantification of the percentage of mesenchymal IFN+ cells, determined by the coexpression of CD271+ with IFN (GBP2) in FFPE biopsies from HD (*n* = 3 donors) MGUS (*n* = 4 patients), and MM (*n* = 6 patients; low IFN, *n* = 3; high IFN, *n* = 3) biopsies. Data are presented as mean ± SD. Statistical significance was assessed using two-sided unpaired Student’s t-tests. **H** IF staining of MSC (CD271, red), IFN response genes (ISG15, green), and nucleus (DAPI) (blue) in MM samples with low or high stromal interferon signal. White arrows point to MSC (CD271 + ) in MM LOW IFN samples, and MSC (CD271 + ) IFN+ (ISG15) in MM HIGH IFN samples. Representative images from independent patient biopsies with similar results. Scale bars, 50 μm. **I** Quantification of the percentage of mesenchymal IFN+ cells, determined by the coexpression of CD271+ with IFN response genes (ISG15) in HD (*n* = 3 donors), MGUS (*n* = 4 patients), and MM (*n* = 6 patients; low interferon, *n* = 3; high interferon, *n* = 3) biopsies. Data are presented as mean ± SD. Statistical significance was assessed using two-sided unpaired Student’s t-tests. **J** Heatmap representing the variation of TF activity score across samples. *P < 0.05; **P < 0.01; ***P < 0.001. Source data are provided as a Source Data file.

To validate our findings in mice, we performed bulk RNA-seq in FACS-isolated MSC from aged-matched healthy donors (HD) (*n * =  8), newly diagnosed MGUS (*n * =  5), SMM (*n*  = 2), or MM (*n*  = 10) patients (Fig. 7A, Supplementary Fig. 15A). PCA revealed a distinction between MM-MSC and HD-MSC, with MGUS-MSC and SMM-MSC mapped between them (Fig. 7B).

The comparison between MGUS-MSC and HD-MSC revealed 180 DEGs (120 upregulated and 60 downregulated) (Fig. 7C, Supplementary Fig. 15B, Supplementary Data 9). Downregulation of genes related to osteoblast differentiation, such as *TNN, SPP1*, and *A2M*, indicates the early remodeling of MSC, as previously described in our mouse data. Additionally, MGUS-MSC upregulated genes involved in the cell cycle, protein ubiquitination, ATP metabolism, and oxidative stress, supporting their role in MM pathogenesis (Supplementary Fig. 15C).

Consistent with the high transcriptional similarity observed in mice between MGUS and MM-MSC, the fewest DEGs were yielded when comparing MGUS and MM-MSC (41 upregulated and 33 downregulated) (Fig. 7C, Supplementary Fig. 15D, Supplementary Data 10). MM-MSC showed enrichment in inflammatory processes, indicating that a pro-inflammatory microenvironment is required for the transition from premalignant stages to MM, in line with previous studies^12,13^. In addition, MM-MSC over-expressed *PHLDA1*, *FOXC2*, *PPA1*, and *CTNNB1*, genes associated with Epithelial-Mesenchymal Transition, which could be related to MSC´s potential to induce Epithelial-Mesenchymal Transition in MM^47^ (Supplementary Fig. 15D, E).

Defective osteogenic differentiation and dysfunctional immunomodulatory ability were confirmed when comparing MM- vs HD-MSC (448 upregulated and 566 downregulated) (Fig. 7C, Supplementary Fig. 15F, G, Supplementary Data 11). Furthermore, consistent with the impaired myogenic phenotype observed in mice, human MM-MSC showed downregulation of muscle-related genes. Gene set enrichment analysis (GSEA) revealed enrichment of TNF-α signaling via NF-ΚB and IFN response in MM-MSC (Fig. 7D). Interestingly, the IFN signature previously identified in murine MM BME was also enriched in MSC from a subset of MM patients (Fig. 7E and Supplementary Fig. 15H). A similar enrichment was observed in a subgroup of patients upon analysis of publicly available data from de Jong et al^12^. (Supplementary Fig. 5J, K, Supplementary Fig. 13L, M). IF staining of BM samples from MGUS and MM patients showed an enrichment of IFN-driven MM signaling in MSC from a subset of MM patients, further supporting our transcriptional findings (Fig. 7F-I, Supplementary Fig. 16A, B).

Finally, we determined changes in TF activity during myeloma progression (Fig. 7J). Enhanced activity of TFs involved in the NF-ΚB pathway, such as NFΚB and RELA, was observed in MSC from both MGUS and MM patients, highlighting NF-κB activation as an early event in the evolution of MM. Furthermore, MM-MSC exhibited increased activity of IRF1 and IRF3, which again emphasizes the importance of IFN signaling for the disease. In conclusion, the transcriptional changes of MSC between MSC from HD, MGUS, and MM patients mirror our findings in mouse models.

## Discussion

Over the last decades, our understanding of MM biology has advanced significantly^48–52^, explicitly revealing the contribution of the BME to the outgrowth and relapse of this hematological cancer and exerting a supportive function for the expansion of malignant PC^3,12,53–55^. However, research has mainly focused on PC and their immune microenvironment, highlighting the need for further exploration of the non-hematopoietic BME^9,10,14,56–59^. Here, using our validated BI_cγ1_ and MI_cγ1_ murine models^15^, which closely mimic key features of human MM progression, we provided a comprehensive overview of transcriptional deregulation in EC and MSC during the transition from healthy to MGUS and MM as well as the reshaping of the non-hematopoietic BME in response to VRd treatment. Further, we validated our results in MSC from MM patients across various disease stages, both in our data as well as in the only publicly available single-cell MM patient dataset^12^.

Through analysis of transcriptomic alterations along disease progression, we uncovered a stress state in MGUS EC linked to protein folding and ion homeostasis, in which upregulation of *Slpi*, *Mt1*, *Mt2*, and *Hspa5* could serve as an early myeloma fingerprint in EC. Disease progression from MGUS to MM in EC involved angiogenesis, migration, and metabolic shifts. Xbp1 and Fos, key regulators of vascular development and cancer progression^20,21^, showed enhanced activity during myeloma progression, highlighting their potential as disease targets. In contrast, transcriptional changes in MSC previously described in advanced MM patients^8,11^, were already present during the precursor MGUS-like stage. In addition to impaired osteogenic differentiation, MGUS MSCs exhibited a downregulation of myogenesis-related genes. Consistent with these murine findings, our bulk RNA-seq showed impaired MSC differentiation in human patients, even at premalignant stages. Similarly, MGUS prompted a metabolic shift in human MSC with increased protein folding. These findings suggest that MGUS MSCs show a reshaping of their differentiation capacity and metabolism from the early stages of the disease that may contribute to a permissive microenvironment for MM cells.

Understanding the crosstalk between the BME and PC is crucial for unraveling mechanisms of tumor progression; this study contributes to elucidating the interactions between EC, MSC, and malignant PC throughout the disease evolution. Consistent with a previous study in MM patients^12^ we found *Vegfa* secretion from PC to be a hallmark of MM angiogenesis through interaction with *Kdr*, *Nrp1*, and *Nrp2* endothelial receptors. MSC-PC interactions were associated with adhesion, matrix organization, and migration, suggesting that MSC-PC crosstalk may be linked to MM extravasation and disease dissemination.

A fundamental finding of our study is the identification of a myeloma-specific IFN signature in EC that extends to MSC and other BM cells, indicating a shared BME response during MM progression. This signature, which differs from the inflammatory phenotype characterized by TNFα and NF-κB signaling described by de Jong et al. ^12^ is marked by upregulation of IFN-inducible genes and master regulators (Irf7, Stat1, Stat2). STAT1/2 signaling, closely linked to IFN in MM, supports tumor-promoting roles, complementing *STAT3*’s known oncogenic function in MM migration and BM homing^60^. While prior scRNA-seq studies reported IFN activity in PC and immune cells^3,4^, only limited evidence suggested an IFNα response in MSC^9,56^. Considering the reported role of *Isg15* in modulating IL-12 release and Th1 differentiation, IFN-responsive stromal cells may influence local Il-12 availability and Th1 polarization^61^, thereby contributing to immune modulation within the non-immune microenvironment of MM. In addition, our observation of C3-mediated interactions between PC and EC or MSC further supports the presence of multiple immunoregulatory pathways operating within non-immune MM. Together, the MM IFN-inducible response uncovered in our study supports an immunomodulatory role for the non-hematopoietic BME, with EC and MSC potentially contributing to immune remodeling and T-cell exhaustion under inflammatory conditions^17^.

Our findings expand on the concept of pro-inflammatory signaling within MM MSC^12^, identifying an enrichment of this IFN signature in both EC and MSC from a subset of MM patients, highlighting its clinical relevance for patient stratification based on inflammatory features. More importantly, the suppression of this MM-IFN signature in human MM patients after induction therapy further supports its clinical relevance. In addition, using our MGUS-MM mouse models, we demonstrate that VRd treatment impacts the non-hematopoietic BME by suppressing this MM IFN signaling. While combinatorial regimens improve patient outcomes by targeting multiple pathways, our results support their potential to reshape the BME^62^ by inducing adaptive stress in EC and restoring MSC osteogenic potential, thereby shifting the niche toward a less tumor-permissive state. These findings highlight the importance of targeting BME interactions and enhancing anti-myeloma immune responses^63–65^.

In summary, the use of the BI_cγ1_ murine myeloma model, which mimics MGUS-to-MM progression, alongside scRNA-seq, has enabled a comprehensive dissection of the non-hematopoietic BME. The myeloma-specific IFN signature, the transition stress state in MGUS EC, the early transcriptional shifts in MSC, the evolving cellular communication networks, and the reperssion of IFN signaling post-VRd therapy collectively support the role of EC and MSC in MM development and treatment response. Validation in human patients reinforces the study’s translational relevance, suggesting that non-hematopoietic BME changes may foster MM development from premalignant stages and shift toward a less tumor-permissive state after treatment.

## Methods

All research complied with all relevant ethical regulations. Animal experiments were approved by the Ethics Committee for Animal Experimentation of the University of Navarra (protocol CEEA082-20) and were conducted in accordance with institutional and national guidelines. Human sample collection and analysis were approved by the University of Navarra Institutional Review Board (Project 2022.100mod1) and the Navarra Department of Health Ethics Committee for Drug Research (CEIm, PI_2022/92 MS-1), and all participants provided written informed consent in accordance with the Declaration of Helsinki.

### Mice

A detailed description of all mice utilized in the study is provided in Supplementary Data 12. We used BI_cγ_ mice (B6(Cg)-Gt (ROSA)26Sor^tm4(Ikbkb)Rsky^/J mice and B6. Cg-Tg (BCL2)22Wehi/J mice crossed with cγ1-cre mice (B6.129P2(Cg)-Ighg1^tm1(cre)Cgn^/J mice; all from The Jackson Laboratory) that recapitulate the most common changes observed in human MM, preceded by a MGUS-like stage^15^. In addition, we used MI_cγ_ mice (B6(Cg)-*Gt (ROSA)26Sor*^*tm4(Ikbkb)Rsky*^/J and C57BL/6N-Gt (ROSA)26Sor^tm13(CAG-MYC, -CD2*) Rsky^/J mice crossed with cγ1-cre mice), a fast progression model with a less marked MGUS stage. As controls, mice carrying a YFP reporter (B6.129 × 1-Gt (ROSA)26Sor^tm1(EYFP)Cos^/J mice; The Jackson Laboratory) were crossed with cγ1-cre mice. To induce PC generation in mice kept in specific pathogen-free (SPF) animal facilities, eight-to ten-week-old mice were immunized with sheep red blood cells (SRBCs) every 21 days for 3-4 months.

To assess the role of the BME in treatment response, we used the triplet therapy VRd of bortezomib (Velcade®), lenalidomide (Revlimid®), and dexamethasone on BI_cγ1_, with regimens optimized based on human protocols^66^. Given mouse cells´ inherent resistance to immunomodulatory drugs (IMiDs), BI_cγ1_ mice were crossed with a humanized *Crbn* ^I391V^ gene strain, enabling sensitivity to IMiDs^15,31,67^. Before initiating therapy, tumor burden was assessed by quantifying the serum immunoglobulin gamma fraction (M-spikes) by electrophoresis. Treatment was administered upon the emergence of M-spikes. Mice received 1 mg/kg (Monday and Friday) of bortezomib (Velcade®), lenalidomide (Revlimid®), 50 mg/kg (Monday, Wednesday and Friday), and dexamethasone, 20 mg/kg (Monday) for 20 weeks.

For each scRNA-seq experiment, a pool of four, five, or six adult mice per group comprising control, MGUS, and MM stages, as well as VRd-treated subjects, was used. Mice of both sexes were used in the study. Mice were kept under SPF conditions in the Center for Applied Medical Research (CIMA) animal facilities at the University of Navarra. Animals were maintained on a 12-h light/dark cycle at a constant temperature of 18 °C–23 °C.

All animal procedures were conducted in accordance with Directive 2010/63/EU and Spanish Royal Decree 53/2013 and were approved by the Animal Care and Use Committee of the University of Navarra (protocol CEEA082-20). As this study used a disseminated hematological malignancy model rather than solid tumors, maximal disease burden was defined by pre-established humane endpoint criteria rather than tumor size. Mice were monitored at least twice weekly for signs of disease or distress, including hunching, ruffled fur, labored breathing, reduced mobility, hypothermia, or >20% body weight loss. No animals exceeded the approved humane endpoint limits, and those that met endpoint criteria were euthanized by CO2 asphyxiation.

### Isolation and fluorescence-activated cell sorting of murine BMM and PC

Mice (Supplementary Data 12) were euthanized by CO_2_ asphyxiation. Bones from the humerus, radius, iliac crests, femurs, and tibia were harvested in Phosphate-buffered saline (PBS) 1X containing 2% fetal bovine serum (FBS) and two mM EDTA (modified PBS). All steps were performed on ice to preserve cell viability and RNA integrity. Muscles and soft tissue were removed from the bones, and BM cells were obtained by crushing in modified PBS. Cells were then filtered through a 70 μm cell strainer, and red blood cells were lysed with ACK buffer (NH_4_Cl 150 mM, KHCO_3_ 10 mM, and Na_2_EDTA 0,1 mM) for 10 min at room temperature (RT). 25 μl were stained with PE-CD138, APC-B220, and BV421-IgM antibodies (Biolegend) and analyzed by flow cytometry to determine PC infiltration (Supplementary Data 12). The remaining bone fragments were collected in a 50 ml conical tube, digested with the appropriate volume of PBS (3 ml/mouse) containing 0.3% collagenase I and dispase (5 U/ml) for 15 min at 37 °C with shaking at 200 rpm. FBS, representing 10% of the digestion volume, was added to stop collagenase digestion. After digestion, the collagenized fractions were filtered through a 70 μm filter into a collection tube and pooled into one sample. To determine live-cell concentration and viability, 10 μl of each mouse sample was stained with and acridine orange/propidium iodide (AO/PI) solution (Nexcelom) and analyzed using a Cellometer K2 Image Cytometer (Nexcelom Bioscience). Cells were subsequently stained for 20 minutes on ice first in the appropriate volume of modified PBS 1X (3 ml/mouse) with 160 μl/mouse of biotinylated lineage cocktail (Mac1, CD3, Gr1, B220, and TER119) followed by incubation with streptavidin magnetic microbeads (100 μl/mouse). Negative selection was performed using Miltenyi LD columns according to the manufacturer’s protocol, and the resulting cells from both fractions were counted as described before to confirm selection efficiency.

After selection, the negative fraction was stained with the following combination of conjugated antibodies at a concentration of 1/200: APC-Cy7 labeled streptavidin, BV510 labeled anti-CD45, APC labeled anti-CD45, and APC labeled anti-Ter119. Samples were then stained with 0.05 μM Vybrant dye orange (VDO) at 37 °C for 30 min to label living cells. Cells were stained with 5 μL of 7AAD dye (up to 1×10^6^ cells) to discard apoptotic and dead cells from the sample.

BM non-hematopoietic cells and GFP^+^ PC were FACS using a BD FACSAria II sorter (supplementary Fig. 1). and collected in PBS 1X supplemented with 0.05% UltraPure BSA for the subsequent sequencing protocol. Cell viability of sorted cells was assessed using an Nexcelom Cellometer, as described above.

### Single-cell RNA sequencing

scRNA-seq was performed using the Single Cell 3’ Reagent Kits v3.1 (10X Genomics) according to the manufacturer’s instructions. PC and BME sorted cells for each sample were pooled before scRNA-seq was performed. Approximately 15,000 cells were loaded at a concentration of 1000 cells/µL on a Chromium Controller instrument (10X Genomics) to generate single-cell gel bead-in-emulsions (GEMs). In this step, each cell was encapsulated with primers containing a fixed Illumina Read one sequence, followed by a cell-identifying 16-bp 10X barcode, a 12-bp Unique Molecular Identifier (UMI), and a poly-dT sequence. A subsequent reverse transcription yielded full-length, barcoded cDNA. This cDNA was then released from the GEMs, PCR-amplified, and purified with magnetic beads (SPRIselect, Beckman Colter). Enzymatic Fragmentation and Size Selection were used to optimize cDNA size before library construction. Illumina adapter sequences were added, and the resulting library was amplified via end repair, A-tailing, adapter ligation, and PCR. Libraries’ quality control and quantification were performed using Qubit 3.0 Fluorometer (Life Technologies) and Agilent’s 4200 TapeStation System (Agilent), respectively. Sequencing was performed in a NextSeq2000 (Illumina) (Read 1: 28cycles, i7 Index: 10cycles, i5 Index:10 Read 2: 90cycles) at an average depth of 45,000 reads/cell followed by computational alignment using CellRanger (version (v) 6.1.1, 10x Genomics).

### Single-cell RNA sequencing analysis

The scRNA-seq analysis of the BM samples was performed using R v 4.1.3 and Seurat v 4.3.0^68^. For data preprocessing, ambient RNA detection was performed using SoupX v.1.6.2^69^, and doublet scores were calculated by scDblFinder v.1.8.0^70^. Datasets were filtered individually based on a library complexity of more than 200 features, features detected in more than three cells, doublets, and high percentages of mitochondrial genes >5%). Because the exploratory data analysis revealed potential ambient RNA contamination, immunoglobulin genes were excluded from the downstream analysis of niche cells.

For the transcriptomic analysis during disease progression, the Control, MGUS, and MM cell datasets were merged, normalized using the SCTransform method^71^, and analyzed by principal-component analysis (PCA) on the most variable genes (k = 2000) across all cells. With the principal components (PCs) (k = 30), unsupervised clustering was performed by computing the K-nearest neighbors, applying the Louvain algorithm at resolution 0.4, and cells were projected in two dimensions using UMAP^72^. Significantly upregulated genes in each cluster compared to all other clusters (Bonferroni-adjusted p-values < 0.05) were identified using the Seurat Function FindMarkers. Manual cell type annotations identified cell types according to published canonical marker genes. The same analytical approach was applied to analyze the VRd treatment dataset individually.

To characterize BM cells at a high resolution, EC and MSC were subsetted into separate objects. Additionally, outlier cells within EC and MSC, lacking canonical marker genes for their respective population, were excluded from downstream analysis.

### Differential expression analysis

DEGs were determined with the Seurat FindMarkers function for transcripts detected in at least 30% of cells using a log2FC threshold of 0.5 with MAST (Model-based Analysis of Single-cell Transcriptomics) methodology^73^. An adjusted *P*-value threshold of 0.05 based on Bonferroni multiple testing correction was used as a cutoff to select significant DEGs. Significant DEGs for each group were used as input for a GO ORA using the clusterProfiler R package (v 4.14.6)^74^ to investigate the biological differences between groups, GO terms with corrected *P*-values less than 0.05 were considered significantly enriched. GSEA was also calculated using the GO and Hallmark gene sets from the Molecular Signatures Database v.7.5.1 in the list of DEGs ranked according to log2FC. Heatmap plots were generated using the SCP package v.0.5.4^75^.

As samples were pooled prior to sequencing without hashing or barcoding, analyses were restricted to stage-associated transcriptional signatures rather than statistical inference at the level of individual animals.

### Signature score

The MM IFN-signature score was computed using Seurat’s AddModuleScore function using the top 50 DEGs of the MM2 EC subgroup calculated with the Seurat FindMarkers function (parameters min.pct=0.4 thresh.use=1, test = ’MAST’). This signature score was applied across all cell types, including the treatment dataset, to assess IFN pathway activation. The resulting score provides a quantitative measure that reflects how strongly a particular gene expression profile—often derived from a set of genes associated with a biological process or cell state—is expressed in a given cell or population. Unlike MM2 EC, which formed a clearly separated cluster based on their transcriptomic profile, MM MSC with elevated IFN signaling were defined based on a signature score exceeding a threshold of 0.25.

### Sex inference

To ensure that the identified cluster was not biased by unequal contributions from individual mice—particularly since both sexes were included in the pooled samples—we inferred the sex of origin for each cell using two complementary approaches (Supplementary Fig. 3). First, PCA was computed using (i) the expression of sex-specific genes and (ii) exclusively Y chromosome–encoded genes. Both analyses revealed separation of cells by sex, as denoted by the segregation of cells with more than one count for Y-linked genes. Second, we calculated the expression ratio of Y-linked genes (*Uty, Eif2s3y*) to X-linked genes (*Tsix, Xist*) to assign the most likely sex of origin to each cell following the strategy previously described^76^. Together, these analyses confirmed that both male- and female-derived cells contributed to all EC and MSC states, supporting that the observed cluster represents pooled contributions from multiple animals rather than being dominated by any single individual or sex.

### Gene regulatory network analysis

GRN activity was interrogated following the workflow implemented by SCENIC (v 1.3.1)^16^. This approach identifies regulons, defined as sets of genes co-expressed with and predicted to be regulated by a given TF. Thereby capturing coordinated transcriptional programs rather than individual genes.

To ensure comparability across conditions, an equal number of cells per condition and cell type was randomly selected for the analysis. Briefly, all the genes were trained in the GENIE3 package and used to develop a random forest model for selecting co-expression modules between TFs and target genes (regulons). Regions for TFs searching were restricted to a 10-k distance centered on the transcriptional start site (TSS) or 500 bp upstream of the TSSs. Predicted TF–target relationships were then refined using RcisTarget to retain only targets supported by enrichment of the corresponding TF-binding motifs. Finally, regulon activity was calculated for each cell using the AUCell package, generating per-cell activity scores that reflect whether a given regulatory program is active or inactive.

To identify candidate regulators of MM2 IFN EC, GRN inference was first computed by comparing MM2 EC to non-IFN MM EC and subsequently extended to include all EC across disease stages (Fig. 2D; supplementary Fig. 4A–C).

### Cell-to-cell communication analysis

To assess receptor-ligand interactions between the two BM niche populations (EC and MSC) and the myeloma PC, we used Liana v.0.1.13^28^ with five different methods for predicting cell-cell interactions (cellphonedb, connectome, NATMI, logFC, and SingleCellSignalR). The results only retain interactions specifically between one cell type and another while excluding interactions within each cell type. The liana_aggregate function was used to generate an aggregate rank score for each interaction, reflecting only the specificity of interactions, and interactions were filtered based on their statistical significance (*p*-value < 0.01). Further bootstrapping analysis was conducted, and an equal number of cells were selected from each cell type. Subsequently, the LIANA algorithm was employed to predict interactions, iterating this process 100 times for robustness. The outcomes were assessed based on the percentage of predictions for each interaction across these iterations. The OmnipathR R package^77^ was used to import functional annotations for all genes involved in ligand-receptor interactions. To illustrate the strength of specific interactions between different groups, several ligand-receptor pairs with high mean values were selected for visualization.

### Immunofluorescence staining of mice BM samples

Femurs were collected from control (YFP_cγ1_), MGUS (BI_cγ1_), MM (BI_cγ1_ and MI_cγ1_), alongside VRd-treated humanized BI_cγ1_-Crbn^I391V^ mice after 20 weeks of therapy. Bone tissue sections were fixed in formol 4% (PanReac) for 24 h at 4 °C and then decalcified using EDTA 0.25 M pH 6.95 (Invitrogen) for 10 days at 4 °C with agitation. Following decalcification, the samples were washed in distilled water for 5 min and sequentially dehydrated in ethanol at increasing concentrations: 70% for 1 h, 80% for 1 h, 96% for 1 h, and 100% overnight. The samples were then cleared in xylol (PanReac) for 4 h. Fixed bone samples were subsequently embedded in paraffin and incubated at 60 °C overnight. Subsequently, 4 µm tissue sections were mounted on microscopy slides and dried in a desiccator at 37 °C overnight. The preparations were deparaffinized and rehydrated through a series of graded alcohols. Antigen retrieval was carried out at 95 °C for 30 min in either 10 mM citrate buffer (pH 6.0) or, specifically for CD271 staining combinations, in 10 mM Tris-1 mM EDTA buffer (pH 9.0).

For IF staining, the FFPE sections were blocked with BSA 5% for 30 min at RT. The following primary antibodies were used: rat anti-Endomucin antibody (V.7C7) (Santa Cruz Technology 1: 50 dilution), mouse anti-CD271 monoclonal antibody (NGF Receptor) (ME20.4) (Invitrogen, 1: 50 dilution), rabbit GBP2 Polyclonal Antibody (Proteintech, 1: 100 dilution), rabbit ISG15 Polyclonal Antibody (Proteintech, 1: 100 dilution), VEGF Monoclonal Antibody (JH121) (Invitrogen, 1: 20 dilution), Integrin Beta 1/CD29 Polyclonal antibody (Proteintech, 1: 200 dilution), Complement C3 Polyclonal Antibody (Invitrogen, 1: 100 dilution), Anti-LRP1 antibody (abcam, 1: 50 dilution), CD304 (Neuropilin-1) Recombinant Rabbit Monoclonal Antibody (ST05-30) (Invitrogen, 1: 50 dilution) and Goat anti-GFP (Novus Biologicals, 1:4000).

The slides were subsequently incubated with the corresponding secondary antibodies in a 1:200 dilution: Goat anti-Rabbit IgG (H + L) Highly Cross-Adsorbed Secondary Antibody, Alexa Fluor™ 647 (Invitrogen); Goat anti-Rabbit IgG (H + L) Highly Cross-Adsorbed Secondary Antibody, Alexa Fluor™ 488 (Invitrogen); Goat anti-Rat IgG (H + L) Cross-Adsorbed Secondary Antibody, Alexa Fluor™ 568; Goat anti-Mouse IgG (H + L) Highly Cross-Adsorbed Secondary Antibody, Alexa Fluor™ Plus 488; Goat anti-Rabbit IgG (H + L) Cross-Adsorbed Secondary Antibody, Alexa Fluor™ 488, Goat anti-Mouse IgG (H + L) Cross-Adsorbed Secondary Antibody, Alexa Fluor™ 568 (Invitrogen), Donkey anti-Mouse IgG (H + L) Highly Cross-Adsorbed Secondary Antibody, Alexa Fluor™ 555 (Invitrogen), Donkey anti-Goat IgG (H + L) Cross-Adsorbed Secondary Antibody, Alexa Fluor™ 488 (Invitrogen). Nuclei were stained with DAPI (Vectashield 1:10 dilution). Fluorescence images were acquired using the Vectra Polaris Multispectral Imaging System (Perkin Elmer).

### Mesensphere isolation and culture

Mesenspheres were generated from mouse primary BM cells as previously described by Forte et al^18^. Clean mouse bones were crushed in a mortar with 2 ml of 0.25% Collagenase Type I (Stem Cell Technologies). The resulting suspension was incubated for 45 min at 37 °C in agitation. The enzymatic reaction was quenched with PBS + 2% FBS, and cells were filtered through a 40 μm cell strainer. The erythrocytes in the cell suspension were lysed by incubation for 10 min with RBC Lysis Buffer at RT. Then, erythroid and hematopoietic lineages were magnetically depleted. Therefore, cells were incubated with biotin-conjugated primary antibodies against Cd45 and Ter119 (BD Biosciences, 1:100), followed by incubation with streptavidin-conjugated magnetic beads (BD Biosciences). The negative fraction was plated at low density (<500,000 cells/cm2) in ultralow-adherence 35 mm dishes (StemCell Technologies) previously coated with Poly-Hema (Sigma). The growth medium for spheres contained 0.1 mM β-mercaptoethanol; 1% nonessential amino acids (Sigma); 1% N_2_ and 2% B27 supplements (Invitrogen); recombinant human fibroblast growth factor (FGF)-basic, recombinant human epidermal growth factor (EGF), recombinant human platelet-derived growth factor (PDGF-AA), recombinant human oncostatin M (227 aa OSM) and recombinant human IGF-1 in Dulbecco’s modified Eagle’s medium (DMEM)/F12 (1:1) / human endothelial (1:2) serum-free medium (Invitrogen). All cytokines (Preprotech) were added at a final concentration of 20 ng/ml, except for IGF-1, which was added at 40 ng/ml. Additionally, mesenchyme medium was supplemented with 15% chicken embryo extract prepared as described previously^78^. Fertilized chicken eggs were incubated for 11 days at 38 °C in a humidified incubator. Embryos were washed with DMEM (Invitrogen), macerated by passage through a 50 ml syringe, and diluted 1:1 in the same medium. Hyaluronidase (2 mg, Sigma) was added to each 50 ml tube and incubated for 1 h at 4 °C with agitation. Following 6 h ultracentrifugation (3 × 10^4^ g) at 4 °C was decanted and filtered with 0.45 and 0.22 μm sterile filters (Millipore). Aliquots were stored at −80 °C until use. The cultures were incubated at 37 °C with 5% CO_2_, 20% O_2_ in a water-jacketed incubator and left untouched for 1 week. Afterward, half-medium changes were performed twice a week. Following a 14-day incubation period, the resulting spheres were harvested for downstream applications, including RNA isolation and co-culture experiments. The mesenspheres used in this paper were obtained from control mice (4 months old) and MM mice. (BIc_γ1_ and MIc_γ1_, 6–12 months old). Tumor burden was quantified by flow cytometry as the percentage of PC (GFP^+^) within the total BM population. For all MM-derived MSC mesenspheres cultures, mice were selected based on a confirmed tumor burden of 3–8% PC. For each independent experiment, mesenspheres were generated from a pool of BM cells harvested from three mice.

### RNA isolation and qPCR

Total RNA was isolated using the MagMAX mirVana Total RNA Isolation Kit (Applied Biosystems, Thermo Fisher Scientific) according to the manufacturer’s instructions and finally resuspended in 14 µL of Elution Buffer (EB) provided with the kit. RNA concentration was determined using the Qubit 3.0 Fluorometer with the Qubit RNA HS Assay Kit (Thermo Fisher Scientific), and RNA integrity and quality were assessed using the Agilent 4200 TapeStation System (Agilent Technologies), following the manufacturers’ protocols. Reverse transcription was performed using PrimeScript RT reagent Kit (Takara, Kusatsu, Japan), following the manufacturer’s recommendations. qPCR was performed using the PowerUp SYBR Green Master Mix (Applied Biosystems, Waltham, MA, USA) and QuantStudio 5 Real Time PCR System (Thermo Fisher Scientific, Waltham, MA, USA). To quantify gene expression ΔΔCt method was employed. Gene expression was normalized to the housekeeping gene *Gapdh* to calculate ΔCt. Then ΔΔCt values were obtained by comparing ΔCt the samples of interest to ΔCt mean of two control samples (ΔCt sample – Control mean ΔCt). Final expression levels were calculated as 2^-ΔΔCt and subsequently transformed to log2(2-ΔΔCt) for statistical analysis and comparison against the control baseline. The primer sequences were as follows: mouse *Stat1*^79^ forward: GAACGCGCTCTGCTCAA; reverse: TGCGAATAATATCTGGGAAAGTAA. Mouse *Gbp2* (Origene, NM_010260) forward: AGATGCCCACAGAAACCCTCCA; reverse: AAGGCATCTCGCTTGGCTACCA. Mouse *Yy1*^80^ forward: TCAGACCCTAAGCAACTGGCAGAA; reverse: TTGAGCTCTCAACGAACGCTTTGC. Mouse *Xbp1*^81^ forward: GGGTCTGCTGAGTCC; reverse: CAGACTCAGAATCTGAAGAGG. Mouse *Elk3* (Origene, NM_013508) forward: ACACTTCTGGAGCAGCCTTAGTC; reverse: CATGTGACCGTTGAGCAGTGTG. Housekeeping gene, mouse *Gapdh*^82^ forward: AGTGGCAAAGTGGAGATT; reverse: GTGGAGTCATACTGGAACA.

### Coculture and cell–cell interaction imaging

To evaluate interactions between MM-PC and MSC, BM mesenspheres derived from MM MIc_γ1_ mice were co-cultured with the murine MM cell line MM5080^15^. This cell line was previously established from an original MM developed in P53-BIcγ1 mice^15^.

For imaging experiments 7,5×10^4^ PC were co-cultured with 60 mesenspheres in a µ-Slide 8 Well ^high^ ibiTreat (Ibidi). Cultures were incubated for 24 h at 37 °C in a water-jacketed incubator with 5% CO_2_. Following incubation, adherent spheres were washed with PBS to remove non-adherent cells and fixed with formol 4% (PanReac) for 20 min at RT. Fixed cells were incubated in a blocking buffer (PBS containing 5% BSA and 0.1% Tween-20) for 1 h at RT. Primary antibodies against Vegf (VEGF Mouse monoclonal Antibody (JH121) (Invitrogen, 1: 20 dilution)), Itgb1 (Integrin Beta 1/CD29 Rabbit polyclonal antibody (Proteintech, 1: 200 dilution)), and Nrp1(CD304 (Neuropilin-1) Recombinant rabbit monoclonal antibody (ST05-30)) were diluted in PBS-0.1% Tween-20 and incubated overnight at 4 °C. At this point, we performed a standard IF and a proximity ligation assay (PLA). For IF, samples were incubated with the following secondary antibodies (1:200 dilution): Goat anti-Rabbit IgG (H + L) Highly Cross-Adsorbed Secondary Antibody, Alexa Fluor™ 647 and Goat anti-Mouse IgG (H + L) Highly Cross-Adsorbed Secondary Antibody, Alexa Fluor™ Plus 488 (both from Invitrogen). Nuclei were counterstained with DAPI (Invitrogen; 1:1000).

On the other hand, to assess the proximity of specific ligand-receptor pairs, the NaveniFlex Cell Atto647N kit (Navinci) was used according to the manufacturer’s instructions. This assay used the previously bound mouse and rabbit primary antibodies (Vegf-Itgb1/Vegf-Nrp1) to detect specific ligand-receptor interactions. Images were recorded with a Confocal Scanning Laser Microscope (Zeiss LSM 800).

### Human

For human study, clinical BM aspirate samples from individuals of both sexes with newly diagnosed MGUS (*n * =  5), SMM (*n * = 2), or MM (*n * = 10) were obtained from the University of Navarra Biobank. Human BM samples from healthy adult donors (50–80 years of age), healthy donors (HD) (*n* = 8) undergoing orthopedic surgery (hip or knee replacement) from Hospital Universitario de Navarra (HUN). For IF staining, archived paraffin-embedded human BM biopsies of MGUS (*n* = 4) and MM (*n* = 6) patients were obtained from the Pathology Department of Clínica Universidad de Navarra (CUN). Bone chips from HD (*n* = 2) undergoing orthopedic surgery (hip or knee replacement) from HUN and CUN were also used as controls. The clinical characteristics of all individuals are described in Supplementary Data 13. All samples were collected, processed, banked, and thawed for further processing. This study was approved by the University of Navarra’s Institutional Review Board (Project 2022.100mod1) and the Navarra Department of Health Ethics Committee for Drug Research (CEIm, PI_2022/92 MS-1) and was conducted under the Declaration of Helsinki. Informed consent was obtained from all patients. Personal data was kept confidential following the Organic Law 3/2018 on personal data protection and Spanish Law 14/2007 on Biomedical research. All collection samples are codified; only authorized personnel can correlate the patient’s identity with the codes.

### Isolation and fluorescence-activated cell sorting of human BM mesenchymal-osteolineage primed cells

After sample collection, red blood cells were lysed twice for 15 min at room temperature with rotation, using a ratio of 45 ml of ACK lysis buffer to 5 ml of human sample. In the case of samples from healthy adult donors, portions of bones present in the samples were cut into small pieces, and after being crushed, the remaining fragments were collagenized with the appropriate volume of PBS (3 ml/sample) with 0.3% collagenase I and dispase (5U/ml) for 15 min at 37 °C and shaking at 200 rpm. FBS, representing 10% of the digestion volume, was added to stop the collagenase digestion. After digestion, the collagenized fractions were filtered through a 70 μm filter into a collection tube and pooled into one sample. To determine live-cell concentration and viability, 10 μl of each sample was stained with an acridine orange/ propidium iodide (AO/PI) solution (Nexcelom) and analyzed using a Cellometer K2 Image Cytometer (Nexcelom Bioscience). Cells were subsequently cryopreserved with FBS and 10% dimethyl sulfoxide (DMSO, Sigma-Aldrich) in liquid nitrogen until use.

Frozen BM samples were thawed at 37 °C in dextran-40 solution supplemented with 25% human albumin at 20%. Cell viability of thawed cells was assessed using Nexcelom Cellometer as described above. The sample was then centrifuged and stained for 30 min on ice with the following combination of conjugated antibodies: BV510-labeled anti-Lin (including CD3, CD19, CD45 and CD64), BV421-labeled anti-CD235, BV421-labeled anti-CD45, FITC-labeled anti-CD16, PE-Cy7-labeled anti-CD56, PE-labeled anti-CD31, APC-Cy7-labeled anti-CD9, and PerCP-Cy5.5-labeled anti-CD271. All antibodies were added at a concentration of 1/100 except anti-Lin, where we added 3 μl/test- test 25x106cells, anti-CD16, 10 μl/test- test 25x106cells and 1/50 anti-CD31 and anti-CD56. First, dead cells and debris were excluded by FSC, SSC, and adding 10 μl of TO-PRO-3. BM niche MSC were prospectively isolated based on the following immunophenotype: TO-PRO-3^-^/Lin^-^/CD45^-^/CD235^-^/CD16^-^/CD56^-^/CD31^-^/CD271^+^ (supplementary Fig. 15A). For bulk RNA-sequencing (bulk RNA-seq) studies, MSC were sorted into 100 μl of Lysis/Binding Buffer (Ambion), vortexed, and stored at −80 °C until further processing.

### Bulk RNA sequencing

Total RNA of human BM MSC sorted from old individuals and patients was isolated with the MagMAX mirVana Total RNA Isolation Kit (Applied Biosystems, Thermo Fisher) according to the manufacturer’s protocol but finally resuspended in 15 ul of ultrapure water and examined using Takara’s SMART-Seq v4 plus kit (Cat. No. R400753, Takara Bio.) as indicated by the manufacturer’s instructions. Briefly, oligo(dt) primers are used to obtain a full-length cDNA through the SMART® (Switching Mechanism at 5′ end of RNA Template) technology. Once full-length cDNA is synthesized, libraries are prepared by the enzymatic fragmentation method, followed by library amplification and indexing sequencing libraries using unique dual indexes (Cat. R400745, Takara Bio). Libraries were quantified with Qubit dsDNA HS Assay Kit, and their profile was examined using Agilent’s HS D5000 ScreenTape Assay. Sequencing was carried out in an Illumina NextSeq2000 using paired-end dual-index sequencing (Rd1: 60 cycles; i7: 8 cycles; i5: 8 cycles; Rd2: 60 cycles) at a depth of 10–20 million reads per sample.

Raw reads were demultiplexed via bclfastq2 Conversion Software v 2.20 (Illumina) to convert base call (BCL) files into FastQ files. Quality control of FastQ files was performed using fastQC (Bioinformatics Babraham Institute), and reads were trimmed for contaminating sequence adapters and poor-quality bases using Trimmomatic^83^. Sequencing reads were aligned to hg19 human reference using STAR v 2.6^84^. Uniquely aligned reads were assigned to GENCODE18 gene annotations using HTSeq v 0.11^85^ and quantified with htseq-counts run in stranded mode with default parameters.

### Bulk RNA sequencing analysis

Before proceeding with the analysis, we extensively evaluated the MSC proportion in our samples. First, we used the CIBERSORTx deconvolution platform to estimate the relative cell fractions of MSC and contaminating cells present in our previous work with human scRNA-seq experiments (Supplementary Fig. 17A). The signature matrix was prepared using pseudobulks of EC, MSC, neutrophils, megakaryocytes, T cells, NK, dendritic cells, PC, and B cells. Gene counts were normalized for MSC (excluding the pseudobulk for other populations) using the voom-limma R package after removing genes with low mean expression (less than 10 counts). The signature matrix from our monoclonal gammopathy samples was uploaded to the CIBERSORTx website following its instructions. Permutations were set to 500, and the rest of the parameters retained the default. After running CIBERSORTx, we obtained the relative proportions of MSC (including pericytes) and the contaminating populations in each sample. Next, gene signature enrichment analysis using the AUCell^16^ method was applied to quantify each sample´s mesenchymal signatures. First, we used the Gene Set function from the GSEAbase R package to create a gene list of 20 markers that define the MSC. After processing the gene sets, the genes were ranked for each sample from the ones with the highest expression to those with the lowest using AUCell´s AUC_buildRanking function.

After determining the proportions of MSC in our samples and confirming the identity by the expression of MSC canonical markers (Supplementary Fig. 17B), we proceeded with the subsequent analysis. We filtered those genes with an average expression per condition lower than 2 CPM (counts per million). Next, the batch effect was corrected using the sva package (v 3.46)^86^. These gene count estimates were normalized, prefiltered, and used for determining the differential expression of genes between the different groups through the DESeq2 package^87^. In this differential expression analysis, the proportions of MSC estimated by CIBERSORTx were included in the model as a covariate to avoid their possible impact on the results. The Benjamini-Hochberg False discovery rate was applied to adjust for multiple hypothesis testing, and genes with an adjusted *p*-value below 0.05 were considered differentially expressed. To evaluate the contribution of contaminating cells to differential gene expression, we calculated the Spearman correlation between the expression of previously identified DEGs and the niche proportions quantified with CIBERSORTx and the cell-type signature score calculated with AUC. Genes whose expression was significantly correlated with any of those two variables with an adjusted *p*-value below 0.05 were excluded from the results. Next, to address with the high variability observed among the samples, we performed a Wilcoxon Test, where we selected those genes with an adjusted *p*-value below 0.05. This set of genes was the one used for the downstream analysis.

ORA using the functions enrichGO and compareCluster in the package clusterProfiler (v 4.6.2)^74^ were conducted to interpret the enrichment and pathways of the filtered DEGs. Gene sets with an adjusted *p*-value below 0.05 were considered statistically significant. Additionally, GSEA was calculated using the Hallmark gene sets from the Molecular Signatures Database v.7.5.1 in the list of DEGs ranked according to log2FC. In addition, the AUCell method was applied to quantify the IFN signature discovered in the mouse data.

The transcription factor activity was estimated by Decoupler^88^. It compiles information from CollecTRI^89^ database via Omnipath package^77^, and applies a Univariate Linear Model (ulm) to normalized log-transformed RNA counts so it can predict the observed gene expression based on the TF’s TF-Gene interaction weights.

### Immunofluorescence staining of human BM biopsies

The bone chips from HD were processed for IF staining according to the previously described protocol for mouse samples. Subsequently, FFPE samples from HD and archived human BM biopsies were blocked with 5% BSA for 30 min at RT before incubation with the following primary antibodies: mouse anti-CD271 monoclonal antibody (NGF Receptor) (ME20.4) (Invitrogen™ 1: 50 dilution), rabbit GBP2 Polyclonal Antibody (Proteintech, 1: 100 dilution), and rabbit ISG15 Polyclonal Antibody (Proteintech, 1: 100 dilution).

The slides were subsequently incubated with the corresponding secondary antibodies in a 1:200 dilution: Goat anti-Rabbit IgG (H + L) Highly Cross-Adsorbed Secondary Antibody, Alexa Fluor™ 488 (Invitrogen), Goat anti-Rabbit IgG (H + L) Highly Cross-Adsorbed Secondary Antibody Alexa Fluor™ 647 (Invitrogen), goat anti-Mouse IgG (H + L) Cross-Adsorbed Secondary Antibody, and Alexa Fluor™ 568 (Invitrogen). Nuclei were stained with DAPI (Vectashield 1:10 dilution). Fluorescence images were acquired using the Vectra Polaris Multispectral Imaging System (Perkin Elmer).

### Immunofluorescence analysis of human BM biopsies

This analysis assessed mesenchymal IFN-enriched cells by evaluating the coexpression of MSC marker and IFN-responsive genes. The percentage of MSC was quantified based on CD271expression, while IFN-enriched cells were identified through staining for IFN-responsive genes, including GBP2 and ISG15. Image analysis and quantification were conducted using QuPath (version 0.5.1), providing a detailed assessment of IFN-driven mesenchymal alterations.

MM samples were stratified into IFN-high and IFN-low groups using the 80th percentile as the discriminatory threshold.

### Statistics & reproducibility

No statistical method was used to predetermine sample size. Sample sizes were based on the availability of the corresponding mouse models and human samples, as well as consistency with previous studies of related design. No data were excluded from the analyses unless they failed predefined quality-control criteria, including low library complexity, high mitochondrial RNA content, predicted doublets, or lack of canonical marker expression after cell subsetting, as described above. The experiments were not randomized. The investigators were not blinded to allocation during experiments and outcome assessment.

For single-cell RNA-seq experiments, BM cells from 4–6 mice per group were pooled before library preparation, and analyses were therefore restricted to stage-associated transcriptional signatures rather than statistical inference at the level of individual animals. For bulk RNA-seq and imaging-based analyses, sample sizes for each experiment are indicated in the corresponding Results, figure legends, or Methods subsections. Statistical analyses were performed using R (v4.1.3) and GraphPad Prism (v8.0.2). For single-cell RNA-seq differential expression analyses, two-sided MAST tests were used as implemented in Seurat, followed by Bonferroni correction for multiple testing. For bulk RNA-seq differential expression analyses, two-sided Wald tests implemented in DESeq2 were used, followed by Benjamini–Hochberg false-discovery rate correction. Enrichment analyses were corrected for multiple testing using the Benjamini–Hochberg method. Two-sided Wilcoxon rank-sum tests were used for comparisons of signature scores and gene expression distributions where indicated. Two-sided Spearman correlation tests were used for correlation analyses. Quantitative IF analyses were assessed using unpaired two-tailed Student’s t-tests. Exact statistical tests used for each experiment are reported in the corresponding figure legends.

Representative immunofluorescence images shown in the figures were obtained from independent biological samples processed using the same staining and imaging workflows. Quantifications were performed using the full set of samples indicated for each experiment.

### Reporting summary

Further information on research design is available in the Nature Portfolio Reporting Summary linked to this article.

## Supplementary information

Supplementary Information

Description of Additional Supplementary Files

Supplementary data

Reporting Summary

Transparent Peer Review file

## Source data

Source data

## Data Availability

The murine BM single-cell RNA-seq data generated in this study have been deposited in the NCBI Gene Expression Omnibus (GEO) database under accession GSE305390. The human BM MSC bulk RNA-seq data generated in this study have been deposited in the GEO database under accession GSE305389. These datasets are linked under BioProject PRJNA1303025. The public scRNA-seq datasets of the non-hematopoietic BME of newly diagnosed individuals with MM and individuals without MM used in this study are available in the ArrayExpress database under accession code E-MTAB-9139, and were used to validate the enrichment of the IFN signature in a subset of MM patients^12^. The bulk RNA-sequencing datasets of MSC from individuals with MM at diagnosis and after induction treatment used in this study are available in the ArrayExpress database under accession code E-MTAB-9285, and were used to confirm suppression of the IFN signature following therapy^12^. Source data are provided with this paper.

## Code availability

The code used for single-cell RNA-seq and bulk RNA-seq analyses in this study is publicly available at https://github.com/icenzano/NicheMGUSMM_2025 and permanently archived at Zenodo (10.5281/zenodo.18631631). The archived release (v1.0.0) corresponds to the version used for the analyses reported in this manuscript.
