# Supplementary material for: Stromal and endothelial transcriptional changes during progression from MGUS to myeloma and after treatment response
Source: Nat Commun. 2026 Jun 23;17:7893. doi: 10.1038/s41467-026-74389-y (PMC13443197; doi:10.1038/s41467-026-74389-y)
Supplement: Supplementary file 1 [file 41467_2026_74389_MOESM1_ESM.pdf]

SUPPLEMENTARY FIGURES

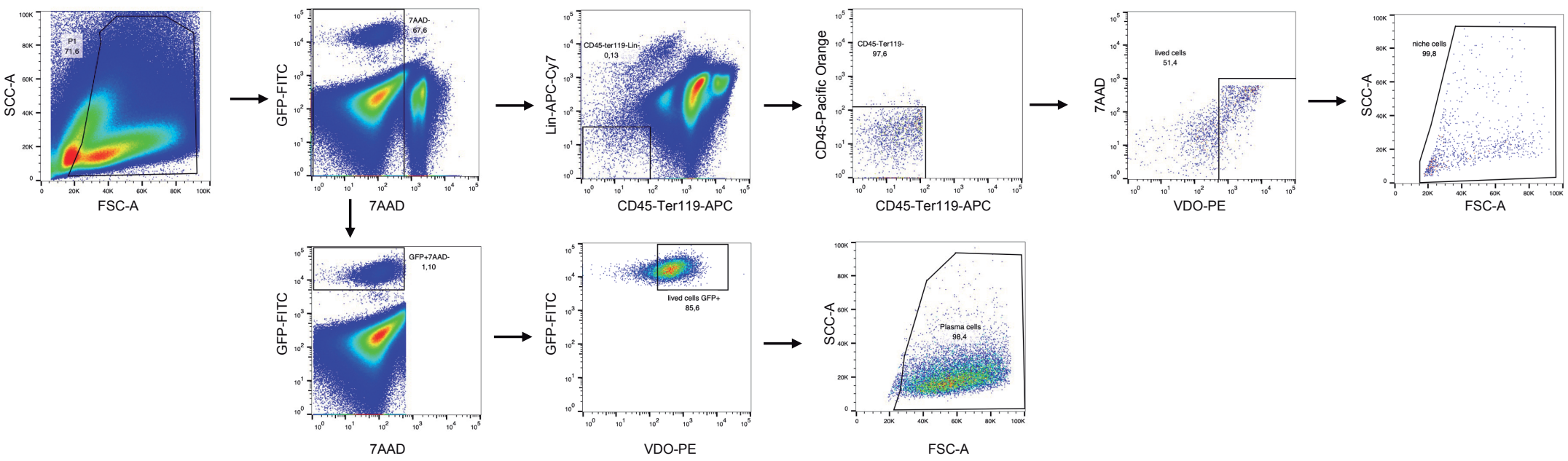

Supplementary Figure 1: Cell sorting strategy for murine scRNA-seq of BM cells. Cell sorting strategy for the isolation of mouse BME cells (Lived non-hematopoietic BM cells: 7-AAD$^-$ Lin$^-$ CD45$^-$ Ter119$^-$ VDO$^+$) and PC (Lived PC: 7-AAD$^-$ VDO$^+$ GFP$^+$).

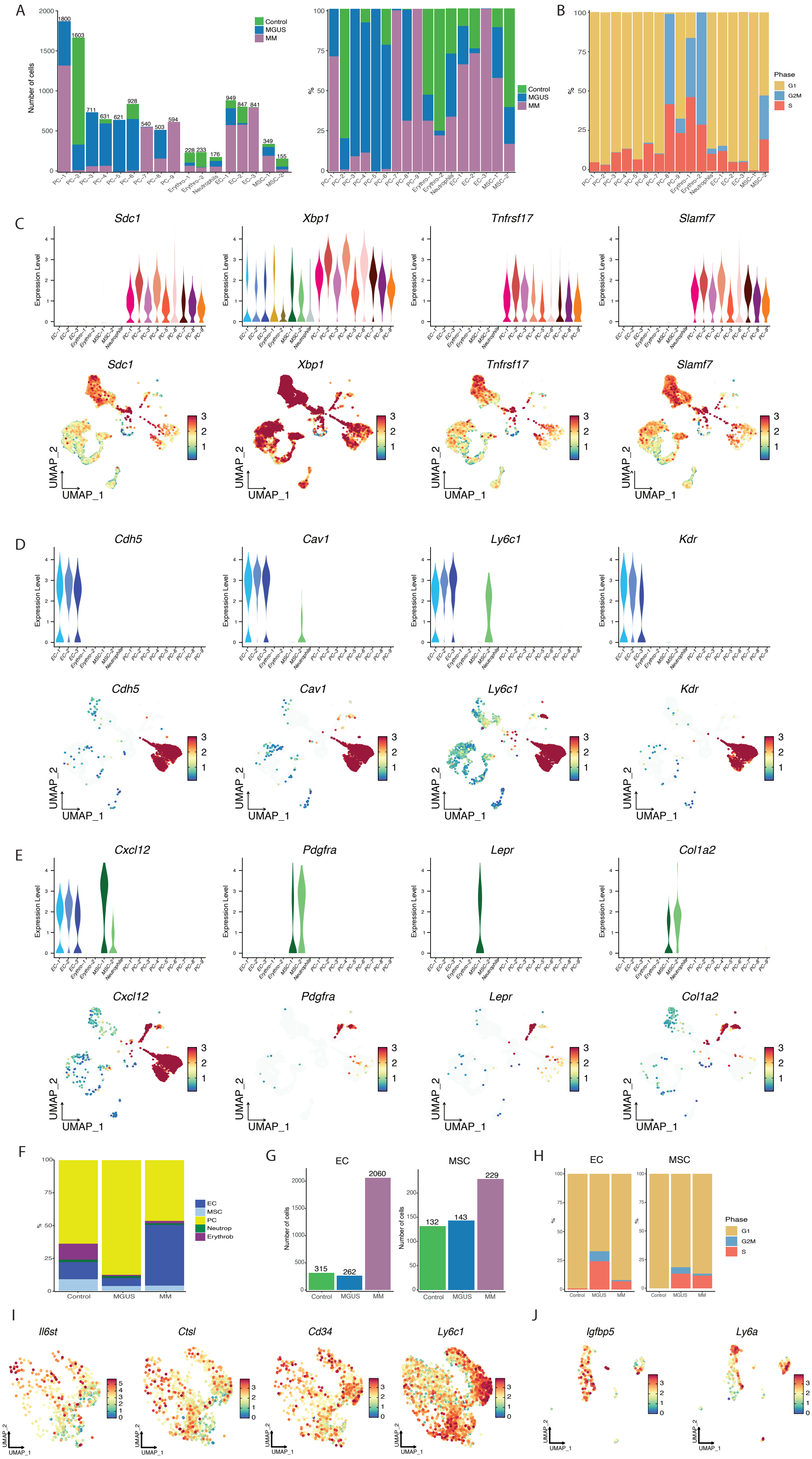
A
Number of cells
Control
MGUS
MM
1800
1603
711
631
621
928
540
503
594
228
233
176
949
847
841
349
155
%
B
Phase
G1
G2M
S
C
Sdc1
Xbp1
Tnfrsf17
Slamf7
Expression Level
UMAP_1
UMAP_2
D
Cdh5
Cav1
Ly6c1
Kdr
E
Cxcl12
Pdgfra
Lepr
Col1a2
F
EC
MSC
PC
Neutrop
Erythrob
G
EC
MSC
315
262
2060
132
143
229
H
I
Il6st
Ctsl
Cd34
Ly6c1
J
Igfbp5
Ly6a

6

Supplementary Figure 2: scRNA-seq analysis of murine BM cells. (A) Left panel: bar plots showing the number of cells per cluster and dataset; Right panel: stacked bar plot representing the proportion of cells per cluster and dataset. (B) Stacked bar plot of the proportion of cells per cycle stage in each cluster. (C) Upper panel: Violin plot of gene expression of well-known markers for PC. Bottom panel: UMAP visualization of representative canonical markers for PC. (D-E) Similar to C for EC (D) and MSC (E). (F) Stacked bar plot of the proportion of BM cell populations per disease dataset. (G) Bar plot depicting the total number of cells per stage for EC (left) and MSC (right) after final filtering (see methods). (H) Stacked bar plot of the proportion of cells per cycle stage in each stage for EC (left) and MSC (right). (I) UMAP visualization of markers for sinusoids (*Il6st, Ctsl*) and arteries (*Cd34,Ly6c1*) in EC. (J) UMAP visualization of MSC canonical markers in MSC.

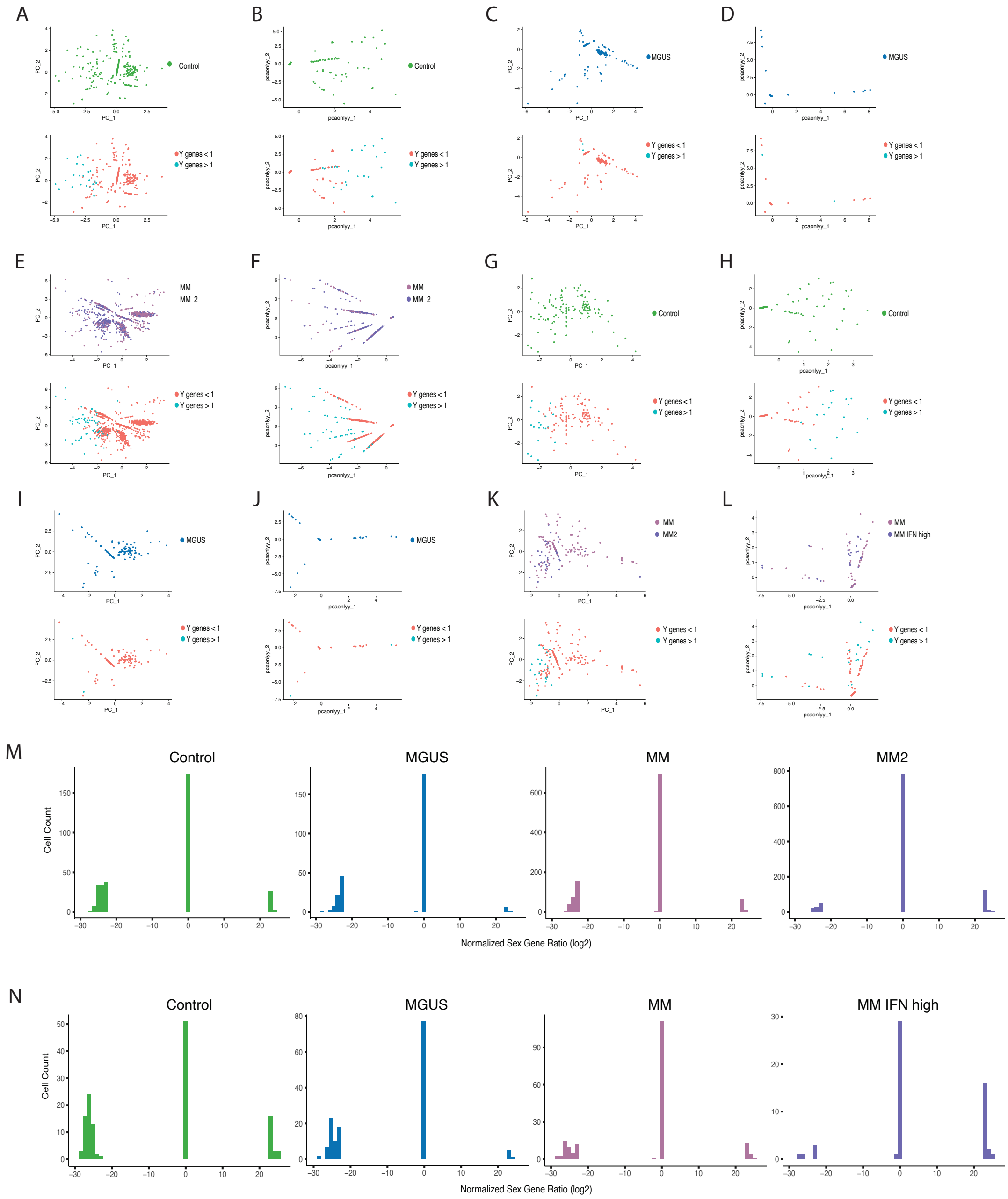

Supplementary Figure 3: Analysis of mouse contribution to control, MGUS, and MM $BI_{c\gamma1}$ EC and MSC using sex-specific genes. (A) PCA plot showing separation of control EC based only on the expression of sex-specific genes colored by disease (upper panel) and Y-chromosome gene expression, where blue denotes cells with more than one count in Y-chromosome genes (bottom panel). (B) PCA plot showing separation of control EC based only on the expression of Y-chromosome genes colored by disease (upper panel) and Y-chromosome gene expression, where blue denotes cells with more than one count in Y-chromosome genes (bottom panel). (C,D) Similar to A,B for MGUS EC. (E,F) Similar to A,B for MM EC. MM EC cells were split into

MM and MM2 clusters. (G,H) Similar to A,B for Control MSC. (I,J) Similar to A,B for MGUS MSC. (K,L) Similar to A,B for MM MSC. MM MSC cells were split into MM IFN low and high score. (M) Histogram of number of EC plotted by male to female gene ratios (scored continuously along the x-axis) in control, MGUS, and MM $BI_{c\gamma1}$ EC. MM EC cells were split into MM and MM2 clusters. Three discrete peaks emerge, representing cells likely originating from either sex or those with indeterminate ratios due to technical drop-off in scRNA-seq reads. (N) Similar to M for MSC.

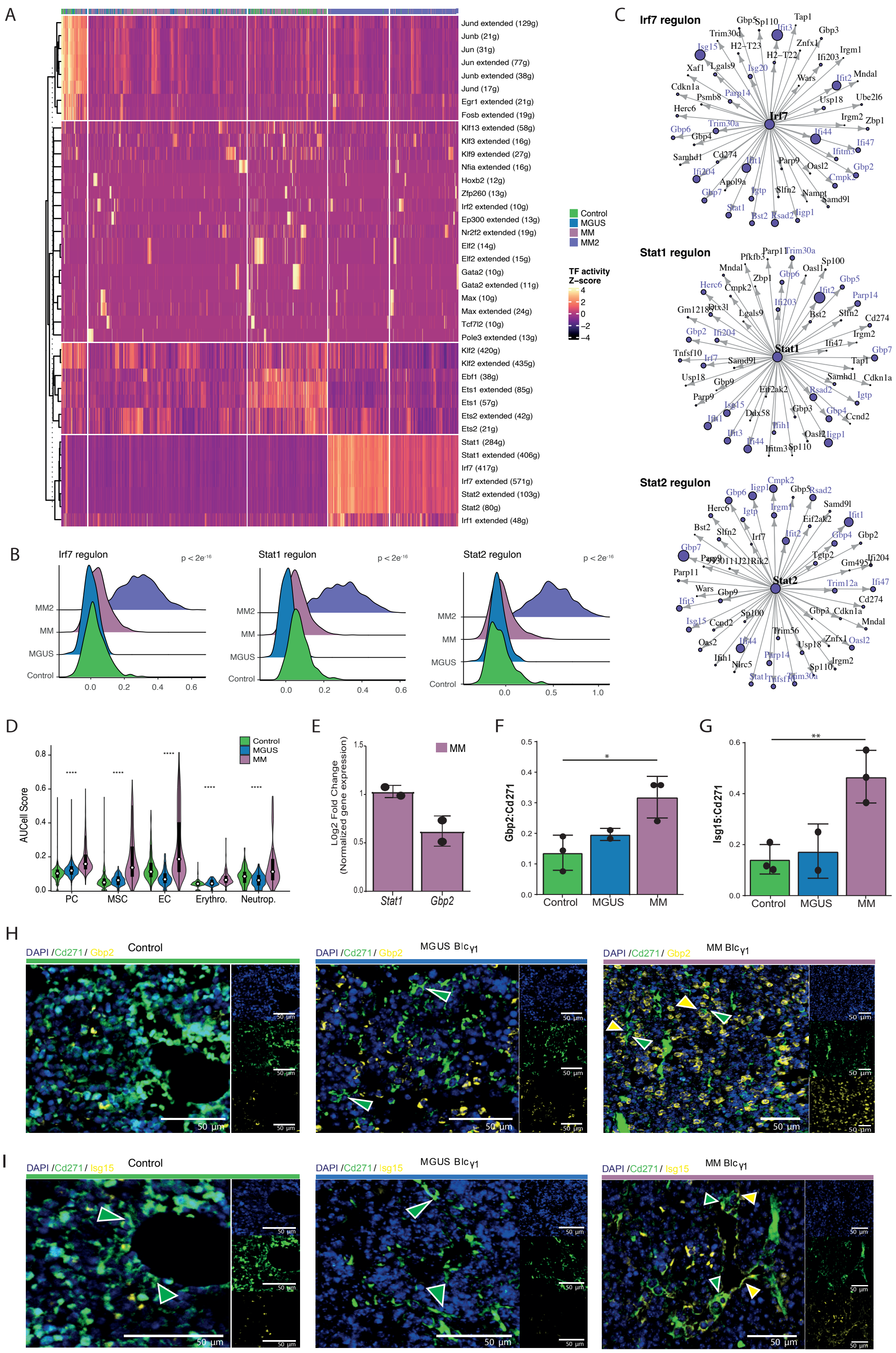
A
Jund extended (129g)
Junb (21g)
Jun (31g)
Jun extended (77g)
Junb extended (38g)
Jund (17g)
Egr1 extended (21g)
Fosb extended (19g)
Klf13 extended (58g)
Klf3 extended (16g)
Klf9 extended (27g)
Nfia extended (16g)
Hoxb2 (12g)
Zfp260 (13g)
Irf2 extended (10g)
Ep300 extended (13g)
Nr2f2 extended (19g)
Elf2 (14g)
Elf2 extended (15g)
Gata2 (10g)
Gata2 extended (11g)
Max (10g)
Max extended (24g)
Tcf7l2 (10g)
Pole3 extended (13g)
Klf2 (420g)
Klf2 extended (435g)
Ebf1 (38g)
Ets1 extended (85g)
Ets1 (57g)
Ets2 extended (42g)
Ets2 (21g)
Stat1 (284g)
Stat1 extended (406g)
Irf7 (417g)
Irf7 extended (571g)
Stat2 extended (103g)
Stat2 (80g)
Irf1 extended (48g)
Control
MGUS
MM
MM2
TF activity Z-score
4
2
0
−2
−4
C
Irf7 regulon
Irf7
Stat1 regulon
Stat1
Stat2 regulon
Stat2
B
Irf7 regulon
Stat1 regulon
Stat2 regulon
MM2
MM
MGUS
Control
D
AUCell Score
PC
MSC
EC
Erythro.
Neutrop.
E
Log2 Fold Change (Normalized gene expression)
MM
Stat1
Gbp2
F
Gbp2:Cd271
Control
MGUS
MM
G
Isg15:Cd271
Control
MGUS
MM
H
DAPI /Cd271 / Gbp2
Control
MGUS Blcγ1
MM Blcγ1
50 µm
I
DAPI /Cd271 / Isg15
Control
MGUS Blcγ1
MM Blcγ1
50 µm

Supplementary Figure 4: Additional information on the MM IFN-related signature in the $\mathbf{BI_{c\gamma1}}$ model. (A) Heatmap showing the activities of the enriched regulons in EC (Z-score of regulon activity). (B) Density plots showing the score of Irf7, Stat1, and Stat2 regulons in EC (p. = adjusted p-value). (C) Regulon network of Irf7, Stat1, and Stat2 representing the top 50 target genes. (D) Violin plots showing AUC-score for MM IFN-related signature within BM populations in the $BI_{c\gamma1}$ model. (E) Quantitative PCR (qPCR) analysis of the IFN regulon (*Stat1)* and the associated gene (*Gbp2)* in MSC derived from mesenspheres of MM BIcγ1 mice. Gene expression is presented as log_2 fold change relative to MSC derived from mesenspheres of control mice. Data were normalized to the reference gene *Gapdh*. n=2 independent biological replicates per group. (F-G) Quantification of the percentage of mesenchymal stromal cells IFN-enriched cells, based on the coexpression of $Cd271^+$ with IFN+ cells (Gbp2, (F); Isg15, (G)) in femurs from Control, MGUS $BI_{c\gamma1}$ and MM $BI_{c\gamma1}$ mice. n=3 independent biological replicates per control and MM group, and n=2 independent biological replicates for MGUS group. (H-I) IF staining of MSC (Cd271, green), and IFN response genes markers (Gbp2 (yellow, (H)); Isg15 (yellow, (I))), and nucleus (DAPI) (blue) in FFPE femurs from Control, MGUS $BI_{c\gamma1}$, and MM $BI_{c\gamma1}$ mice. Green arrows point to MSC ($Cd271^+$), and yellow arrows point to IFN^+ (Gbp2/Isg15). n=3 independent biological replicates per control and MM group, and n=2 independent biological replicates for MGUS group. Scale bars, 50 µm.

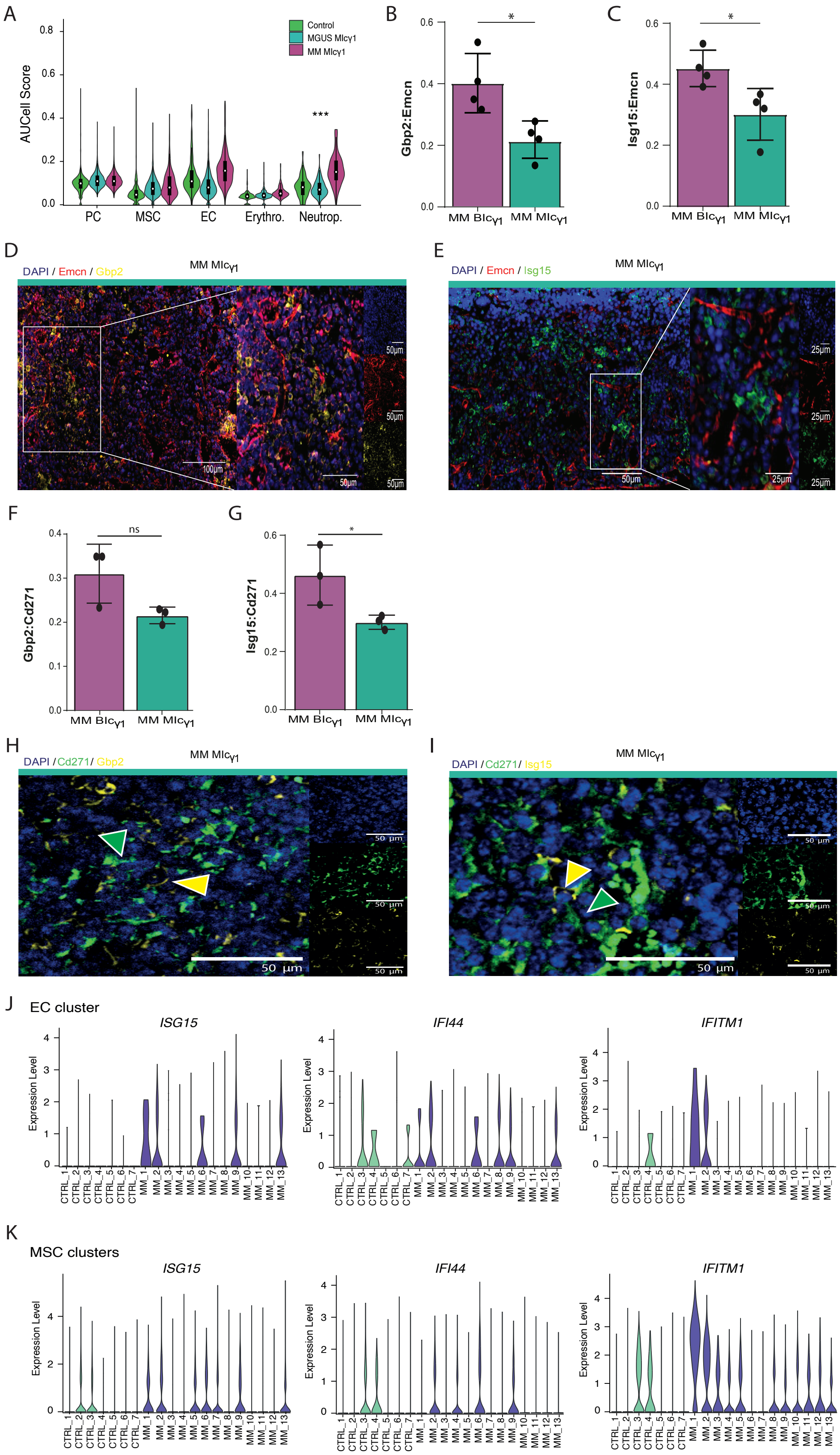
A
AUCell Score
Control
MGUS MIcγ1
MM MIcγ1
***
PC
MSC
EC
Erythro.
Neutrop.
B
Gbp2:Emcn
MM BIcγ1
MM MIcγ1
C
Isg15:Emcn
D
DAPI / Emcn / Gbp2
MM MIcγ1
E
DAPI / Emcn / Isg15
F
Gbp2:Cd271
ns
G
Isg15:Cd271
H
DAPI / Cd271 / Gbp2
I
DAPI / Cd271 / Isg15
J
EC cluster
ISG15
IFI44
IFITM1
Expression Level
K
MSC clusters

53

Supplementary Figure 5: Validation in the $MI_{c\gamma1}$ model and scRNA-seq human data.

(A) Violin plots showing AUC-score for MM IFN-related signature within BM populations in the $MI_{c\gamma1}$ model. (B-C) Quantification of the percentage of endothelial IFN-enriched cells, based on the coexpression of Emcn with IFN+ cells (Gbp2, (B); Isg15, (C)) in femurs from MM $BI_{c\gamma1}$ and MM $MI_{c\gamma1}$ mice. n = 4 mice per group. (D-E) IF staining of EC (Emcn, red), and IFN response genes markers (Gbp2 (yellow, (D)); Isg15 (green, (E))), and nucleus (DAPI) (blue) in FFPE femurs from MM $MI_{c\gamma1}$ mice. Representative images from n = 4 mice with similar results. Scale bars, 50 µm. (F-G) Quantification of the percentage of mesenchymal stromal cells IFN-enriched cells, based on the coexpression of $Cd271^{+}$ with IFN+ cells (Gbp2, (F); Isg15, (G)) in femurs from MM $BI_{c\gamma1}$ and MM $MI_{c\gamma1}$ mice. n = 3 mice per group. (H-I) IF staining of MSC (Cd271, green), and IFN response genes markers (Gbp2 (yellow, (H)); Isg15 (yellow, (I))), and nucleus (DAPI) (blue) in FFPE femurs from MM $MI_{c\gamma1}$ mice. Green arrows point to MSC ($Cd271^{+}$), and yellow arrows point to IFN^{+} (Gbp2/Isg15). Representative images from n = 3 mice with similar results. Scale bars, 50 µm. (J) Violin plots comparing the distribution of the gene expression of *ISG15*, *IFI44*, and *IFITM1* in EC from MM patients and healthy controls from scRNA-seq published data[1]. (K) Similar to J for MSC from the same healthy controls and MM human patients from scRNA-seq published data[1].

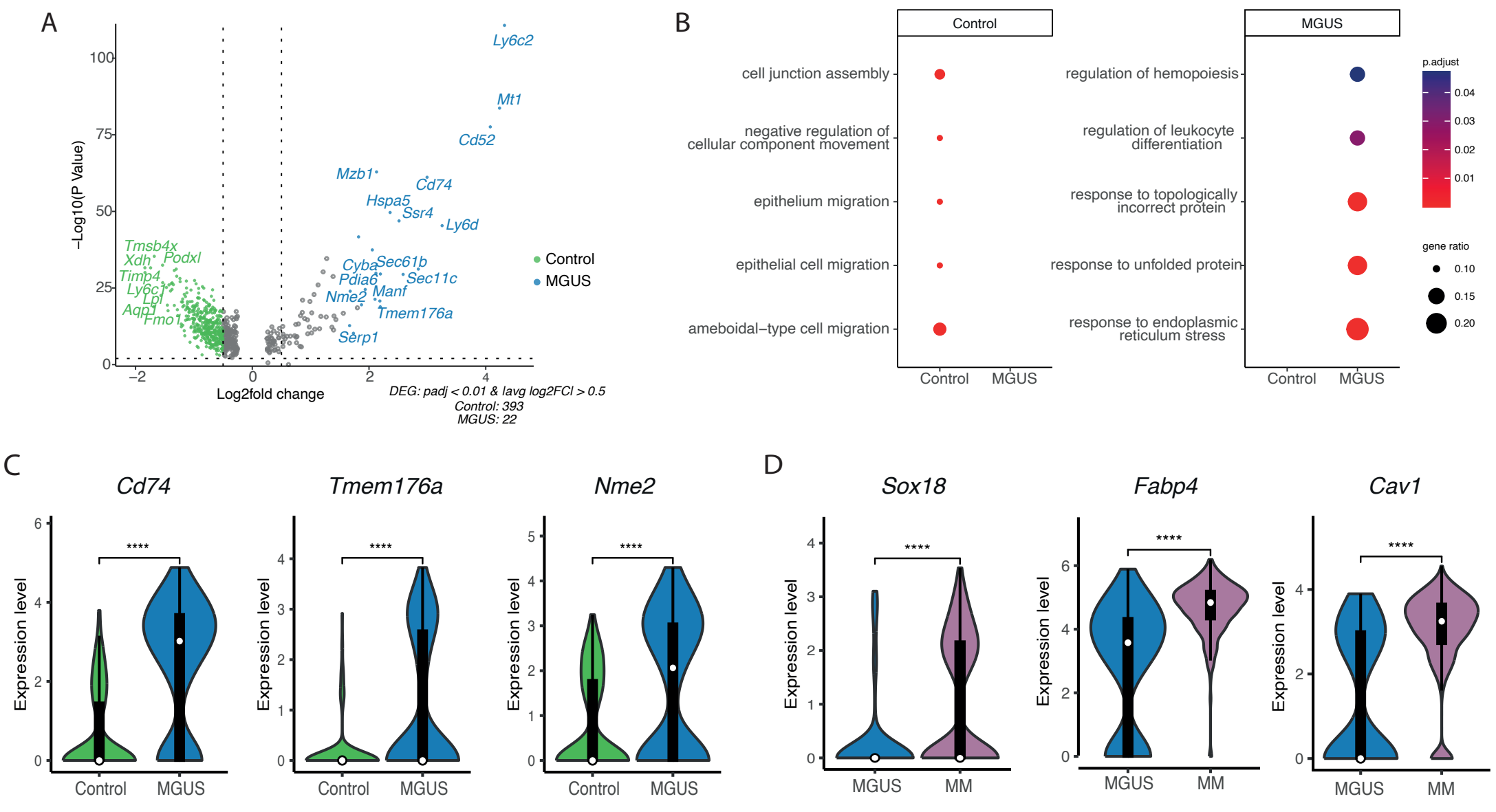

Supplementary Figure 6: Additional visualizations of transcriptional differences detected between EC from control, MGUS, and MM excluding MM IFN cells. (A) Volcano plots for the differential expression analysis between EC from control and MGUS. The y-axis represents the -$\log_{10}$(p-value), and the x-axis represents the $\log_2$FC of the gene. The dot's color denotes the disease stage for which that DEG was detected, with grey dots representing non-significant genes. (B) GO ORA analysis between EC from control and MGUS. (C) Violin plots showing the expression of relevant upregulated genes in MGUS vs control EC. (D) Violin plots showing the expression of upregulated genes in EC from MM compared to MGUS.

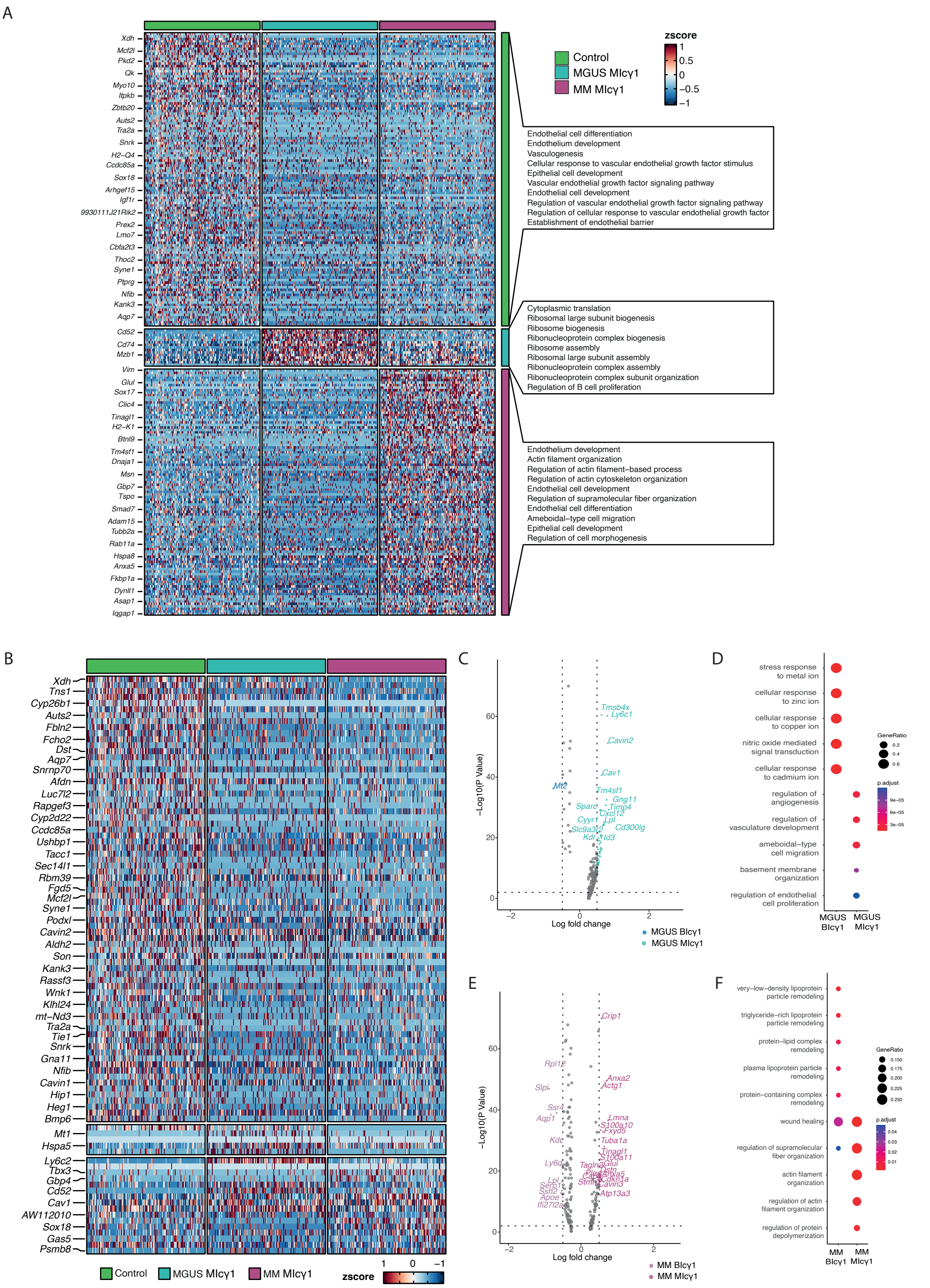

Supplementary Figure 7: Transcriptional changes in EC during myeloma progression in the MI$_{c\gamma1}$ model. (A) Heatmap of differential gene expression analysis in EC across control, MGUS, and MM. Enriched GO terms were identified for each disease stage, and the top 10 enriched terms are flagged on the right. Color represents the z-score scaled expression values. (B)

Heatmap projecting $MI_{c\gamma1}$ model gene expression of DEGs identified in EC from $BI_{c\gamma1}$ at different stages of the disease. Color represents the z-score scaled expression values of EC from the $MI_{c\gamma1}$ model. (C) Volcano plot of the DEGs between EC from MGUS in $BI_{c\gamma1}$ and $MI_{c\gamma1}$ model. The y-axis represents the -log10(p-value), and the x-axis represents the log_2FC of the gene. The dot's color denotes the model for which that DEG was detected, with grey dots representing non-significant genes. (D) GO ORA analysis between EC from $BI_{c\gamma1}$ and $MI_{c\gamma1}$ model at the MGUS stage. (E-F) Similar to (C-D) for the comparison between $BI_{c\gamma1}$ and $MI_{c\gamma1}$ model in the MM stage.

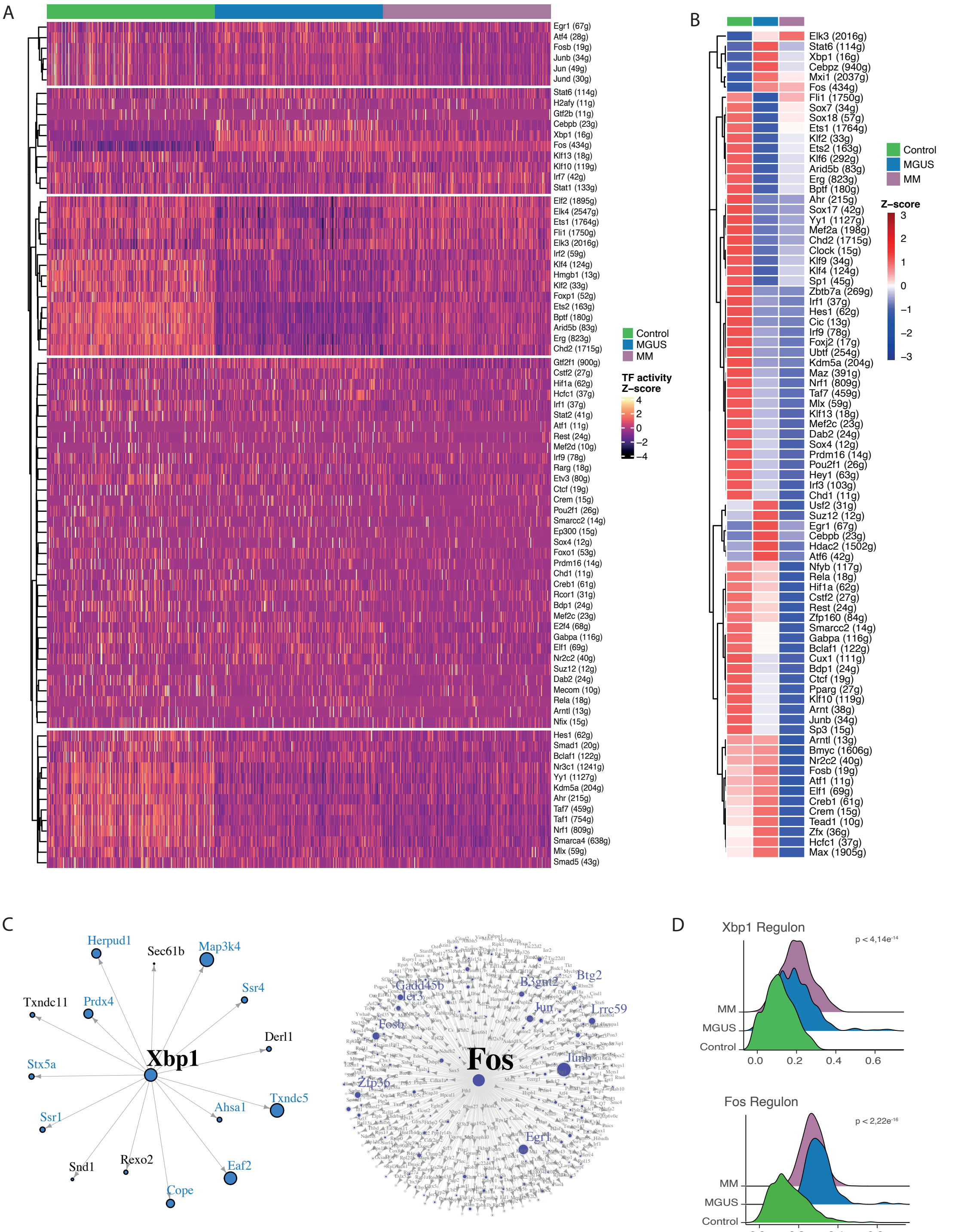

Supplementary Figure 8: Gene regulatory network of EC during MM progression excluding MM IFN signal. (A) Heatmap showing the activities of the enriched regulons in EC from control, MGUS and MM (Z-score of regulon activity). (B) Heatmap of mean regulon activity score of significantly enriched regulons in each stage. The color scale represents the z-score of the activity scaled by row. (C) Regulon network of Xbp1 and Fos representing the top target genes. (D) Density plots showing the score of Xbp1 and Fos regulons in EC.

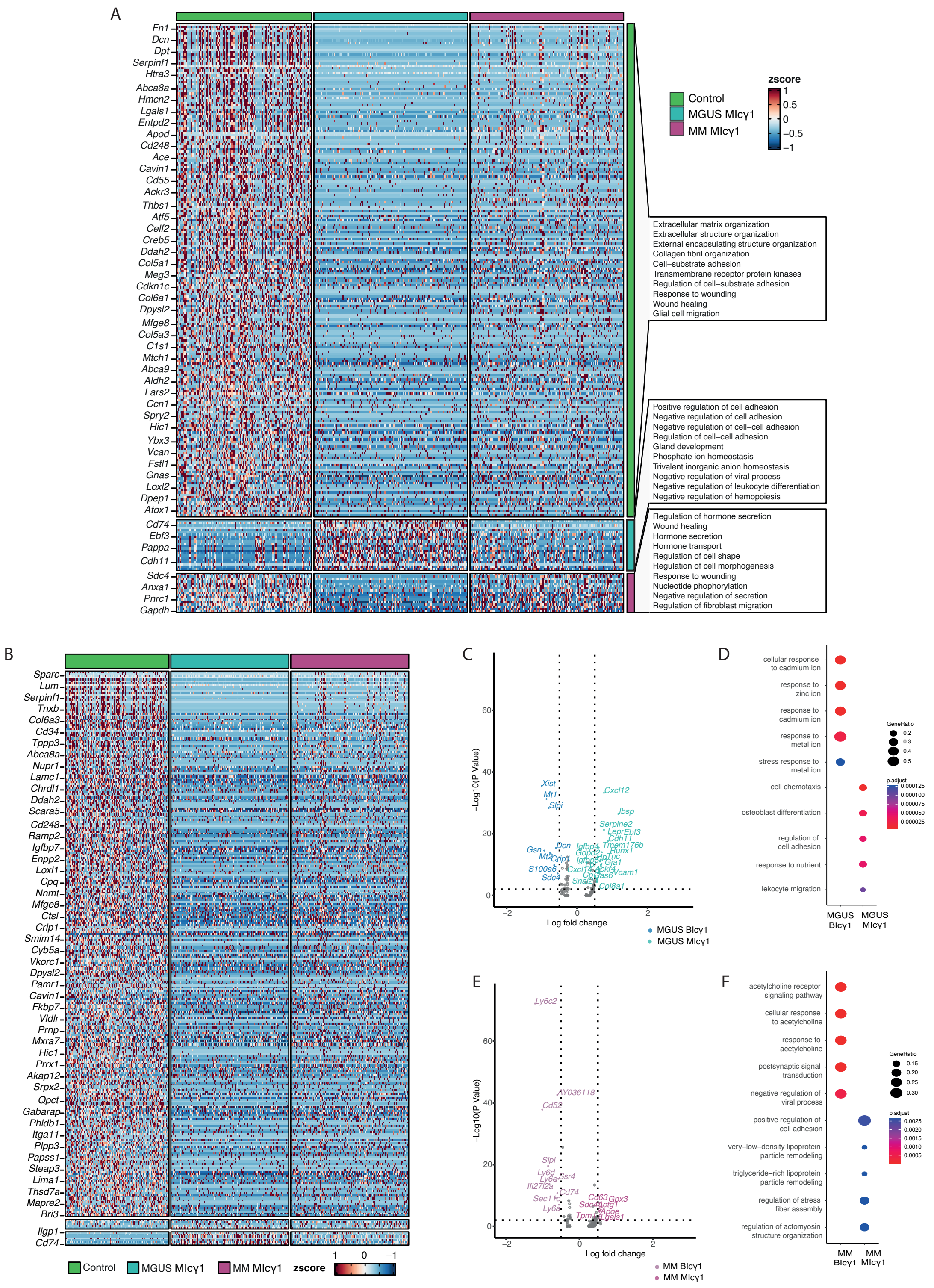

Supplementary Figure 9: Transcriptional remodeling of MSC during myeloma development in the MI$_{c\gamma1}$ model. (A) Heatmap of differential gene expression analysis in MSC across control, MGUS, and MM stratified by stage. Enriched GO terms were identified for each disease stage, and the top 10 enriched terms are flagged on the right. Color represents the z-score scaled expression values. (B) Heatmap projecting MI$_{c\gamma1}$ model gene expression of DEG identified

from $BI_{c\gamma1}$ model in control, MGUS and MM. Color represents the z-score scaled expression values of MSC from the $MI_{c\gamma1}$ model. (C) Volcano plot of the DEGs between MSC in MGUS from $BI_{c\gamma1}$ and $MI_{c\gamma1}$ model. The y-axis represents the -log10(p-value), and the x-axis represents the log_2FC of the gene. The dot's color denotes the model from which that DEG was detected, with grey dots representing non-significant genes. (D) GO ORA between MSC from $BI_{c\gamma1}$ and $MI_{c\gamma1}$ model at the MGUS stage. (E-F) Similar to (C-D) for the comparison between MSC from $BI_{c\gamma1}$ and $MI_{c\gamma1}$ model in the MM stage.

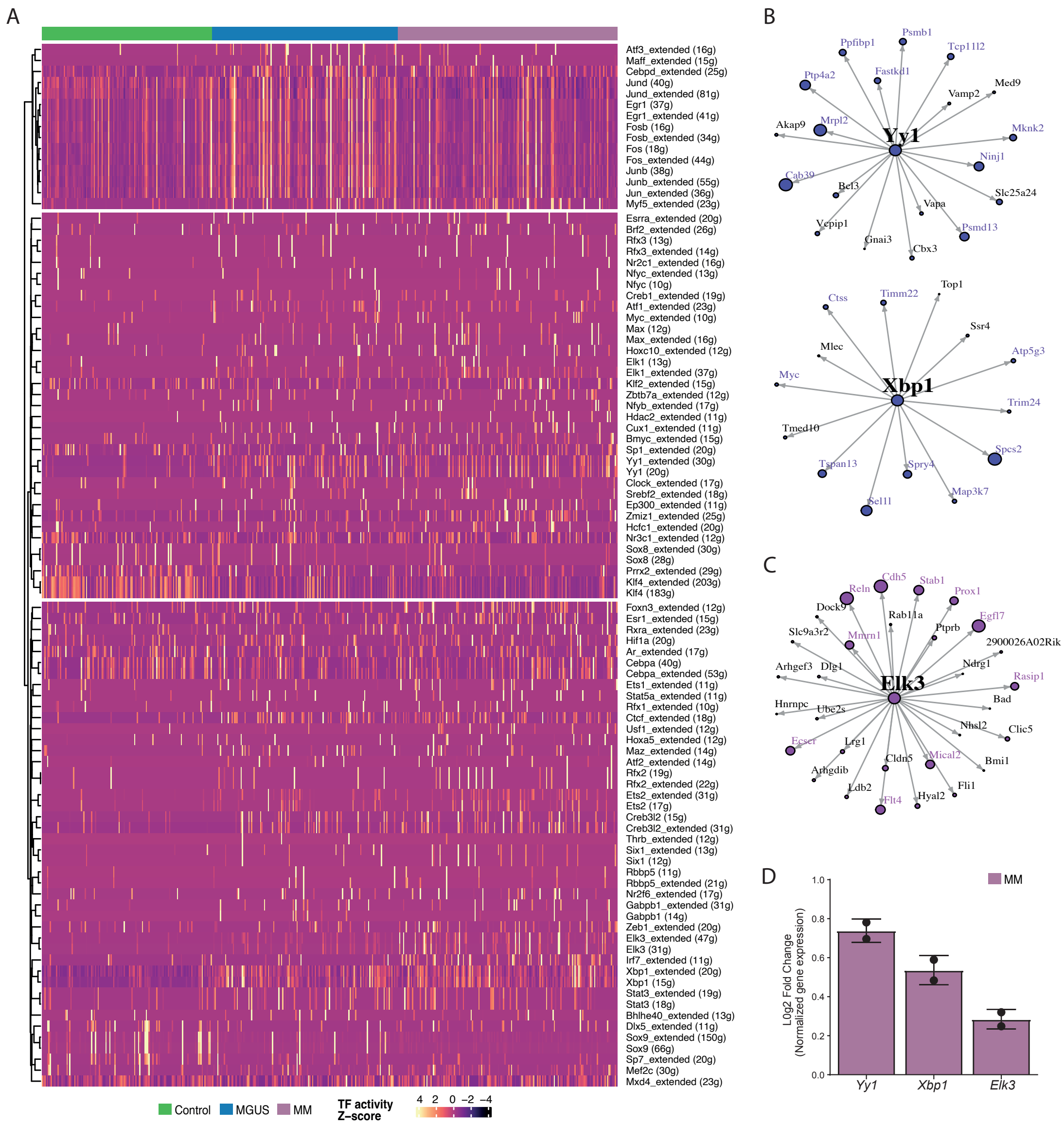

Supplementary Figure 10: Gene regulatory network of MSC during MM progression excluding MM IFN signal. (A) Heatmap showing the activities of the enriched regulons in MSC from control, MGUS, and MM (Z-score of regulon activity). (B) Regulon network of Yy1 and Xbp1, representing the top associated genes (C) Regulon network of Elk3 representing the top associated genes. (D) Quantitative PCR (qPCR) analysis of the enriched regulons (*Yy1*, *Xbp1*, and *Elk3*) in MSC derived from mesenspheres of MM BIcγ1 mice. Gene expression is presented as $\log_2$ fold change relative to MSC derived from mesenspheres of control mice. Data were

normalized to the reference gene *Gapdh*. qPCR data are mean ± s.e.m.; n = 2 independent biological replicates per group.

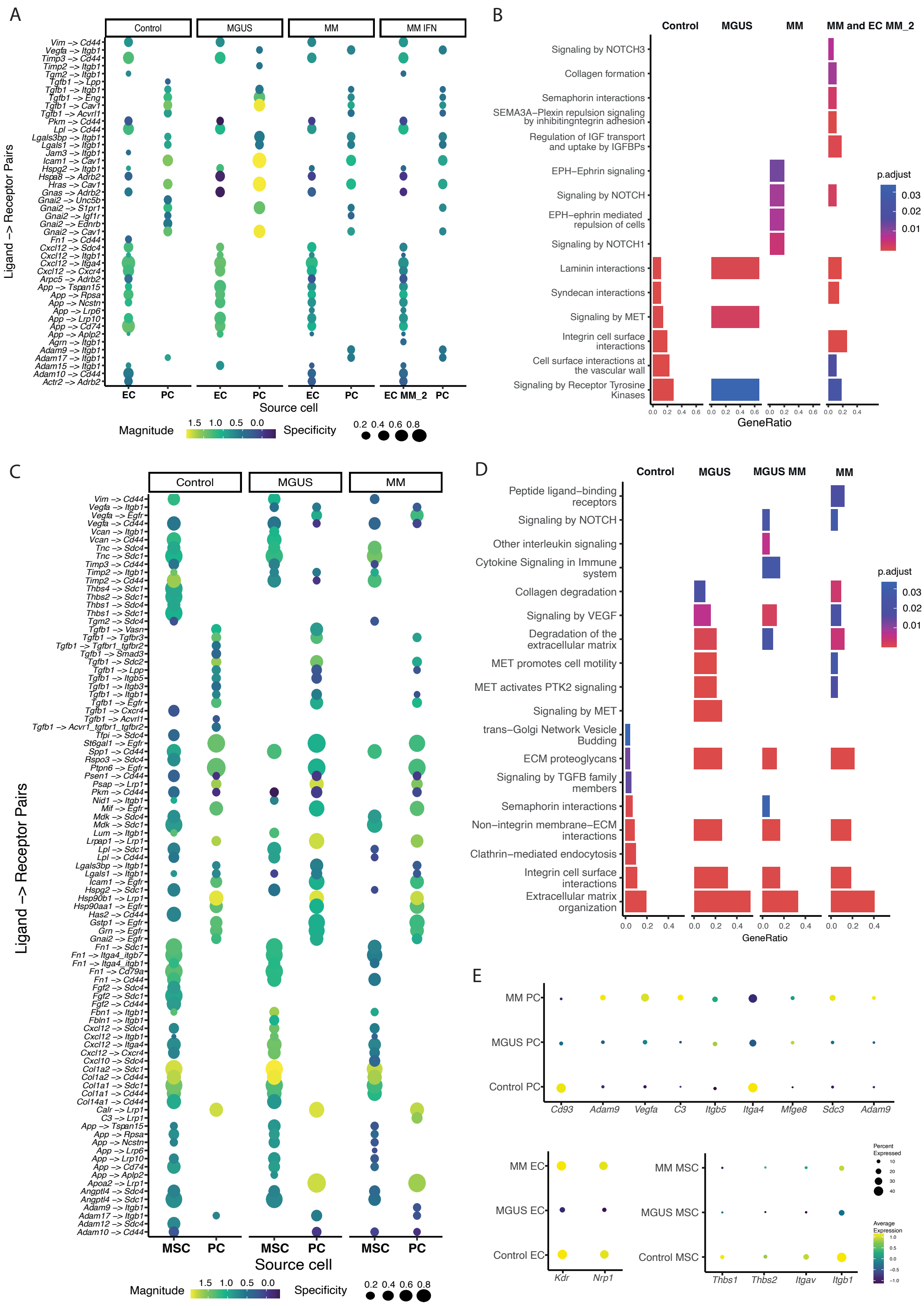

Supplementary Figure 11: Cell-cell communication analysis between EC, MSC and PC. (A)

Dot plots of ligand-receptor interactions between PC and EC that are maintained along the disease stages. Dot size indicates specificity score; color indicates interaction strength. (B) Representative Reactome pathways enriched in EC-PC L-R pairs specific for each stage. (C-D) Similar to (A-B) for MSC-PC communication. (E) Dot plot showing the expression of relevant ligands and receptors in PC, EC and MSC across disease stages. The dot size reflects the percentage of cells within the cluster expressing each gene, and the color represents the average expression level.

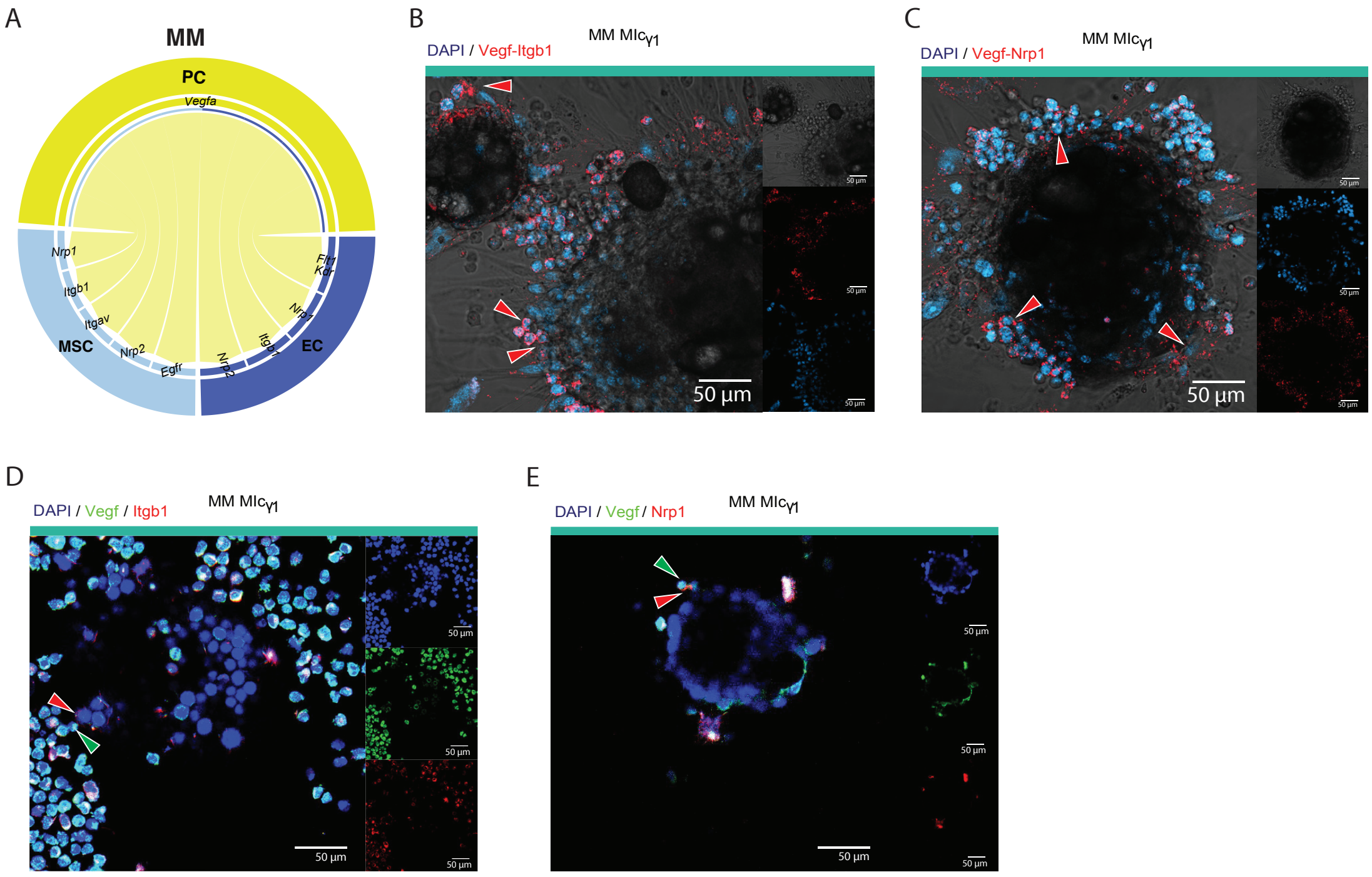

Supplementary Figure 12. MM-specific interactions among EC, MSC, and PC in MM BIcγ1 mice and functional validation assays. (A) Chord diagrams illustrating L-R pairs shared between PC–EC and PC–MSC communication within the MM stage from the MM MIcγ1 mouse model. Colors and width represent signal senders and strength, respectively. PC-derived *Vegfa* engages multiple receptors in both cell types. (B-C) Proximity ligation assay (Navinci Kit) analysis of MM MIcγ1 interactions (Vegf-Itgb1 (B) and Vegf-Nrp1 (C)) in coculture of PC (cell line MM5080) with MSC derived from mesenspheres of MM MIcγ1 mice. Representative confocal images show PLA-positive puncta (red arrows), indicating protein–protein interactions, with nuclei counterstained with DAPI (blue). (D-E) IF staining of PC ligands (Vegf (green)), with MSC-receptors (Itgb1 (red (D)) and Nrp1 (red (E)), and nucleus (DAPI) (blue) in coculture of PC (cell line MM5080) with MSC derived from mesenspheres of MM MIcγ1 mice. Green arrows point to PC-Vegf^{+}, and red arrows point to MSC-Itgb1 (D) or Nrp1 (E). (B-E) Representative images from n = 2 independent coculture experiments with similar results. Scale bars, 50 µm.

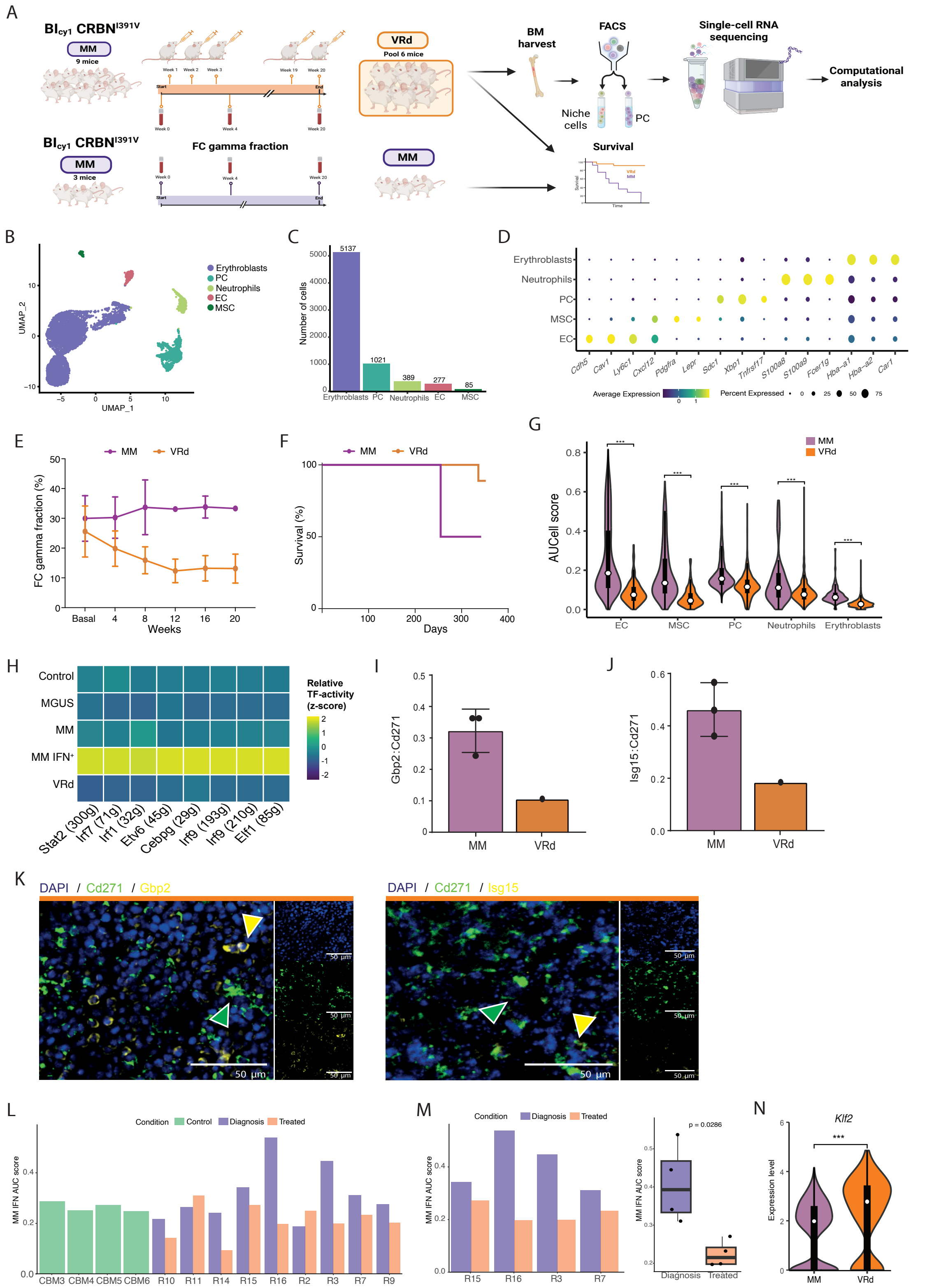

Supplementary Figure 13: Additional analysis of VRd-treated mice and transcriptional alterations in EC and MSC from human patients. (A) VRd experiment overview representing

treatment administration, serum collection for FC gamma fraction, and scRNA-seq isolation of PC, MSC, and EC from MM BI$_{c\gamma1}$ *Crbn*^{I391V} (pool of six mice). MM BI$_{c\gamma1}$ *Crbn*^{I391V} were used as controls for FC gamma fraction and survival. Created with BioRender.com. (B) UMAP representation of BM microenvironment cells from BI$_{c\gamma1}$-*Crbn*^{I391V} VRd-treated mice, colored by cell clustering. (C) Bar plots showing the number of cells per BM population. (D) Dot plot of canonical markers used to define PC, EC, MSC, erythroblasts, and neutrophils. The dot size reflects the percentage of cells within the cluster expressing each gene, and the color represents the average expression level. (E) Representation of the percentage of serum FC gamma fraction in VRd-treated versus the MM group. (F) Kaplan–Meier survival curves of treated and non-treated MM BI$_{c\gamma1}$ mice. (G) Violin plots showing AUC-score for MM IFN-related signature within BM populations in VRd-treated and MM BI$_{c\gamma1}$ mice. (H) Heatmap representing z-scaled mean regulon activity score of IFN-related regulons in EC across each condition. (I-J) Quantification of the percentage of mesenchymal IFN$^+$ cells, determined by the coexpression of Cd271 with IFN response genes (Gbp2 (I) and Isg15 (J) in femurs from n=3 MM BIc$_{\gamma1}$ (MM) and n=1 MM BI$_{c\gamma1}$-*Crbn*^{I391V} VRd-treated (VRd) mice. (K) IF staining of MSC (Cd271, green), IFN response (Gbp2, yellow (left plot) and Isg15, yellow (right plot)), and nucleus (DAPI) (blue) in FFPE femurs from VRd-treated mice. Green arrows point to MSC (Cd271$^+$), and yellow arrows point to IFN$^+$ (Gbp2/Isg15). n = 1 mice per group. Scale bars, 50 µm. (L) Bar plots showing the AUC-score for MM IFN-related signature in MSC from healthy controls and MM human patients before and post-induction treatment from bulk RNA-seq published data[1]. (M) Bar plots showing the AUC-score for MM IFN-related in MSC from the subset of high IFN MM human patients before and after induction treatment[1] by individuals (left panel) and grouped by disease (right panel). (N) Violin plot displaying the upregulation of *Klf2* in VRd-treated EC vs non-treated MM EC.

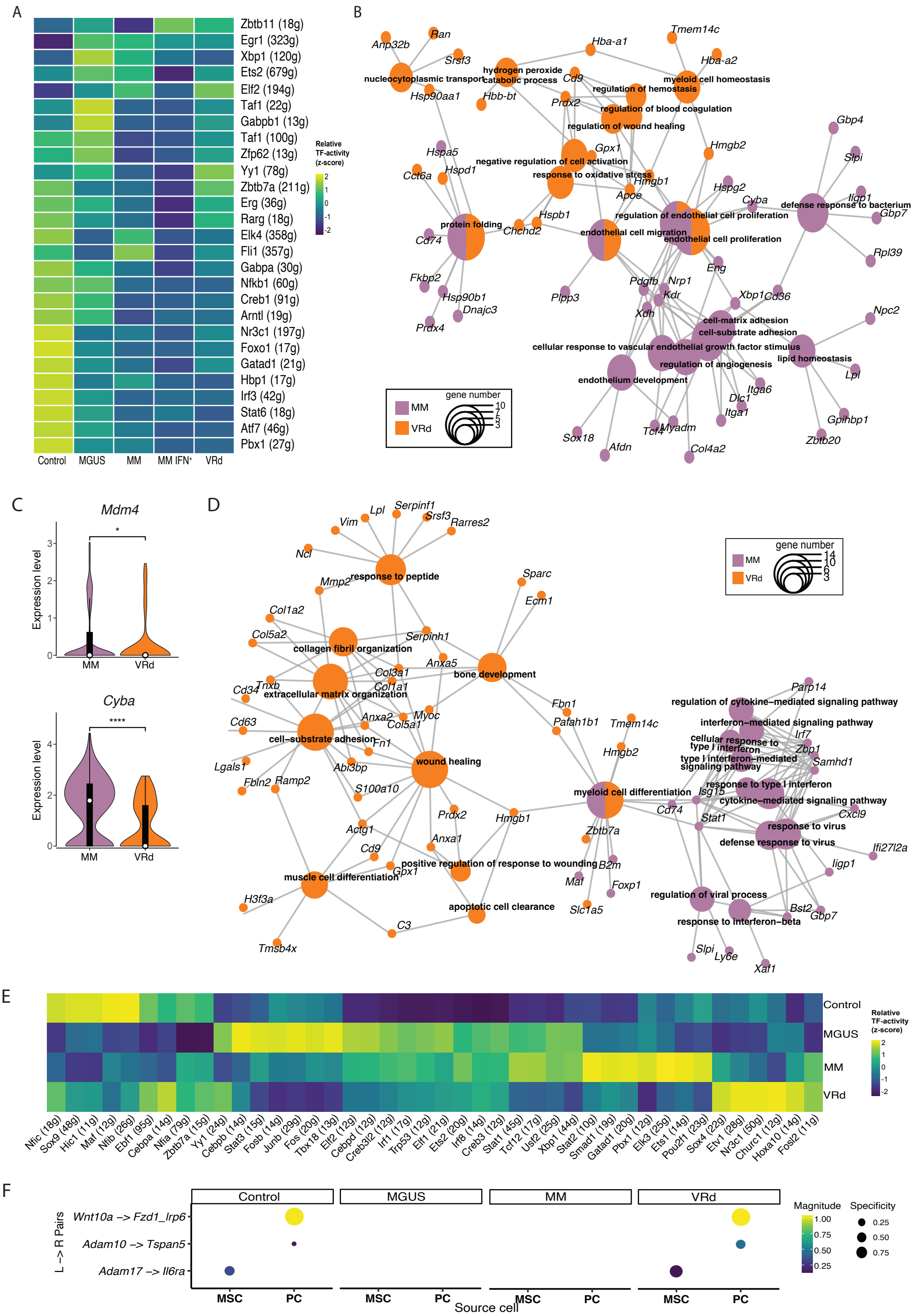

Supplementary Figure 14: Additional information of the transcriptional remodeling of EC and MSC following VRd treatment. (A) Heatmap representing z-scaled mean regulon activity score of inferred top specific regulons for EC in each condition. (B) Cnetplot showing the links

between genes and biological processes derived from the comparison between VRd-treated and non-IFN MM EC. Node size reflects the number of significantly enriched genes in the node and colors the group condition. (C) Violin plots displaying the expression of *Mdm4* and *Cyba* in VRd-treated and MM $BI_{c\gamma 1}$ MSC. (D) Cnetplot showing the links between genes and biological processes derived from the comparison between VRd-treated and MM MSC. Node size reflects the number of significantly enriched genes in the node and colors the group condition (E) Heatmap representing z-scaled mean regulon activity score of inferred top specific regulons for MSC in each condition. (F) Dot plots representing L-R pairs common to control and VRd-treated mice involved in the communication between MSC and PC. Dot color represent the strength and size indicate the specificity of the interaction.

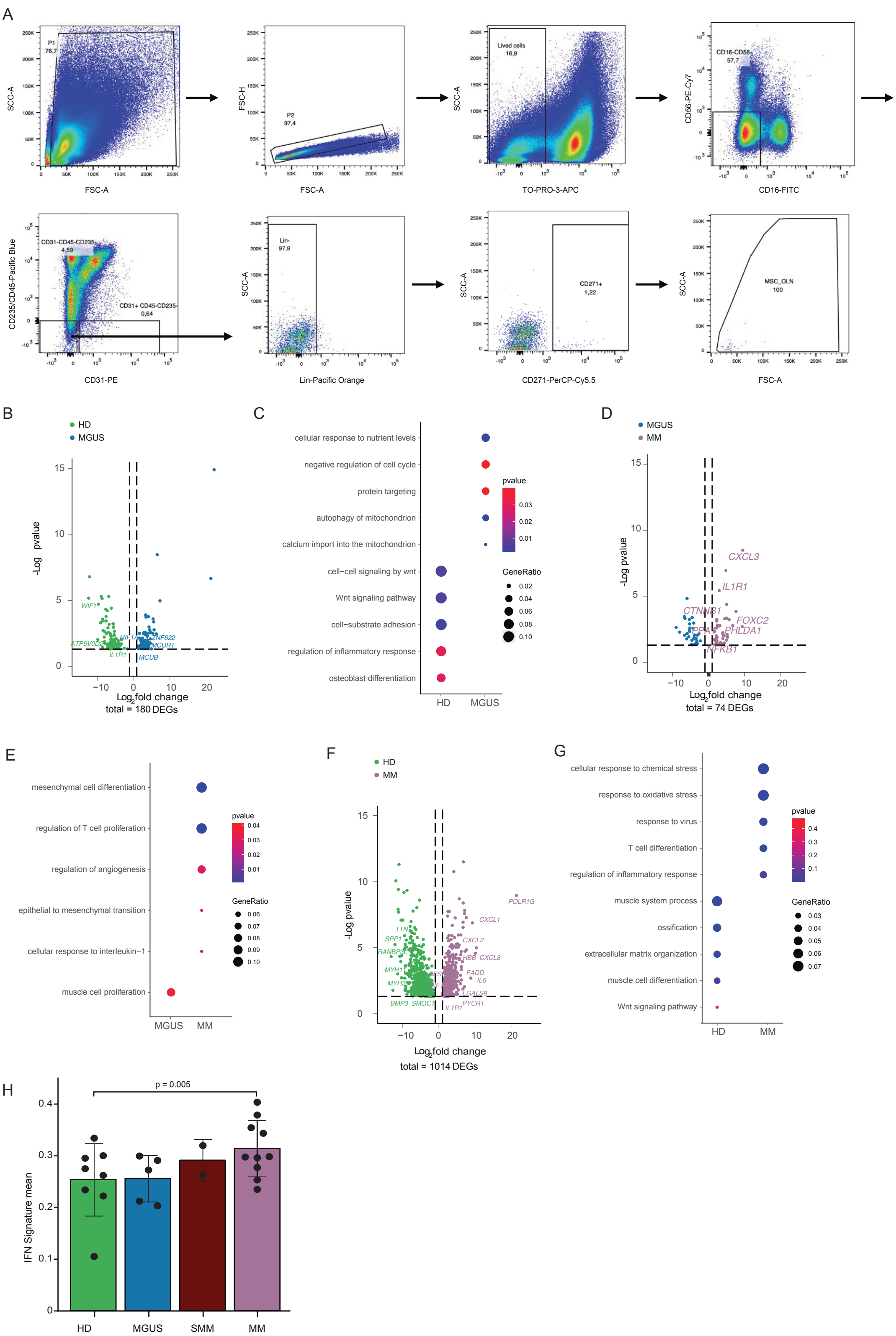

Supplementary Figure 15: Cell sorting strategy and additional information on the transcriptional alterations of human MSC during myeloma progression. (A) Sorting gating strategy for isolation of human BM MSC (TO-PRO-3-, CD16-, CD56-, CD45-, CD235-, CD31-,

Lin$^-$, CD271$^+$). (B) Volcano plot of the DEGs between MGUS-MSC and HD-MSC. The y-axis represents the -log_{10}(p-value), and the x-axis represents the log_2FC of the gene. The dot's color represents the patient's disease condition. (C) GO ORA between MSC from HD and MGUS patients. (D-E) Similar to (B-C) for the comparison between MSC from MGUS and MM patients. (F-G) Similar to (B-C) for the comparison between HD-MSC and MM-MSC. (H) Bar plot depicting the mean IFN signature score across different disease conditions. Points represent individual human samples; HD n =8, MGUS n = 5, SMM n = 2 and MM n = 10. Data are presented as mean ± SD. Statistical significance was determined using an unpaired two-sided Student's t-test (assuming equal variances), exact p values provided.

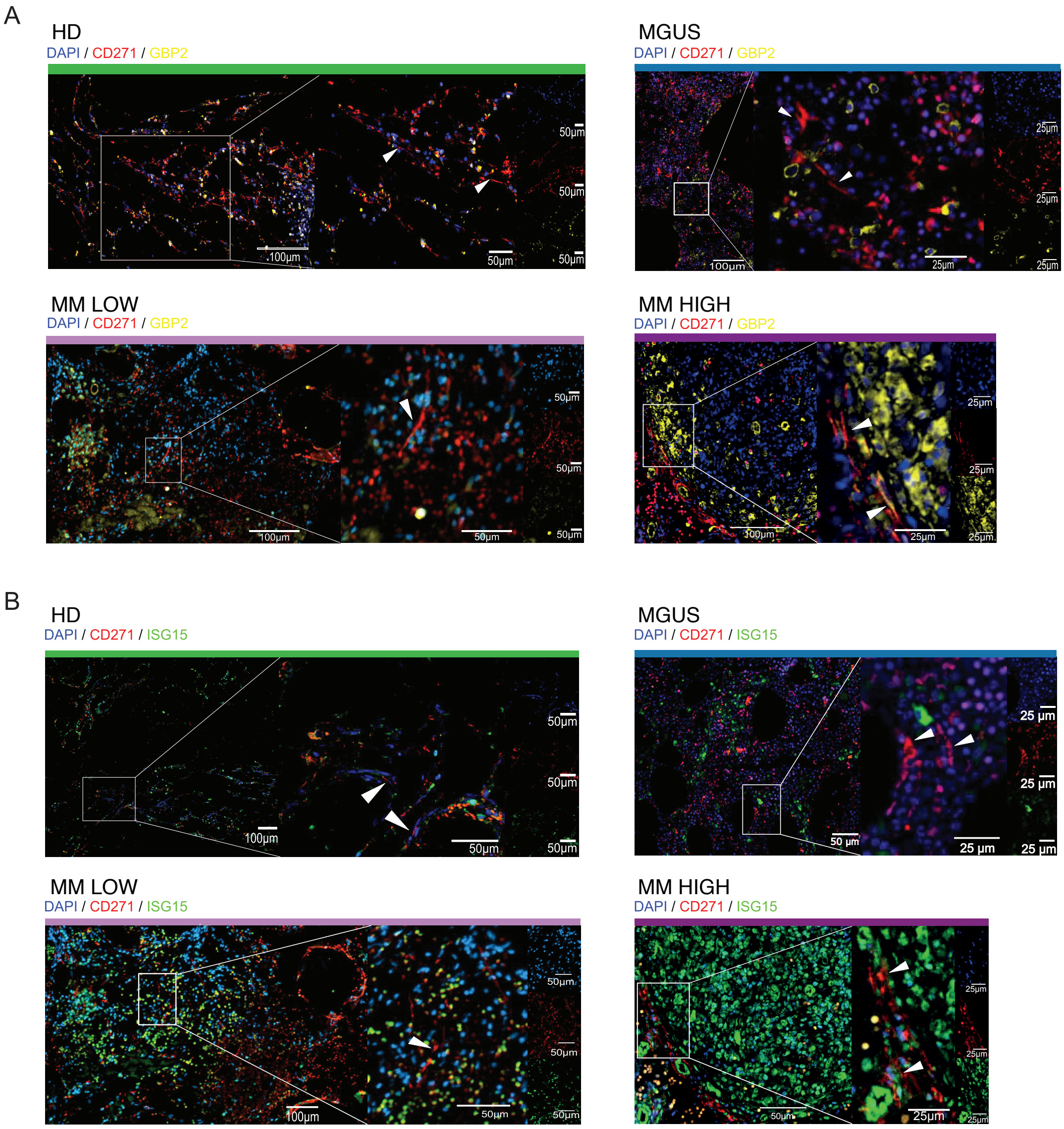

Supplementary Figure 16: Expanded immunofluorescence analysis of human MSC during myeloma progression. (A-B) IF staining of MSC (CD271, red), IFN response genes (*GBP2*, yellow (A); *ISG15,* green (B)), and nucleus (DAPI) (blue) in FFPE biopsies from HD and archived paraffined human BM biopsies of MGUS and MM patients with high (MM HIGH) and low IFN signal (MM LOW) in stromal cells (see Supplementary Data 13). White arrows point MSC CD271+ in HD, MGUS and MM LOW. In MM HIGH white arrows point MSC CD271+ IFN+ (GBP2/ISG15). Representative images from HD (n = 3), MGUS (n = 4), MM low IFN (n = 3), MM high IFN (n = 3) samples. Scale bars, 50 µm.

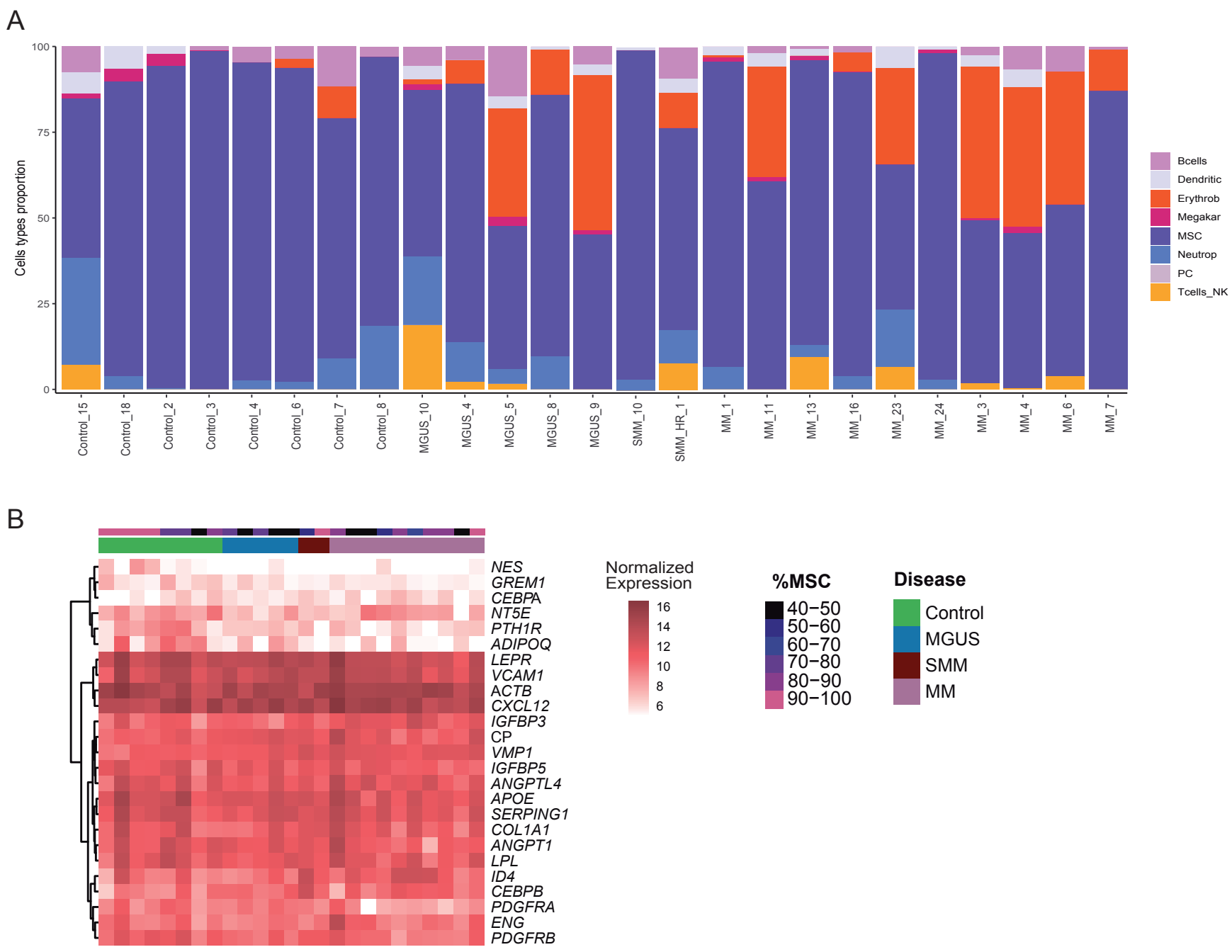

Supplementary Figure 17: Evaluation of MSC proportion in bulk RNA-seq human samples. (A) Bar plot showing the percentage of different cell types present on each sample estimated by CibersortX. Each bar represents one human sample. (B) Heatmap of MSC canonical markers gene expression correlated with MSC percentage estimated by CibersortX.

SUPPLEMENTARY TABLES

Supplementary Data 1. Differentially expressed genes between MGUS and control EC. Differential expression was assessed using two-sided MAST with Bonferroni correction.

Supplementary Data 2. Differentially expressed genes between MM and MGUS EC. Differential expression was assessed using two-sided MAST with Bonferroni correction.

Supplementary Data 3. Gene Ontology over-representation analysis across MSC stages. Adjusted P values were calculated using the Benjamini–Hochberg method.

Supplementary Data 4. Differentially expressed genes between MGUS and control MSC. Differential expression was assessed using two-sided MAST with Bonferroni correction.

Supplementary Data 5. Differentially expressed genes between MM and MGUS MSC. Differential expression was assessed using two-sided MAST with Bonferroni correction.

Supplementary Data 6. Full list of significant ligand–receptor interactions between EC and PC, and between MSC and PC. Interactions were identified using LIANA aggregate ranking.

Supplementary Data 7. Differentially expressed genes between VRd-treated and untreated MM EC. Differential expression was assessed using two-sided MAST with Bonferroni correction.

Supplementary Data 8. Differentially expressed genes between VRd-treated and untreated MM MSC. Differential expression was assessed using two-sided MAST with Bonferroni correction.

Supplementary Data 9. Differential expression analysis comparing human MGUS-MSC and healthy donor MSC. Differential expression was performed using the two-sided Wald test in DESeq2 with Benjamini–Hochberg correction.

Supplementary Data 10. Differential expression analysis comparing human MM-MSC and MGUS-MSC. Differential expression was performed using the two-sided Wald test in DESeq2 with Benjamini–Hochberg correction.

Supplementary Data 11. Differential expression analysis comparing human MM-MSC and healthy donor MSC. Differential expression was performed using the two-sided Wald test in DESeq2 with Benjamini–Hochberg correction.

Supplementary Data 12. Description of mice used in the study for scRNA-seq and immunofluorescence analyses.

Supplementary Data 13. Clinical characteristics of human individuals used for bulk RNA-seq and immunofluorescence analyses.

REFERENCES

1 de Jong MME, Kellermayer Z, Papazian N, Tahri S, Hofste op Bruinink D, Hoogenboezem R *et al.* The multiple myeloma microenvironment is defined by an inflammatory stromal cell landscape. *Nature Immunology 2021 22:6* 2021; **22**: 769–780.
