# Supplementary material for: Stromal and endothelial transcriptional changes during progression from MGUS to myeloma and after treatment response
Source: Nat Commun. 2026 Jun 23;17:7893. doi: 10.1038/s41467-026-74389-y (PMC13443197; doi:10.1038/s41467-026-74389-y)
Supplement: Supplementary file 2 [file 41467_2026_74389_MOESM2_ESM.pdf]

Description of Additional Supplementary Files

Supplementary Data 1: Differentially expressed genes between MGUS and control EC. Differential expression was assessed using two-sided MAST with Bonferroni correction.

Supplementary Data 2: Differentially expressed genes between MM and MGUS EC. Differential expression was assessed using two-sided MAST with Bonferroni correction.

Supplementary Data 3: Gene Ontology over-representation analysis across MSC stages. Adjusted P values were calculated using the Benjamini–Hochberg method.

Supplementary Data 4: Differentially expressed genes between MGUS and control MSC. Differential expression was assessed using two-sided MAST with Bonferroni correction.

Supplementary Data 5: Differentially expressed genes between MM and MGUS MSC. Differential expression was assessed using two-sided MAST with Bonferroni correction.

Supplementary Data 6: Full list of significant ligand–receptor interactions between EC and PC, and between MSC and PC. Interactions were identified using LIANA aggregate ranking.

Supplementary Data 7: Differentially expressed genes between VRd-treated and untreated MM EC. Differential expression was assessed using two-sided MAST with Bonferroni correction.

Supplementary Data 8: Differentially expressed genes between VRd-treated and untreated MM MSC. Differential expression was assessed using two-sided MAST with Bonferroni correction.

Supplementary Data 9: Differential expression analysis comparing human MGUS-MSC and healthy donor MSC. Differential expression was performed using the two-sided Wald test in DESeq2 with Benjamini–Hochberg correction.

Supplementary Data 10: Differential expression analysis comparing human MM-MSC and MGUS-MSC. Differential expression was performed using the two-sided Wald test in DESeq2 with Benjamini–Hochberg correction.

Supplementary Data 11: Differential expression analysis comparing human MM-MSC and healthy donor MSC. Differential expression was performed using the two-sided Wald test in DESeq2 with Benjamini–Hochberg correction.

Supplementary Data 12: Description of mice used in the study for scRNA-seq and immunofluorescence analyses.

Supplementary Data 13: Clinical characteristics of human individuals used for bulk RNAseq and immunofluorescence analyses.
