# Supplementary material for: Stromal and endothelial transcriptional changes during progression from MGUS to myeloma and after treatment response
Source: Nat Commun. 2026 Jun 23;17:7893. doi: 10.1038/s41467-026-74389-y (PMC13443197; doi:10.1038/s41467-026-74389-y)
Supplement: Supplementary file 3 [file 41467_2026_74389_MOESM4_ESM.pdf]

nature portfolio

Corresponding author(s): Felipe Propser
Last updated by author(s): Aug 7, 2024

Reporting Summary

Nature Portfolio wishes to improve the reproducibility of the work that we publish. This form provides structure for consistency and transparency in reporting. For further information on Nature Portfolio policies, see our Editorial Policies and the Editorial Policy Checklist.

Statistics

For all statistical analyses, confirm that the following items are present in the figure legend, table legend, main text, or Methods section.

n/a	Confirmed	
☐	☒	The exact sample size (*n*) for each experimental group/condition, given as a discrete number and unit of measurement
☐	☒	A statement on whether measurements were taken from distinct samples or whether the same sample was measured repeatedly
☐	☒	The statistical test(s) used AND whether they are one- or two-sided<br>*Only common tests should be described solely by name; describe more complex techniques in the Methods section.*
☐	☒	A description of all covariates tested
☐	☒	A description of any assumptions or corrections, such as tests of normality and adjustment for multiple comparisons
☐	☒	A full description of the statistical parameters including central tendency (e.g. means) or other basic estimates (e.g. regression coefficient) AND variation (e.g. standard deviation) or associated estimates of uncertainty (e.g. confidence intervals)
☐	☒	For null hypothesis testing, the test statistic (e.g. *F*, *t*, *r*) with confidence intervals, effect sizes, degrees of freedom and *P* value noted<br>*Give P values as exact values whenever suitable.*
☒	☐	For Bayesian analysis, information on the choice of priors and Markov chain Monte Carlo settings
☒	☐	For hierarchical and complex designs, identification of the appropriate level for tests and full reporting of outcomes
☐	☒	Estimates of effect sizes (e.g. Cohen's *d*, Pearson's *r*), indicating how they were calculated

Our web collection on statistics for biologists contains articles on many of the points above.

Software and code

Policy information about availability of computer code

Data collection

scRNAseq data were sequenced on Illumina NEXTseq2000.
Flow cytometry data was collected used FACS Canto II cytometer.
Fluorescence images were acquired using the Vectra Polaris Multispectral Imaging System (Perkin Elmer).

Data analysis

FACS sorting was performed on a BD FACSAria II sorter. Cell viability was determined using Nexcelom Cellometer. We also used Flowjo 10.7.1 for FACS analysis.
Code for pre-processing and basic analysis of single-cell RNA-seqdata of mice was conducted using CellRanger (v6.1.1), R (v4.1.3), Seurat (v4.3.0). Differentially expressed genes (DEGs) were determined with the Seurat FindMarkers function for transcripts detected in at least 30% of cells using a log2-fold-change (log2FC) threshold of 0.5 with MAST. Significant DEGs for each group were used as input for a Gene Ontology (GO) Over-representation analysis (ORA) using the clusterProfiler R package (v 4.14.6). Gene Set Enrichment Analysis (GSEA) was also calculated using the GO and Hallmark gene sets from the Molecular Signatures Database v.7.5.1 in the list of DEGs ranked according to log2FC. The MM interferon (IFN)-signature score was computed using Seurat's AddModuleScore function using the top 50 DEGs of the MM2 EC subgroup calculated with the Seurat FindMarkers function (parameters min.pct=0.4 thresh.use=1, test='MAST'). Gene Regulatory Network (GRN) activity was interrogated following the workflow implemented by SCENIC(v 1.3.1). To assess receptor-ligand interactions between the two BM niche populations (EC and MSC) and the myeloma PC, we used Liana v.0.1.13.
Before proceeding with the analysis, of human bulkRNA-seq we evaluated the MSC proportion in our samples using CIBERSORTx web portal and AUCell (v 1.20.2). After determining the proportions of MSC in our samples we proceeded with the subsequent analysis in R (v 4.1.3). Batch effect was corrected using the sva package (v 3.46), gene count estimates were normalized, prefiltered, and used for determining the differential expression of genes between the different groups through the DESeq2 package (v 1.38.3). The proportions of MSC estimated by CIBERSORTx were included in the model as a covariate. ORA was conducted using the functions enrichGO and compareCluster in the package

clusterProfiler (v 4.6.2).AUCell method was applied to quantify the IFN signature. The transcription factor activity was estimated by Decoupler (v 2.9.1). The code used for single-cell RNA-seq and bulk RNA-seq analyses in this study is publicly available at https://github.com/icenzano/NicheMGUSMM_2025 and permanently archived at Zenodo (https://doi.org/10.5281/zenodo.18631631). The archived release (v1.0.0) corresponds to the version used for the analyses reported in this manuscript.

For manuscripts utilizing custom algorithms or software that are central to the research but not yet described in published literature, software must be made available to editors and reviewers. We strongly encourage code deposition in a community repository (e.g. GitHub). See the Nature Portfolio guidelines for submitting code & software for further information.

Data

Policy information about availability of data

All manuscripts must include a data availability statement. This statement should provide the following information, where applicable:

- Accession codes, unique identifiers, or web links for publicly available datasets
- A description of any restrictions on data availability
- For clinical datasets or third party data, please ensure that the statement adheres to our policy

The murine BM single-cell RNA-seq data generated in this study have been deposited in the NCBI Gene Expression Omnibus (GEO) database under accession GSE305390 (https://www.ncbi.nlm.nih.gov/geo/query/acc.cgi?acc=GSE305390). The human BM MSC bulk RNA-seq data generated in this study have been deposited in the GEO database under accession GSE305389 (https://www.ncbi.nlm.nih.gov/geo/query/acc.cgi?acc=GSE305389). These datasets are linked under BioProject PRJNA1303025 (https://www.ncbi.nlm.nih.gov/bioproject/PRJNA1303025).The public scRNA-seq datasets of the non-hematopoietic BME of newly diagnosed individuals with MM and individuals without MM used in this study are available in the ArrayExpress database under accession code E-MTAB-9139 (https://www.ebi.ac.uk/biostudies/arrayexpress/studies/E-MTAB-9139), and were used to validate the enrichment of the IFN signature in a subset of MM patients. The bulk RNA-sequencing datasets of MSC from individuals with MM at diagnosis and after induction treatment used in this study are available in the ArrayExpress database under accession code E-MTAB-9285 (https://www.ebi.ac.uk/biostudies/arrayexpress/studies/E-MTAB-9285), and were used to confirm suppression of the IFN signature following therapy. Source data are provided with this paper.

Research involving human participants, their data, or biological material

Policy information about studies with human participants or human data. See also policy information about sex, gender (identity/presentation), and sexual orientation and race, ethnicity and racism.

Reporting on sex and gender	For the human study, biological sex information was recorded. Samples from male and female participants with newly diagnosed MGUS, smoldering multiple myeloma (SMM), multiple myeloma (MM), and healthy adult donors were included.
Reporting on race, ethnicity, or other socially relevant groupings	Race, ethnicity, or other socially relevant groupings were not collected or used in this study, and no analyses were performed on these variables.
Population characteristics	Individuals ranging 39-82 years of age (see Supplementary Data 13)
Recruitment	Samples for bulkRNAseq BM aspirates and BM samples derived from individuals undergoing orthopedic surgery were collected between 2014 and 2023. For IF staining, archived paraffined human BM biopsies and Bone chips from healthy donors were collected between 2021 and 2025.<br>Participants were included based on diagnosis, clinical availability, and sample availability.
Ethics oversight	This study was approved by the University of Navarra's Institutional Review Board (Project 2022.100mod1) and the Navarra Department of Health Ethics Committee for Drug Research (CEIm, PI_2022/92 MS-1) and conducted under the Declaration of Helsinki. Informed consent was obtained from all patients. Personal data was kept confidential following the Organic Law 3/2018 on personal data protection and Spanish Law 14/2007 on Biomedical research. All samples were coded and only authorized personnel could link participant identity to sample codes.

Note that full information on the approval of the study protocol must also be provided in the manuscript.

Field-specific reporting

Please select the one below that is the best fit for your research. If you are not sure, read the appropriate sections before making your selection.

☒ Life sciences ☐ Behavioural & social sciences ☐ Ecological, evolutionary & environmental sciences

For a reference copy of the document with all sections, see nature.com/documents/nr-reporting-summary-flat.pdf

Life sciences study design

All studies must disclose on these points even when the disclosure is negative.

Sample size	No statistical method was used to predetermine sample size. Sample sizes were based on avail-ability of the relevant mouse models and human samples and were consistent with previous studies of related designs. For each single cell RNA sequencing (scRNA-seq) experiment, a pool of four, five or six adult mice per group compromising control, MGUS, and MM stages, as well as velcade, lenalidomide and dexamethasone (VRd)-treated subjects, was used. For human samples, the sample size was limited by the availabilty of samples compiled in the biobank of Clinica Universidad de Navarra.

Data exclusions	We have used the following pre-established cell exclusion criteria: low quality, hematopoietic cells, and doublets were filtered out computationally (see Methods). Cells with more than 5% (mouse sample) or 10% (human sample) mitochondrial genes were also removed.
Replication	Computational findings were verified across independent biological samples and complementary cohorts obtaining consistent results. Specifically, the IFN-signature was validated across BIcγ1 and MIcγ1 murine models, human bulk RNA-seq cohorts, and publicly available datasets of human Multiple Myeloma (MM) and treated subjects. To further support these findings, key transcriptomic changes were validated via qPCR using mesenspheres derived from BIcγ1 and control mice. All qPCR and mesensphere assays were performed with three technical replicates, representing N=2/3 independent biological replicates per group. Furthermore, experimental findings were replicated in bone biopsy samples from both mice and humans. A minimum of N=3 biological replicates per condition was utilized to ensure statistical robustness, with the exception of specific groups where sample availability was strictly limited (MGUS BIcγ1, N=2; VRd-treated subjects, N=1).
Randomization	Randomization was not applicable to this study as mice were allocated to experimental groups based on their specific genotypes (YFPcγ1 BIcγ1 and MIcγ1) and disease progression stages. However, within each genotype, mice were age- and sex-matched to control for potential covariates. For human datasets, patients were allocated to groups based on their clinical diagnosis (Control, MGUS, or MM) according to international staging criteria. Age-matched eldelry donors were used as controls. Subjects for both sexes were included randomly in all conditions.
Blinding	Blinding was not feasible during sample collection and processing because investigators needed to identify specific transgenic mouse models, treatment groups, and clinical diagnoses to perform experimental procedures. To minimize bias, computational analyses were performed using predefined pipelines and quality-control criteria. Quantification of imaging-based experiments was performed blinded to group identity whenever feasible.

Reporting for specific materials, systems and methods

We require information from authors about some types of materials, experimental systems and methods used in many studies. Here, indicate whether each material, system or method listed is relevant to your study. If you are not sure if a list item applies to your research, read the appropriate section before selecting a response.

Materials & experimental systems

n/a	Involved in the study
☐	☒ Antibodies
☒	☐ Eukaryotic cell lines
☒	☐ Palaeontology and archaeology
☐	☒ Animals and other organisms
☒	☐ Clinical data
☒	☐ Dual use research of concern
☒	☐ Plants

Methods

n/a	Involved in the study
☒	☐ ChIP-seq
☐	☒ Flow cytometry
☒	☐ MRI-based neuroimaging

Antibodies

Antibodies used

Mouse FACS
PE-CD138 (Biolegend, Cat#142503, clone 281-2) 1:100 dilution
APC-B220 (Biolegend, Cat#103211) 1:100 dilution
BV421-IgM (Biolegend, Cat#406517, clone RMM-1) 1:100 dilution
Biotin antimouse-human CD3 Cat#100304 1:50 clone 145-2C11
Biotin antimouse-human Gr1 Cat#108404 1:50 clone RB6-8C5
Biotin antimouse-human B220 Cat#103204 1:50 clone RA3-6B2
Biotin antimouse-human CD11b Cat#101204 1:50 clone M1/70
Biotin antimouse-human TER119 Cat#116204 1:50 clone TER119
Streptavidin magnetics microbeads (Miltneyi, Cat#1130-048-101, dilution 100ul/mouse)
APC-Cy7 labeled streptavidin (Biolegend-dedham, Cat#1405208, dilution 1:200)
APC labeled anti-CD45 (Biolegend-dedham, Cat#1103112, dilution 1:200, clone 30-F11)
APC labeled anti- Ter119 (Biolegend-dedham, Cat#1116212, dilution 1:200, clone TER119)
BV510 labeled anti-CD45 (Biolegend-dedham, Cat#154103137, dilution 1:200, clone 30-F11)
Vybrant dye orange (VDO) (Thermofisher scientific, Cat#1V35005, concentration 0.05μM)
Annexin V -FITC (PHARMINGEN, Cat#1556419, dilution 1μl/mouse)
7AAD 5 μL (BD Bioscience, Cat#1559925, dilution 5μl up to 1x106 cells)

Human FACS:
BV510 CD3 Cat#317332, Biolegend-dedham, clone OKT3, dilution 3ul/test- test 25x106cells
BV510 CD45 Cat#560779, Biolegend-dedham, clone HI30 dilution 3ul/test- test 25x106cells
BV510 CD19 Cat#363020, Biolegend-dedham, clone SJ25C1 dilution 3ul/test- test 25x106cells
BV510 CD64 Cat#305028, Biolegend-dedham, clone 10.1 dilution 3ul/test- test 25x106cells
BV421 labeled anti-CD235 (Biolegend-dedham, clone HI264 Cat#349108, dilution 1:100)
BV421 labeled anti-CD45 (Biolegend-dedham, clone HI30 Cat#304032, dilution 1:100)
FITC labelled anti-CD16 (Beckman, clone 3G8 Cat#B49215 dilution 1:100)
PE labeled anti-CD31 (BD Bioscience, clone WM59 Cat#555446, dilution 1:50)
PECγ7 labeled anti-CD56 (Beckman, clone C218, Cat#A21692, dilution 1:50)

APC-Cy7 labeled anti-CD9 (Biolegend-dedham, clone HI9a Cat#312114, dilution 1:100)
PerCP-Cy5.5 labelled anti-CD271 (Biolegend-dedham, clone ME20.4 Cat#345112, dilution 1:100)
To-PRO-3 IODIDE (642/661) (Thermofisher scientific,Cat#T3605, dilution 10µl/sample)

Mouse immunofluorescence staining

Rat anti-Endomucin antibody (clone V.7C7) (Santa Cruz Technology 1: 50 dilution, Cat#sc-65495)
Mouse anti-CD271 monoclonal antibody (NGF Receptor) (clone ME20.4) (Invitrogen, 1: 50 dilution, Cat#14-9400-82)
Rabbit GBP2 Polyclonal Antibody (Proteintech, 1: 100 dilution, Cat#11854-1-AP)
Rabbit ISG15 Polyclonal Antibody (Proteintech, 1: 100 dilution, Cat#15981-1-AP)
VEGF Monoclonal Antibody (Clone JH121) (Invitrogen, 1: 20 dilution, Cat#MA5-13182)
Integrin Beta 1/CD29 Polyclonal antibody (Proteintech, 1: 200 dilution, Cat#12594-1-AP)
Complement C3 Polyclonal Antibody (Invitrogen, 1: 100 dilution, Cat#PA1-29715)
Anti-LRP1 antibody (abcam, 1: 50 dilution, Cat#ab92544, clone EPR3724)
CD304 (Neuropilin-1) Recombinant Rabbit Monoclonal Antibody (clone ST05-30) (Invitrogen, 1: 50 dilution, Cat#MA5-32179)
Goat anti-GFP polyclonal (Novus Biologicals, 1:4000, Cat#NB100-1770).

Secondary antibodies in a 1:200 dilution:
Goat anti-Rabbit IgG (H+L) Highly Cross-Adsorbed Secondary Antibody, Alexa Fluor™ 647 (Invitrogen, Cat#A-21245)
Goat anti-Rat IgG (H+L) Cross-Adsorbed Secondary Antibody, Alexa Fluor™ 568 (Invitrogen, Cat#A11077)
Goat anti-Mouse IgG (H+L) Highly Cross-Adsorbed Secondary Antibody, Alexa Fluor™ Plus 488 (Invitrogen, Cat#A32723)
Goat anti-Rabbit IgG (H+L) Cross-Adsorbed Secondary Antibody, Alexa Fluor™ 488 (Invitrogen, Cat#A11034)
Goat anti-Mouse IgG (H+L) Cross-Adsorbed Secondary Antibody, Alexa Fluor™ 568 (Invitrogen, Cat#A11004)
Donkey anti-Mouse IgG (H+L) Highly Cross-Adsorbed Secondary Antibody, Alexa Fluor™ 555 (Invitrogen, Cat#A-31570)
Donkey anti-Goat IgG (H+L) Cross-Adsorbed Secondary Antibody, Alexa Fluor™ 488 (Invitrogen, Cat#A-11055).

Nuclei were stained with DAPI (Vectashield 1:10 dilution, Cat# V6H-1200-10).

Human
Mouse anti-CD271 monoclonal antibody (NGF Receptor) (ME20.4) (Invitrogen, 1: 50 dilution, Cat#14-9400-82)
Rabbit GBP2 Polyclonal Antibody (Proteintech, 1: 100 dilution, Cat#11854-1-AP)
Rabbit ISG15 Polyclonal Antibody (Proteintech, 1: 100 dilution, Cat#15981-1-AP)

Secondary antibodies in a 1:200 dilution:
Goat anti-Rabbit IgG (H+L) Highly Cross-Adsorbed Secondary Antibody, Alexa Fluor™ 488 (Invitrogen, Cat#A11034)
Goat anti-Rabbit IgG (H+L) Highly Cross-Adsorbed Secondary Antibody Alexa Fluor™ 647 (Invitrogen, Cat#A-21245)
Goat anti-Mouse IgG (H+L) Cross-Adsorbed Secondary Antibody, and Alexa Fluor™ 568 (Invitrogen,Cat# A11004).

Nuclei were stained with DAPI (Vectashield 1:10 dilution, Cat# V6H-1200-10).

Mesensphere isolation and culture
Biotin Rat Anti-Mouse CD45 (BD Bioseciences, Cat#553078, dilution 1:100, clone 30-F11)
Biotin Rat Anti-Mouse TER-119/Erythroid Cells (BD Bioseciences, Cat#553672, dilution 1:100 clone TER119)
BD IMag™ Streptavidin Particles Plus - DM (BD Bioseciences, Cat#557812)

Coculture and cell-cell interaction imaging
VEGF Monoclonal Antibody (Clone JH121) (Invitrogen, 1: 20 dilution, Cat#MA5-13182)
Integrin Beta 1/CD29 Polyclonal antibody (Proteintech, 1: 200 dilution, Cat#12594-1-AP)
CD304 (Neuropilin-1) Recombinant Rabbit Monoclonal Antibody (Clone ST05-30) (Invitrogen, 1: 50 dilution, Cat#MA5-32179)

Secondary antibodies (1:200 dilution):
Goat anti-Rabbit IgG (H+L) Highly Cross-Adsorbed Secondary Antibody, Alexa Fluor™ 647 (Invitrogen, Cat#A-21245)
Goat anti-Mouse IgG (H+L) Highly Cross-Adsorbed Secondary Antibody Alexa Fluor™ Plus 488 (Invitrogen, Cat#A32723).
Nuclei were counterstained with DAPI (Invitrogen, Cat#D1306; 1:1000).

Validation

The antibodies listed previously have been extensively used in the field for the prospective isolation of the populations of interest. Data generated by us and published by others (see below) demonstrate their specificity.
To isolate mouse BM microenvironment cells (doi.org/10.1016/j.cell.2019.04.040, doi.org/10.1038/s41556-019-0439-6, doi.org/10.1016/j.celrep.2019.06.031, doi.org/10.3389/fcell.2021.653308 and doi.org/10.1016/j.stem.2020.11.004).
For isolation of human BM endothelial and mesenchymal cells (doi.org/10.1016/j.celrep.2017.12.070, doi.org/10.1182/blood-2004-09-3606, doi.org/10.1002/ajh.2830380408, doi.org/10.1016/s0301-472x(02)00812-3, doi.org/10.1089/scd.2016.0169, doi.org/10.1016/j.stem.2020.11.004 and doi.org/10.1016/j.xpro.2021.100538)

Animals and other research organisms

Policy information about studies involving animals; ARRIVE guidelines recommended for reporting animal research, and Sex and Gender in Research

Laboratory animals

BIcy mice come from crossing:

B6(Cg)-Gt (ROSA)26Sortm4(Ikbkb)Rsky/J mice; The Jackson Laboratory; Strain code: 008242
with
B6. Cg-Tg (BCL2)22Wehi/J The Jackson Laboratory; Strain code: 002319

with
cγ1-cre mice (B6.129P2(Cg)-Ighg1tm1(cre)Cgn/J mice The Jackson Laboratory; Strain code: 010611

MIcγ mice:

B6(Cg)-Gt (ROSA)26Sortm4(Ikbkb)Rsky/J The Jackson Laboratory; Strain code: 008242
with
C57BL/6N-Gt (ROSA)26Sortm13(CAG-MYC, -CD2*) Rsky/J The Jackson Laboratory; Strain code: 020458
and
cγ1-cre mice (B6.129P2(Cg)-Ighg1tm1(cre)Cgn/J mice The Jackson Laboratory; Strain code: 010611

YFP mice:
B6.129X1-Gt (ROSA)26Sortm1(EYFP)Cos/J mice; The Jackson Laboratory, Strain Code: 006148

BIcγ1 mice were crossed with a humanized cereblon (Crbn I391V) gene strain: C57BL/6-Crbntm2.1Ble/J The Jackson Laboratory, Strain Code:035831

MGUS BIcγ1 were around 180 days old.
MGUS MIcγ1 were around 130 days old.
MM BIcγ1 were around 217-410 days old
MM MIcγ1 were around 260 days old
YFP control mice were around 250 days old.

MM BIcγ1 Crbn treated with VRd were around 360 days old.
Mice were kept under specific pathogen-free conditions and ligth/dark cycles of 12h. The temperature was constantly mantained between 18-23ºC

Wild animals	NA
Reporting on sex	Mice of both sexes were used in the study.
Field-collected samples	NA
Ethics oversight	Mice were kept under SPF conditions in the Center for Applied Medical Research (CIMA) animal facilities at the University of Navarra. All animal experimentation was approved by the Ethics Committee for Clinical Research at the University of Navarra (protocol CEEA082-20).

Note that full information on the approval of the study protocol must also be provided in the manuscript.

Plants

Seed stocks	*Report on the source of all seed stocks or other plant material used. If applicable, state the seed stock centre and catalogue number. If plant specimens were collected from the field, describe the collection location, date and sampling procedures.*
Novel plant genotypes	*Describe the methods by which all novel plant genotypes were produced. This includes those generated by transgenic approaches, gene editing, chemical/radiation-based mutagenesis and hybridization. For transgenic lines, describe the transformation method, the number of independent lines analyzed and the generation upon which experiments were performed. For gene-edited lines, describe the editor used, the endogenous sequence targeted for editing, the targeting guide RNA sequence (if applicable) and how the editor was applied.*
Authentication	*Describe any authentication procedures for each seed stock used or novel genotype generated. Describe any experiments used to assess the effect of a mutation and, where applicable, how potential secondary effects (e.g. second site T-DNA insertions, mosiacism, off-target gene editing) were examined.*

Flow Cytometry

Plots

Confirm that:

☒ The axis labels state the marker and fluorochrome used (e.g. CD4-FITC).

☒ The axis scales are clearly visible. Include numbers along axes only for bottom left plot of group (a 'group' is an analysis of identical markers).

☒ All plots are contour plots with outliers or pseudocolor plots.

☒ A numerical value for number of cells or percentage (with statistics) is provided.

Methodology

Sample preparation	Isolation and fluorescence-activated cell sorting of murine BM microenvironment and PC<br>Mice were euthanized by CO2 asphyxiation. Bones from the humerus, radius, iliac crests, femurs, and tibia were harvested in

Phosphate-buffered saline (PBS) 1X containing 2 % Fetal bovine serum (FBS) and two mM EDTA (modified PBS). All steps were performed on ice to preserve cell viability and RNA integrity. Muscles and soft tissue were removed from the bones, and bone marrow (BM) cells were obtained by crushing in modified PBS. Cells were then filtered through a 70 µm cell strainer, and red blood cells were lysed with ACK buffer (NH4Cl 150 mM, KHCO3 10 mM, and Na2EDTA 0,1 mM) for 10 minutes at room temperature (RT). 25 µl were stained with PE-CD138, APC-B220, and BV421-IgM antibodies (Biolegend) and analyzed by flow cytometry to determine PC infiltration (supplementary Data 12). The remaining bone fragments were collected on a 50 ml conical tube, digested with the appropriate volume of PBS (3ml/mouse) with 0.3% collagenase I, and dispase (5U/ml) for 15 min at 37ºC and shaking at 200 rpm. FBS, representing 10% of the digestion volume, was added to stop the digestion of collagenase. After digestion, the collagenized fractions were filtered through a 70 µm filter into a collection tube and pooled into one sample. To determine live cell concentration and viability, 10 µl of each mouse sample was stained with acridine orange and propidium iodide (AO/PI) solution (Nexcelom) and analyzed with Cellometer K2 Image Cytometer (Nexcelom Bioscience). Cells were subsequently stained for 20 minutes on the ice first in the appropriate volume of modified PBS 1X (3 ml/mouse) with 160 µl/mouse of biotinylated lineage cocktail (Mac1, CD3, Gr1, B220, and TER119) followed by incubation with streptavidin magnetic microbeads (100 µl/mouse). Negative selection was performed using Miltenyi LD columns according to the manufacturer's protocol, and resulting cells from both fractions were counted as described before to confirm selection efficiency.
After selection, the negative fraction was stained with the following combination of conjugated antibodies at a concentration of 1/200: APC-Cy7 labeled streptavidin, BV510 labeled anti-CD45, APC labeled anti-CD45, and APC labeled anti-Ter119. Samples were then stained with 0.05 µM of Vybrant dye orange (VDO) at 37ºC for 30 minutes to label living cells. 7AAD was added to discard apoptotic and dead cells from the sample. Cells were stained with one µl/mouse of Annexin V-FITC for annexin V staining on an appropriate volume of 1X Annexin V binding buffer in the dark for 15 min at RT. Samples were resuspended in 1X Annexin V buffer, and five µl of 7AAD dye (up to 1x106 cells) were added.
BM non-hematopoietic cells and GFP-positive (GFP+) PC were FACS using a BD FACSAria II sorter and collected in PBS 1X supplemented with 0.05% UltraPure BSA for the subsequent sequencing protocol. Cell viability of sorted cells was assessed using a Nexcelom Cellometer, as described above.
Isolation and fluorescence-activated cell sorting of human BM mesenchymal-osteolineage primed cells
After sample collection, red blood cells were lysed twice for 15 minutes at room temperature with rotation following the ratio of 45 ml of ACK lysis buffer per 5 ml of human sample. In the case of samples from healthy adult donors, portions of bones present in the samples were cut into small pieces, and after being crushed, the remaining fragments were collagenized with the appropriate volume of PBS (3ml/sample) with 0.3% collagenase I and dispase (5U/ml) for 15 min at 37ºC and shaking at 200 rpm. FBS, representing 10% of the digestion volume, was added to stop the digestion of collagenase. After digestion, the collagenized fractions were filtered through a 70 µm filter into a collection tube and pooled into one sample. To determine live cell concentration and viability, 10 µl of each sample was stained with acridine orange and propidium iodide (AO/PI) solution (Nexcelom) and analyzed with Cellometer K2 Image Cytometer (Nexcelom Bioscience). Cells were subsequently cryopreserved with FBS and 10% dimethyl sulfoxide (DMSO, Sigma-Aldrich) in liquid nitrogen until use.
Frozen BM samples were thawed at 37ºC in dextran-40 solution supplemented with 25% human albumin at 20%. Cell viability of thawed cells was assessed using Nexcelom Cellometer as described above. The sample was then centrifuged and stained for 30 min on ice with the following combination of conjugated antibodies: : BV510 labelled anti-Lin (including CD3, CD19, CD45 and CD64), BV421 labelled anti-CD235, BV421 labelled anti-CD45, FITC labelled anti-CD16, PE-Cy7 labelled anti-CD56, PE labelled anti-CD31, APC-Cy7 labelled anti-CD9, and PerCP-Cy5.5 labelled anti-CD271. All antibodies were added at a concentration of 1/100 except anti-Lin, where we added 3 µl/test- test 25x106cells, anti-CD16, 10 µl/test- test 25x106cells and 1/50 anti-CD31 and anti-CD56. First, dead cells and debris were excluded by FSC, SSC and adding 10 µl of TO-PRO-3. BM niche MSC were prospectively isolated based on the following immunophenotype: TO-PRO-3-/Lin-/CD45-/CD235-/CD16-/CD56-/CD31-/CD271+. For bulk RNA-sequencing (bulk RNA-seq) studies, MSC were sorted in 100 µl of Lysis/Binding Buffer (Ambion), vortexed, and stored at -80ºC until further processing.

Instrument

FACS sorting was performed on a BD FACSAria II sorter.

Software

Data were analyzed by FACSDiva (BD) or FlowJo (version 10.7.1) software.

Cell population abundance

Describe the abundance of the relevant cell populations within post-sort fractions, providing details on the purity of the samples and how it was determined.

Gating strategy

Cell sorting strategy for the isolation of mouse BME cells (Lived non-hematopoietic BM cells: 7-AAD- Lin- CD45- Ter119- VDO +) and PC (Lived PC: 7-AAD- VDO+ GFP+) (see figure S1).
Cell sorting strategy for isolation of human bone marrow mesenchymal cells (TO-PRO-3-, Lin-, CD45-, CD235-, CD16-, CD56-, CD31-, CD271+, CD146+/-) cells (see figure S15).

☒ Tick this box to confirm that a figure exemplifying the gating strategy is provided in the Supplementary Information.
