# Supplementary material for: Stromal and endothelial transcriptional changes during progression from MGUS to myeloma and after treatment response
Source: Nat Commun. 2026 Jun 23;17:7893. doi: 10.1038/s41467-026-74389-y (PMC13443197; doi:10.1038/s41467-026-74389-y)
Supplement: Supplementary file 4 [file 41467_2026_74389_MOESM5_ESM.pdf]

nature portfolio

Peer Review File

Stromal and Endothelial Transcriptional Changes during Progression from MGUS to Myeloma and after Treatment Response

Corresponding Author: Dr Felipe Prosper

This file contains all reviewer reports in order by version, followed by all author rebuttals in order by version.

Version 0:

Reviewer comments:

Reviewer #1

(Remarks to the Author)
The work of Cenzano et al. shows that non-immune cells in the bone marrow microenvironment, particularly endothelial cells and mesenchymal stem cells, undergo changes during the transition from MGUS to active myeloma and may be partly responsible for the progression of the disease. Interestingly, an IFN signature in the above-mentioned cells appears to be significant for the progression of the disease.
The findings are new and could be of clinical significance, as the authors present convincing mouse models as well as human data. It should be emphasised that, through their collaboration with Benjamin Ebert, the authors are able to show in an elegant model that lenalidomide therapy can reverse these changes. This work provides the basis for further investigation of the IFN signalling pathway and its significance for MM progression.

I only have minor comments:
1. Please check the references: References 12 and 48 are identical.
2. The images are very small. This makes the immunofluorescence images particularly difficult to interpret.
3. A quantification of ISG15 and GBP2 would be useful here.
4. There is also a lack of information about these proteins. They are used as markers, but are hardly discussed. ISG15 in particular, which is also important for a Th1 response, should be discussed.
5. I am having difficulty understanding the meaning of Figures 5A and 5D. It would be helpful
if you could explain them in one or two sentences.

Heiko Bruns

Reviewer #2

(Remarks to the Author)
This study investigates the role of the non-hematopoietic bone marrow microenvironment (BME) in multiple myeloma (MM) progression and treatment response. Using BIcγ1 and MIcγ1 murine models that recapitulate MGUS-to-MM evolution, complemented by validation in patient-derived samples, the authors profile transcriptional changes in endothelial and mesenchymal stromal cells (EC and MSC) during disease progression and following VRd therapy. The study reports early stress responses in MGUS EC, impaired differentiation and metabolic reprogramming in MSC, and a myeloma-specific interferon (IFN) signature across the BME that is reduced after treatment, suggesting that non-hematopoietic BME remodeling contributes to MM pathogenesis from premalignant stages onward.
However, the analysis is superficial due to the very limited number of cells analyzed. Although the authors pooled and sorted cells from multiple samples, only a few hundred cells per group were captured, substantially constraining the depth and reliability of the single-cell analysis. The downstream analyses—including DEG identification, pathway enrichment, and regulon activity—are consequently limited in interpretive power and robustness. The IFN signature has been reported to be widely present in myeloma cells as well as in the microenvironment in several prior publications; thus, the novelty is somewhat compromised. In addition, the immunofluorescence validation lacks quantitative assessment, making it unclear whether the presented examples are representative. In the human samples, the extremely low proportion of MSC further

raises doubts about the purity of the sorted populations and the reliability of the derived transcriptomic signals. Overall, while the study provides potentially interesting insights into non-hematopoietic BME remodeling in MM, the superficial analyses and limited data robustness considerably weaken the strength and interpretability of the conclusions.

Reviewer #3

(Remarks to the Author)
The study of Cenzano and Cocera et al takes advantage of recent advances in genetically-engineered murine models of myeloma to probe the interaction between malignant plasma cells and the non-immune component of the bone marrow microenvironment. The authors rightly address a gap in the MM field where almost all microenvironment studies only focus on immune components of the microenvironment, ignoring the non-immune components like endothelial cells and mesenchymal stromal cells. Here to profile these cells the authors largely deployed flow cytometry-based sorting followed by single cell sequencing and associated bioinformatics. There is immunohistochemistry level validation in some cases. The authors' major biological finding is an interferon-like signature in the MSCs of some of the analyzed mice that is recapitulated in a similar limited fraction of European-descent patients. The authors also show that this signature can be reversed by VRd treatment in mice.

Overall, this study appears to well executed from a technical perspective. While it is a largely descriptive study, it likely has relevance in the myeloma field in terms of elucidating potentially relevant biology not previously explored. However, the main critique is that based on the results of Fig. 7, it seems that a lot of the relevant biological pathways could also be identified in MSCs from humans. Therefore, this final figure begs the question: what is really the advantage of using the mouse model for this study? Why not just study the human and go from there? In general, the advantage of mouse modeling is the ability to selectively perturb the system to test specific hypotheses, which is often highly challenging if not impossible to do in humans, depending on the question. Here, some of this testing takes place with the one VRd treatment study. But in general everything else presented is a static picture. The authors in particular rely heavily on bioinformatic analysis of predicted transcriptional "regulons" as well as prediction of receptor-ligand interactions; however, these analyses can both readily have false negatives and false positives. Therefore, performing additional mechanistic studies testing at least one or two biological hypotheses extending from the descriptive studies here would provide significant value to the paper by more clearly demonstrating the utility of monitoring MSC dynamics in this murine model.

Major comments:
1. As noted above, the main gap in the manuscript is some additional mechanistic investigation of any of the findings in the paper, particularly around transcriptional regulation pathways or relevant cell-cell interactions between plasma cells and non-immune microenvironment cells. I don't want to be prescriptive into what exactly needs to be explored – I'll leave this up to the authors – but I see this as critical to showing the value of exploring these cellular phenotypes in the murine models. It would be great to validate one aspect of the regulon analysis and another from the cell-cell interaction predictions in particular, if possible.
2. What is the relevance of a "subset" of MM patients being defined like a more interferon-like response in the BME? Do they have different prognosis or response to therapy? Do they correspond to a specific genomic subtype (say high risk disease)? This would be very useful to comment on if any relevant clinical data is available. And, particularly if clinical data is available but this IFN-like signature does not carry any clinical prognostic relevance, why not?
3. Overall the immunohistochemistry images do not provide much information, at least in the way I can visualize it in the submitted manuscript. It would be very beneficial to have some quantification of co-localized staining to more clearly show differences in the IFN-high versus IFN-low conditions.

Minor comments:
1. I don't really understand this line (138-140): "Considering that BIcγ1 mice received T-cell-driven immunization, we included similarly immunized YFPcγ1 mice, carrying a yellow-fluorescence reporter activated from germinal-center B cells, as controls (Fig. 1A)." What exactly was immunized in these models?
2. Additional explanation of chosen flow markers for sorting would be useful. For example, what is VDO (line 141)?
3. The term "GRN inference" (line 163) should be more clearly explained in the main text, at least briefly, in addition to being explained in detail in the supplementary methods, given that a substantial part of the analysis relies on this method.

Reviewer #4

(Remarks to the Author)
The groups of Drs. Calvo, Gómez-Cabrero, and Prosper performed single-cell RNA sequencing on two recently described mouse models of myeloma to investigate the roles of mesenchymal stromal and endothelial cells in disease progression from precursor stages to myeloma. This is a highly relevant research question. However, I have several comments and concerns:
1. While the observed differences are interesting, the authors should better explain why these two models are suitable to study the transition from precursor stages to active myeloma. Even in the slow-progression model, the time difference between MGUS and MM appears to be only 4–6 months, whereas in human disease it often spans a decade or more. What criteria define multiple myeloma in these models (e.g., bone disease)? Does tumor load increase linearly over time, or is there a stable phase followed by rapid expansion? Furthermore, in human disease the transition is often driven by acquisition of driver mutations and emerging subclones. Do the authors observe anything similar in their models?
2. Can the authors exclude that the changes in gene signatures are a consequence rather than a cause of tumor expansion?

If not, I recommend avoiding conclusions that stage-specific BME–PC interactions drive disease progression.
3. Do the new findings correlate with tumor load? For example, do mice at the myeloma stage with lower plasma cell levels show the same changes as those with high tumor burden?
4. Please clarify how comparisons between stages were performed. If I understand correctly, the authors compared pooled samples from multiple mice without hashing or barcoding. In that case, cell-level comparisons may not be appropriate for drawing conclusions at the mouse level, even if "cells within the MM2 cluster originated from both male and female mice." Separately analyzed biological replicates are essential.
5. The differences between the two models should be more clearly described and discussed. In several parts, it is unclear whether results are consistent across both models or if significant differences exist. For instance, how do the authors explain that the interferon-associated MM signature was detected in ECs and MSCs from the BIcγ1 model but absent in the more aggressive MIcγ1 model?
6. The IF stainings shown in Figures 6 and 7 are difficult to interpret. There appears to be considerable background signal, and it is not clear whether the arrows indeed point to IFN-response–positive ECs or MSCs.
7. The authors should be more specific about the human data. How many patients show enrichment of the murine MM–IFN-related genes in ECs and MSCs, and how is "enrichment" defined? Do all of these patients exhibit a "shared immunomodulatory response of the BME"?
8. Please describe in detail how murine bone marrow non-hematopoietic cells were isolated. Is the sampling procedure comparable to that used for human bone marrow aspirates? How were samples from orthopedic surgery donors processed? Differences in sample type and processing could significantly affect EC and MSC composition and thus represent a potential confounder.

Version 1:

Reviewer comments:

Reviewer #1

(Remarks to the Author)
The authors have addressed all my comments. Nice work!

(Remarks on code availability)

Reviewer #2

(Remarks to the Author)
My initial concern was the limited number of cells analyzed. The authors have addressed this issue by applying stringent parameters in the data analysis and performing orthogonal validations across independent cohorts and platforms to ensure the robustness of their findings. While non-hematopoietic cells are difficult to capture in single-cell studies, which substantially limits their investigation, the current study still provides meaningful insights into these cell populations and offers valuable clues regarding their potential roles and interactions within the tumor microenvironment.

(Remarks on code availability)

Reviewer #3

(Remarks to the Author)
I appreciate the authors' efforts in addressing my comments on the manuscript. I do not have any further comments and now find the manuscript acceptable for publication.

(Remarks on code availability)

Reviewer #4

(Remarks to the Author)
I have no more comments.

(Remarks on code availability)

Open Access This Peer Review File is licensed under a Creative Commons Attribution 4.0 International License, which permits use, sharing, adaptation, distribution and reproduction in any medium or format, as long as you give appropriate credit to the original author(s) and the source, provide a link to the Creative Commons license, and indicate if changes were made.
In cases where reviewers are anonymous, credit should be given to 'Anonymous Referee' and the source.
The images or other third party material in this Peer Review File are included in the article's Creative Commons license, unless indicated otherwise in a credit line to the material. If material is not included in the article's Creative Commons license and your intended use is not permitted by statutory regulation or exceeds the permitted use, you will need to obtain permission directly from the copyright holder.
To view a copy of this license, visit https://creativecommons.org/licenses/by/4.0/

Answers to Reviewers

We **sincerely appreciate the thorough review** of our manuscript (NCOMMS-25-53828) and the valuable feedback provided **by both the reviewers and the editorial team**.

In the revised version, guided by the reviewers' comments, we have thoroughly addressed the reviewers' concerns and incorporated their suggestions through additional immunofluorescence validation quantitative assessment, new functional validation experiments and the edition of the manuscript accordingly.

Below, we provide a detailed, point-by-point response to the reviewer's comments. Revisions are highlighted in the manuscript in **red color**, and reviewer comments are reproduced below in **black color**, followed by our responses in **blue color**. We hope the revised manuscript meets with your approval and thank you for your consideration.

In summary, we believe **these revisions have substantially strengthened the manuscript**, and we sincerely **recognize the reviewers'** insights that guided these improvements.

REVIEWER COMMENTS

Reviewer #1 (Remarks to the Author):

The work of Cenzano et al. shows that non-immune cells in the bone marrow microenvironment, particularly endothelial cells and mesenchymal stem cells, undergo changes during the transition from MGUS to active myeloma and may be partly responsible for the progression of the disease. Interestingly, an IFN signature in the above-mentioned cells appears to be significant for the progression of the disease.

The findings are new and could be of clinical significance, as the authors present convincing mouse models as well as human data. It should be emphasised that, through their collaboration with Benjamin Ebert, the authors are able to show in an elegant model that lenalidomide therapy can reverse these changes. This work provides the basis for further investigation of the IFN signalling pathway and its significance for MM progression.

We **appreciate the reviewer's positive feedback** on our work.

I only have minor comments:

1. Please check the references: References 12 and 48 are identical.

Thank you for pointing this out. **We have edited in the revised manuscript**.

2. The images are very small. This makes the immunofluorescence images particularly difficult to interpret.

We appreciate the reviewer for bringing this point to our attention. **We have modified accordingly in the revised manuscript** (Figure 2, 6, 7 and Supplementary Figure 4, and 13).

3. A quantification of ISG15 and GBP2 would be useful here.

We thank the reviewer for this valuable suggestion. **We have quantified** the previously performed IF experiments and, where possible, increased the number of samples (control mice, MM BI$c_{\gamma 1}$ and MM MI$c_{\gamma 1}$, as well as healthy human samples). As we expected, these quantifications confirmed our previous IF imaging results.

The resulting data have been incorporated into the IF figures (see Figures 2I and J; 6B; 7G and I; and Supplementary Figure 5B and C).

FIGURE 2

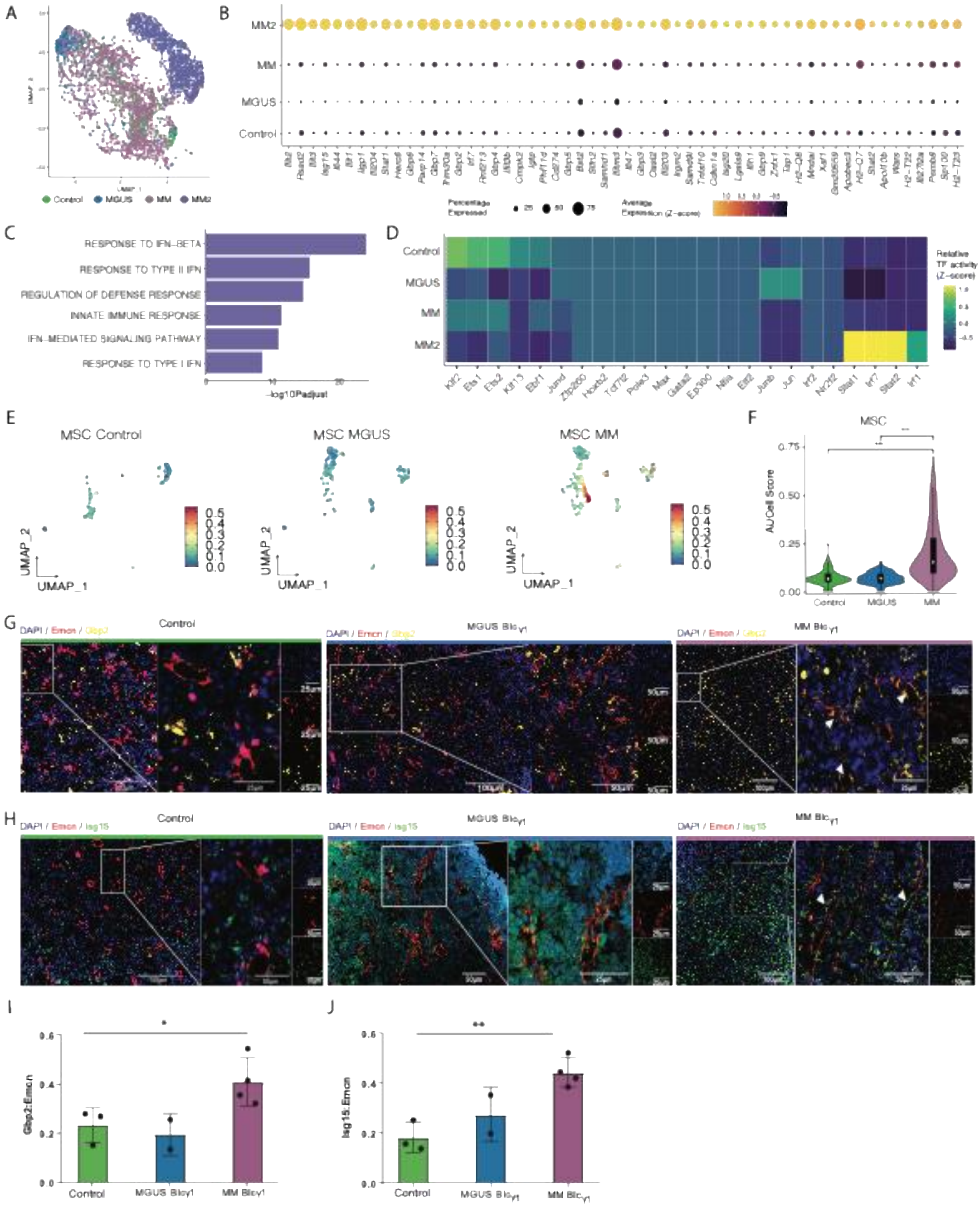

Fig. 2. Identification of a MM-specific IFN signature in the BME.
(A) UMAP dimension reduction of EC revealing a transcriptionally distinct population in MM. (B) Dot plot visualization of the markers defining EC MM2 cluster. Average gene expression is shown by color intensity, and the percentage of cells expressing each gene is shown by size. (C) Bar charts of enriched GO terms from ORA (adjusted p. value) computed using MM2 cluster markers. The horizontal axis represents the negative of the base 10 logarithm of the adjusted p. value. (D) Heatmap representing z-scaled mean regulon activity score of inferred top specific regulons for each stage. (E) UMAP plot illustrating the distribution of the IFN-related signature in MSC across control, MGUS, and MM. (F) Violin plots showing AUC-score for IFN-

related signature within MSC in control, MGUS and MM cells. (G-H) Immunofluorescence (IF) staining of EC (Emcn, red), IFN response genes markers of the MM2 cluster (Gbp2, yellow (G); Isg15, green (H)), and nucleus (DAPI) (blue) in Formalin-Fixed Paraffin-Embedded (FFPE) femurs from Control (YFP$_{c\gamma1}$), MGUS BI$_{c\gamma1}$ and MM BI$_{c\gamma1}$. White arrows point EC Ecmn^{+} IFN^{+} (Gbp2 (G)/Isg15 (H)) in MM BI$_{c\gamma1}$. (I-J) Quantification of the percentage of endothelial IFN-enriched cells, based on the coexpression of Emcn with IFN+ cells (Gbp2, (I); Isg15, (J)) in femurs from Control (YFP$_{c\gamma1}$), MGUS BI$_{c\gamma1}$ and MM BI$_{c\gamma1}$ mice.

FIGURE 6

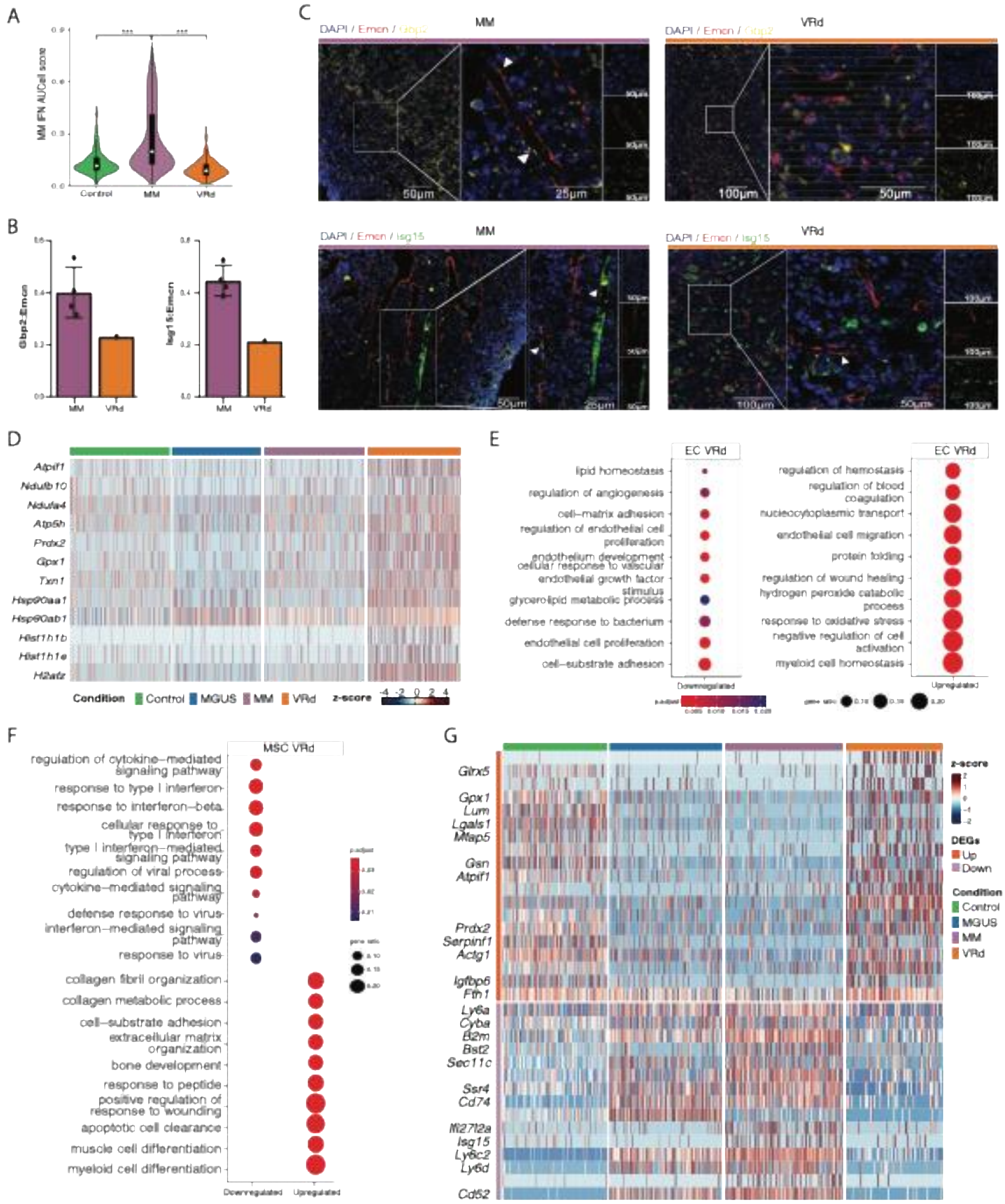

Fig. 6. VRd induces transcriptional remodeling of EC and MSC in MM. (A) Violin plot showing AUC-score for IFN-related signature in EC from Control (YFP$_{c\gamma1}$), MM BIc$_{\gamma1}$ (MM) and MM BI$_{c\gamma1}$-*Crbn*^{I391V} VRd-treated (VRd) mice. (B) Quantification of the percentage of endothelial IFN^{+} cells, determined by the coexpression of Emcn with IFN response genes (Gbp2 (left plot) and Isg15 (right plot) in femurs from MM BIc$_{\gamma1}$ (MM) and MM BI$_{c\gamma1}$-*Crbn*^{I391V} VRd-treated (VRd) mice. (C) IF staining of EC (Emcn, red), IFN

response genes (Gbp2 (yellow, (upper plot)); Isg15 (green, (bottom plot)), and nucleus (DAPI) (blue) in FFPE femurs from MM $BI_{c\gamma1}$ mice (MM) and VRd-treated mice (VRd). White arrows point EC ($Ecmn^{+}$) IFN^{+} (Gbp2/Isg15). (D) Heatmap showing the expression of stress-related DEGs identified in the comparison between treated and non-treated non-IFN MM EC at different stages of the disease. Color represents the z-score scaled expression values. (E) Significant terms derived from ORA between VRd-treated and untreated non-IFN MM EC. (F) Significant terms derived from ORA of MSC from MM mice before and after VRd treatment. (G) Heatmap projecting the expression of DEGs identified in the comparison between non-treated and treated MSC at different stages of the disease. Color represents the z-score scaled expression values.

FIGURE 7

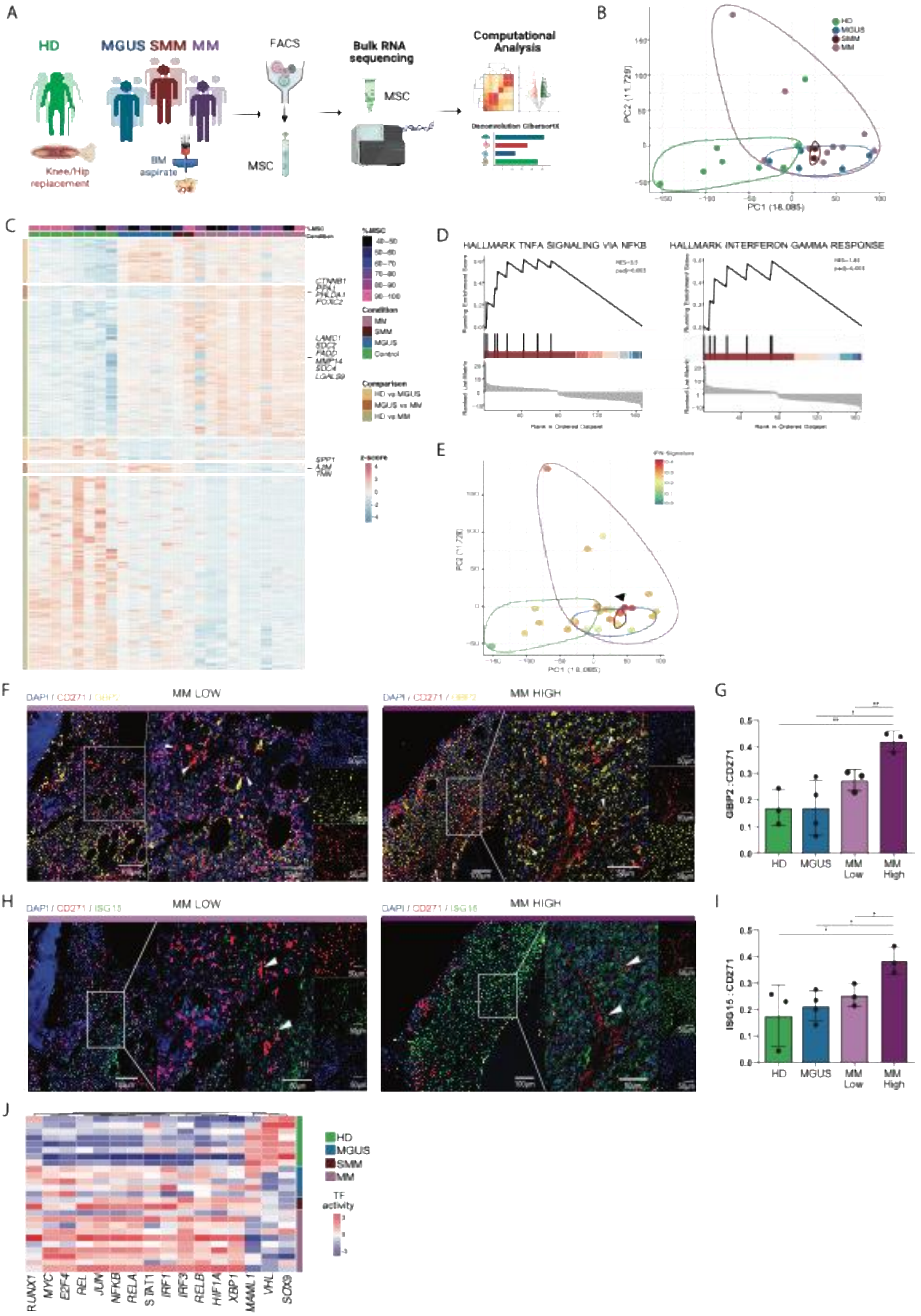
A
HD
MGUS SMM MM
Knee/Hip replacement
BM aspirate
FACS
MSC
Bulk RNA sequencing
MSC
Computational Analysis
B
PC1 (18.085)
C
D
HALLMARK TNFA SIGNALING VIA NFKB
HALLMARK INTERFERON GAMMA RESPONSE
E
F
MM LOW
MM HIGH
G
HD
MGUS
MM Low
MM High
H
MM LOW
MM HIGH
I
HD
MGUS
MM Low
MM High
J
HD
MGUS
SMM
MM
TF activity
RUNX1
MYC
E2F4
REL
JUN
NFKB
RELA
STAT1
IRF1
IRF3
RELB
HIF1A
XBP1
MAML1
VHL
SOX9

Fig. 7. Transcriptional dynamics of human MSC during MM progression. (A) Experimental workflow used for the isolation and bulk RNA-seq analysis of human BM MSC. (B) PCA of MSC expression profiles shows clustering by disease stage. The color of the dots represents disease stages. (C) Heatmap showing the expression profiles of DEGs identified in each pairwise comparison across disease progression. (D) GSEA plots for the gene sets 'TNFα signaling via NFκB' (left panel) and 'Interferon gamma response' (right panel) in the comparison between healthy donors (HD)- MSC and MM-MSC. (E) PCA plot showing the MM-IFN signature score in each human sample, previously identified murine BM MM cells. (F) IF staining of MSC (CD271, red), IFN response genes (*GBP2*, yellow), and nucleus (DAPI) (blue) in archived paraffin-embedded human BM biopsies of MM patients with low (MM LOW) and high IFN signal (MM HIGH) in stromal cells (see Supplementary Table 2). White arrows point to MSC ($CD271^+$) in MM LOW IFN samples, and MSC ($CD271^+$) IFN^+ (GBP2) in MM HIGH IFN samples. (G) Quantification of the percentage of mesenchymal IFN^+ cells, determined by the coexpression of $CD271^+$ with IFN response genes (*GBP2*) in FFPE biopsies from HD (three donors) and archived paraffined human BM biopsies of MGUS (four patients) and MM patients (six patients with low (three) and high (three) IFN signal in stromal cells). (H) IF staining of MSC (CD271, red), IFN response genes (*ISG15*, green), and nucleus (DAPI) (blue) in archived paraffin-embedded human BM biopsies of MM patients with low (MM LOW) and high IFN signal (MM HIGH) in stromal cells (see Supplementary Table 2). White arrows point to MSC ($CD271^+$) in MM LOW IFN samples, and MSC ($CD271^+$) IFN^+ (ISG15) in MM HIGH IFN samples. (I) Quantification of the percentage of mesenchymal IFN^+ cells, determined by the coexpression of $CD271^+$ with IFN response genes (*ISG15*) in FFPE biopsies from HD (three donors) and archived paraffined human BM biopsies of MGUS (four patients) and MM patients (six patients with low (three) and high (three) IFN signal in stromal cells). (J) Heatmap representing the variation of TF activity score across samples.

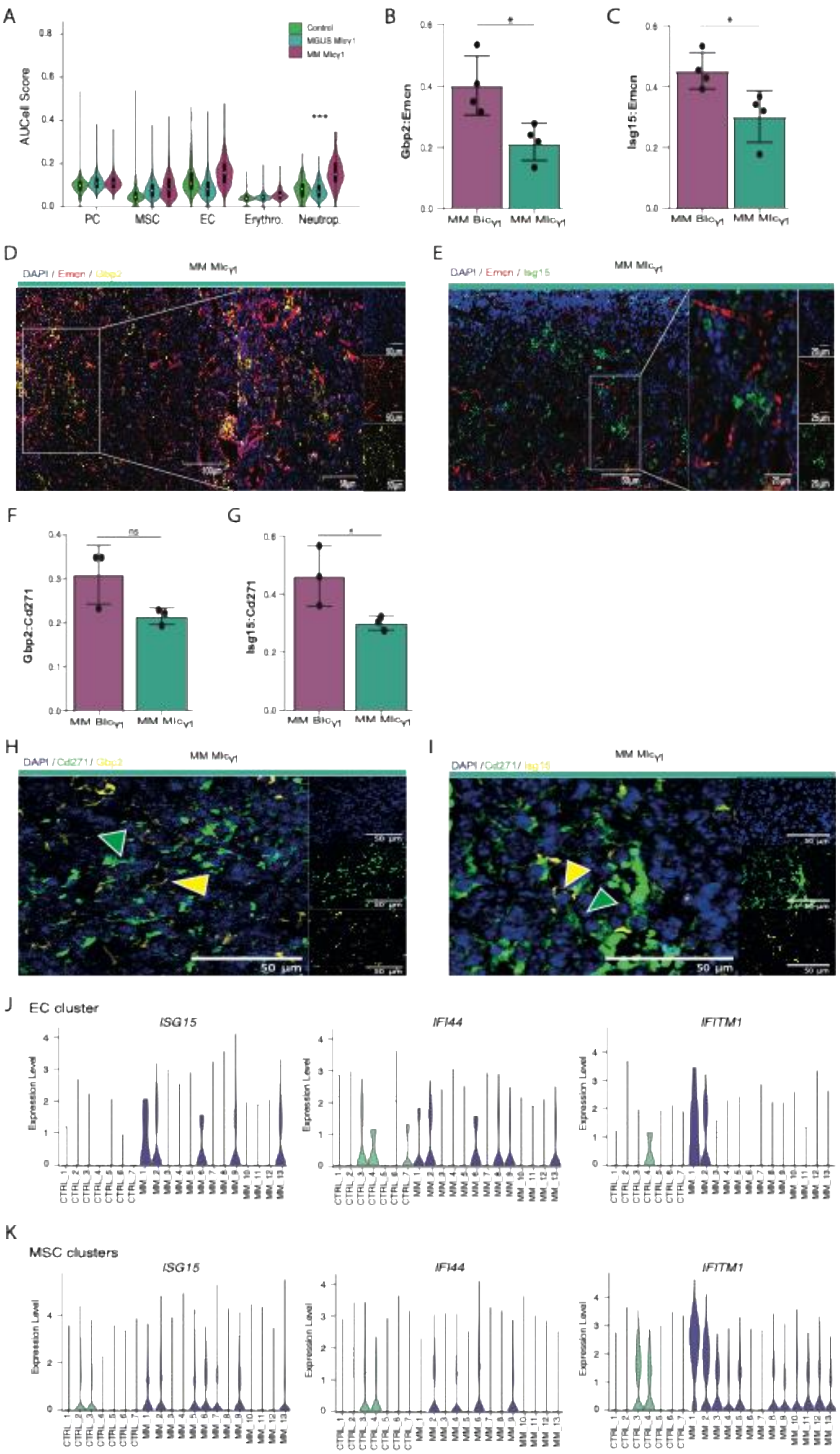
A
AUCell Score
Control
MGUS Mlcγ1
MM Mlcγ1
***
PC
MSC
EC
Erythro.
Neutrop.
B
Gbp2:Emcn
*
MM Blcγ1
MM Mlcγ1
C
Isg15:Emcn
*
MM Blcγ1
MM Mlcγ1
D
DAPI / Emcn / Gbp2
MM Mlcγ1
E
DAPI / Emcn / Isg15
MM Mlcγ1
F
Gbp2:Cd271
ns
MM Blcγ1
MM Mlcγ1
G
Isg15:Cd271
*
MM Blcγ1
MM Mlcγ1
H
DAPI / Cd271 / Gbp2
MM Mlcγ1
50 µm
I
DAPI / Cd271 / Isg15
MM Mlcγ1
50 µm
J
EC cluster
ISG15
IFI44
IFITM1
Expression Level
K
MSC clusters
ISG15
IFI44
IFITM1
Expression Level

Supplemental Figure 5: Validation in the $MI_{c\gamma1}$ model and scRNA-seq human data. (A) Violin plots showing AUC-score for MM IFN-related signature within BM populations in the $MI_{c\gamma1}$ model. (B-C) Quantification of the percentage of endothelial IFN-enriched cells, based on the coexpression of Emcn with IFN+ cells (Gbp2, (B); Isg15, (C)) in femurs from MM $BI_{c\gamma1}$ and MM $MI_{c\gamma1}$ mice. (D-E) IF staining of EC (Emcn, red), and IFN response genes markers (Gbp2 (yellow, (D)); Isg15 (green, (E))), and nucleus (DAPI) (blue) in FFPE femurs from MM $MI_{c\gamma1}$ mice. (F-G) Quantification of the percentage of mesenchymal stromal cells IFN-enriched cells, based on the coexpression of $Cd271^{+}$ with IFN+ cells (Gbp2, (F); Isg15, (G)) in femurs from MM $BI_{c\gamma1}$ and MM $MI_{c\gamma1}$ mice. (H-I) IF staining of MSC (Cd271, green), and IFN response genes markers (Gbp2 (yellow, (H)); Isg15 (yellow, (I))), and nucleus (DAPI) (blue) in FFPE femurs from MM $MI_{c\gamma1}$ mice. Green arrows point to MSC ($Cd271^{+}$), and yellow arrows point to IFN^{+} (Gbp2/Isg15). (J) Violin plots comparing the distribution of the gene expression of *ISG15*, *IFI44*, and *IFITM1* in EC from MM patients and healthy controls from scRNA-seq published data[1]. (K) Similar to J for MSC from the same healthy controls and MM human patients from scRNA-seq published data [1].

Moreover, **to further confirm** our interesting **IFN results in MM-MSC** by scRNA-seq, we performed additional IF staining for IFN-signature (Isg15 and Gbp2) in MSC ($Cd271^{+}$ cells) from Ctrl, MGUS $BIc_{\gamma1}$, MM $BIc_{\gamma1}$, MM $MIc_{\gamma1}$, and MM $BI_{c\gamma1}$-$Crbn^{I391V}$ VRd-treated (VRd) mice and quantified the staining across all groups. These new IF results recapitulate the pattern previously observed in EC. Specifically, an increase of IFN signaling in MM $BIc_{\gamma1}$ cells in comparison to control, MGUS and MM $MIc_{\gamma1}$, as well as a reduction in MM $BI_{c\gamma1}$-$Crbn^{I391V}$ VRd-treated (VRd) mice.

The corresponding results have been incorporated into the Results section (lines 184-185: *We confirm these scRNA-seq results by IF staining of EC and MSC IFN^{+} in BM biopsies from MM $MI_{c\gamma1}$ mice (**Supplementary Fig. 5B-I**)*, and Supplementary Figures 4F–I, and new Supplementary 5 B-I (see previously), and 13I and K.

The experimental details have been added to the Methods section (lines 206-208: *Antigen retrieval was carried out at 95°C for 30 minutes in either 10 mM citrate buffer (pH 6.0) or, specifically for CD271 staining combinations, in 10 mM Tris-1 mM EDTA buffer (pH 9.0)).*

Lines 209-212:

For immunofluorescence (IF) staining, the Formalin-Fixed Paraffin-Embedded (FFPE) sections were blocked with BSA 5% for 30 min at RT. The following primary antibodies were used: rat anti-Endomucin antibody (V.7C7) (Santa Cruz Technology 1: 50 dilution), mouse anti-CD271 monoclonal antibody (NGF Receptor) (ME20.4) (Invitrogen, 1: 50 dilution) …

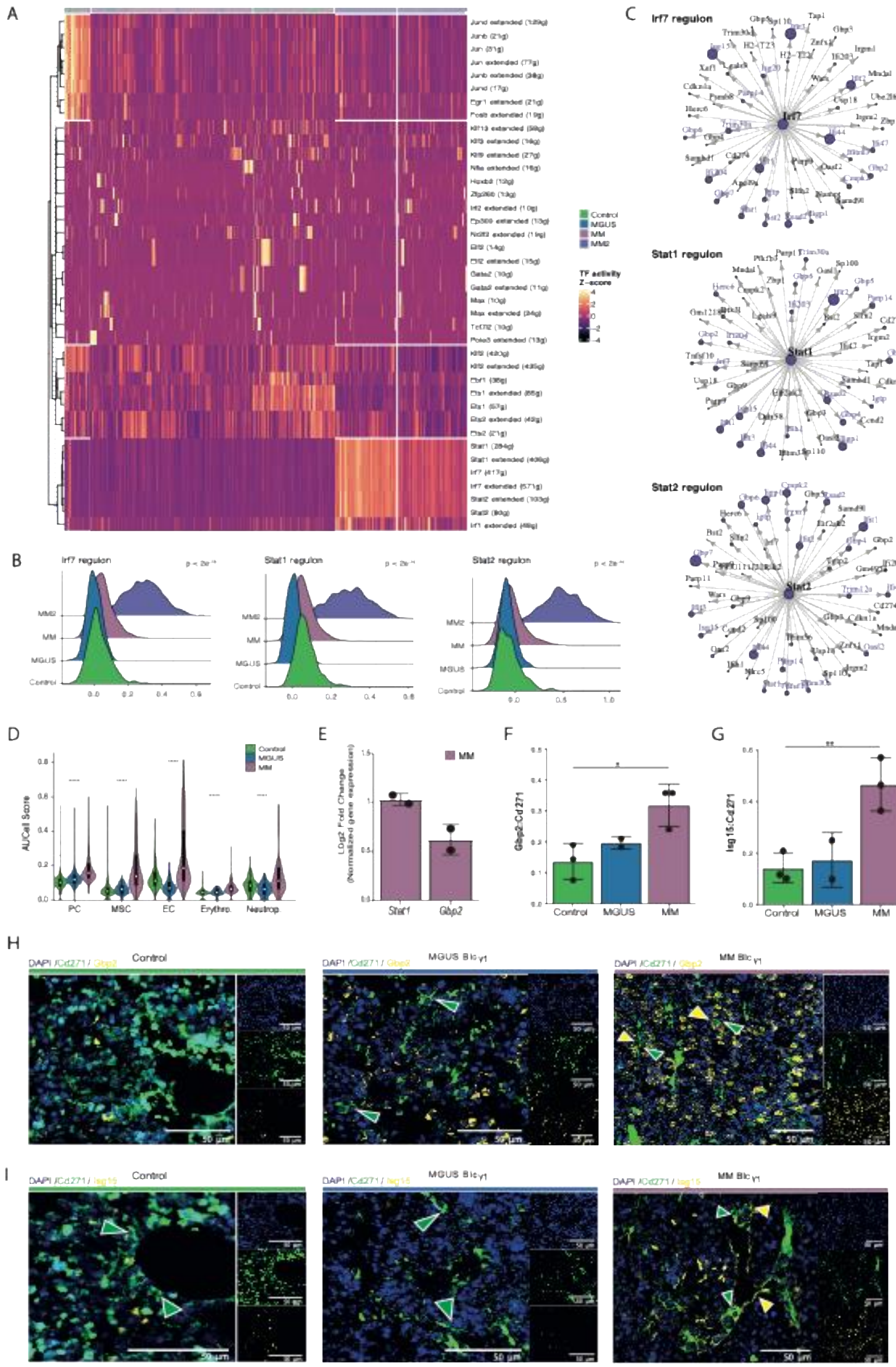
A
C
Irf7 regulon
Stat1 regulon
Stat2 regulon
Control
MGUS
MM
MM2
TF activity Z-score
B
Irf7 regulon
Stat1 regulon
Stat2 regulon
D
AUCell Score
PC
MSC
EC
Erythro.
Neutrop.
E
Log2 Fold Change (Normalized gene expression)
Stat1
Gbp2
F
Gbp2:Cd271
G
Isg15:Cd271
H
DAPI /Cd271 / Gbp2
Control
MGUS Blc γ1
MM Blc γ1
50 µm
I
DAPI /Cd271 / Isg15
Control
MGUS Blc γ1
MM Blc γ1
50 µm

Supplemental Figure 4: Additional information on the MM IFN-related signature in the $BI_{c\gamma1}$ model. (A) Heatmap showing the activities of the enriched regulons in EC (Z-score of regulon activity). (B) Density plots showing the score of Irf7, Stat1, and Stat2 regulons in EC (p. = adjusted p-value). (C) Regulon network of Irf7, Stat1, and Stat2 representing the top 50 target genes. (D) Violin plots showing AUC-score for MM IFN-related signature within BM populations in the $BI_{c\gamma1}$ model. (E) Quantitative PCR (qPCR) analysis of the IFN regulon (*Stat1)* and the associated gene (*Gbp2)* in MSC derived from mesenspheres of MM BIcγ1 mice. Gene expression is presented as log_2 fold change relative to MSC derived from mesenspheres of control mice. (F-G) Quantification of the percentage of mesenchymal stromal cells IFN-enriched cells, based on the coexpression of $Cd271^+$ with IFN+ cells (Gbp2, (F); Isg15, (G)) in femurs from Control, MGUS $BI_{c\gamma1}$ and MM $BI_{c\gamma1}$ mice. (H-I) IF staining of MSC (Cd271, green), and IFN response genes markers (Gbp2 (yellow, (H)); Isg15 (yellow, (I))), and nucleus (DAPI) (blue) in FFPE femurs from Control, MGUS $BI_{c\gamma1}$, and MM $BI_{c\gamma1}$ mice. Green arrows point to MSC ($Cd271^+$), and yellow arrows point to IFN^+ (Gbp2/Isg15).

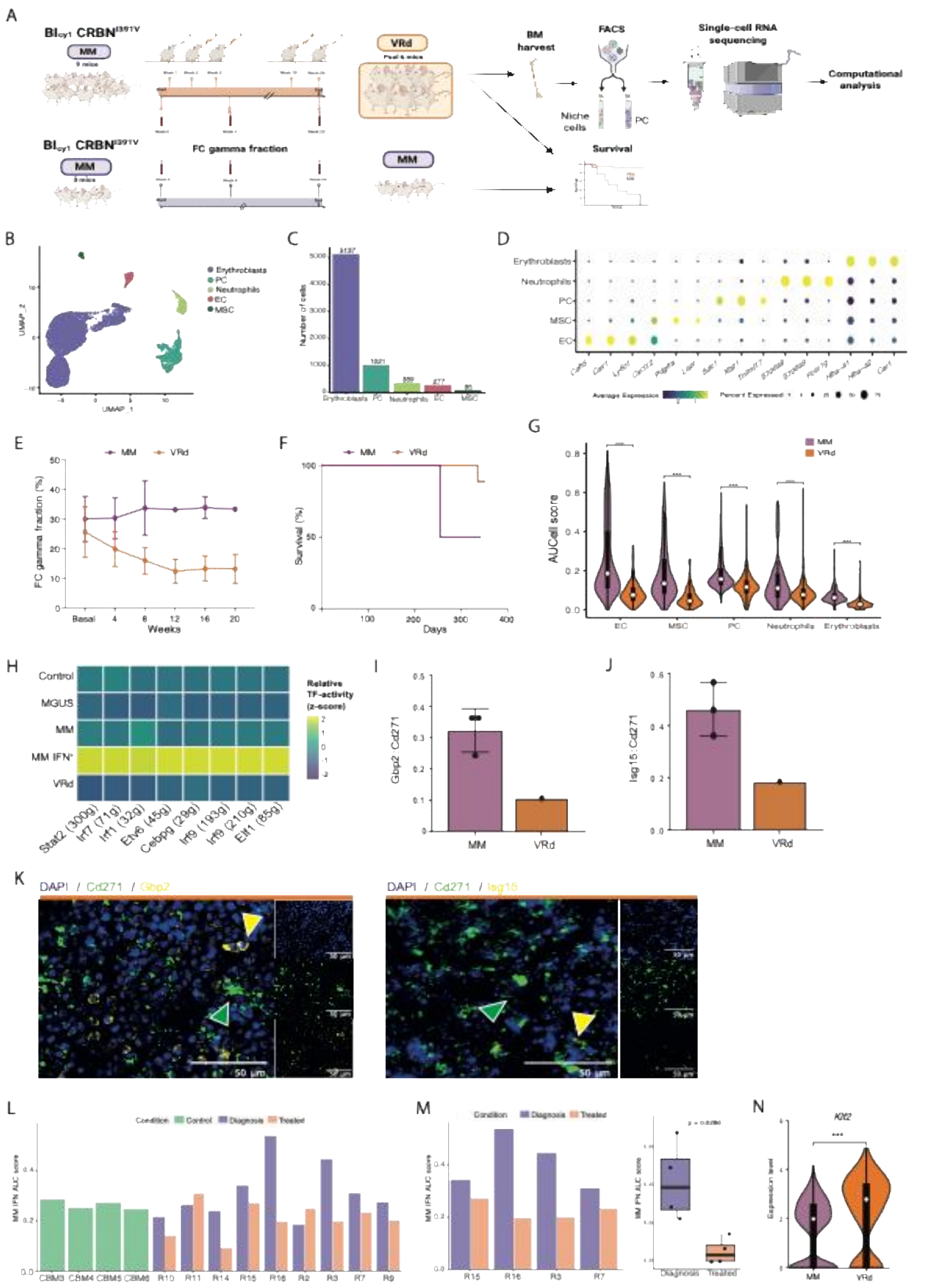

Supplemental Figure 13: Additional analysis of VRd-treated mice and transcriptional alterations in EC and MSC from human patients. (A) VRd experiment overview representing treatment administration, serum collection for FC gamma fraction, and scRNA-seq isolation of PC, MSC, and EC from MM BI$_{c\gamma1}$ *Crbn*^{I391V} (pool of six mice). MM BI$_{c\gamma1}$ *Crbn*^{I391V} were used as controls for FC gamma fraction and

survival. (B) UMAP representation of BM microenvironment cells from $BI_{c\gamma1}$-*Crbn*^{I391V} VRd-treated mice, colored by cell clustering. (C) Bar plots showing the number of cells per BM population. (D) Dot plot of canonical markers used to define PC, EC, MSC, erythroblasts, and neutrophils. The dot size reflects the percentage of cells within the cluster expressing each gene, and the color represents the average expression level. (E) Representation of the percentage of serum FC gamma fraction in VRd-treated versus the MM group. (F) Kaplan–Meier survival curves of treated and non-treated MM $BI_{c\gamma1}$ mice. (G) Violin plots showing AUC-score for MM IFN-related signature within BM populations in VRd-treated and MM $BI_{c\gamma1}$ mice. (H) Heatmap representing z-scaled mean regulon activity score of IFN-related regulons in EC across each condition. (I-J) Quantification of the percentage of mesenchymal IFN^+ cells, determined by the coexpression of Cd271 with IFN response genes (Gbp2 (I) and Isg15 (J) in femurs from MM $BIc_{\gamma1}$ (MM) and MM $BI_{c\gamma1}$-*Crbn*^{I391V} VRd-treated (VRd) mice. (K) IF staining of MSC (Cd271, green), IFN response (Gbp2, yellow (left plot) and Isg15, yellow (right plot)), and nucleus (DAPI) (blue) in FFPE femurs from VRd-treated mice. Green arrows point to MSC ($Cd271^+$), and yellow arrows point to IFN^+ (Gbp2/Isg15). (L) Bar plots showing the AUC-score for MM IFN-related signature in MSC from healthy controls and MM human patients before and post-induction treatment from bulk RNA-seq published data[1] . (M) Bar plots showing the AUC-score for MM IFN-related in MSC from the subset of high IFN MM human patients before and after induction treatment[1] by individuals (left panel) and grouped by disease (right panel). (N) Violin plot displaying the upregulation of *Klf2* in VRd-treated EC vs non-treated MM EC.

4. There is also a lack of information about these proteins. They are used as markers, but are hardly discussed. ISG15 in particular, which is also important for a Th1 response, should be discussed.

We thank the reviewer for this insightful comment. We have expanded the discussion in the revised manuscript (lines 411-418).

Considering the reported role of Isg15 in modulating Il-12 release and Th1 differentiation, IFN-responsive stromal cells may influence local Il-12 availability and Th1 polarization[63], thereby contributing to immune modulation within the non-immune microenvironment of MM. In addition, our observation of C3-mediated interactions between PC and EC or MSC further supports the presence of multiple immunoregulatory pathways operating within non-immune MM. Together, the MM IFN-inducible response uncovered in our study, supports an immunomodulatory role of the non-hematopoietic BME, with EC and MSC potentially contributing to immune remodeling and T-cell exhaustion under inflammatory conditions[20].

5. I am having difficulty understanding the meaning of Figures 5A and 5D. It would be helpful if you could explain them in one or two sentences.

We thank the reviewer for highlighting the need for clearer interpretation of Figures 5A and 5D.

Figure 5A summarizes the total number and directionality of predicted ligand–receptor (L-R) interactions between PC and niche populations (EC and MSC) across disease stages. The heatmap shows a reduction of EC-derived outgoing signals at MGUS and a shift toward increased PC-driven interactions in MM, indicating a progressive rewiring of communication dynamics.

Figure 5D highlights L-R pairs that are shared between PC-EC and PC-MSC communication within each stage, illustrating stage-specific interactions that are common to both niche populations. PC-derived ligand *Vegfa* engage multiple receptors across both EC and MSC in MGUS and MM, whereas shared *C3*-mediated interactions between PC and both niche populations emerge specifically in MM.

To improve clarity and facilitate interpretation, we removed the marginal bar plots from Figure 5A to reduce visual complexity, and we have simplified Figure 5D by removing secondary interactions (*Itgb5*-mediated interactions) that were not central to the main biological message, and we have updated the legend accordingly.

We have now added a concise explanatory description in the main text and revised the figure legends (see below) to guide interpretation of these panels. *Lines 265-268: … We uncovered L-R pairs shared between EC-PC and MSC-PC communication across disease stages (Fig. 5D). Consistent with previous findings in human MM patients, malignant PC engaged multiple receptors in EC and MSC through Vegfa secretion, supporting a pro angiogenic signaling in MM. Additionally, shared C3-mediated interactions between PC and the non-hematopoietic BME emerged at MM stage, warranting further …*

Moreover, we validated some of the previously identified interactions in MGUS-BIcγ1 mice and MM BIcγ1 FFPE biopsies by IF. Specifically, we focused on the interactions between the PC-derived ligands *Vegfa* and *C3* and their respective receptors (*Itgb1* and *Lrp1*), expressed by EC and MSC.
As expected, these new IF results further validate the interactions predicted by our scRNA-seq analysis These results are summarized in new Figure 5F, G (see below).
The corresponding results have been incorporated into the Results section (lines 270-275: *To further confirm the interactions identified in MGUS and MM BIcγ1 mice, we performed IF analysis on BM FFPE biopsies* ***(Supplementary Fig. 11E and Fig. 5F, G)****. We observed that the PC-derived ligand Vegfa and the EC/MSC receptor Itgb1 are expressed in close spatial proximity, suggesting functional crosstalk between these cell populations. In contrast, the spatial co-localization of the PC-derived ligand C3 and its corresponding MSC receptor Lrp1 was detected exclusively at the MM stage, thereby validating our previous findings from the scRNA-seq analysis.*
The experimental details have been added to the Methods section:
Lines 206-208:
Antigen retrieval was carried out at 95°C for 30 minutes in either 10 mM citrate buffer (pH 6.0) or, specifically for CD271 staining combinations, in 10 mM Tris-1 mM EDTA buffer (pH 9.0).
Lines 209-212:
For immunofluorescence (IF) staining, the Formalin-Fixed Paraffin-Embedded (FFPE) sections were blocked with BSA 5% for 30 min at RT. The following primary antibodies were used: rat anti-Endomucin antibody (V.7C7) (Santa Cruz Technology 1: 50 dilution), mouse anti-CD271 monoclonal antibody (NGF Receptor) (ME20.4) (Invitrogen, 1: 50 dilution) …
Lines 214-218:
VEGF Monoclonal Antibody (JH121) (Invitrogen, 1: 20 dilution), Integrin Beta 1/CD29 Polyclonal antibody (Proteintech, 1: 200 dilution), Complement C3 Polyclonal Antibody (Invitrogen, 1: 100 dilution), Anti-LRP1 antibody (abcam, 1: 50 dilution), CD304 (Neuropilin-1) Recombinant Rabbit Monoclonal Antibody (ST05-30) (Invitrogen, 1: 50 dilution) and Goat anti-GFP (Novus Biologicals, 1:4000).
Lines 222-228:
Goat anti-Rat IgG (H+L) Cross-Adsorbed Secondary Antibody, Alexa Fluor™ 568; Goat anti-Mouse IgG (H+L) Highly Cross-Adsorbed Secondary Antibody, Alexa Fluor™ Plus 488; Goat anti-Rabbit IgG (H+L) Cross-Adsorbed Secondary Antibody, Alexa Fluor™ 488, Goat anti-Mouse IgG (H+L) Cross-Adsorbed Secondary Antibody, Alexa Fluor™ 568 (Invitrogen), Donkey anti-Mouse IgG (H+L) Highly Cross-Adsorbed Secondary Antibody, Alexa Fluor™ 555 (Invitrogen), Donkey anti-Goat IgG (H+L) Cross-Adsorbed Secondary Antibody, Alexa Fluor™ 488 (Invitrogen).

FIGURE 5

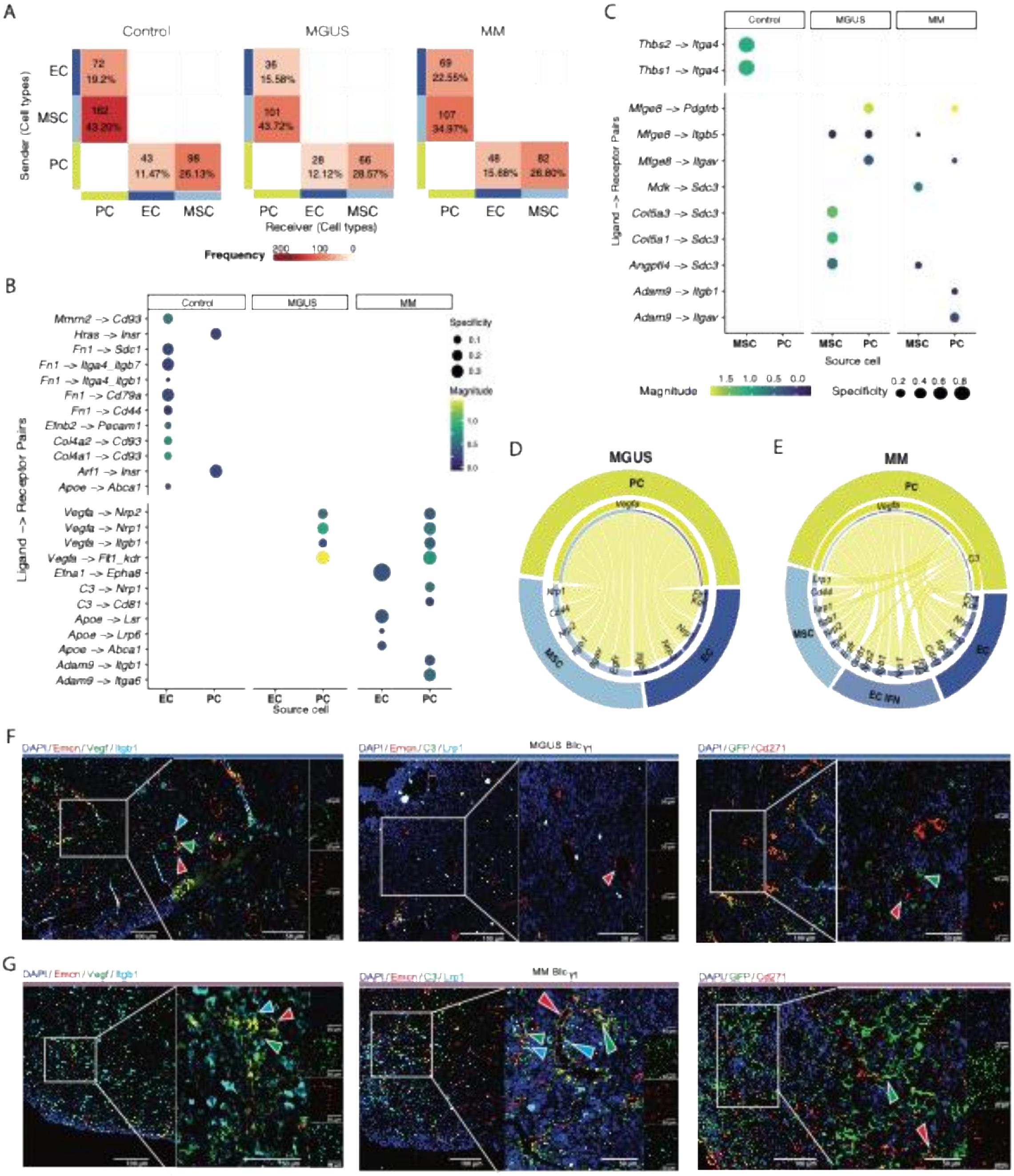

Fig. 5. Altered crosstalk between EC and MSC with malignant PC is potentially associated with myeloma development (A) Heatmap showing the total number and directionality of predicted L–R interactions between PC and niche populations (EC and MSC) across disease stages. Rows represent sender cell types and columns represent receiver cell types. Colors are proportional to the number of ligand-receptor pairs. (B-C) Dot plots of representative ligand-receptor interactions between PC and EC (B) or MSC (C) during disease progression. Dot size and color indicate the interaction's specificity and strength, respectively. (D-E) Chord diagrams illustrating L-R pairs shared between PC–EC and PC–MSC communication within MGUS (D) and MM (E) stages. Colors and width represent signal senders and strength, respectively. PC-derived *Vegfa* engages multiple receptors in both stages (D-E), while *C3*-mediated interactions emerge in MM (E). (F-G) IF staining of PC–EC/MSC interactions: PC-derived ligands Vegf (left panel, green) and C3 (middle panel, green) are shown in PC (GFP^{+}, green, right panel). The corresponding receptors Itgb1 (blue, left panel) and Lrp1 (blue, middle panel) are detected in EC (Emcn^{+}, red, left and middle panels) and MSC (Cd271^{+}, red, right panel). Stainings were performed on FFPE femur sections from MGUS BIcγ1 (F) and MM BIcγ1 mice (G).

Reviewer #2 (Remarks to the Author):

This study investigates the role of the non-hematopoietic bone marrow microenvironment (BME) in multiple myeloma (MM) progression and treatment response. Using BIcγ1 and MIcγ1 murine models that recapitulate MGUS-to-MM evolution, complemented by validation in patient-derived samples, the authors profile transcriptional changes in endothelial and mesenchymal stromal cells (EC and MSC) during disease progression and following VRd therapy. The study reports early stress responses in MGUS EC, impaired differentiation and metabolic reprogramming in MSC, and a myeloma-specific interferon (IFN) signature across the BME that is reduced after treatment, suggesting that non-hematopoietic BME remodeling contributes to MM pathogenesis from premalignant stages onward. However, the analysis is superficial due to the very limited number of cells analyzed. Although the authors pooled and sorted cells from multiple samples, only a few hundred cells per group were captured, substantially constraining the depth and reliability of the single-cell analysis. The downstream analyses—including DEG identification, pathway enrichment, and regulon activity—are consequently limited in interpretive power and robustness. The IFN signature has been reported to be widely present in myeloma cells as well as in the microenvironment in several prior publications; thus, the novelty is somewhat compromised. In addition, the immunofluorescence validation lacks quantitative assessment, making it unclear whether the presented examples are representative. In the human samples, the extremely low proportion of MSC further raises doubts about the purity of the sorted populations and the reliability of the derived transcriptomic signals. Overall, while the study provides potentially interesting insights into non-hematopoietic BME remodeling in MM, the superficial analyses and limited data robustness considerably weaken the strength and interpretability of the conclusions.

We thank the reviewer for the thoughtful and detailed evaluation of our work and for recognizing the potential relevance of investigating non-hematopoietic BME remodeling during MM progression and treatment response. In the following subsections, we address each comment in detail and describe the corresponding revisions and additional analyses performed to resolve them.

(1) Limited cell numbers and robustness

We appreciate the reviewer's concern regarding the limited number of EC and MSC captured by scRNA-seq. We would like to emphasize that we initially shared this concern, as our original objective was to enrich for larger cell numbers. However, due to technical limitations encountered during cell sorting, yield was lower than anticipated. Considering this, **we implemented a stringent and conservative analytical framework** designed specifically **to ensure robustness under low-cell-number conditions**. We intentionally restricted our conclusions to results that remained stable under these conservative criteria.

Regarding the experimental setting: these populations are intrinsically rare in the BM, and their isolation requires stringent FACS-based enrichment, which inevitably limits cell yield. To maximize detection of transcriptional states while preserving biological relevance, we pooled cells from multiple mice per condition. This strategy is commonly applied in niche-focused single-cell studies to mitigate biological variability while improving representation of rare populations.

Regarding the bioinformatic analysis: We implemented a multi-layered strategy specifically tailored to identify robust signals in the context of limited cell numbers:

(i) *Conservative differential expression framework*. Differential expression analyses were performed using MAST[3], a model-based approach specifically designed for single-cell data that accounts for detection rate variability and zero inflation. Analyses were restricted to genes expressed in at least 30% of cells within a group (min.pct = 0.3), with a minimum difference in detection rate between groups (min.diff.pct = 0.15), thereby reducing spurious associations driven by rare or sparsely expressed genes. Bonferroni-adjusted p-values were applied to control for multiple testing.

(ii) *Balanced Gene regulatory network (GRN) inference*. GRN analysis was conducted using *equalized cell numbers per condition* to avoid bias introduced by unequal cluster sizes and to ensure comparability of regulon activity estimates.

(iii) *Stability assessment of ligand–receptor (L-R) interactions*. L-R analyses incorporated bootstrapping (100 iterations), and *only recurrent interactions were retained*, thereby minimizing instability due to sampling variability.

(iv) *Cross-model and cross-platform validation*. All *major findings were validated across independent systems*, including a second murine model (MIcγ1), VRd-treated mice, bulk RNA-seq of human MSC, and IF analyses in murine and human samples. The recurrence of key transcriptional findings across these orthogonal datasets strengthens confidence in their biological relevance.

(v) *Assessment of pooled-animal contribution*. Because samples were pooled prior to sequencing, *we performed sex-inference analyses based on sex-specific gene expression to ensure that identified cell states were not driven by a single animal*. These analyses confirmed contributions from both male and female mice across EC and MSC states, including the MM2 cluster (Supplementary Fig. 3), supporting that the observed transcriptional programs reflect pooled biological variation rather than individual outliers.

In summary, while the limited number of EC and MSC restricts the depth of single-cell granularity, we adopted a deliberately conservative analytical strategy and emphasize the consistency of transcriptional signatures across models, treatment conditions, and human datasets, which collectively supports the robustness and interpretability of our conclusions.

We have not included this additional clarification in the Methods section of the main text to maintain conciseness. However, if the reviewer feels that its inclusion would improve clarity, we would be very happy to incorporate it into the revised manuscript.

(2) Novelty of the IFN signature

We agree that IFN-associated transcriptional programs have previously been reported in MM PC and immune compartments[4,5], and that limited evidence has suggested an IFNα response in MSC[6,7]. However, prior studies have primarily focused on hematopoietic components of the MM microenvironment and did not investigate premalignant MGUS stages.

The novelty of our findings lies in the identification of a stage-specific IFN signature within EC that extends to MSC, defining a coordinated niche response distinct from the previously described TNFα/NF-κB–dominated inflammatory phenotype in MM stroma[1]. Moreover, through GRN inference analysis, we identified Irf7, Stat1, and Stat2 as candidate upstream regulators of this IFN signature, thereby providing regulatory insight beyond differential expression analyses reported in prior studies. Importantly, this IFN signature is absent at the precursor MGUS stage and emerges specifically at the MM stage in the BIcγ1 model, is not detected in the MIcγ1 model, and is suppressed following VRd therapy. Furthermore, we demonstrate that this niche-associated IFN signature is detectable in MSC from a subset of MGUS and MM patients and is reduced after induction therapy, suggesting a potential translational relevance. Together, these features reinforce the novelty of our findings.

We have not included this additional clarification in the Methods section of the main text to maintain conciseness. However, if the reviewer feels that its inclusion would improve clarity, we would be very happy to incorporate it into the revised manuscript.

(3) Lack of IF quantification

Regarding the IFN signature, we appreciate the reviewer´s valuable suggestion. **We have quantified the previously performed IF experiments** and, where possible, increased the number of samples

(control mice, MM BIc and MM MIc, as well as healthy human samples). As expected, these quantifications confirmed our previous IF imaging results. The associated data and analysis have been incorporated into the IF figures (see Figures 2I and J; 6B; 7G and I; and Supplementary Figure 5B-C).

FIGURE 2

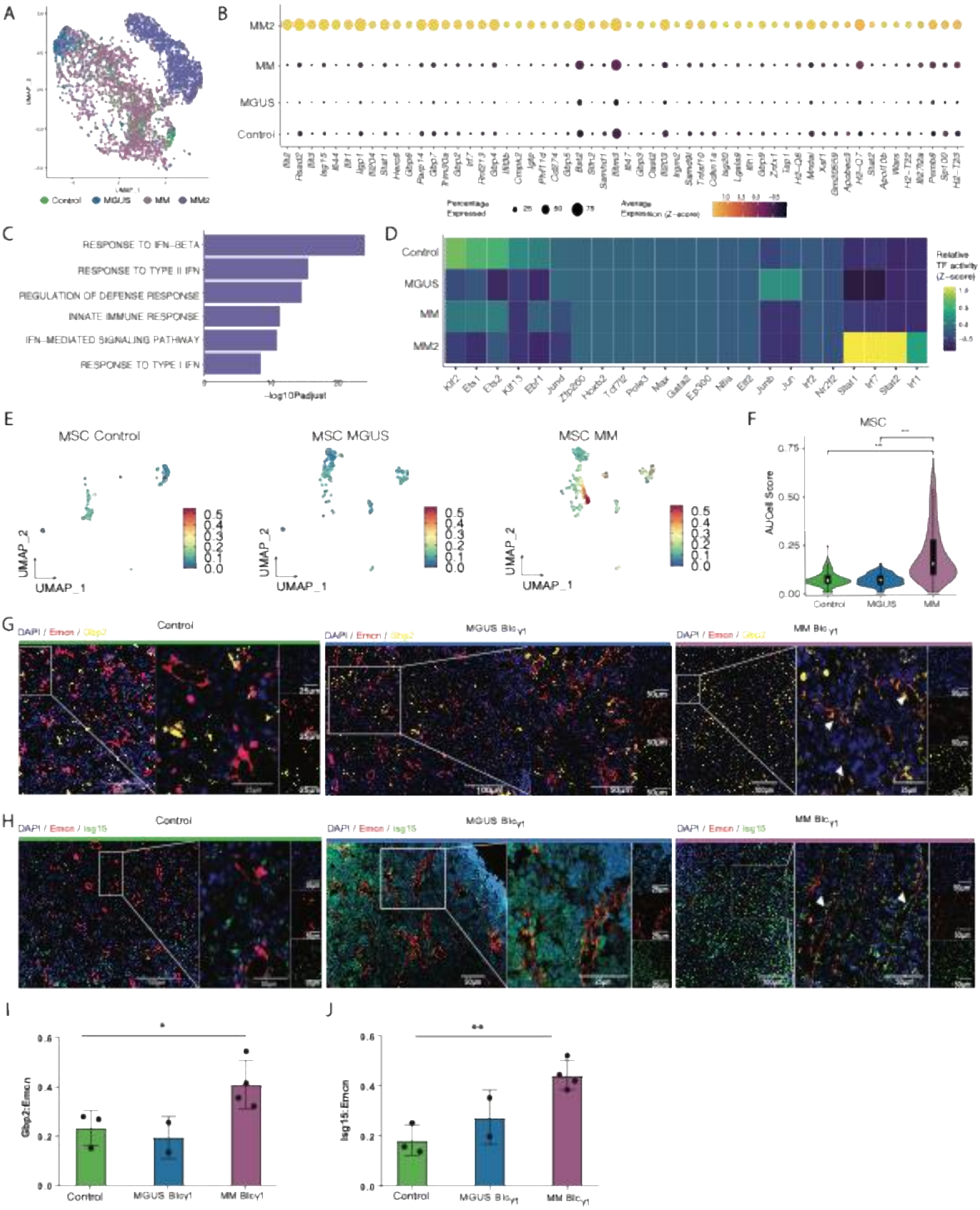

Fig. 2. Identification of a MM-specific IFN signature in the BME.
(A) UMAP dimension reduction of EC revealing a transcriptionally distinct population in MM. (B) Dot plot visualization of the markers defining EC MM2 cluster. Average gene expression is shown by color intensity,

and the percentage of cells expressing each gene is shown by size. (C) Bar charts of enriched GO terms from ORA (adjusted p. value) computed using MM2 cluster markers. The horizontal axis represents the negative of the base 10 logarithm of the adjusted p. value. (D) Heatmap representing z-scaled mean regulon activity score of inferred top specific regulons for each stage. (E) UMAP plot illustrating the distribution of the IFN-related signature in MSC across control, MGUS, and MM. (F) Violin plots showing AUC-score for IFN-related signature within MSC in control, MGUS and MM cells. (G-H) Immunofluorescence (IF) staining of EC (Emcn, red), IFN response genes markers of the MM2 cluster (Gbp2, yellow (G); Isg15, green (H)), and nucleus (DAPI) (blue) in Formalin-Fixed Paraffin-Embedded (FFPE) femurs from Control ($YFP_{c\gamma1}$), MGUS $BI_{c\gamma1}$ and MM $BI_{c\gamma1}$. White arrows point EC Ecmn^{+} IFN^{+} (Gbp2 (G)/Isg15 (H)) in MM $BI_{c\gamma1}$. (I-J) Quantification of the percentage of endothelial IFN-enriched cells, based on the coexpression of Emcn with IFN+ cells (Gbp2, (I); Isg15, (J)) in femurs from Control ($YFP_{c\gamma1}$), MGUS $BI_{c\gamma1}$ and MM $BI_{c\gamma1}$ mice.

FIGURE 6

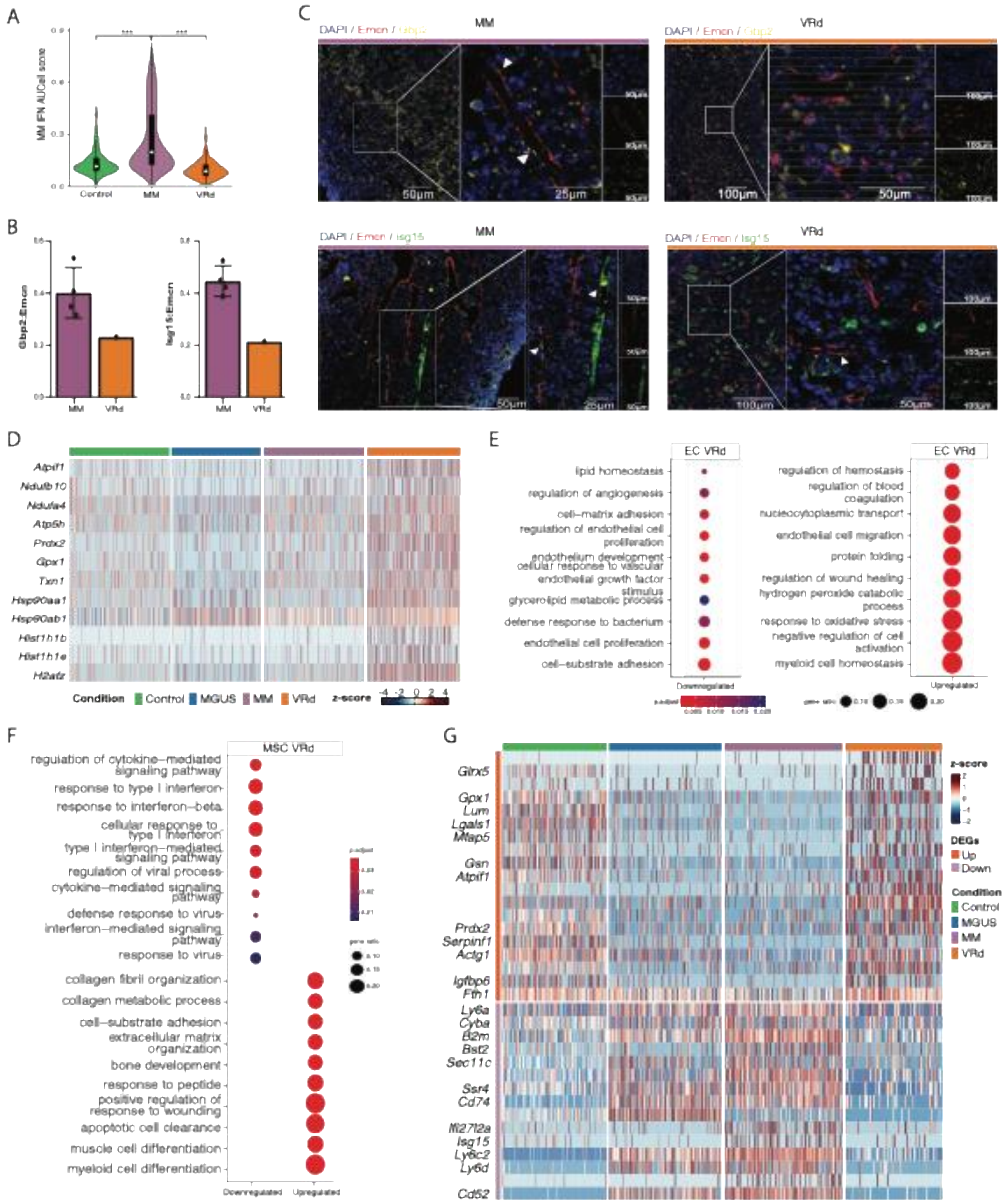

Fig. 6. VRd induces transcriptional remodeling of EC and MSC in MM.
(A) Violin plot showing AUC-score for IFN-related signature in EC from Control ($YFP_{c\gamma1}$), MM $BIc_{\gamma1}$ (MM) and MM $BI_{c\gamma1}$-*Crbn*^{I391V} VRd-treated (VRd) mice. (B) Quantification of the percentage of endothelial IFN^{+} cells, determined by the coexpression of Emcn with IFN response genes (Gbp2 (left plot) and Isg15 (right plot) in femurs from MM $BIc_{\gamma1}$ (MM) and MM $BI_{c\gamma1}$-*Crbn*^{I391V} VRd-treated (VRd) mice. (C) IF staining of EC (Emcn, red), IFN response genes (Gbp2 (yellow, (upper plot)); Isg15 (green, (bottom plot)), and nucleus (DAPI) (blue) in FFPE femurs from MM $BI_{c\gamma1}$ mice (MM) and VRd-treated mice (VRd). White arrows point EC (Ecmn^{+}) IFN^{+} (Gbp2/Isg15). (D) Heatmap showing the expression of stress-related DEGs identified in the comparison between treated and non-treated non-IFN MM EC at different stages of the disease. Color represents the z-score scaled expression values. (E) Significant terms derived from ORA between VRd-treated and untreated non-IFN MM EC. (F) Significant terms derived from ORA of MSC from MM mice before and after VRd treatment. (G) Heatmap projecting the expression of DEGs identified in the comparison between non-treated and treated MSC at different stages of the disease. Color represents the z-score scaled expression values.

FIGURE 7

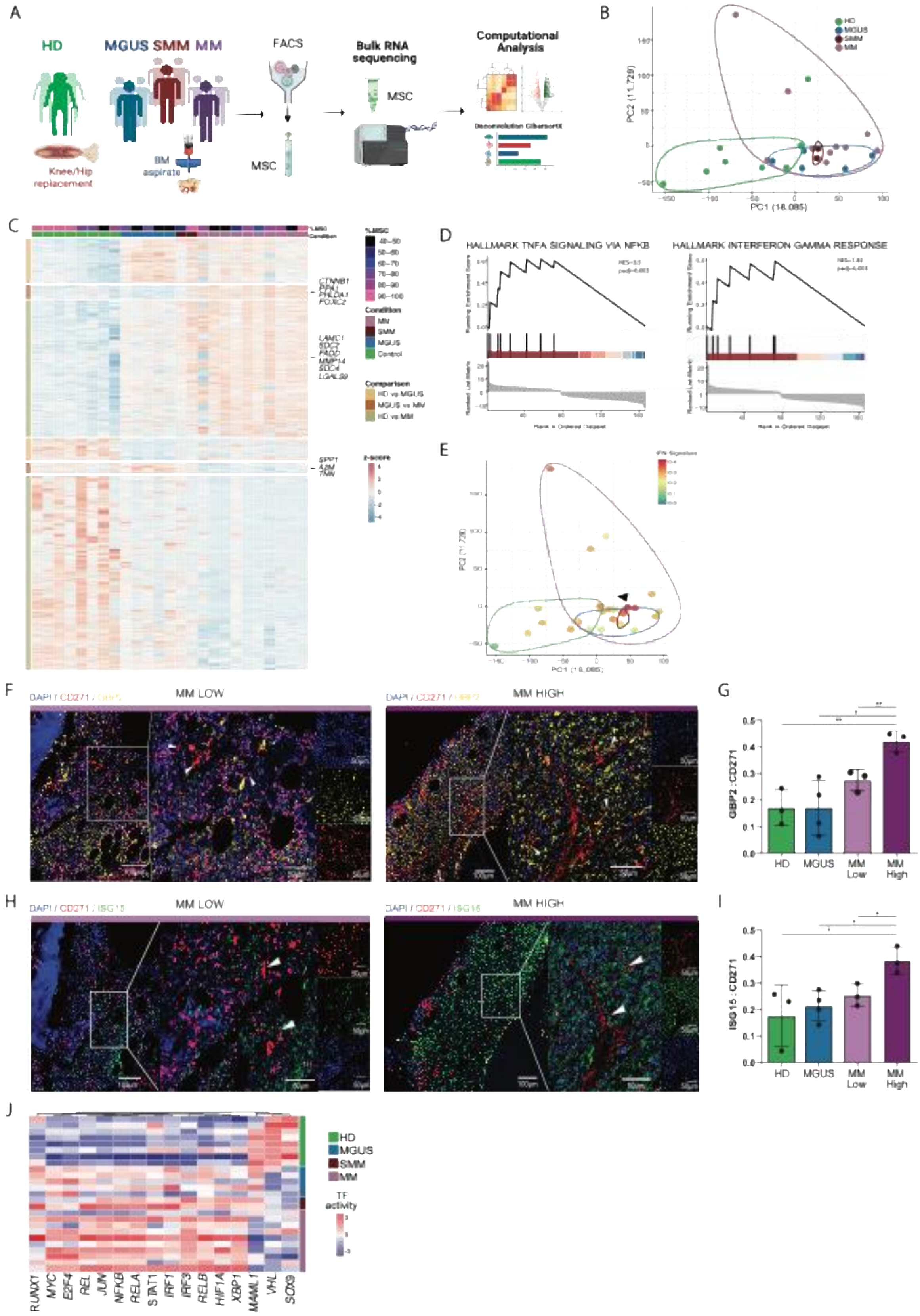
A
HD
MGUS SMM MM
Knee/Hip replacement
BM aspirate
FACS
MSC
Bulk RNA sequencing
MSC
Computational Analysis
B
HD
MGUS
SMM
MM
PC2 (11.728)
PC1 (18.085)
C
CTNNB1
RPA1
PHLDA1
FOXC2
LAMC1
SDC2
FADD
MMP14
SDC4
LGALS9
SPP1
A2M
TNN
%MSC
Condition
MM
SMM
MGUS
Control
Comparison
HD vs MGUS
MGUS vs MM
HD vs MM
z-score
D
HALLMARK TNFA SIGNALING VIA NFKB
HALLMARK INTERFERON GAMMA RESPONSE
E
PC2 (11.728)
PC1 (18.085)
F
MM LOW
MM HIGH
G
GBP2 : CD271
HD
MGUS
MM Low
MM High
H
DAPI / CD271 / ISG15
MM LOW
MM HIGH
I
ISG15 : CD271
HD
MGUS
MM Low
MM High
J
HD
MGUS
SMM
MM
TF activity
RUNX1
MYC
E2F4
REL
JUN
NFKB
RELA
STAT1
IRF1
IRF3
RELB
HIF1A
XBP1
MAML1
VHL
SOX9

Fig. 7. Transcriptional dynamics of human MSC during MM progression.

(A) Experimental workflow used for the isolation and bulk RNA-seq analysis of human BM MSC. (B) PCA of MSC expression profiles shows clustering by disease stage. The color of the dots represents disease stages. (C) Heatmap showing the expression profiles of DEGs identified in each pairwise comparison across disease progression. (D) GSEA plots for the gene sets 'TNFα signaling via NFκB' (left panel) and 'Interferon gamma response' (right panel) in the comparison between healthy donors (HD)- MSC and MM-MSC. (E) PCA plot showing the MM-IFN signature score in each human sample, previously identified murine BM MM cells. (F) IF staining of MSC (CD271, red), IFN response genes (*GBP2*, yellow), and nucleus (DAPI) (blue) in archived paraffin-embedded human BM biopsies of MM patients with low (MM LOW) and high IFN signal (MM HIGH) in stromal cells (see Supplementary Table 2). White arrows point to MSC ($CD271^+$) in MM LOW IFN samples, and MSC ($CD271^+$) IFN^+ (GBP2) in MM HIGH IFN samples. (G) Quantification of the percentage of mesenchymal IFN^+ cells, determined by the coexpression of $CD271^+$ with IFN response genes (*GBP2*) in FFPE biopsies from HD (three donors) and archived paraffined human BM biopsies of MGUS (four patients) and MM patients (six patients with low (three) and high (three) IFN signal in stromal cells). (H) IF staining of MSC (CD271, red), IFN response genes (*ISG15*, green), and nucleus (DAPI) (blue) in archived paraffin-embedded human BM biopsies of MM patients with low (MM LOW) and high IFN signal (MM HIGH) in stromal cells (see Supplementary Table 2). White arrows point to MSC ($CD271^+$) in MM LOW IFN samples, and MSC ($CD271^+$) IFN^+ (ISG15) in MM HIGH IFN samples. (I) Quantification of the percentage of mesenchymal IFN^+ cells, determined by the coexpression of $CD271^+$ with IFN response genes (*ISG15*) in FFPE biopsies from HD (three donors) and archived paraffined human BM biopsies of MGUS (four patients) and MM patients (six patients with low (three) and high (three) IFN signal in stromal cells). (J) Heatmap representing the variation of TF activity score across samples.

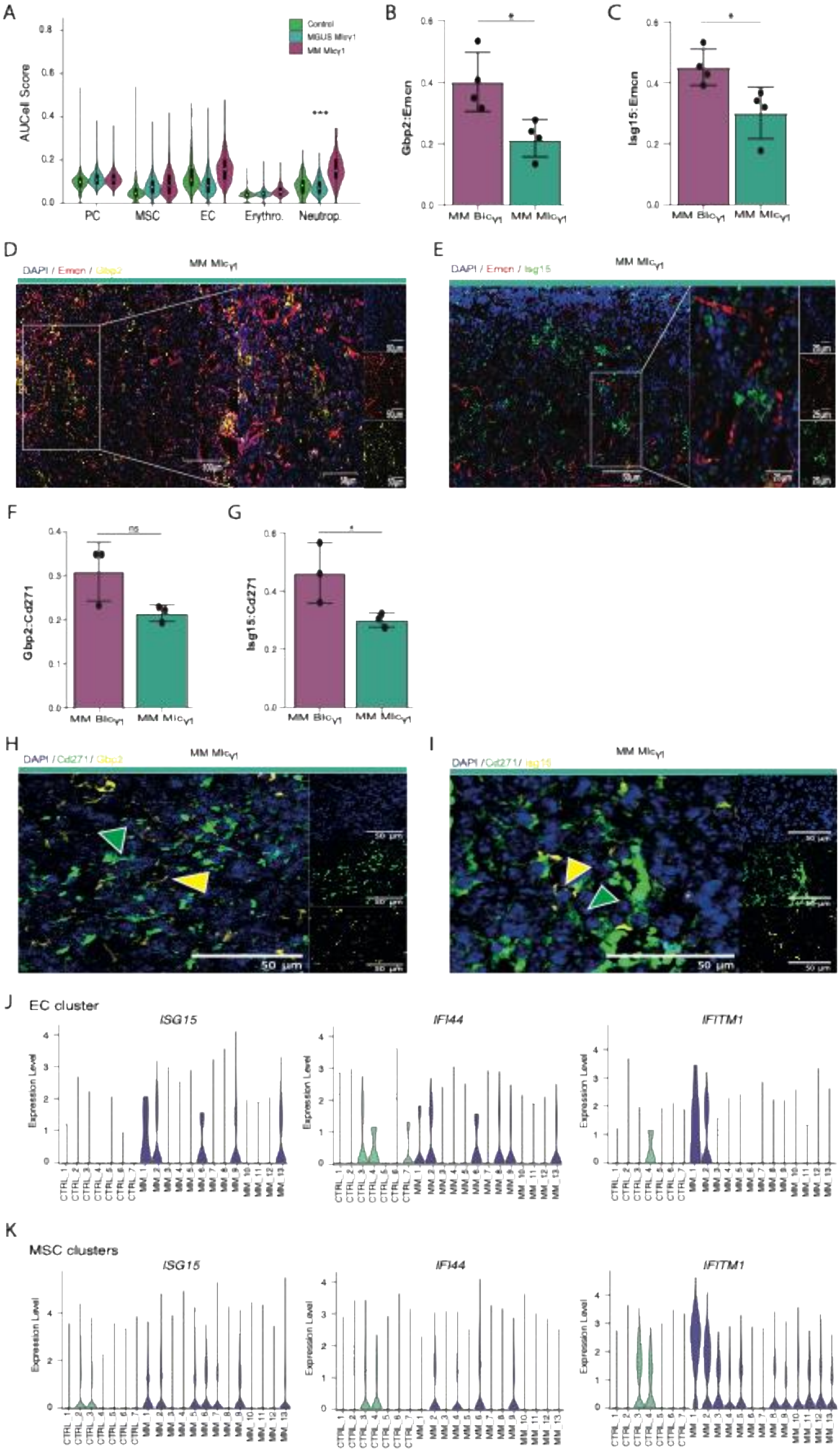
A
Control
MGUS Mic γ1
MM Mic γ1
AUCell Score
***
PC
MSC
EC
Erythro.
Neutrop.
B
Gbp2:Emcn
*
MM Blc γ1
MM Mlc γ1
C
Isg15:Emcn
*
MM Blc γ1
MM Mlc γ1
D
DAPI / Emcn / Gbp2
MM Mlc γ1
E
DAPI / Emcn / Isg15
MM Mlc γ1
F
Gbp2:Cd271
ns
MM Blc γ1
MM Mlc γ1
G
Isg15:Cd271
*
MM Blc γ1
MM Mlc γ1
H
DAPI / Cd271 / Gbp2
MM Mlc γ1
50 µm
I
DAPI / Cd271 / Isg15
MM Mlc γ1
50 µm
J
EC cluster
ISG15
IFI44
IFITM1
Expression Level
K
MSC clusters
ISG15
IFI44
IFITM1
Expression Level

Supplemental Figure 5: Validation in the $MI_{c\gamma1}$ model and scRNA-seq human data.
(A) Violin plots showing AUC-score for MM IFN-related signature within BM populations in the $MI_{c\gamma1}$ model. (B-C) Quantification of the percentage of endothelial IFN-enriched cells, based on the coexpression of Emcn with IFN+ cells (Gbp2, (B); Isg15, (C)) in femurs from MM $BI_{c\gamma1}$ and MM $MI_{c\gamma1}$ mice. (D-E) IF staining of EC (Emcn, red), and IFN response genes markers (Gbp2 (yellow, (D)); Isg15 (green, (E))), and nucleus (DAPI) (blue) in FFPE femurs from MM $MI_{c\gamma1}$ mice. (F-G) Quantification of the percentage of mesenchymal stromal cells IFN-enriched cells, based on the coexpression of $Cd271^{+}$ with IFN+ cells (Gbp2, (F); Isg15, (G)) in femurs from MM $BI_{c\gamma1}$ and MM $MI_{c\gamma1}$ mice. (H-I) IF staining of MSC (Cd271, green), and IFN response genes markers (Gbp2 (yellow, (H)); Isg15 (yellow, (I))), and nucleus (DAPI) (blue) in FFPE femurs from MM $MI_{c\gamma1}$ mice. Green arrows point to MSC ($Cd271^{+}$), and yellow arrows point to IFN^{+} (Gbp2/Isg15). (J) Violin plots comparing the distribution of the gene expression of *ISG15*, *IFI44*, and *IFITM1* in EC from MM patients and healthy controls from scRNA-seq published data[1]. (K) Similar to J for MSC from the same healthy controls and MM human patients from scRNA-seq published data [1].

Moreover, **to further confirm** our interesting **IFN results in MM-MSC** by scRNA-seq, we performed additional IF staining for IFN-signature (Isg15 and Gbp2) in MSC ($Cd271^{+}$ cells) from Ctrl, MGUS $BIc_{\gamma1}$, MM $BIc_{\gamma1}$, MM $MIc_{\gamma1}$, and MM $BI_{c\gamma1}$-*Crbn*^{I391V} VRd-treated (VRd) mice and quantified the staining across all groups. These new IF results recapitulate the pattern previously observed in EC. Specifically, an increase of IFN signaling in MM $BIc_{\gamma1}$ cells in comparison to control, MGUS and MM $MIc_{\gamma1}$, as well as a reduction in MM $BI_{c\gamma1}$-*Crbn*^{I391V} VRd-treated (VRd) mice.

The corresponding results have been incorporated into the Results section (lines 184-185: *We confirm these scRNA-seq results by IF staining of EC and MSC IFN^{+} in BM biopsies from MM $MI_{c\gamma1}$ mice (**Supplementary Fig. 5B-I**)*, and Supplementary Figures 4F–I, and new Supplementary 5 B-I (see previously), and 13I and K.
The experimental details have been added to the Methods section (lines 206-208: *Antigen retrieval was carried out at 95°C for 30 minutes in either 10 mM citrate buffer (pH 6.0) or, specifically for CD271 staining combinations, in 10 mM Tris-1 mM EDTA buffer (pH 9.0)).*
Lines 209-212:
For immunofluorescence (IF) staining, the Formalin-Fixed Paraffin-Embedded (FFPE) sections were blocked with BSA 5% for 30 min at RT. The following primary antibodies were used: rat anti-Endomucin antibody (V.7C7) (Santa Cruz Technology 1: 50 dilution), mouse anti-CD271 monoclonal antibody (NGF Receptor) (ME20.4) (Invitrogen, 1: 50 dilution) …

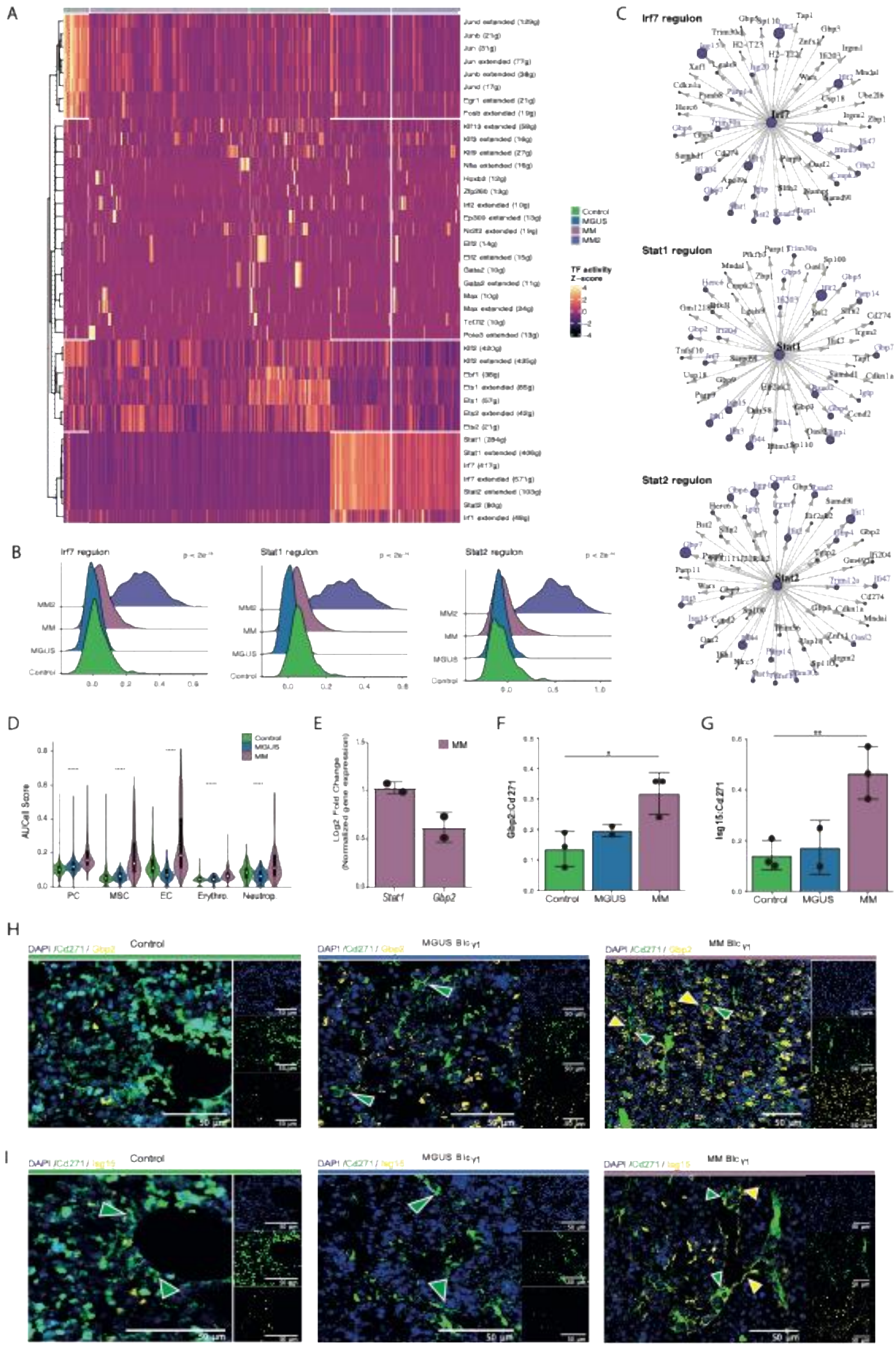

Supplemental Figure 4: Additional information on the MM IFN-related signature in the $BI_{c\gamma1}$ model. (A) Heatmap showing the activities of the enriched regulons in EC (Z-score of regulon activity). (B) Density plots showing the score of Irf7, Stat1, and Stat2 regulons in EC (p. = adjusted p-value). (C) Regulon network of Irf7, Stat1, and Stat2 representing the top 50 target genes. (D) Violin plots showing AUC-score for MM IFN-related signature within BM populations in the $BI_{c\gamma1}$ model. (E) Quantitative PCR (qPCR) analysis of the IFN regulon (*Stat1)* and the associated gene (*Gbp2)* in MSC derived from mesenspheres of MM BIcγ1 mice. Gene expression is presented as log_2 fold change relative to MSC derived from mesenspheres of control mice. Data were normalized to the reference gene *Gapdh*. N=2 independent biological replicates per group. (F-G) Quantification of the percentage of mesenchymal stromal cells IFN-enriched cells, based on the coexpression of $Cd271^+$ with IFN+ cells (Gbp2, (F); Isg15, (G)) in femurs from Control, MGUS $BI_{c\gamma1}$ and MM $BI_{c\gamma1}$ mice. (H-I) IF staining of MSC (Cd271, green), and IFN response genes markers (Gbp2 (yellow, (H)); Isg15 (yellow, (I))), and nucleus (DAPI) (blue) in FFPE femurs from Control, MGUS $BI_{c\gamma1}$, and MM $BI_{c\gamma1}$ mice. Green arrows point to MSC ($Cd271^+$), and yellow arrows point to IFN^+ (Gbp2/Isg15).

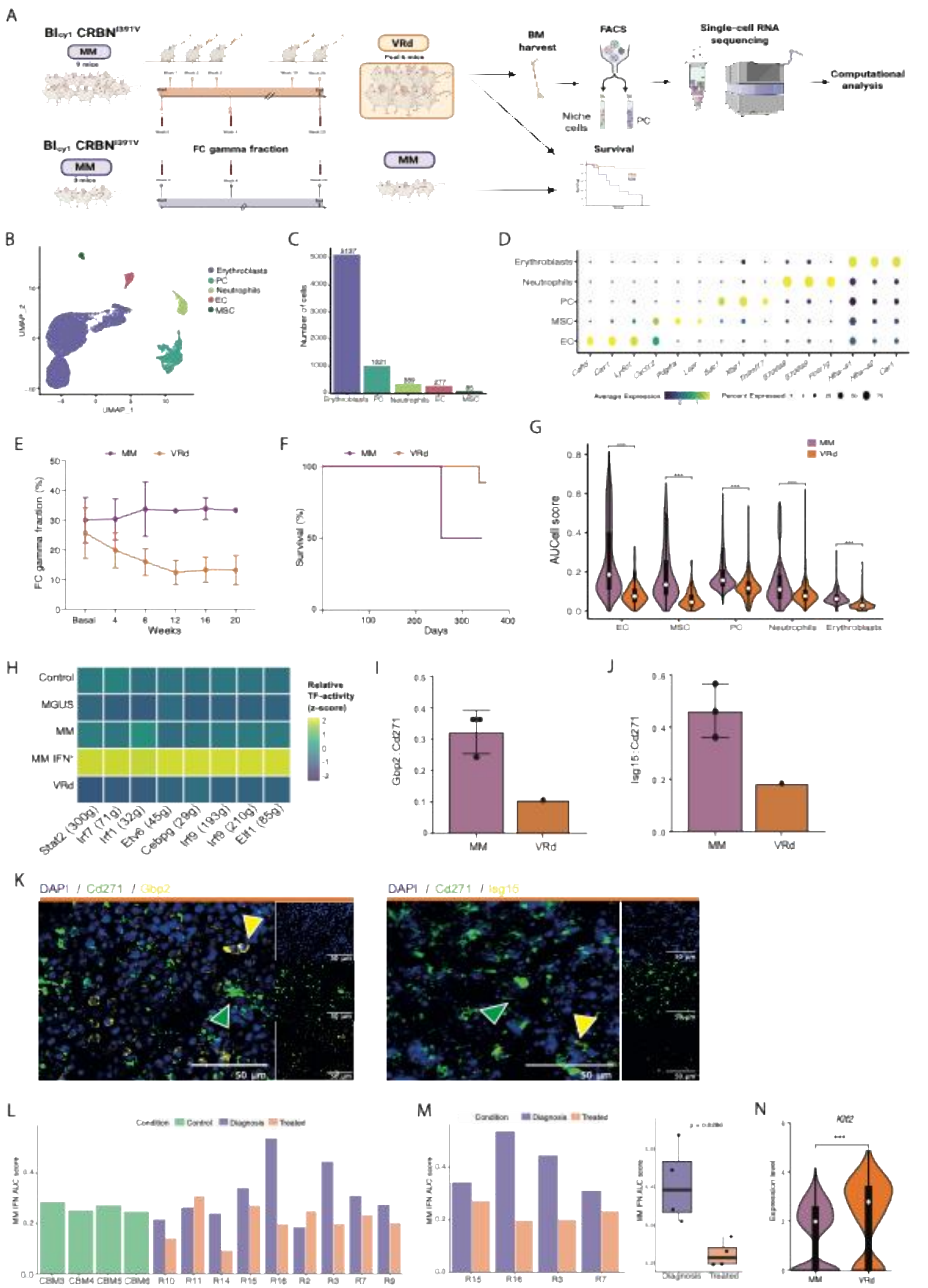

Supplemental Figure 13: Additional analysis of VRd-treated mice and transcriptional alterations in EC and MSC from human patients. (A) VRd experiment overview representing treatment administration, serum collection for FC gamma fraction, and scRNA-seq isolation of PC, MSC, and EC from MM BI$_{c\gamma1}$ *Crbn*^{I391V} (pool of six mice). MM BI$_{c\gamma1}$ *Crbn*^{I391V} were used as controls for FC gamma fraction and

survival. (B) UMAP representation of BM microenvironment cells from $BI_{c\gamma1}$-*Crbn*^{I391V} VRd-treated mice, colored by cell clustering. (C) Bar plots showing the number of cells per BM population. (D) Dot plot of canonical markers used to define PC, EC, MSC, erythroblasts, and neutrophils. The dot size reflects the percentage of cells within the cluster expressing each gene, and the color represents the average expression level. (E) Representation of the percentage of serum FC gamma fraction in VRd-treated versus the MM group. (F) Kaplan–Meier survival curves of treated and non-treated MM $BI_{c\gamma1}$ mice. (G) Violin plots showing AUC-score for MM IFN-related signature within BM populations in VRd-treated and MM $BI_{c\gamma1}$ mice. (H) Heatmap representing z-scaled mean regulon activity score of IFN-related regulons in EC across each condition. (I-J) Quantification of the percentage of mesenchymal IFN^+ cells, determined by the coexpression of Cd271 with IFN response genes (Gbp2 (I) and Isg15 (J) in femurs from MM $BIc_{\gamma1}$ (MM) and MM $BI_{c\gamma1}$-*Crbn*^{I391V} VRd-treated (VRd) mice. (K) IF staining of MSC (Cd271, green), IFN response (Gbp2, yellow (left plot) and Isg15, yellow (right plot)), and nucleus (DAPI) (blue) in FFPE femurs from VRd-treated mice. Green arrows point to MSC ($Cd271^+$), and yellow arrows point to IFN^+ (Gbp2/Isg15). (L) Bar plots showing the AUC-score for MM IFN-related signature in MSC from healthy controls and MM human patients before and post-induction treatment from bulk RNA-seq published data[1] . (M) Bar plots showing the AUC-score for MM IFN-related in MSC from the subset of high IFN MM human patients before and after induction treatment[1] by individuals (left panel) and grouped by disease (right panel). (N) Violin plot displaying the upregulation of *Klf2* in VRd-treated EC vs non-treated MM EC.

(4) Purity of human MSC samples

We thank the reviewer for raising the issue of MSC purity in human samples. We would like to emphasize that the **human bulk RNA-seq analysis was designed primarily to validate murine findings rather than to serve as a discovery platform**. Accordingly, our conclusions are anchored in the murine single-cell datasets and supported by consistent transcriptional patterns observed in human MSC.

Given the low abundance of MSC in human bone marrow aspirates, we implemented multiple complementary strategies to ensure sample integrity, minimize potential contamination, and retain only robust signals. Our analytical framework included:

- (i) Prospective FACS isolation using a stringent immunophenotype,
- (ii) **Identification of low-purity samples** through CIBERSORTx-based deconvolution to estimate contaminating cell fractions (see Supplementary Fig. 17A), followed by exclusion of highly contaminated samples,
- (iii) **Confirmation of mesenchymal identity** using AUCell-based MSC signature scoring to verify enrichment of bona fide MSC transcriptional programs.,
- (iv) **Contamination as a covariate**, incorporating estimated MSC proportions as a covariate in differential expression analyses to account for residual variability.
- (v) **Exclusion of contamination-associated genes**, removing genes significantly correlated with contaminating cell fractions from downstream analyses.

Finally, after determining MSC proportions, we further confirmed the identity of the filtered MSC population by the expression of canonical MSC markers (see Supplementary Fig. 17B). Together, these orthogonal quality-control and analytical steps ensure that the reported transcriptional changes reflect bona fide MSC-associated signals rather than artifacts of contaminating populations.

Reviewer #3 (Remarks to the Author):

The study of Cenzano and Cocera et al takes advantage of recent advances in genetically-engineered murine models of myeloma to probe the interaction between malignant plasma cells and the non-immune component of the bone marrow microenvironment. The authors rightly address a gap in the MM field where almost all microenvironment studies only focus on immune components of the microenvironment, ignoring the non-immune components like endothelial cells and mesenchymal stromal cells. Here to profile these cells the authors largely deployed flow cytometry-based sorting followed by single cell sequencing and associated bioinformatics. There is immunohistochemistry level validation in some cases. The authors' major biological finding is an interferon-like signature in the MSCs of some of the analyzed mice that is recapitulated in a similar limited fraction of European-descent patients. The authors also show that this signature can be reversed by VRd treatment in mice.

Overall, this study appears to well executed from a technical perspective. While it is a largely descriptive study, it likely has relevance in the myeloma field in terms of elucidating potentially relevant biology not previously explored.

We acknowledge and appreciate the reviewer's positive feedback regarding our work.

However, the main critique is that based on the results of Fig. 7, it seems that a lot of the relevant biological pathways could also be identified in MSCs from humans. Therefore, this final figure begs the question: what is really the **advantage of using the mouse model for this study**? Why not just study the human and go from there? In general, the advantage of mouse modeling is the ability to selectively perturb the system to test specific hypotheses, which is often highly challenging if not impossible to do in humans, depending on the question. Here, some of this testing takes place with the one VRd treatment study. But in general everything else presented is a static picture. The authors in particular rely heavily on bioinformatic analysis of predicted transcriptional **"regulons" as well as prediction of receptor-ligand interactions**; however, these analyses can both readily have false negatives and false positives. Therefore, performing additional mechanistic studies testing at least one or two biological hypotheses extending from the descriptive studies here would provide significant value to the paper by more clearly demonstrating the utility of monitoring MSC dynamics in this murine model.

Before addressing the reviewer's, major comments point by point, **we would like to further justify the use of the MM mouse model** developed by Larrayoz et al. in Nature Medicine[8].

In our view, the MM mouse model described by Larrayoz et al. offers several important **advantages** for the current study and for future investigations, both in comparison with human samples and in relation to other available models.

Benefits of Larrayoz et al. MM mouse model when comparing with human samples

1. Isolation of enough number of non-hematopoietic bone marrow (BM) cells

These models enable the recovery of substantially more non-hematopoietic BM cells. In mice, we collect not only BM cells but also bone fragments, which are mechanically crushed and enzymatically digested (collagenase treatment), thereby maximizing cell yield from the niche. In contrast, human BM samples obtained by aspiration contain a relatively low proportion of non-hematopoietic stromal cells. As previously reported, this limited yield makes the reliable isolation of endothelial cells (EC) from human aspirates technically challenging, if not unfeasible[9].

As we demonstrate in figure 7, we only performed bulk RNA-seq due to the limited number of cells.

However, following the reviewer´s comment, we consider conducting **scRNA-seq experiments on BM samples from MGUS-MM patients**. Before submitting the manuscript, we attempted this; however, we could not obtain enough non-hematopoietic BM cells (EC and MSC) to perform scRNA-seq

successfully. To address this limitation in our experimental design, we validated our murine MM-specific IFN signature using human MSC data from publicly available datasets[1] (see **Supplemental Figure 5J–K**). Interestingly, we observed enrichment of this signature in a subset of MM patients, and notably, this enrichment appeared to be independent of underlying genomic alterations.

Following the revision process and in response to the reviewer's comments, we collected BM samples from HD, MGUS-SMM, and MM patients over the past several months. We subsequently conducted two independent scRNA-seq experiments using **On-Chip Multiplexing (10x Genomics)**, in which multiple samples, HD (n=1), MGUS (n=4), SMM (n=2) and MM (n=2) were pooled to maximize cell recovery and sequencing efficiency. Despite this, after sequencing and manual annotation, we found that the number of MSC cells obtained was very low compared to publicly available datasets[1].

	Total Cells (x106)	Sorter Events EC	Sorter Events MSC	Sorter Events PC	Cells Cell Counter	Viability (niche+PC)	Cells	Confidently mapped reads in cells	Median genes per cell	Median UMI counts per cell	Total genes detected
**HD_1**	12	162	484	6106	3533	43%	381	86,70%	2.855	10.669	20.961
**MGUS_1**	33	49	759	21,6 K	No count		479	72%	2.054	5.664	22.756
**MGUS_2**	17	947	457	40 k	No count + 4450 (PC)	66% (PC)	1.628	88%	3.789	12.203	27.258
**MGUS_3-4**	71	1474 + 874	527+3	4445+43231	280 + 2000 PC + 2000 PC	100%, 56% and 40%	1542	89,50%	2.236	6.437	25.392
**SMM_1**	32	5359	3290	227,68 K	1473 + 3900 (PC)	34% + 72%	1.258	95,20%	5.991	53.436	27.206
**SMM_2**	39	124	2004	92,422 K	280 + 4000 (PC)	100% +38%	1630	90,20%	4.036	19.192	26.598
**MM_1**	31	714	73	108,53 K	No count + 3542 (PC)	70% (PC)	1.512	92%	5.124	27.778	27.474
**MM_2**	22	866	721	980,74 k	No count + 5000 (PC)	77%	2.832	86,30%	2.495	6.789	26.995

Despite the limited number of cells, we proceeded with manual annotation of all captured cells. For reference, the number of cells per population is shown on the left, and the number of MSC per sample is displayed on the right.

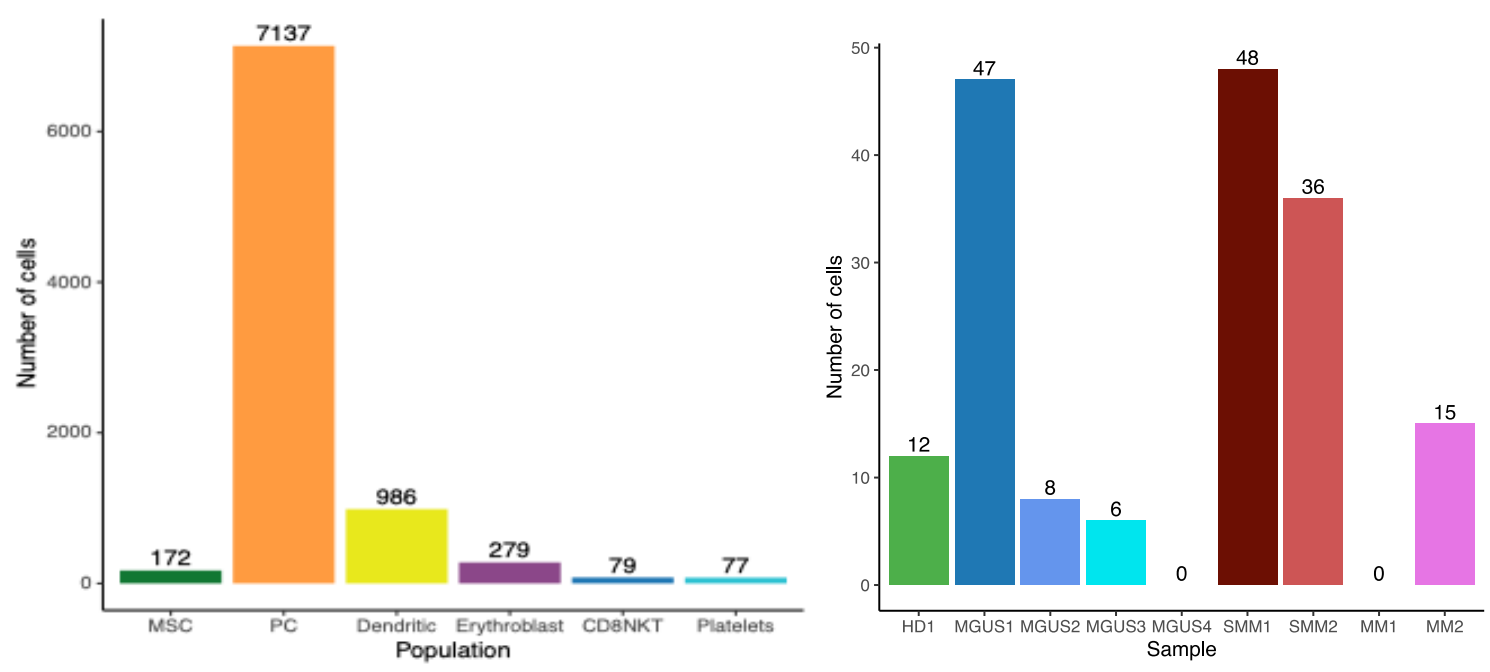

We confirmed the identity of each cell type based on the expression of well-established canonical markers, as shown below.

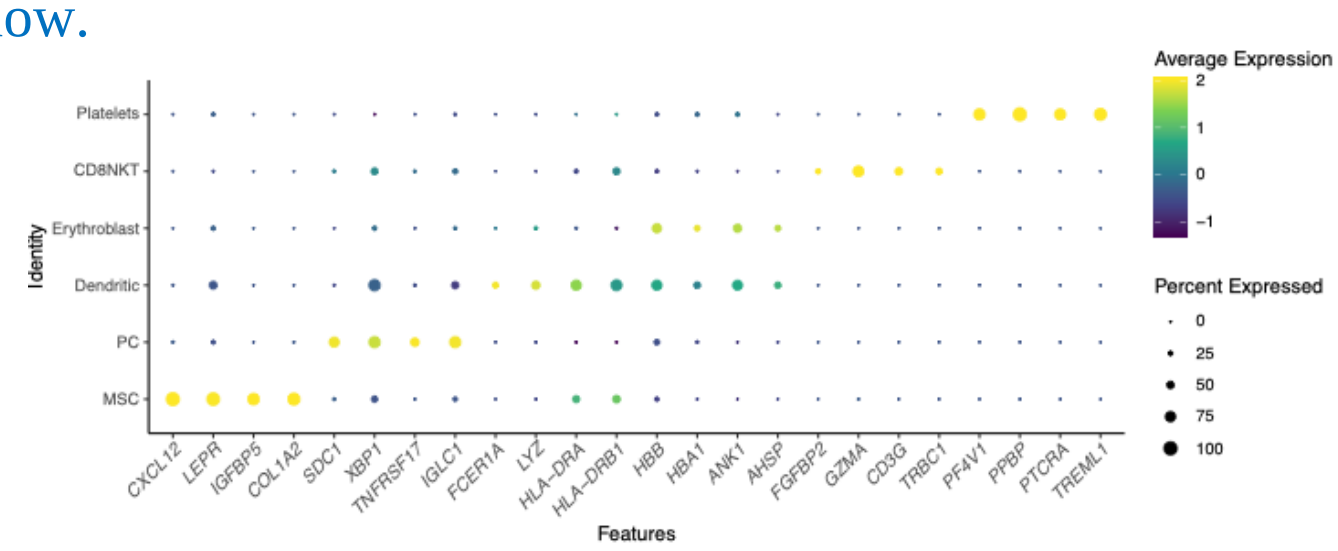

Moreover, we decided to merge these samples with freshly collected elderly HD and MM samples available samples in our laboratory to perform an exploratory analysis.

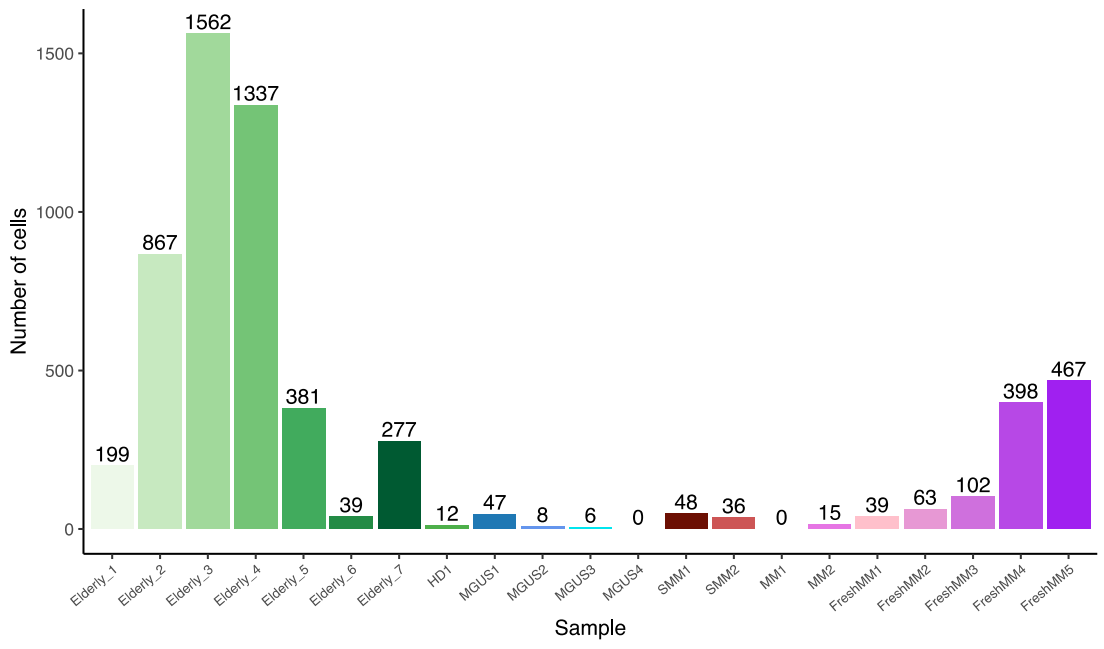

As a first step, we projected the MM-IFN signature onto MSC from all available samples. Consistent with our analysis using the Cupedo dataset[1] (see Supplemental Figure 4I–J) and our human bulk RNA-seq data (see Figure 7E) we observed enrichment of this MM-IFN signature only in a subset of MM patients, specifically in MM2 and MM4 fresh samples.

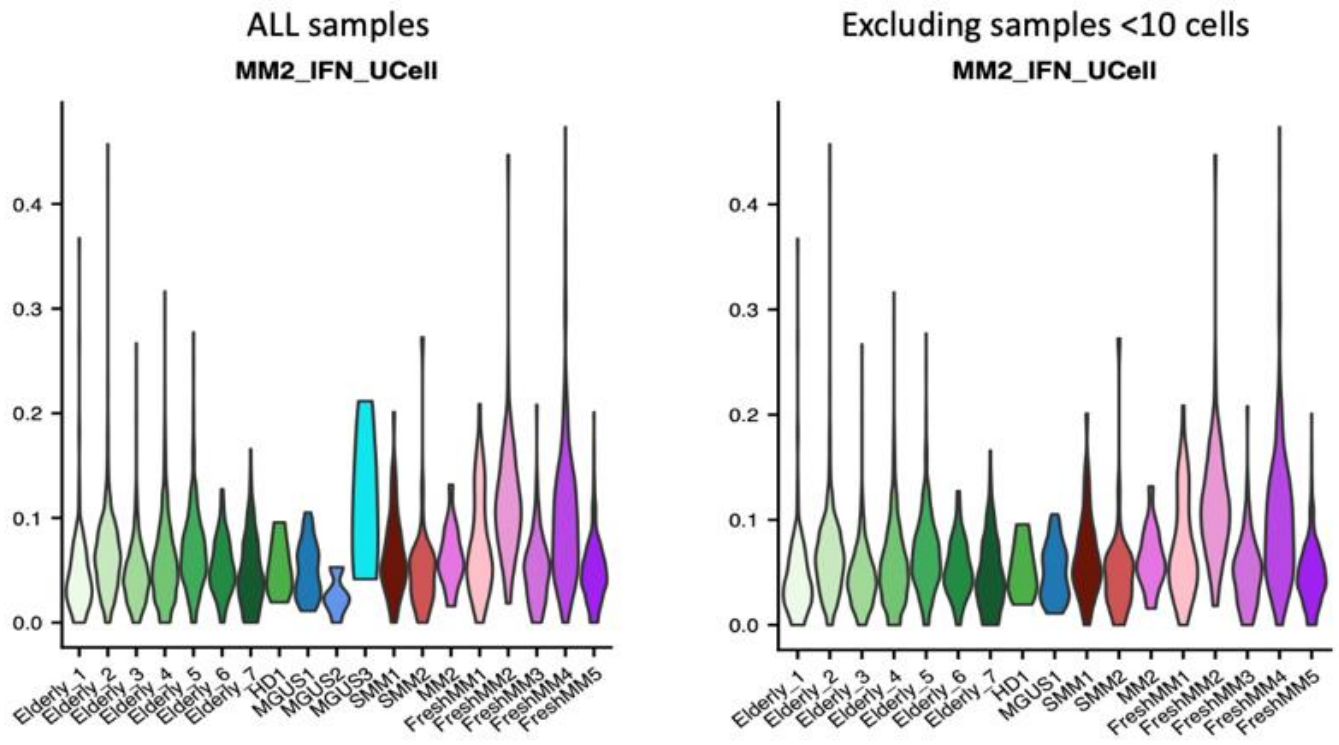

Interestingly, we observed upregulation of *CD74*, an antigen presentation-related gene, across the MGUS-SMM-MM samples, consistent with our previous findings in the MM mouse model. This confirms a potential role for *CD74* in myeloma progression.

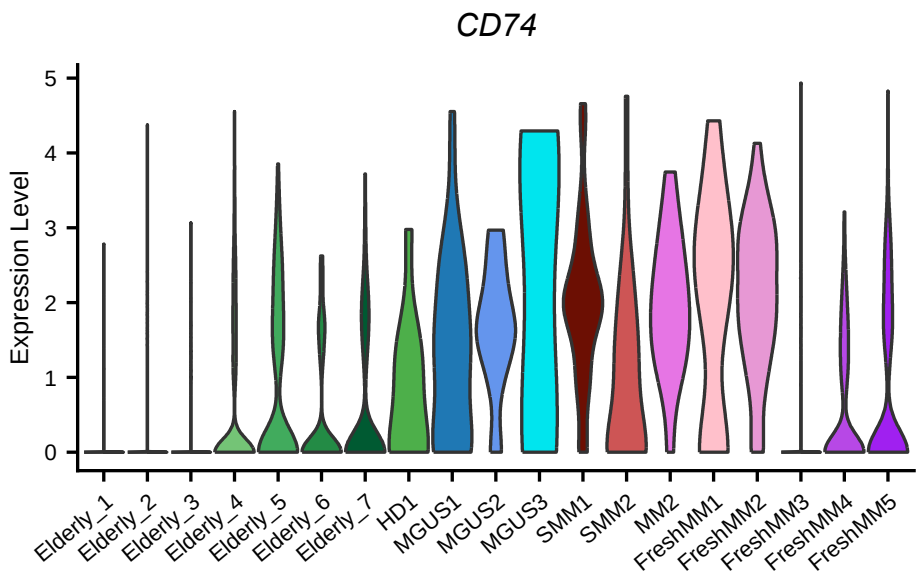

Taken together, these preliminary scRNA-seq results of MSC during MM progression support the relevance of the MM-IFN signature in both murine and human disease. Additionally, they validate the expression of several DEGs previously identified in our murine scRNA-seq analysis.

Due to these limitations, we have decided not to include these new experiments in the revised manuscript. Nevertheless, we felt it was important to share this effort with the reviewer to highlight the advantages to use the used MM mouse models.

2. Manipulation of non-hematopoietic BM niche cells

These MM mouse models provide a powerful and versatile platform to genetically and functionally manipulate EC and MSC *in vivo*. Because the disease arises spontaneously in an immunocompetent host and within an intact microenvironment, stromal cell populations can be specifically targeted using

lineage-restricted Cre drivers, inducible systems, or viral approaches without disrupting the overall niche architecture. This enables precise dissection of stromal-intrinsic pathways and their roles in tumor initiation, progression, immune modulation, and therapeutic resistance. Importantly, selective modulation of defined signaling pathways in MSC, EC, or other niche components allows causal validation of candidate mechanisms and facilitates the identification and preclinical evaluation of novel stromal-dependent therapeutic targets in a physiologically relevant context.

3. Evaluation of new therapeutic strategies

These MM mouse models also enable the anticipation of treatment responses to next-generation immunotherapies and allow the experimental modeling of clinical scenarios associated with poor prognosis, such as high-risk multiple myeloma, extramedullary disease, or acquired therapeutic resistance. This provides valuable opportunities to investigate innovative therapeutic strategies and to optimize the design of future immunotherapy-based clinical trials. Moreover, these models can be used to test novel treatments at disease stages in which MM cells may be particularly vulnerable, including early precursor conditions or the minimal residual disease (MRD) state following therapy.

Benefits of Larrayoz et al. MM mouse model when comparing with other models

1. Disease development

Unlike transplantation-based systems, this model develops MM spontaneously and progressively, recapitulating the transition from precursor stages (e.g., MGUS) to MM. This feature enables the study of stromal alterations not only in established disease but also during early transformation and progression.

2. Intact and evolving microenvironment

Because MM develops over time, the BM niche progressively adapts, closely mimicking the natural history of human disease. As these models do not rely on the injection of artificial tumor cells, they avoid confounding effects associated with irradiation or transplantation-induced niche disruption. This makes them particularly well suited to dissecting intrinsic stromal alterations, inflammatory signaling pathways, and niche-dependent mechanisms that drive MM progression and influence therapeutic response.

We believe that our previous response has carefully addressed the reviewer's question regarding the advantages of using the mouse model in this study. We hope that our explanation now more clearly conveys the rationale for employing the described MM mouse models and clarifies their relevance to our experimental objectives. However, we have not incorporated these additional clarifications into the Methods section of the main text. However, if the reviewer considers that their inclusion would improve clarity, we would be pleased to revise the manuscript accordingly.

Major comments:

1. As noted above, the main gap in the manuscript is some additional mechanistic investigation of any of the findings in the paper, particularly around transcriptional regulation pathways or relevant cell-cell interactions between plasma cells and non-immune microenvironment cells. I don't want to be prescriptive into what exactly needs to be explored – I'll leave this up to the authors – but I see this as critical to showing the value of exploring these cellular phenotypes in the murine models. It would be great to validate one aspect of the regulon analysis and another from the cell-cell interaction predictions in particular, if possible.

We thank the reviewer for this valuable and insightful suggestion, which also reflects a point of internal discussion within our team. As suggested, we attempted to perform additional experiments to functionally validate our scRNA-seq findings.

Importantly, disease development of the used MM mouse models (MM-MI$_{c\gamma1}$ mice and MM-BI$_{c\gamma1}$ mice) requires approximately 6–12 months, which constrains the rapid generation of new experimental cohorts. For this reason, given the review timeline and the limited availability of these MM mouse models, the additional experiments were performed using BM-MSC derived from mouse mesenspheres[10] that were already being maintained in our laboratory at the time of the current revision.

Mesenspheres were generated from mouse primary BM cells as previously described[10], with the exception that CD31^{+} cells were not depleted to remove EC. In our case, BM cells were immunomagnetically depleted of hematopoietic (Cd45^{+}) and erythroid (Ter119^{+}) cells prior to plating. Mesenspheres were obtained from control mice (4-6 months of age) and MM-BI$_{c\gamma1}$ mice (12 months of age). In each experimental condition, cells were pooled from three independent mice. After two weeks of mesenpheres formation, cells were harvested, total RNA was isolated, and RT-PCR and quantitative PCR (qPCR) analyses were performed. MM-associated differences were assessed by qPCR evaluation of the previously identified regulons (*Stat1*, *Yy1*, *Xbp1* and *Elk3*) and IFN gene (*Gbp2*) derived from our single-cell RNA-sequencing (scRNA-seq) dataset.
As we expected, these new data demonstrate an enrichment of these regulons and associated IFN gene *Gbp2* in the MM-BI$_{c\gamma1}$ mice compared with control mice.

The corresponding results have been incorporated into the Results section (lines 172-176: *To further validate the MM-specific IFN signature, we performed quantitative PCR (qPCR) analysis of the IFN regulon (gene Stat1) and the associated gene (Gbp2) in MSC derived from mesenspheres[21] of control and MM BIcγ1 mice (**Supplementary Fig. 4E**). We observed an enrichment of both the IFN Stat1 and Gbp2 expression in MSC from MM BIcγ1 mice compared with controls, confirming activation of the IFN signaling.* These results are summarized in Supplementary Figure 4E (see below).
Also reflected in lines 243-246: *We next validated the activation of these regulons in MM MSC derived from mesenspheres[10] of control and MM BIcγ1 mice by qPCR analysis (**Supplementary Fig. 10D**). Consistent with our previous findings, we observed an increased expression of these regulons in the MM stage compared with controls)* and summarized in Supplementary Figure 10D (see below).
The experimental details have been added to the Methods section (lines232-290: …).
Mesensphere isolation and culture
Mesenspheres were generated from mouse primary BM cells as previously described by Forte et al.[10] Clean mouse bones were crushed in a mortar with 2 ml of 0.25% Collagenase Type I (Stem Cell Technologies). The resulting suspension was incubated for 45 minutes at 37 °C in agitation. The enzymatic reaction was quenched with PBS+ 2% FBS, and cells were filtered through a 40 µm cell strainer. The erythrocytes in the cell suspension were lysed by incubation for 10 minutes with RBC Lysis Buffer at RT. Then, erythroid and hematopoietic lineages were magnetically depleted. Therefore, cells were incubated with biotin-conjugated primary antibodies against Cd45 and Ter119 (BD Biosciences, 1:100) followed by incubation with streptavidin-conjugated magnetic beads (BD Biosciences). The negative fraction was plated at low density (<500,000 cells/cm2) in ultralow-adherence 35 mm dishes (StemCell Technologies) previously coated with Poly-Hema (Sigma). The growth medium for spheres contained 0.1 mM β-mercaptoethanol; 1% nonessential amino acids (Sigma); 1% N_2 and 2% B27 supplements (Invitrogen); recombinant human fibroblast growth factor (FGF)-basic, recombinant human epidermal growth factor (EGF), recombinant human platelet-derived growth factor (PDGF-AA), recombinant human oncostatin M (227 aa OSM) and recombinant human IGF-1 in Dulbecco's modified Eagle's medium (DMEM)/F12 (1:1) / human endothelial (1:2) serum-free medium (Invitrogen). All cytokines (Preprotech) were added at a final concentration of 20 ng/ml, except for IGF-1, which was added at 40 ng/ml. Additionally, mesenspheres medium was supplemented with 15% chicken embryo extract prepared as described previously[11]. Fertilized chicken eggs were incubated for 11 days at 38°C in a humidified incubator. Embryos were washed with DMEM (Invitrogen), macerated by passage through a 50 ml syringe, and diluted 1:1 in the same medium. Hyaluronidase (2 mg, Sigma) was added to each 50 ml tube and incubated for 1h at 4°C with agitation. Following 6 h ultracentrifugation ($3x10^4$ g) at 4°C was decanted and filtered with 0.45 µm and 0.22 µm sterile filters (Millipore). Aliquots were stored at -80°C until use. The cultures were incubated at 37°C with 5% CO_2, 20% O_2 in a water-jacketed incubator and left untouched for 1 week. Afterwards, half-medium changes were performed twice a week. Following a 14-day incubation period, the resulting

spheres were harvested for downstream applications, including RNA isolation and co-culture experiments. Cell cultures were incubated at 37°C with 5% CO_2. After 1 week without medium changes, half-medium changes were performed twice weekly. After 2 weeks of culture, mature spheres were harvested for RNA isolation or co-culture assays. The mesenspheres used in this paper were obtained from control mice (4 months old) and MM mice. ($BIc_{\gamma 1}$ and $MIc_{\gamma 1}$, 6–12 months old). Tumor burden was quantified by flow cytometry as the percentage of PC (GFP^+) within the total BM population. For all MM-derived MSC mesenspheres cultures, mice were selected based on a confirmed tumor burden of 3%-8% PC. For each independent experiment, mesenspheres were generated from a pool of BM cells harvested from three mice.

RNA isolation and qPCR

Total RNA was isolated using the MagMAX mirVana Total RNA Isolation Kit (Applied Biosystems, Thermo Fisher Scientific) according to the manufacturer's instructions and finally resuspended in 14 µL of Elution Buffer (EB) provided with the kit. RNA concentration was determined using the Qubit 3.0 Fluorometer with the Qubit RNA HS Assay Kit (Thermo Fisher Scientific), and RNA integrity and quality were assessed using the Agilent 4200 TapeStation System (Agilent Technologies), following the manufacturers' protocols. Reverse transcription was performed using PrimeScript RT reagent Kit (Takara, Kusatsu, Japan), following the manufacturer's recommendations. qPCR was performed using the PowerUp SYBR Green Master Mix (Applied Biosystems, Waltham, MA, USA) and QuantStudio 5 Real Time PCR System (Thermo Fisher Scientific, Waltham, MA, USA). To quantify gene expression ΔΔCt method was employed. Gene expression was normalized to housekeeping gene Gapdh, to calculate ΔCt. Then ΔΔCt values were obtained by comparing ΔCt the samples of interest to ΔCt mean of two control samples (ΔCt sample – Control mean ΔCt). Final expression levels were calculated as 2^-ΔΔCt and subsequently transformed to log2(2-ΔΔCt) for statistical analysis and comparison against the control baseline. The sequences of the primers used were as follows: mouse Stat1 forward: GAACGCGCTCTGCTCAA; reverse: TGCGAATAATATCTGGGAAAGTAA. Mouse Gbp2 forward: AGATGCCCACAGAAACCCTCCA; reverse: AAGGCATCTCGCTTGGCTACCA. Mouse Yy1 forward: TCAGACCCTAAGCAACTGGCAGAA; reverse: TTGAGCTCTCAACGAACGCTTTGC. Mouse Xbp1 forward: GGGTCTGCTGAGTCC; reverse: CAGACTCAGAATCTGAAGAGG. Mouse Elk3 forward: ACACTTCTGGAGCAGCCTTAGTC; reverse: CATGTGACCGTTGAGCAGTGTG. Housekeeping gene, mouse Gapdh forward: AGTGGCAAAGTGGAGATT; reverse: GTGGAGTCATACTGGAACA.

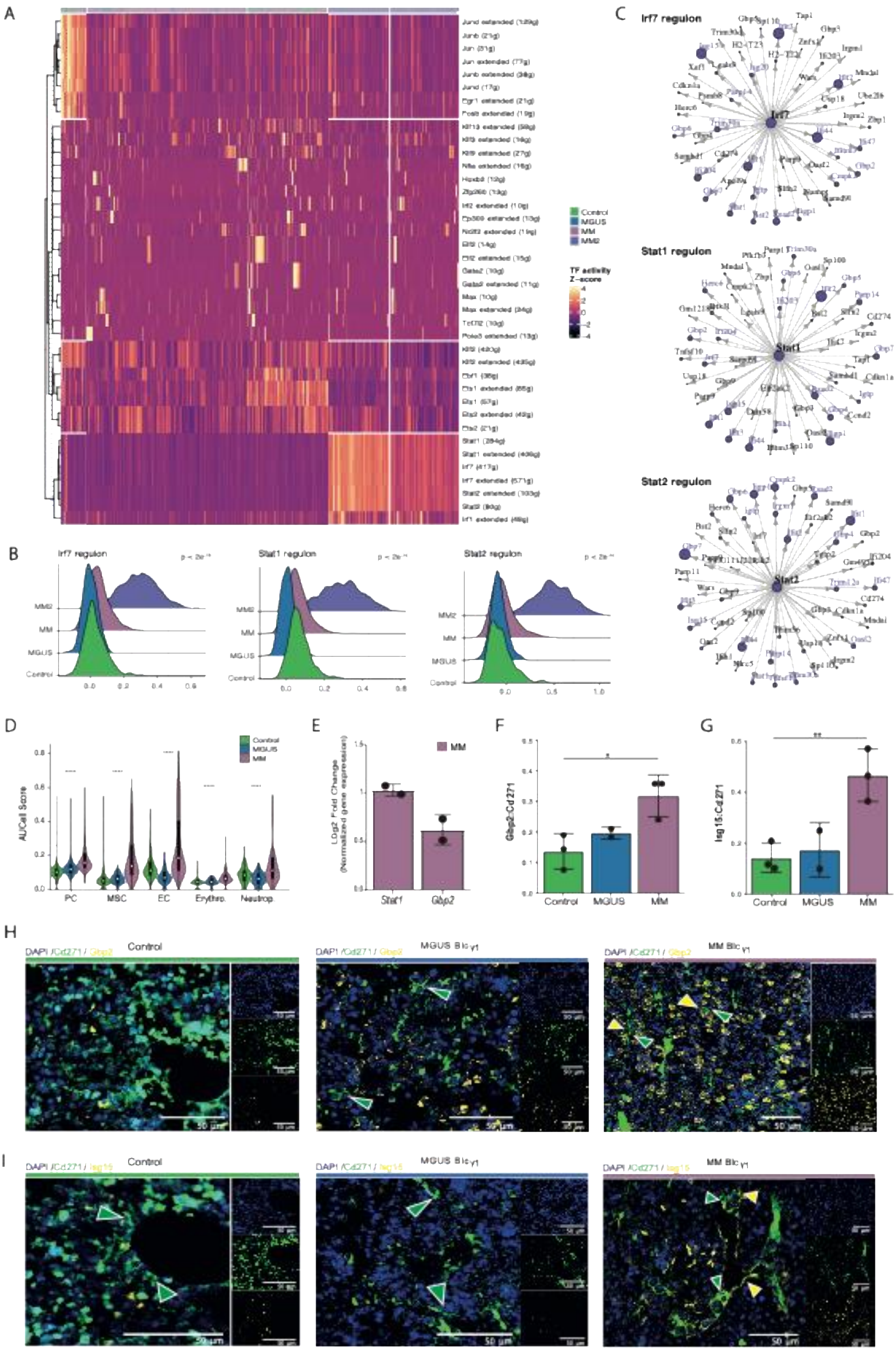

A
B
C
D
E
F
G
H
I
Irf7 regulon
Stat1 regulon
Stat2 regulon
Control
MGUS
MM
MM2
TF activity
Z-score
AUCell Score
PC
MSC
EC
Erythro.
Neutrop.
Log2 Fold Change
(Normalized gene expression)
Stat1
Gbp2
Gbp2:Cd271
Isg15:Cd271
DAPI /Cd271 / Gbp2
DAPI /Cd271 / Isg15
50 µm

Supplemental Figure 4: Additional information on the MM IFN-related signature in the BI$_{c\gamma1}$ model. (A) Heatmap showing the activities of the enriched regulons in EC (Z-score of regulon activity). (B) Density plots showing the score of Irf7, Stat1, and Stat2 regulons in EC (p. = adjusted p-value). (C) Regulon network of Irf7, Stat1, and Stat2 representing the top 50 target genes. (D) Violin plots showing AUC-score for MM IFN-related signature within BM populations in the BI$_{c\gamma1}$ model. (E) Quantitative PCR (qPCR) analysis of the IFN regulon (*Stat1)* and the associated gene (*Gbp2)* in MSC derived from mesenspheres of MM BIcγ1 mice. Gene expression is presented as log$_2$ fold change relative to MSC derived from mesenspheres of control mice. Data were normalized to the reference gene *Gapdh*. N=2 independent biological replicates per group. (F-G) Quantification of the percentage of mesenchymal stromal cells IFN-enriched cells, based on the coexpression of Cd271$^+$ with IFN+ cells (Gbp2, (F); Isg15, (G)) in femurs from Control, MGUS BI$_{c\gamma1}$ and MM BI$_{c\gamma1}$ mice. (H-I) IF staining of MSC (Cd271, green), and IFN response genes markers (Gbp2 (yellow, (H)); Isg15 (yellow, (I))), and nucleus (DAPI) (blue) in FFPE femurs from Control, MGUS BI$_{c\gamma1}$, and MM BI$_{c\gamma1}$ mice. Green arrows point to MSC (Cd271$^+$), and yellow arrows point to IFN$^+$ (Gbp2/Isg15).

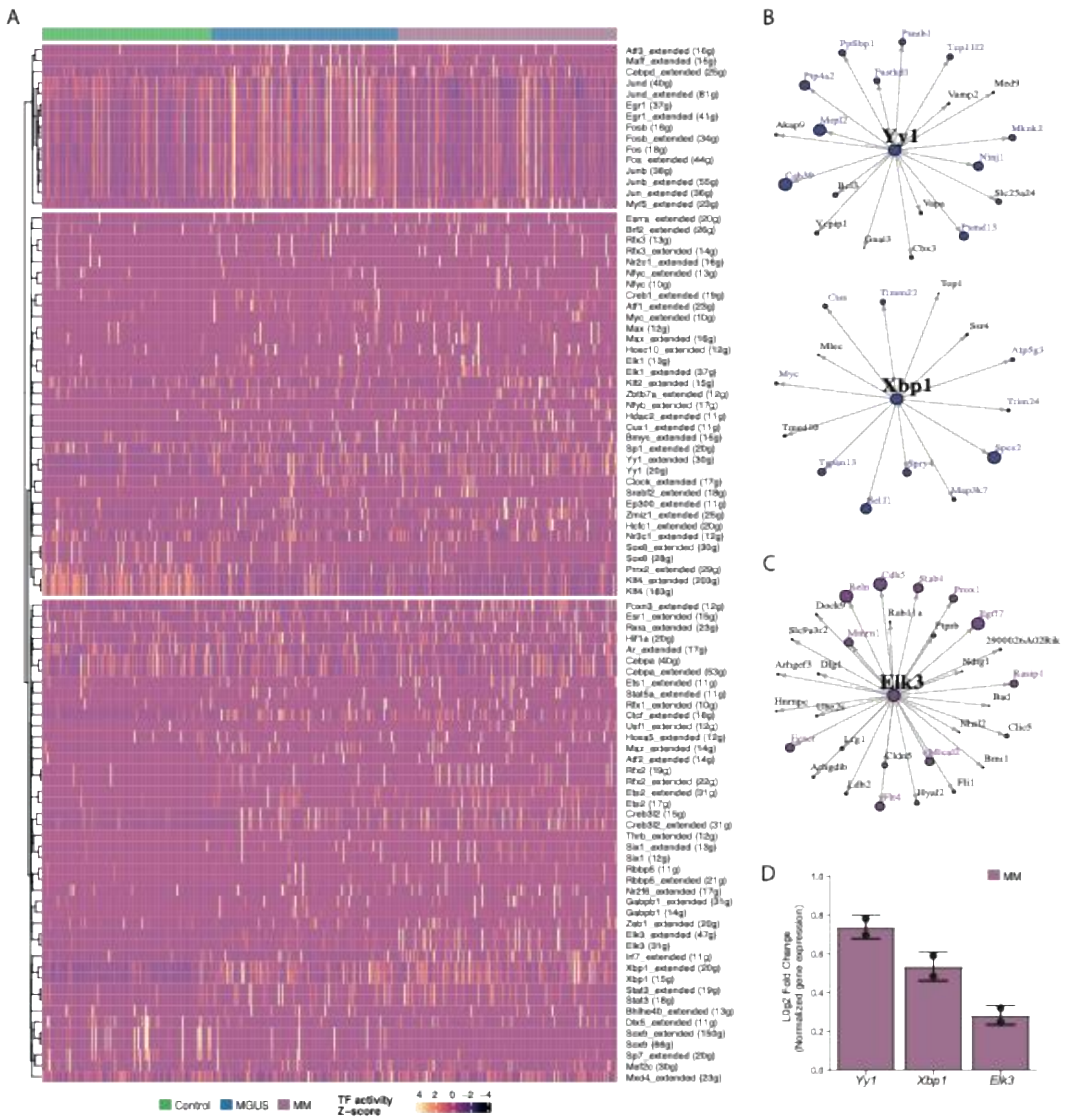

Supplemental Figure 10: Gene regulatory network of MSC during MM progression excluding MM IFN signal. (A) Heatmap showing the activities of the enriched regulons in MSC from control, MGUS, and MM (Z-score of regulon activity). (B) Regulon network of Yy1 and Xbp1, representing the top associated genes (C) Regulon network of Elk3 representing the top associated genes. (D) Quantitative PCR (qPCR) analysis of the enriched regulons (*Yy1*, *Xbp1*, and *Elk3*) in MSC derived from mesenspheres of MM BIcγ1 mice. Gene expression is presented as $\log_2$ fold change relative to MSC derived from mesenspheres of control mice. Data were normalized to the reference gene *Gapdh*. N=2 independent biological replicates per group.

Additionally, as suggested by the reviewer, we validated some of the previously identified interactions in MGUS-BIcγ1 mice and MM BIcγ1 FFPE biopsies by IF. Specifically, we focused on the interactions between the PC-derived ligands *Vegfa* and *C3* and their respective receptors (*Itgb1* and *Lrp1*), expressed by EC and MSC. As expected, these new IF results further validate the interactions predicted by our scRNA-seq analysis.
These results are summarized in Figure 5F, G (see below)
The corresponding results have been incorporated into the Results section (lines 270-275: *To further confirm the interactions identified in MGUS and MM BIcγ1 mice, we performed IF analysis on BM FFPE biopsies* ***(Supplementary Fig. 11E and Fig. 5F, G)****. We observed that the PC-derived ligand Vegfa and the EC/MSC receptor Itgb1 are expressed in close spatial proximity, suggesting functional crosstalk between these cell populations. In contrast, the spatial co-localization of the PC-derived ligand C3 and its corresponding MSC receptor Lrp1 was detected exclusively at the MM stage, thereby validating our previous findings from the scRNA-seq analysis.*
The experimental details have been added to the Methods section:
Lines 206-208:
Antigen retrieval was carried out at 95°C for 30 minutes in either 10 mM citrate buffer (pH 6.0) or, specifically for CD271 staining combinations, in 10 mM Tris-1 mM EDTA buffer (pH 9.0).
Lines 209-212:
For immunofluorescence (IF) staining, the Formalin-Fixed Paraffin-Embedded (FFPE) sections were blocked with BSA 5% for 30 min at RT. The following primary antibodies were used: rat anti-Endomucin antibody (V.7C7) (Santa Cruz Technology 1: 50 dilution), mouse anti-CD271 monoclonal antibody (NGF Receptor) (ME20.4) (Invitrogen, 1: 50 dilution),
Lines 214-218:
VEGF Monoclonal Antibody (JH121) (Invitrogen, 1: 20 dilution), Integrin Beta 1/CD29 Polyclonal antibody (Proteintech, 1: 200 dilution), Complement C3 Polyclonal Antibody (Invitrogen, 1: 100 dilution), Anti-LRP1 antibody (abcam, 1: 50 dilution), CD304 (Neuropilin-1) Recombinant Rabbit Monoclonal Antibody (ST05-30) (Invitrogen, 1: 50 dilution) and Goat anti-GFP (Novus Biologicals, 1:4000).
Lines 222-228:
Goat anti-Rat IgG (H+L) Cross-Adsorbed Secondary Antibody, Alexa Fluor™ 568; Goat anti-Mouse IgG (H+L) Highly Cross-Adsorbed Secondary Antibody, Alexa Fluor™ Plus 488; Goat anti-Rabbit IgG (H+L) Cross-Adsorbed Secondary Antibody, Alexa Fluor™ 488, Goat anti-Mouse IgG (H+L) Cross-Adsorbed Secondary Antibody, Alexa Fluor™ 568 (Invitrogen), Donkey anti-Mouse IgG (H+L) Highly Cross-Adsorbed Secondary Antibody, Alexa Fluor™ 555 (Invitrogen), Donkey anti-Goat IgG (H+L) Cross-Adsorbed Secondary Antibody, Alexa Fluor™ 488 (Invitrogen).

FIGURE 5

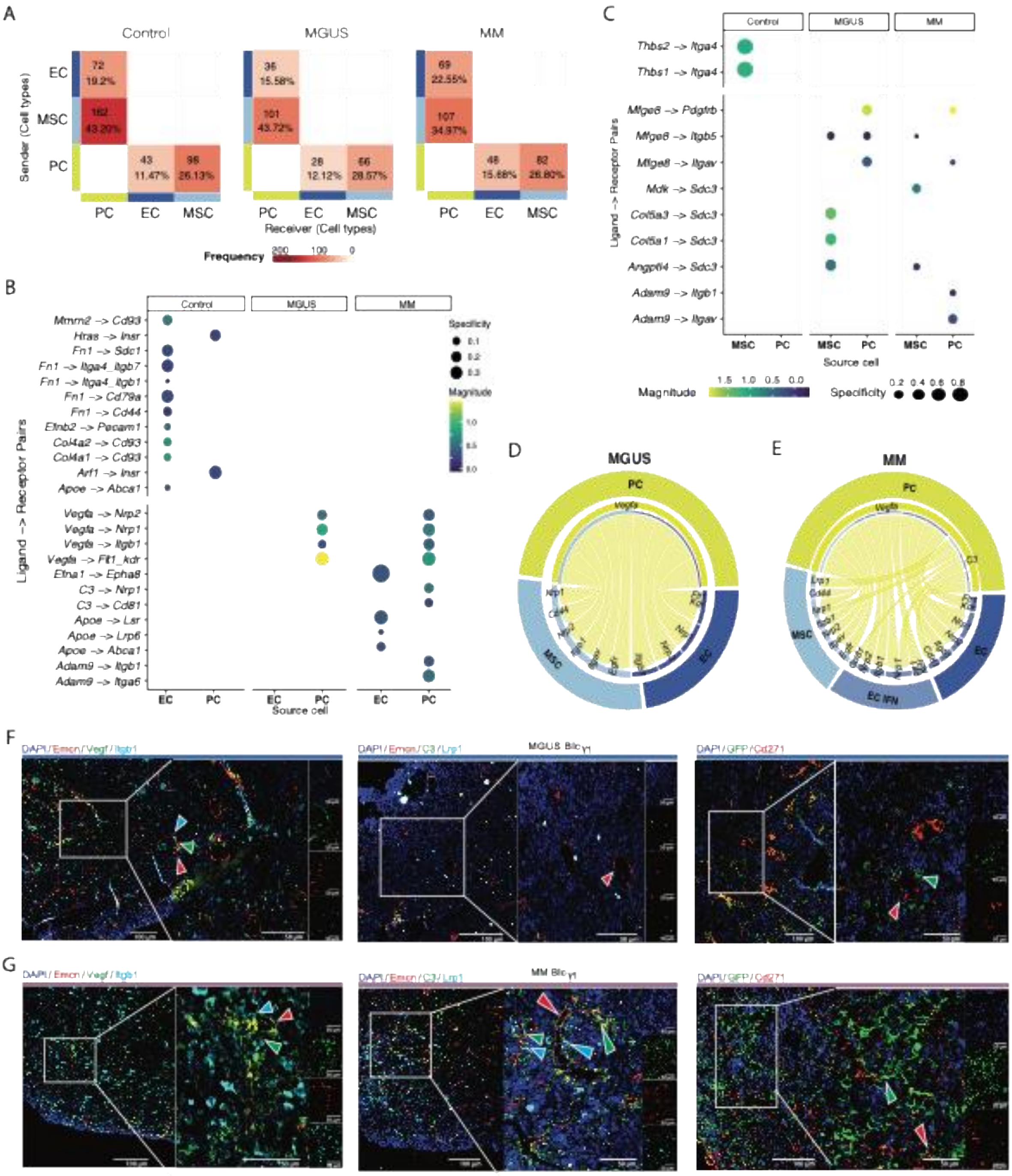

Fig. 5. Altered crosstalk between EC and MSC with malignant PC is potentially associated with myeloma development (A) Heatmap showing the total number and directionality of predicted L–R interactions between PC and niche populations (EC and MSC) across disease stages. Rows represent sender cell types and columns represent receiver cell types. Colors are proportional to the number of ligand-receptor pairs. (B-C) Dot plots of representative ligand-receptor interactions between PC and EC (B) or MSC (C) during disease progression. Dot size and color indicate the interaction's specificity and strength, respectively. (D-E) Chord diagrams illustrating L-R pairs shared between PC–EC and PC–MSC communication within MGUS (D) and MM (E) stages. Colors and width represent signal senders and strength, respectively. PC-derived *Vegfa* engages multiple receptors in both stages (D-E), while *C3*-mediated interactions emerge in MM (E). (F-G) IF staining of PC–EC/MSC interactions: PC-derived ligands Vegf (left panel, green) and C3 (middle panel, green) are shown in PC (GFP+, green, right panel). The corresponding receptors Itgb1 (blue, left panel) and Lrp1 (blue, middle panel) are detected in EC (Emcn+, red, left and middle panels) and MSC

(Cd271^{+}, red, right panel). Stainings were performed on FFPE femur sections from MGUS BIcγ1 (F) and MM BIcγ1 mice (G).

Moreover, to functionally validate the MM-associated interactions identified in the MM MIcγ1 mice, we performed coculture experiments using MM-PC (MM5080[8]) and MSC- mesenspheres[10] derived from MM MIcγ1 mice. We specifically examined the interaction between the PC-derived ligand *Vegfa* and its receptors *Itgb1* and *Nrp1* expressed by MSC by Proximity ligation assay (Navinci Kit) analysis and conventional IF.
These results confirmed the predicted interactions identified by our scRNA-seq analysis.

The corresponding results have been incorporated into the Results section, lines 276-284:
*In addition, we investigated cell–cell interactions in the MIcγ1 mouse model (**Supplementary Table 8**). Consistent with our previous findings, we detected pro-angiogenic signaling in the MM stage of this model, characterized by Vegfa secretion from PC and its interaction with multiple receptors expressed by EC and MSC (**Supplementary Fig. 12A**). To functionally validate the MM-associated interactions identified in MM MIcγ1 mice, we performed coculture experiments using MM-PC (MM5080[15]) and MSC-mesenspheres[21] derived from MM MIcγ1 mice. We specifically assessed the interaction between the PC-derived ligand Vegfa and its MSC-expressed receptors Itgb1 and Nrp1 using a proximity ligation assay (Navinci Kit) (**Supplementary Fig. 12B, C**), as well as conventional IF (**Supplementary Fig. 12D, E**). These experiments are consistent with the interactions predicted by our scRNA-seq analysis.*
These results are summarized in new Supplementary Figure 12 (see below), and the experimental details have been added to the Methods section…

Lines 206-208:
Antigen retrieval was carried out at 95°C for 30 minutes in either 10 mM citrate buffer (pH 6.0) or, specifically for CD271 staining combinations, in 10 mM Tris-1 mM EDTA buffer (pH 9.0).
Lines 209-212:
For immunofluorescence (IF) staining, the Formalin-Fixed Paraffin-Embedded (FFPE) sections were blocked with BSA 5% for 30 min at RT. The following primary antibodies were used: rat anti-Endomucin antibody (V.7C7) (Santa Cruz Technology 1: 50 dilution), mouse anti-CD271 monoclonal antibody (NGF Receptor) (ME20.4) (Invitrogen, 1: 50 dilution),
Lines 214-218:
VEGF Monoclonal Antibody (JH121) (Invitrogen, 1: 20 dilution), Integrin Beta 1/CD29 Polyclonal antibody (Proteintech, 1: 200 dilution), Complement C3 Polyclonal Antibody (Invitrogen, 1: 100 dilution), Anti-LRP1 antibody (abcam, 1: 50 dilution), CD304 (Neuropilin-1) Recombinant Rabbit Monoclonal Antibody (ST05-30) (Invitrogen, 1: 50 dilution) and Goat anti-GFP (Novus Biologicals, 1:4000).
Lines 222-228:
Goat anti-Rat IgG (H+L) Cross-Adsorbed Secondary Antibody, Alexa Fluor™ 568; Goat anti-Mouse IgG (H+L) Highly Cross-Adsorbed Secondary Antibody, Alexa Fluor™ Plus 488; Goat anti-Rabbit IgG (H+L) Cross-Adsorbed Secondary Antibody, Alexa Fluor™ 488, Goat anti-Mouse IgG (H+L) Cross-Adsorbed Secondary Antibody, Alexa Fluor™ 568 (Invitrogen), Donkey anti-Mouse IgG (H+L) Highly Cross-Adsorbed Secondary Antibody, Alexa Fluor™ 555 (Invitrogen), Donkey anti-Goat IgG (H+L) Cross-Adsorbed Secondary Antibody, Alexa Fluor™ 488 (Invitrogen).
Lines 232-265:
Mesensphere isolation and culture
Mesenspheres were generated from mouse primary BM cells as previously described by Forte et al.[10] Clean mouse bones were crushed in a mortar with 2 ml of 0.25% Collagenase Type I (Stem Cell Technologies). The resulting suspension was incubated for 45 minutes at 37 °C in agitation. The enzymatic reaction was quenched with PBS+ 2% FBS, and cells were filtered through a 40 µm cell strainer. The erythrocytes in the cell suspension were lysed by incubation for 10 minutes with RBC Lysis Buffer at RT. Then, erythroid and hematopoietic lineages were magnetically depleted. Therefore, cells were incubated with biotin-conjugated primary antibodies against Cd45 and Ter119 (BD

Biosciences, 1:100) followed by incubation with streptavidin-conjugated magnetic beads (BD Biosciences). The negative fraction was plated at low density (<500,000 cells/cm2) in ultralow-adherence 35 mm dishes (StemCell Technologies) previously coated with Poly-Hema (Sigma). The growth medium for spheres contained 0.1 mM β-mercaptoethanol; 1% nonessential amino acids (Sigma); 1% N_2 and 2% B27 supplements (Invitrogen); recombinant human fibroblast growth factor (FGF)-basic, recombinant human epidermal growth factor (EGF), recombinant human platelet-derived growth factor (PDGF-AA), recombinant human oncostatin M (227 aa OSM) and recombinant human IGF-1 in Dulbecco's modified Eagle's medium (DMEM)/F12 (1:1) / human endothelial (1:2) serum-free medium (Invitrogen). All cytokines (Preprotech) were added at a final concentration of 20 ng/ml, except for IGF-1, which was added at 40 ng/ml. Additionally, mesenspheres medium was supplemented with 15% chicken embryo extract prepared as described previously[11]. Fertilized chicken eggs were incubated for 11 days at 38°C in a humidified incubator. Embryos were washed with DMEM (Invitrogen), macerated by passage through a 50 ml syringe, and diluted 1:1 in the same medium. Hyaluronidase (2 mg, Sigma) was added to each 50 ml tube and incubated for 1h at 4°C with agitation. Following 6 h ultracentrifugation ($3x10^4$ g) at 4°C was decanted and filtered with 0.45 μm and 0.22 μm sterile filters (Millipore). Aliquots were stored at -80°C until use. The cultures were incubated at 37°C with 5% CO_2, 20% O_2 in a water-jacketed incubator and left untouched for 1 week. Afterwards, half-medium changes were performed twice a week. Following a 14-day incubation period, the resulting spheres were harvested for downstream applications, including RNA isolation and co-culture experiments. Cell cultures were incubated at 37°C with 5% CO_2. After 1 week without medium changes, half-medium changes were performed twice weekly. After 2 weeks of culture, mature spheres were harvested for RNA isolation or co-culture assays. The mesenspheres used in this paper were obtained from control mice (4 months old) and MM mice. ($BIc_{\gamma 1}$ and $MIc_{\gamma 1}$, 6–12 months old). Tumor burden was quantified by flow cytometry as the percentage of PC (GFP^+) within the total BM population. For all MM-derived MSC mesenspheres cultures, mice were selected based on a confirmed tumor burden of 3%-8% PC. For each independent experiment, mesenspheres were generated from a pool of BM cells harvested from three mice.

Lines 292-314:

Coculture and cell-cell interaction imaging

To evaluate interactions between MM-PC and MSC, BM mesenspheres derived from MM $MIc_{\gamma 1}$ mice were co-cultured with the murine MM cell line MM5080[8]. This cell line was previously established from an original MM developed in P53-BIcγ1 mice. For imaging experiments $7,5x10^4$ PC were co-cultured with 60 mesenspheres in a μ-Slide 8 Well ^{high} ibiTreat (Ibidi). Cultures were incubated for 24 hours at 37°C in a 5% CO_2 water-jacketed incubator. Following incubation, adherent spheres were washed with PBS to remove non-adherent cells and fixed with formol 4% (PanReac) for 20 minutes at RT. Fixed cells were incubated in a blocking buffer (PBS containing 5% BSA and 0.1% Tween-20) for 1 hour at RT. Primary antibodies against Vegf (VEGF Mouse monoclonal Antibody (JH121) (Invitrogen, 1: 20 dilution)), Itgb1 (Integrin Beta 1/CD29 Rabbit polyclonal antibody (Proteintech, 1: 200 dilution)), and Nrp1(CD304 (Neuropilin-1) Recombinant rabbit monoclonal antibody (ST05-30)) were diluted in PBS-0.1% Tween-20 and incubated overnight at 4°C. At this point, we performed a standard IF and a proximity ligation assay (PLA). For IF samples were incubated with the following secondary antibodies (1:200 dilution): Goat anti-Rabbit IgG (H+L) Highly Cross-Adsorbed Secondary Antibody, Alexa Fluor™ 647 and Goat anti-Mouse IgG (H+L) Highly Cross-Adsorbed Secondary Antibody, Alexa Fluor™ Plus 488 (both from Invitrogen). Nuclei were counterstained with DAPI (Invitrogen; 1:1000). On the other hand, to assess the proximity of specific ligand-receptor pairs, the NaveniFlex Cell Atto647N kit (Navinci) was used according to the manufacturer's instructions. This assay utilized the previously bound mouse and rabbit primary antibodies (Vegf-Itgb1 / Vegf-Nrp1) to detect specific ligand-receptor interactions. Images were recorded with a Confocal Scanning Laser Microscope (Zeiss LSM 800).

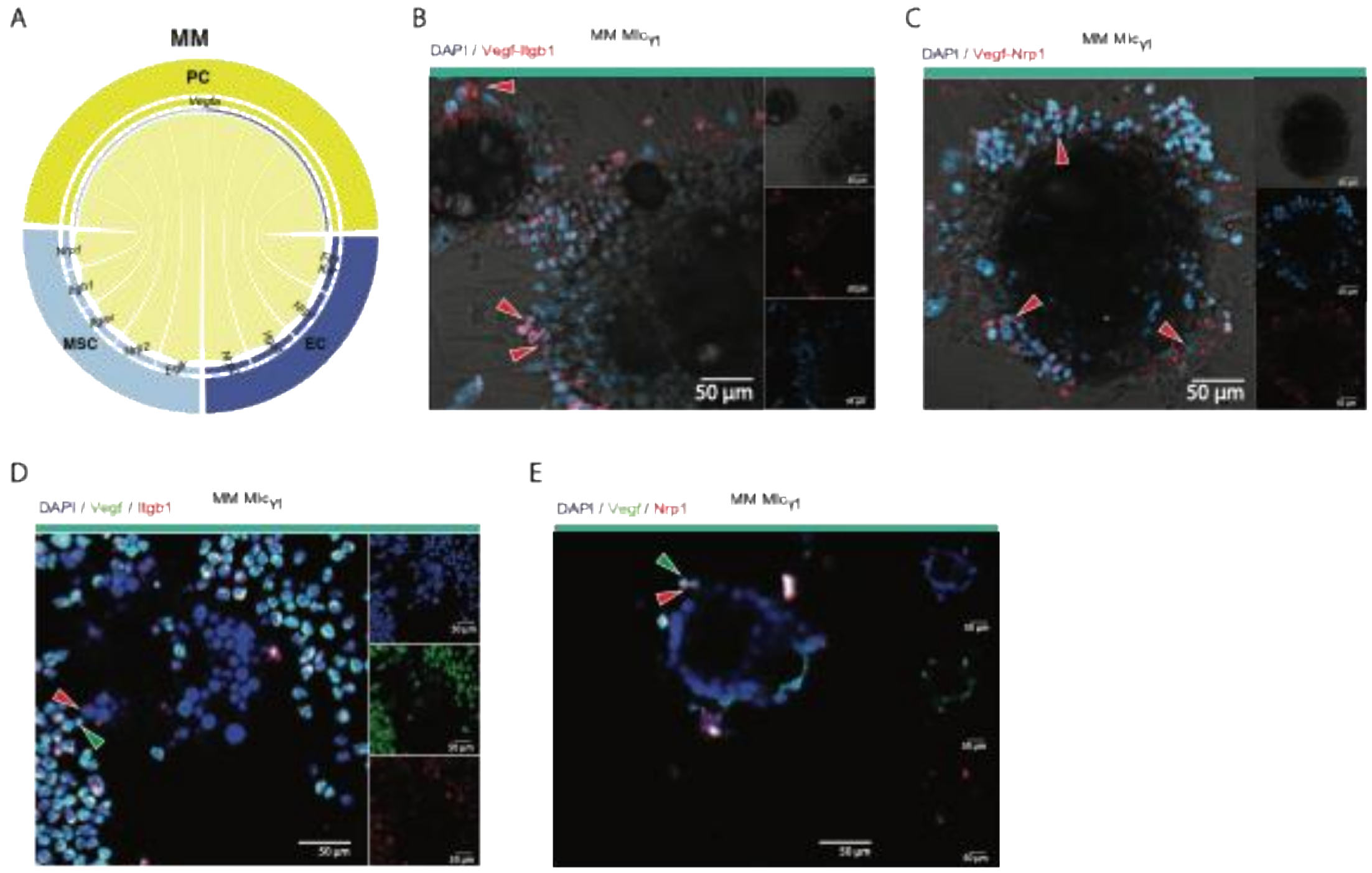

Supplemental Figure 12. MM-specific interactions among EC, MSC, and PC in MM BIcγ1 mice and functional validation assays. (A) Chord diagrams illustrating L-R pairs shared between PC–EC and PC–MSC communication within the MM stage from the MM MIcγ1 mouse model. Colors and width represent signal senders and strength, respectively. PC-derived *Vegfa* engages multiple receptors in both cell types. (B-C) Proximity ligation assay (Navinci Kit) analysis of MM MIcγ1 interactions (Vegf-Itgb1 (B) and Vegf-Nrp1 (C)) in coculture of PC (cell line MM5080) with MSC derived from mesenspheres of MM MIcγ1 mice. Representative confocal images show PLA-positive puncta (red arrows), indicating protein–protein interactions, with nuclei counterstained with DAPI (blue). (D-E) IF staining of PC ligands (Vegf (green)), with MSC-receptors (Itgb1 (red (D)) and Nrp1 (red (E)), and nucleus (DAPI) (blue) in coculture of PC (cell line MM5080) with MSC derived from mesenspheres of MM MIcγ1 mice. Green arrows point to PC-Vegf$^+$, and red arrows point to MSC-Itgb1$^+$ (D) or Nrp1$^+$ (E).

2. What is the relevance of a "subset" of MM patients being defined like a more interferon-like response in the BME? Do they have different prognosis or response to therapy? Do they correspond to a specific genomic subtype (say high risk disease)? This would be very useful to comment on if any relevant clinical data is available. And, particularly if clinical data is available but this IFN-like signature does not carry any clinical prognostic relevance, why not?

We thank the reviewer for this insightful comment. Our team also carefully considered the importance of these results.
First, we did not identify any association between specific genomic subtypes and the presence of an IFN signature (high or low) in our MM patient cohort, nor in publicly available data from de Jong et al[1].
Regarding prognosis, we were likewise interested in the translational relevance of our findings. To this end, we consulted with our collaborators to determine the disease status of the patients included in our RNA-seq study (Fig. 7). Unfortunately, complete clinical data were not available for all patients. Nevertheless, using the available cases, we examined the potential association between the presence of an IFN signature in stromal cells (stratified as high versus low) and clinical outcome. After careful analysis, we were unable to identify a significant correlation between stromal IFN signature status and MM disease progression in this cohort (see tables below). We acknowledge that the limited sample size likely restricts the statistical power of this analysis, and that evaluation in a larger cohort will be necessary to draw definitive conclusions.

Given these limitations and the absence of a clear association, we considered it premature to include these data in the revised main manuscript. However, if the reviewer believes it important, we would be happy to include these details.

Sample	Gender	Age	ant medical hi	BM sample	% PC	Del17p	Amp1q	t(4;14)	Number of sorted cells	High IFN-Signature	Progression free survival (months)	Overall survival (months)
MM_23	MALE	69	MM	Aspirate	10,0%	NO	NO	NO	184	NO	2,6	55,5
MM_24	MALE	78	MM	Aspirate	40,0%	NO	NO	NO	596	NO	no data	no data
**MM_1**	**FEMALE**	**76**	**MM**	**Aspirate**	**45,0%**	**NO**	**NO**	**NO**	**133**	**YES**	**no data**	**no data**
MM_3	FEMALE	79	MM	Aspirate	7,0%	NO	NO	NO	614	NO	52,4	52,4
MM_4	FEMALE	39	MM	Aspirate	16,7%	NO	YES	NO	344	NO	6,5	9,6
**MM_7**	**MALE**	**73**	**MM**	**Aspirate**	**3,6%**	**NO**	**NO**	**NO**	**966**	**YES**	**64,3**	**64,3**
MM_6	MALE	74	MM	Aspirate	33,5%	NO	NO	NO	347	NO	61,1	61,1
**MM_11**	**FEMALE**	**82**	**MM**	**Aspirate**	**51,0%**	**NO**	**YES**	**NO**	**161**	**YES**	**31,8**	**31,8**
**MM_16**	**FEMALE**	**76**	**MM**	**Aspirate**	**19,6%**	**NO**	**NO**	**NO**	**425**	**YES**	**66,4**	**66,4**
MM_13	MALE	78	MM	Aspirate	6,7%	NO	YES	NO	105	NO	no data	no data

Sample	Gender	Age	BM sample	Relevant medical history	High IFN-Signature	Progression free survival (months)	Overall survival (months)
hMM_1	FEMALE	56	bone cylinder	MM	No	46,17	46,17
hMM_2	MALE	70	bone cylinder	MM	No	111,73	111,73
hMM_3	MALE	52	bone cylinder	MM	Yes	13,07	13,07
hMM_4	MALE	70	bone cylinder	MM	Yes	no data	no data
hMM_5	FEMALE	54	bone cylinder	MM	Yes	62,37	74,23
hMM_6	FEMALE	54	bone cylinder	MM	Yes	261,83	261,83

The information presented in this table is included in Supplementary Table 2 (BM aspirate samples and BM biopsies), except for the values highlighted in red, which correspond to prognostic data.

3. Overall the immunohistochemistry images do not provide much information, at least in the way I can visualize it in the submitted manuscript. It would be very beneficial to have some quantification of co-localized staining to more clearly show differences in the IFN-high versus IFN-low conditions.

We appreciate the reviewer for bringing this point to our attention. **Accordingly**, **we have quantified not only the human samples but also the mouse immunofluorescence (IF) experiments.** Where possible, we have also increased the number of samples analyzed (control mice, MM BIcγ1 and MM MIcγ1 , as well as healthy human samples (HD)).
The resulting data have been incorporated into the revised manuscript (Figs. 2I, J and 6D, and Supplementary Figs. 4F–G for the mouse data; and Figs. 7G and 7I for the human data).
In addition, in Figs. 7G and 7I we separately present high and low expression patient groups, highlighting the differences between these two patient subsets. These changes are reflected in supplementary material in lines 451-452: *MM samples were stratified into 'IFN-high' and 'IFN-low' groups using the 80th percentile as the discriminatory threshold.*

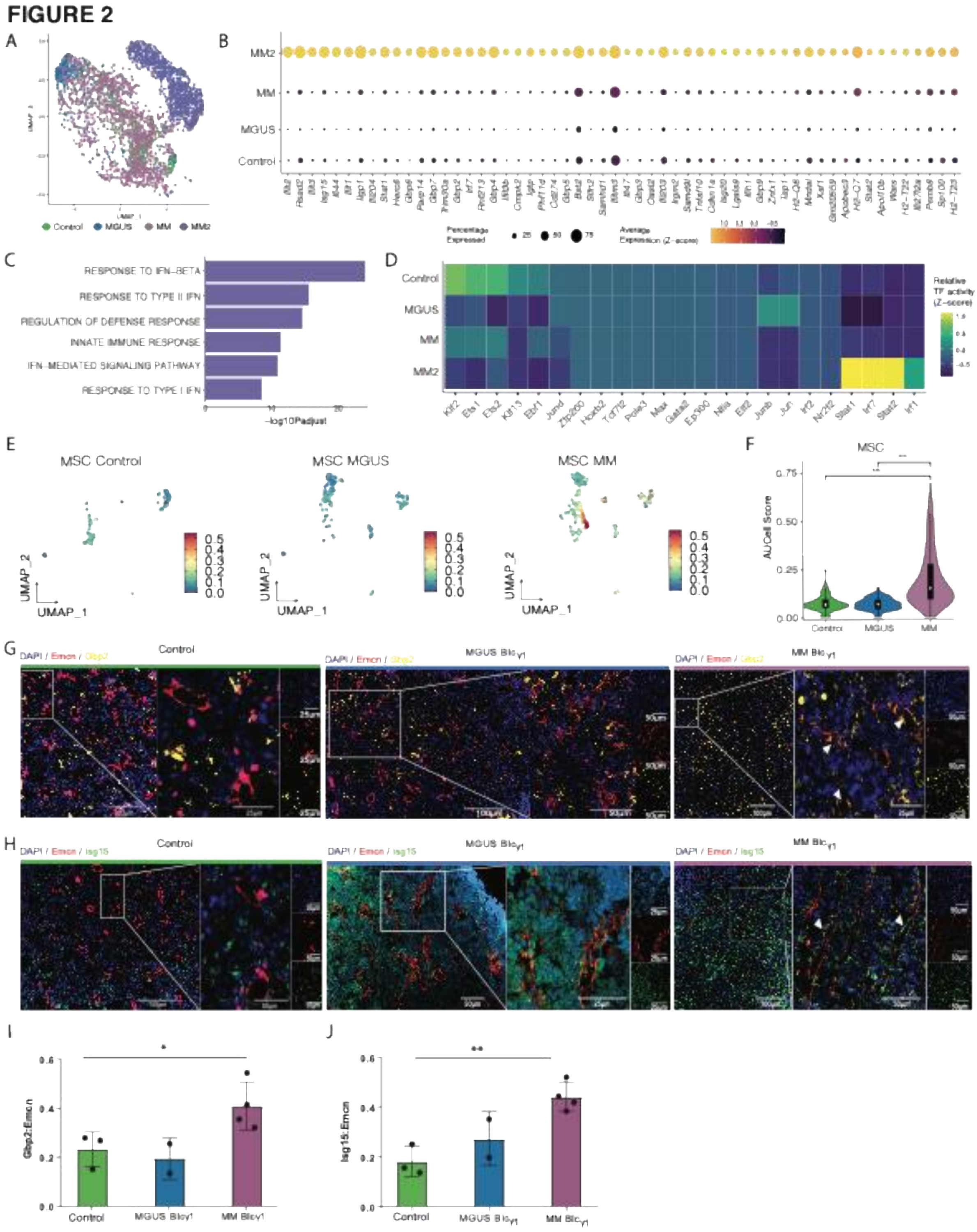

Fig. 2. Identification of a MM-specific IFN signature in the BME.
(A) UMAP dimension reduction of EC revealing a transcriptionally distinct population in MM. (B) Dot plot visualization of the markers defining EC MM2 cluster. Average gene expression is shown by color intensity, and the percentage of cells expressing each gene is shown by size. (C) Bar charts of enriched GO terms from ORA (adjusted p. value) computed using MM2 cluster markers. The horizontal axis represents the negative of the base 10 logarithm of the adjusted p. value. (D) Heatmap representing z-scaled mean regulon activity score of inferred top specific regulons for each stage. (E) UMAP plot illustrating the distribution of the IFN-related signature in MSC across control, MGUS, and MM. (F) Violin plots showing AUC-score for IFN-

related signature within MSC in control, MGUS and MM cells. (G-H) Immunofluorescence (IF) staining of EC (Emcn, red), IFN response genes markers of the MM2 cluster (Gbp2, yellow (G); Isg15, green (H)), and nucleus (DAPI) (blue) in Formalin-Fixed Paraffin-Embedded (FFPE) femurs from Control (YFP$_{c\gamma1}$), MGUS BI$_{c\gamma1}$ and MM BI$_{c\gamma1}$. White arrows point EC Ecmn^{+} IFN^{+} (Gbp2 (G)/Isg15 (H)) in MM BI$_{c\gamma1}$. (I-J) Quantification of the percentage of endothelial IFN-enriched cells, based on the coexpression of Emcn with IFN+ cells (Gbp2, (I); Isg15, (J)) in femurs from Control (YFP$_{c\gamma1}$), MGUS BI$_{c\gamma1}$ and MM BI$_{c\gamma1}$ mice.

FIGURE 6

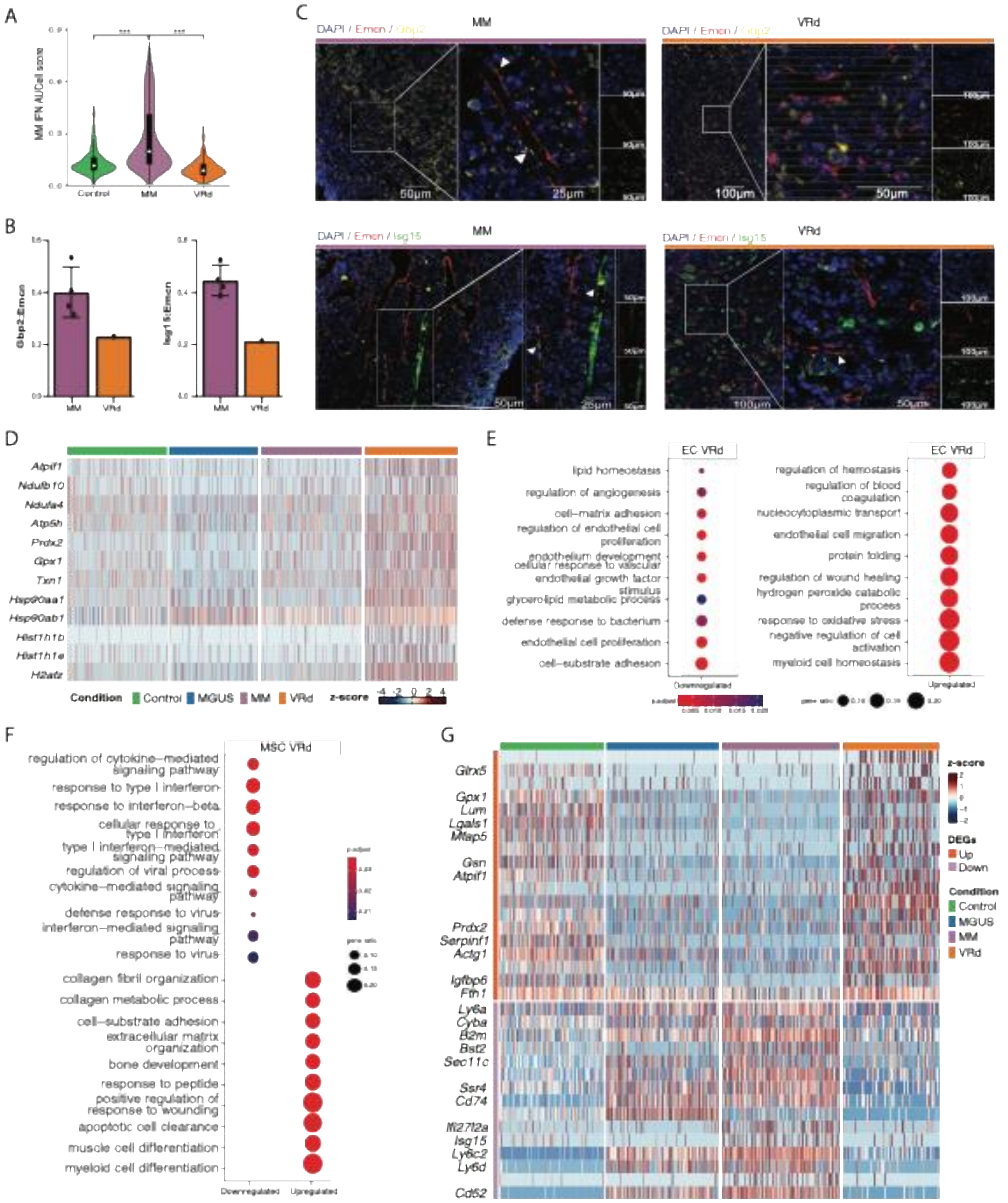

Fig. 6. VRd induces transcriptional remodeling of EC and MSC in MM.

(A) Violin plot showing AUC-score for IFN-related signature in EC from Control (YFP$_{c\gamma1}$), MM BIc$_{\gamma1}$ (MM) and MM BI$_{c\gamma1}$-*Crbn*^{I391V} VRd-treated (VRd) mice. (B) Quantification of the percentage of endothelial IFN^{+} cells, determined by the coexpression of Emcn with IFN response genes (Gbp2 (left plot) and Isg15 (right

plot) in femurs from MM $BIc_{\gamma1}$ (MM) and MM $BI_{c\gamma1}$-*Crbn*^{I391V} VRd-treated (VRd) mice. (C) IF staining of EC (Emcn, red), IFN response genes (Gbp2 (yellow, (upper plot)); Isg15 (green, (bottom plot)), and nucleus (DAPI) (blue) in FFPE femurs from MM $BI_{c\gamma1}$ mice (MM) and VRd-treated mice (VRd). White arrows point EC (Ecmn^{+}) IFN^{+} (Gbp2/Isg15). (D) Heatmap showing the expression of stress-related DEGs identified in the comparison between treated and non-treated non-IFN MM EC at different stages of the disease. Color represents the z-score scaled expression values. (E) Significant terms derived from ORA between VRd-treated and untreated non-IFN MM EC. (F) Significant terms derived from ORA of MSC from MM mice before and after VRd treatment. (G) Heatmap projecting the expression of DEGs identified in the comparison between non-treated and treated MSC at different stages of the disease. Color represents the z-score scaled expression values.

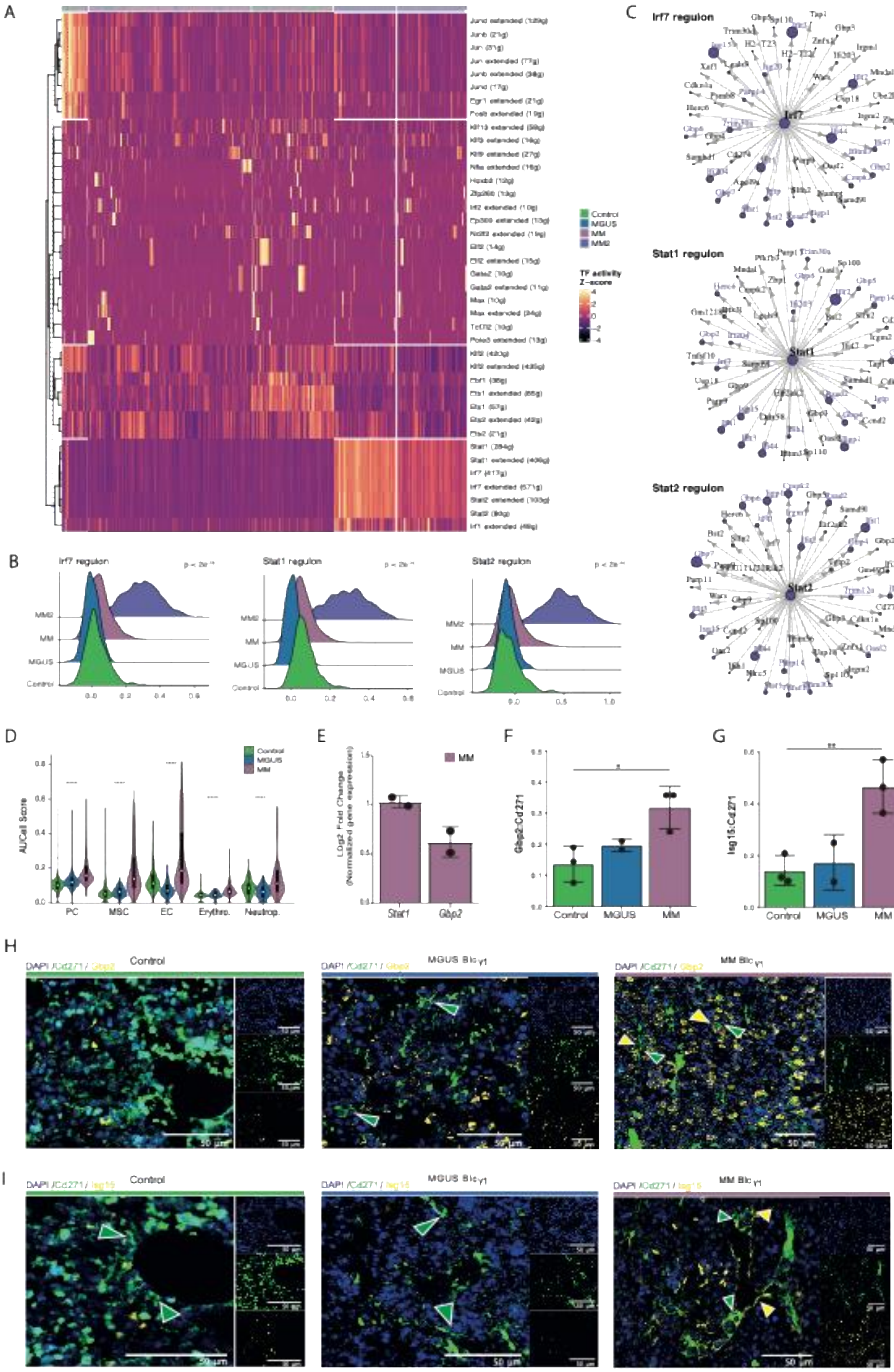
A
B
C
D
E
F
G
H
I
Control
MGUS
MM
MM2
TF activity Z-score
Irf7 regulon
Stat1 regulon
Stat2 regulon
AUCell Score
PC
MSC
EC
Erythro.
Neutrop.
Log2 Fold Change (Normalized gene expression)
Stat1
Gbp2
Gbp2:Cd271
Isg15:Cd271
DAPI /Cd271 / Gbp2
DAPI /Cd271 / Isg15
MGUS Blc γ1
MM Blc γ1
50 µm

Supplemental Figure 4: Additional information on the MM IFN-related signature in the $BI_{c\gamma 1}$ model. (A) Heatmap showing the activities of the enriched regulons in EC (Z-score of regulon activity). (B) Density plots showing the score of Irf7, Stat1, and Stat2 regulons in EC (p. = adjusted p-value). (C) Regulon network of Irf7, Stat1, and Stat2 representing the top 50 target genes. (D) Violin plots showing AUC-score for MM IFN-related signature within BM populations in the $BI_{c\gamma 1}$ model. (E) Quantitative PCR (qPCR) analysis of the IFN regulon (*Stat1)* and the associated gene (*Gbp2)* in MSC derived from mesenspheres of MM BIcγ1 mice. Gene expression is presented as $\log_2$ fold change relative to MSC derived from mesenspheres of control mice. Data were normalized to the reference gene *Gapdh*. N=2 independent biological replicates per group. (F-G) Quantification of the percentage of mesenchymal stromal cells IFN-enriched cells, based on the coexpression of $Cd271^+$ with IFN+ cells (Gbp2, (F); Isg15, (G)) in femurs from Control, MGUS $BI_{c\gamma 1}$ and MM $BI_{c\gamma 1}$ mice. (H-I) IF staining of MSC (Cd271, green), and IFN response genes markers (Gbp2 (yellow, (H)); Isg15 (yellow, (I))), and nucleus (DAPI) (blue) in FFPE femurs from Control, MGUS $BI_{c\gamma 1}$, and MM $BI_{c\gamma 1}$ mice. Green arrows point to MSC ($Cd271^+$), and yellow arrows point to IFN^+ (Gbp2/Isg15).

FIGURE 7

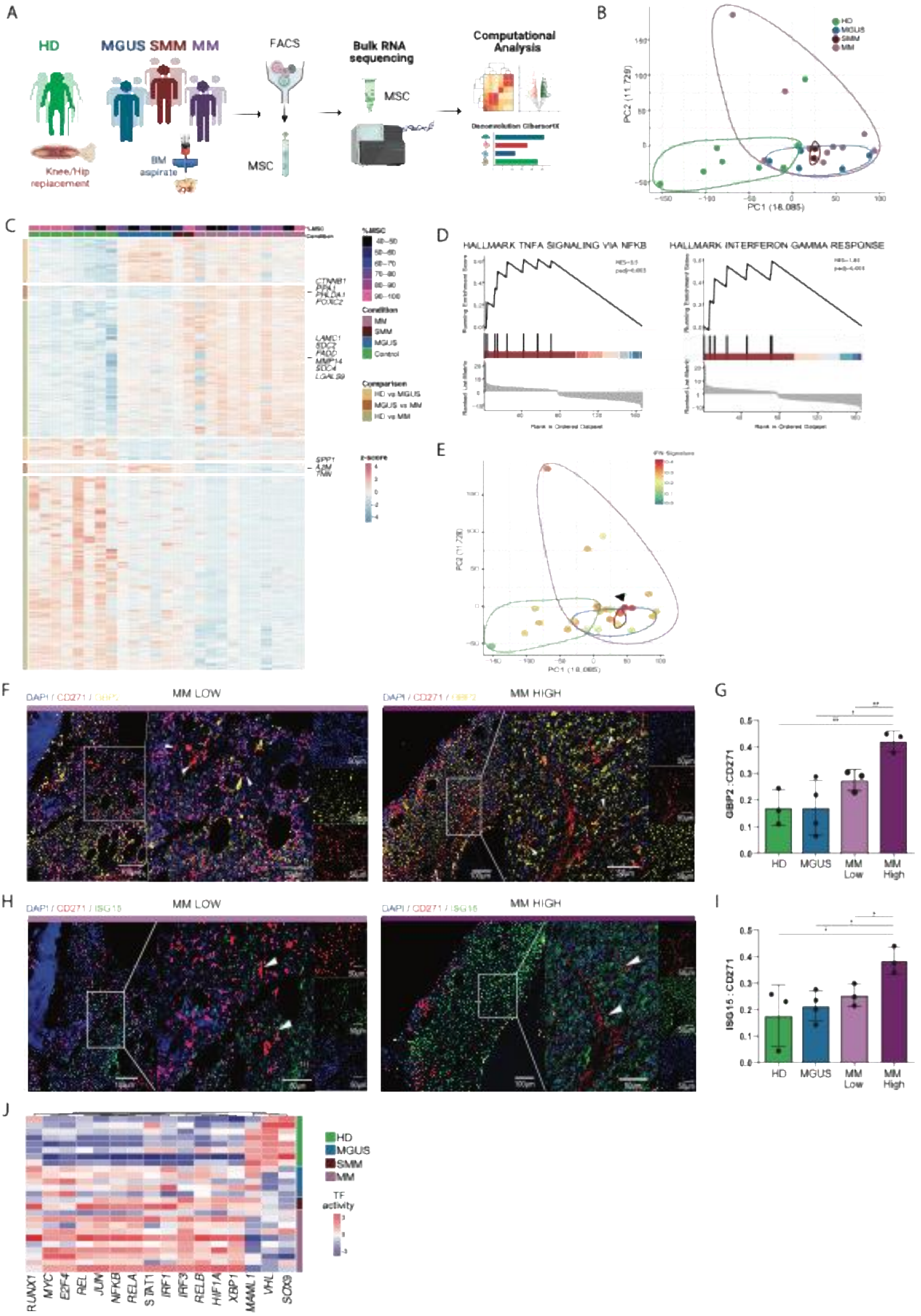

A
HD
MGUS SMM MM
Knee/Hip replacement
BM aspirate
FACS
MSC
Bulk RNA sequencing
MSC
Computational Analysis
Deconvolution CibersortX
B
HD
MGUS
SMM
MM
PC1 (18.085)
PC2 (11.729)
C
CTNNB1
RPA1
PHLDA1
FOXC2
LAMC1
SDC2
PADD
MMP14
SDC4
LGALS9
SPP1
A2M
TNN
%MSC
Condition
MM
SMM
MGUS
Control
Comparison
HD vs MGUS
MGUS vs MM
HD vs MM
z-score
D
HALLMARK TNFA SIGNALING VIA NFKB
HALLMARK INTERFERON GAMMA RESPONSE
Rank in Ordered Dataset
E
IFN Signature
PC1 (18.085)
PC2 (11.729)
F
DAPI / CD271 / GBP2
MM LOW
MM HIGH
G
GBP2 : CD271
HD
MGUS
MM Low
MM High
H
DAPI / CD271 / ISG15
MM LOW
MM HIGH
I
ISG15 : CD271
HD
MGUS
MM Low
MM High
J
HD
MGUS
SMM
MM
TF activity
RUNX1
MYC
E2F4
REL
JUN
NFKB
RELA
STAT1
IRF1
IRF3
RELB
HIF1A
XBP1
MAML1
VHL
SOX9

Fig. 7. Transcriptional dynamics of human MSC during MM progression.

(A) Experimental workflow used for the isolation and bulk RNA-seq analysis of human BM MSC. (B) PCA of MSC expression profiles shows clustering by disease stage. The color of the dots represents disease stages. (C) Heatmap showing the expression profiles of DEGs identified in each pairwise comparison across disease progression. (D) GSEA plots for the gene sets 'TNFα signaling via NFκB' (left panel) and 'Interferon gamma response' (right panel) in the comparison between healthy donors (HD)- MSC and MM-MSC. (E) PCA plot showing the MM-IFN signature score in each human sample, previously identified murine BM MM cells. (F) IF staining of MSC (CD271, red), IFN response genes (*GBP2*, yellow), and nucleus (DAPI) (blue) in archived paraffin-embedded human BM biopsies of MM patients with low (MM LOW) and high IFN signal (MM HIGH) in stromal cells (see Supplementary Table 2). White arrows point to MSC ($CD271^{+}$) in MM LOW IFN samples, and MSC ($CD271^{+}$) IFN^{+} (GBP2) in MM HIGH IFN samples. (G) Quantification of the percentage of mesenchymal IFN^{+} cells, determined by the coexpression of $CD271^{+}$ with IFN response genes (*GBP2*) in FFPE biopsies from HD (three donors) and archived paraffined human BM biopsies of MGUS (four patients) and MM patients (six patients with low (three) and high (three) IFN signal in stromal cells). (H) IF staining of MSC (CD271, red), IFN response genes (*ISG15*, green), and nucleus (DAPI) (blue) in archived paraffin-embedded human BM biopsies of MM patients with low (MM LOW) and high IFN signal (MM HIGH) in stromal cells (see Supplementary Table 2). White arrows point to MSC ($CD271^{+}$) in MM LOW IFN samples, and MSC ($CD271^{+}$) IFN^{+} (ISG15) in MM HIGH IFN samples. (I) Quantification of the percentage of mesenchymal IFN^{+} cells, determined by the coexpression of $CD271^{+}$ with IFN response genes (*ISG15*) in FFPE biopsies from HD (three donors) and archived paraffined human BM biopsies of MGUS (four patients) and MM patients (six patients with low (three) and high (three) IFN signal in stromal cells). (J) Heatmap representing the variation of TF activity score across samples.

Moreover, to further confirm our interesting IFN results in MM-MSC by scRNA-seq, we performed additional IF staining for IFN-signature (Isg15 and Gbp2) in MSC ($Cd271^{+}$ cells) from Ctrl, MGUS BIcγ1, MM BIcγ1, MM MIcγ1, and MM $BI_{c\gamma1}$-*Crbn*^{I391V} VRd-treated mice, and quantified the staining across all groups. These new IF results also show the same tendency as we observed in EC previously, an increase of IFN signaling in MM-BIc cells in comparison to control, MGUS and MM-MIc, as well as a reduction in MM-BIc-VRd treated mice.

The corresponding results have been incorporated into the Results section (lines 184-185: *We confirm these scRNA-seq results by IF staining of EC and MSC IFN^{+} in BM biopsies from MM $MI_{c\gamma1}$ mice (**Supplementary Fig. 5B-I**)*, and Supplementary Figures 4F–I (see previously), and new Supplementary 5 B-I, 13I and K.

The experimental details have been added to the Methods section:

Lines 206-208: *Antigen retrieval was carried out at 95°C for 30 minutes in either 10 mM citrate buffer (pH 6.0) or, specifically for CD271 staining combinations, in 10 mM Tris-1 mM EDTA buffer (pH 9.0)).*

Lines 209-212: *For immunofluorescence (IF) staining, the Formalin-Fixed Paraffin-Embedded (FFPE) sections were blocked with BSA 5% for 30 min at RT. The following primary antibodies were used: rat anti-Endomucin antibody (V.7C7) (Santa Cruz Technology 1: 50 dilution), mouse anti-CD271 monoclonal antibody (NGF Receptor) (ME20.4) (Invitrogen, 1: 50 dilution) …*

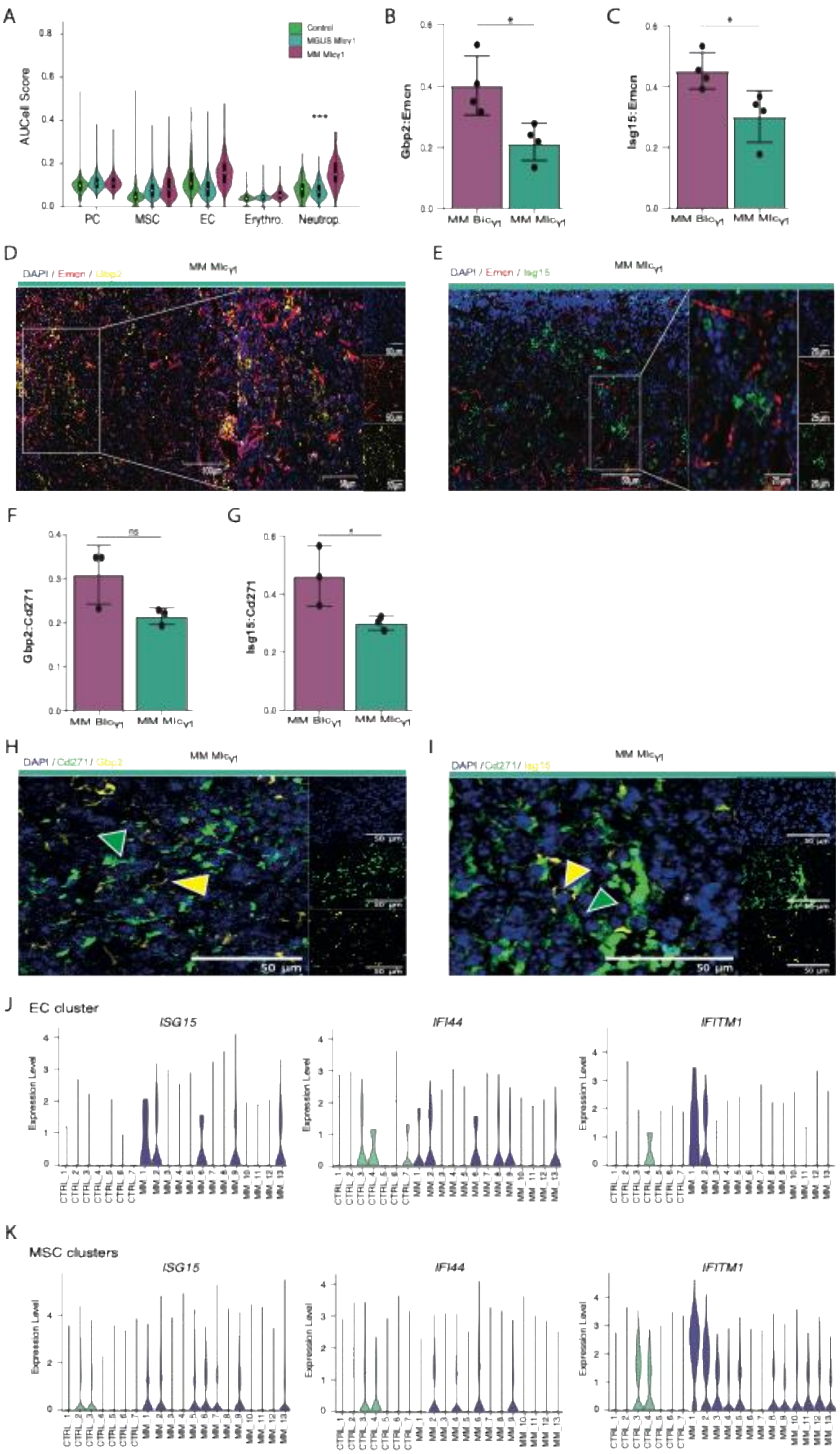

A
AUCell Score
Control
MGUS Mlcγ1
MM Mlcγ1
***
PC
MSC
EC
Erythro.
Neutrop.
B
Gbp2:Emcn
*
MM Blcγ1
MM Mlcγ1
C
Isg15:Emcn
*
MM Blcγ1
MM Mlcγ1
D
DAPI / Emcn / Gbp2
MM Mlcγ1
E
DAPI / Emcn / Isg15
MM Mlcγ1
F
Gbp2:Cd271
ns
MM Blcγ1
MM Mlcγ1
G
Isg15:Cd271
*
MM Blcγ1
MM Mlcγ1
H
DAPI / Cd271 / Gbp2
MM Mlcγ1
50 μm
I
DAPI / Cd271 / Isg15
MM Mlcγ1
50 μm
J
EC cluster
ISG15
IFI44
IFITM1
Expression Level
K
MSC clusters
ISG15
IFI44
IFITM1
Expression Level

Supplemental Figure 5: Validation in the $MI_{c\gamma1}$ model and scRNA-seq human data.

(A) Violin plots showing AUC-score for MM IFN-related signature within BM populations in the $MI_{c\gamma1}$ model. (B-C) Quantification of the percentage of endothelial IFN-enriched cells, based on the coexpression of Emcn with IFN+ cells (Gbp2, (B); Isg15, (C)) in femurs from MM $BI_{c\gamma1}$ and MM $MI_{c\gamma1}$ mice. (D-E) IF staining of EC (Emcn, red), and IFN response genes markers (Gbp2 (yellow, (D)); Isg15 (green, (E))), and nucleus (DAPI) (blue) in FFPE femurs from MM $MI_{c\gamma1}$ mice. (F-G) Quantification of the percentage of mesenchymal stromal cells IFN-enriched cells, based on the coexpression of $Cd271^{+}$ with IFN+ cells (Gbp2, (F); Isg15, (G)) in femurs from MM $BI_{c\gamma1}$ and MM $MI_{c\gamma1}$ mice. (H-I) IF staining of MSC (Cd271, green), and IFN response genes markers (Gbp2 (yellow, (H)); Isg15 (yellow, (I))), and nucleus (DAPI) (blue) in FFPE femurs from MM $MI_{c\gamma1}$ mice. Green arrows point to MSC ($Cd271^{+}$), and yellow arrows point to IFN^{+} (Gbp2/Isg15). (J) Violin plots comparing the distribution of the gene expression of *ISG15*, *IFI44*, and *IFITM1* in EC from MM patients and healthy controls from scRNA-seq published data[1]. (K) Similar to J for MSC from the same healthy controls and MM human patients from scRNA-seq published data [1].

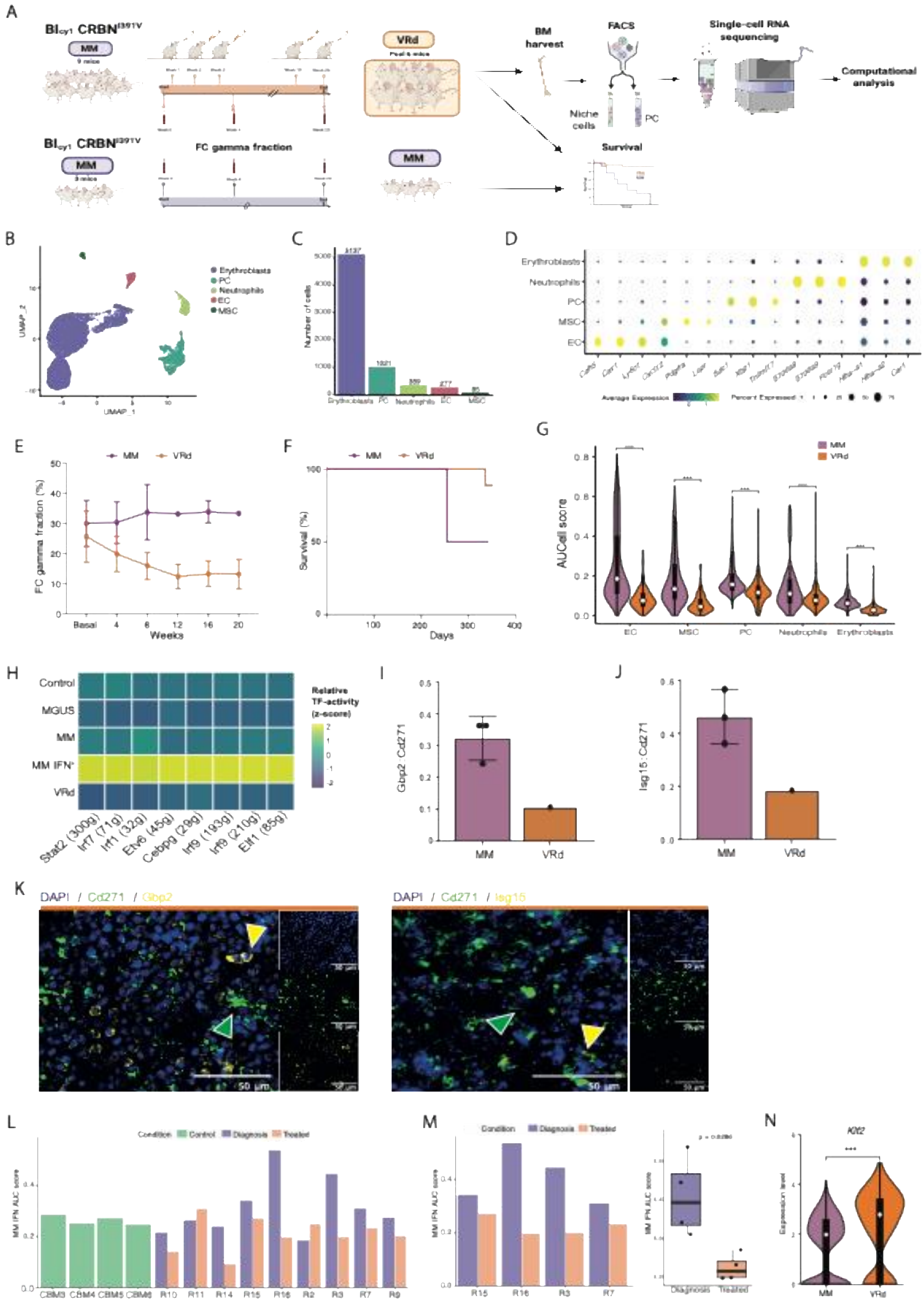

A
MM
VRd
BM harvest
FACS
Single-cell RNA sequencing
Computational analysis
Niche cells
PC
FC gamma fraction
Survival
B
Erythroblasts
PC
Neutrophils
EC
MSC
UMAP_1
UMAP_2
C
Number of cells
5137
1021
D
Average Expression
Percent Expressed
E
FC gamma fraction (%)
Weeks
F
Survival (%)
Days
G
AUCell score
H
Control
MGUS
MM
VRd
Relative TF-activity (z-score)
Stat2 (300g)
Irf7 (71g)
Irf1 (32g)
Etv6 (45g)
Cebpg (29g)
Irf9 (193g)
Irf8 (210g)
Elf1 (85g)
I
Gbp2 : Cd271
J
Isg15 : Cd271
K
DAPI / Cd271 / Gbp2
DAPI / Cd271 / Isg15
50 µm
L
Condition
Control
Diagnosis
Treated
MM IFN AUC score
M
N
Expression level

Supplemental Figure 13: Additional analysis of VRd-treated mice and transcriptional alterations in EC and MSC from human patients. (A) VRd experiment overview representing treatment administration, serum collection for FC gamma fraction, and scRNA-seq isolation of PC, MSC, and EC from MM $BI_{c\gamma1}$ *Crbn*^{I391V} (pool of six mice). MM $BI_{c\gamma1}$ *Crbn*^{I391V} were used as controls for FC gamma fraction and survival. (B) UMAP representation of BM microenvironment cells from $BI_{c\gamma1}$-*Crbn*^{I391V} VRd-treated mice, colored by cell clustering. (C) Bar plots showing the number of cells per BM population. (D) Dot plot of canonical markers used to define PC, EC, MSC, erythroblasts, and neutrophils. The dot size reflects the percentage of cells within the cluster expressing each gene, and the color represents the average expression level. (E) Representation of the percentage of serum FC gamma fraction in VRd-treated versus the MM group. (F) Kaplan–Meier survival curves of treated and non-treated MM $BI_{c\gamma1}$ mice. (G) Violin plots showing AUC-score for MM IFN-related signature within BM populations in VRd-treated and MM $BI_{c\gamma1}$ mice. (H) Heatmap representing z-scaled mean regulon activity score of IFN-related regulons in EC across each condition. (I-J) Quantification of the percentage of mesenchymal IFN$^+$ cells, determined by the coexpression of Cd271 with IFN response genes (Gbp2 (I) and Isg15 (J) in femurs from MM $BIc_{\gamma1}$ (MM) and MM $BI_{c\gamma1}$-*Crbn*^{I391V} VRd-treated (VRd) mice. (K) IF staining of MSC (Cd271, green), IFN response (Gbp2, yellow (left plot) and Isg15, yellow (right plot)), and nucleus (DAPI) (blue) in FFPE femurs from VRd-treated mice. Green arrows point to MSC (Cd271$^+$), and yellow arrows point to IFN$^+$ (Gbp2/Isg15). (L) Bar plots showing the AUC-score for MM IFN-related signature in MSC from healthy controls and MM human patients before and post-induction treatment from bulk RNA-seq published data[1] . (M) Bar plots showing the AUC-score for MM IFN-related in MSC from the subset of high IFN MM human patients before and after induction treatment[1] by individuals (left panel) and grouped by disease (right panel). (N) Violin plot displaying the upregulation of *Klf2* in VRd-treated EC vs non-treated MM EC.

Minor comments: EDITION

1. I don't really understand this line (138-140): "Considering that BIcγ1 mice received T-cell-driven immunization, we included similarly immunized YFPcγ1 mice, carrying a yellow-fluorescence reporter activated from germinal-center B cells, as controls (Fig. 1A)." What exactly was immunized in these models?

We acknowledge the reviewer's observation.

As described in the Introduction, we used our recently established mouse models of MM, which recapitulate the clinical, genetic, molecular, and immunological characteristics of MM patients[8]. These models have been previously described and published in *Nature Medicine*, where details of their generation are provided. The immunization protocol is also reported in that study and is referenced in the Methods section ("Genetic screens and immunization protocol").

*To induce the formation of GFP+ transgenic PCs in mice housed under specific-pathogen-free conditions, animals were subjected to T cell-mediated immunization with SRBCs, which were prepared in a solution of 1×10^{10} cells per ml of 100% stock solution (Fitzerald) diluted in DPBS. Mice were intraperitoneally (i.p.) administered 100 µl of the SRBC solution at 8 weeks of age and were injected again every 21 d for 4 months. After immunization, a fraction of six-month-old mice from each cohort (n = 4–6) were necropsied and analyzed to determine the presence and characteristics of B cells and PCs in spleen and BM (Supplementary Fig. 1). The remaining mice from each cohort were monitored for tumor development up to 12 months of age (Supplementary Table 1). YFPmb1, YFPcγ1 and Vk*MYC mice were similarly immunized and characterized as con trols. Survival rates of these diverse mouse strains were estimated using Kaplan–Meier OS curves.*

We therefore considered it unnecessary to repeat this information in the current manuscript. However, if the reviewer believes it important, we would be happy to include these details.

2. Additional explanation of chosen flow markers for sorting would be useful. For example, what is VDO (line 141)?

VDO refers to Vybrant Dye Orange, which is used to label live cells for flow cytometry (https://www.thermofisher.com/order/catalog/product/V3500). We also use 7AAD as a dead-cell stain. We have now added Vybrant dye orange to line 140 of the main manuscript and line 63 in supplemental methods.

3. The term “GRN inference” (line 163) should be more clearly explained in the main text, at least briefly, in addition to being explained in detail in the supplementary methods, given that a substantial part of the analysis relies on this method.

We thank the reviewer for this helpful suggestion. We have now expanded the description of gene regulatory network (GRN) inference in the main text to briefly explain its conceptual basis and purpose, given its central role in our analyses.

Specifically, we now clarify that GRN inference was used to identify transcription factors (TFs) whose coordinated target gene programs (regulons) underlie the observed transcriptional states, allowing us to move beyond differential gene expression toward the identification of putative upstream regulators. This clarification has been added to the Results section:

Lines 162–165: … *This approach reconstructs TF–centered regulatory programs (regulons), defined as sets of co-expressed genes predicted to be regulated by the same TF, and quantifies their activity at the single-cell level. Using this framework, we identified Irf7, Stat1, and Stat2 as candidate regulators with increased regulon activity in "MM2" EC …*
Lines 210: … *Next, we examined changes in regulons of the BIcγ1 model between MGUS and non-IFN MM EC.*
Lines 212-213: *Xbp1, which promotes endothelial proliferation via VEGF* [12]*, showed the highest regulon activity in MGUS EC, while the Fos regulon activity was maintained…*
Line 237: *Finally, GRN analysis identified regulons associated with MSC alterations during MM progression*
Lines 312-314: ...Furthermore, GRN analysis revealed enhanced activity of Zbtb7a and Rarg regulons, indicating activation of TF programs related to adaptive response...
Line 324-325: ...GRN analysis confirmed osteogenic reprogramming via higher regulon activity of Cebpa, Nfib, Nfic, Churc1 and Sox4 regulons…).

The full computational workflow and parameters used for GRN inference remain described in detail in the Supplementary Methods:
Line 159-162 ... *Gene Regulatory Network (GRN) activity was interrogated following the workflow implemented by SCENIC*[13]*. This approach identifies regulons, defined as sets of genes co-expressed with and predicted to be regulated by a given transcription factor (TF). Thereby capturing coordinated transcriptional programs rather than individual genes.*
Line 164-172: *To ensure comparability across conditions, an equal number of cells per condition and cell type was randomly selected for the analysis. Briefly, all the genes were trained in the GENIE3 package and used to develop a random forest model for selecting co-expression modules between TFs and target genes (regulons). Regions for TFs searching were restricted to a 10-k distance centered on the transcriptional start site (TSS) or 500 bp upstream of the TSSs. Predicted TF–target relationships were then refined using RcisTarget to retain only targets supported by enrichment of the corresponding TF-binding motifs. Finally, regulon activity was calculated for each cell, using the AUCell package, generating per-cell activity scores that reflect whether a given regulatory program is active or inactive.*
Line 174-176: To identify candidate regulators of "MM2" IFN EC, GRN inference was first computed by comparing "MM2" EC to non-IFN MM EC and sunsequently extended to include all EC across disease stages (Figure 2D; supplemental Figure 4A-C) ….).

Reviewer #4 (Remarks to the Author):

The groups of Drs. Calvo, Gómez-Cabrero, and Prosper performed single-cell RNA sequencing on two recently described mouse models of myeloma to investigate the roles of mesenchymal stromal and endothelial cells in disease progression from precursor stages to myeloma. This is a highly relevant research question. However, I have several comments and concerns:

We appreciate the reviewer's positive feedback on our work.

1. While the observed differences are interesting, the authors should better explain why these two models are suitable to study the transition from precursor stages to active myeloma. Even in the slow-progression model, the time difference between MGUS and MM appears to be only 4–6 months, whereas in human disease it often spans a decade or more. What criteria define multiple myeloma in these models (e.g., bone disease)? Does tumor load increase linearly over time, or is there a stable phase followed by rapid expansion? Furthermore, in human disease the transition is often driven by acquisition of driver mutations and emerging subclones. Do the authors observe anything similar in their models?

We thank the reviewer for these valuable and insightful comments, which also reflects a point of internal discussion within our team.

As described in the Introduction, we used our recently established mouse models of MM, which recapitulate the clinical, genetic, molecular, and immunological characteristics of MM patients. These models have been previously described and published in *Nature Medicine*, where details of their generation and characterization are provided[8]. As shown in the cited paper, the different stages are characterized by tumor burden. It is also increasing over time, but in different ways depending on the model used. On the contrary, we do not observe any acquired mutation over time in these MM mouse models, unless we introduce it.
In Figure 1 f-k from the mentioned paper[8], the electrophoresis of immunoglobulin secretion in serum samples from different mice shows M spikes corresponding to the gamma fraction. We also see the quantification of immunoglobulin isotypes in serum samples by ELISA (g), Kaplan–Meier survival curves of mouse lines that develop MM derived from several mice (h-j), as well as Flow cytometry analyses in BIcγ1 and MIcγ1 mice (k) which reveal that precursor states precede clinically evident MM in genetically heterogeneous mice.

2. Can the authors exclude that the changes in gene signatures are a consequence rather than a cause of tumor expansion? If not, I recommend avoiding conclusions that stage-specific BME–PC interactions drive disease progression.

We thank the reviewer for this important comment. We agree that our study does not allow us to formally distinguish whether the observed transcriptional changes in EC and MSC are causal drivers or consequences of tumor expansion. Because our analyses are based on cross-sectional sampling at defined disease stages, they identify stage-associated transcriptional alterations rather than direct causal relationships.

We have therefore revised the manuscript to avoid causal language and now describe these changes as stage-associated remodeling of the BME that occurs alongside MGUS-to-MM progression and therapy response. Statements implying that these alterations "drive" disease progression have been replaced with wording indicating that they may contribute to, reflect, or accompany tumor development. These changes have been implemented throughout the Results and Discussion sections.
Lines 215-216*: …Globally, these findings suggest that a stress-related regulatory state characterizes MGUS EC, prior to the angiogenic phenotype observed in MM and may be associated with disease progression.*

Lines 328-330*: Overall, our results suggest that VRd therapy is associated with suppression of IFN-driven MM signaling and transcriptional remodeling of the BM niche by triggering an adaptive response in EC and restoring MSC osteogenic potential.*
Lines 394-395*: …These findings suggest that MGUS MSC show a reshaping of their differentiation capacity and metabolism from the early stages of the disease that may contribute to a permissive microenvironment for MM cells.*
Lines 401-403*: … MSC-PC interactions were linked to adhesion, matrix organization, and migration, suggesting that MSC-PC crosstalk may be associated with MM extravasation and disease dissemination.*
Lines 416-418*: … Together, the MM IFN-inducible response uncovered in our study, supports an immunomodulatory role of the non-hematopoietic BME, with EC and MSC potentially contributing to immune remodeling and T-cell exhaustion under inflammatory conditions...*

Additionally, to further substantiate the role of the stromal compartment in regulating malignant PC behavior, we performed complementary functional validation assays.

Importantly, disease onset in the MM mouse models used in this study (MM-MIcγ1 and MM-BIcγ1) requires 6–12 months, limiting the rapid generation of additional experimental cohorts. Therefore, given the review timeline and the limited availability of these models, we conducted additional experiments using mouse BM derived MSC from mesenspheres[10] that had already been established in our laboratory at the time of revision.

Mesenspheres were generated from primary mouse BM cells as previously described[10], except we don´t use $Cd31^+$. BM cells were immunomagnetically depleted of $Cd45^+$ hematopoietic and $Ter119^+$ erythroid cells prior to plating. Mesenspheres were obtained from control (4–6-month-old) and MM-BIcγ1 (12-month-old) mice, pooling cells from three independent mice per condition. After two weeks of formation, mesenspheres were cocultured with malignant PC (MM5080[8]) for 48 h. DMSO (vehicle) or bortezomib (BTZ) was added after 24 h. At 48 h, monoculture (PC alone) and coculture samples (PC with MSC from control or MM-BIcγ1 mice) were collected and analyzed by flow cytometry analysis. Dead PC were defined as 7-AAD$^+$ CD138$^+$ cells, and dead MSC as 7-AAD$^+$ PDGFRα$^+$ CD51$^+$ cells.

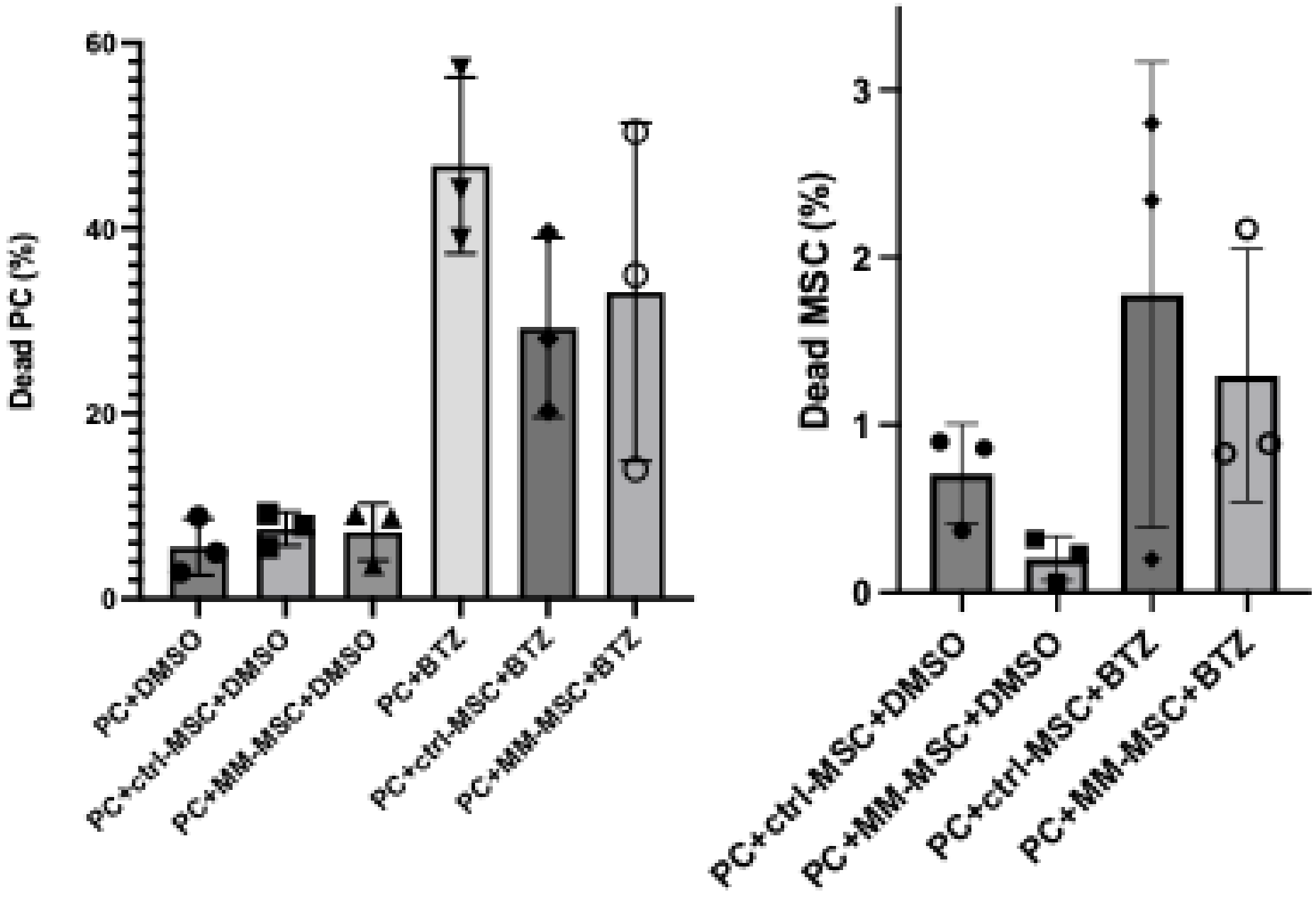

These new results indicate a trend toward a protective effect of MSC on malignant PC following BTZ treatment. Under coculture conditions, BTZ exposure was associated with a lower proportion of dead MM-PC than under monoculture, suggesting that MSC may partially attenuate BTZ-induced cytotoxicity.

In parallel, supernatants collected from the same experimental conditions were analyzed for IFN-α secretion using an IFN-α ELISA assay (https://www.thermofisher.com/elisa/product/Mouse-IFN-alpha-ELISA-Kit-/BMS6027). I

These new results reveal that FN-α levels are higher under coculture than in monoculture conditions. Following BTZ treatment, IFN-α levels are reduced. Interestingly, coculture of PC with MSC derived from control mice exhibited differential responses to stimulus, whereas coculture with MSC from MM-BIcγ1 showed limited variation across conditions. These observations may reflect a baseline activation state of MM-derived MSC, consistent with a constitutively stimulated microenvironment.

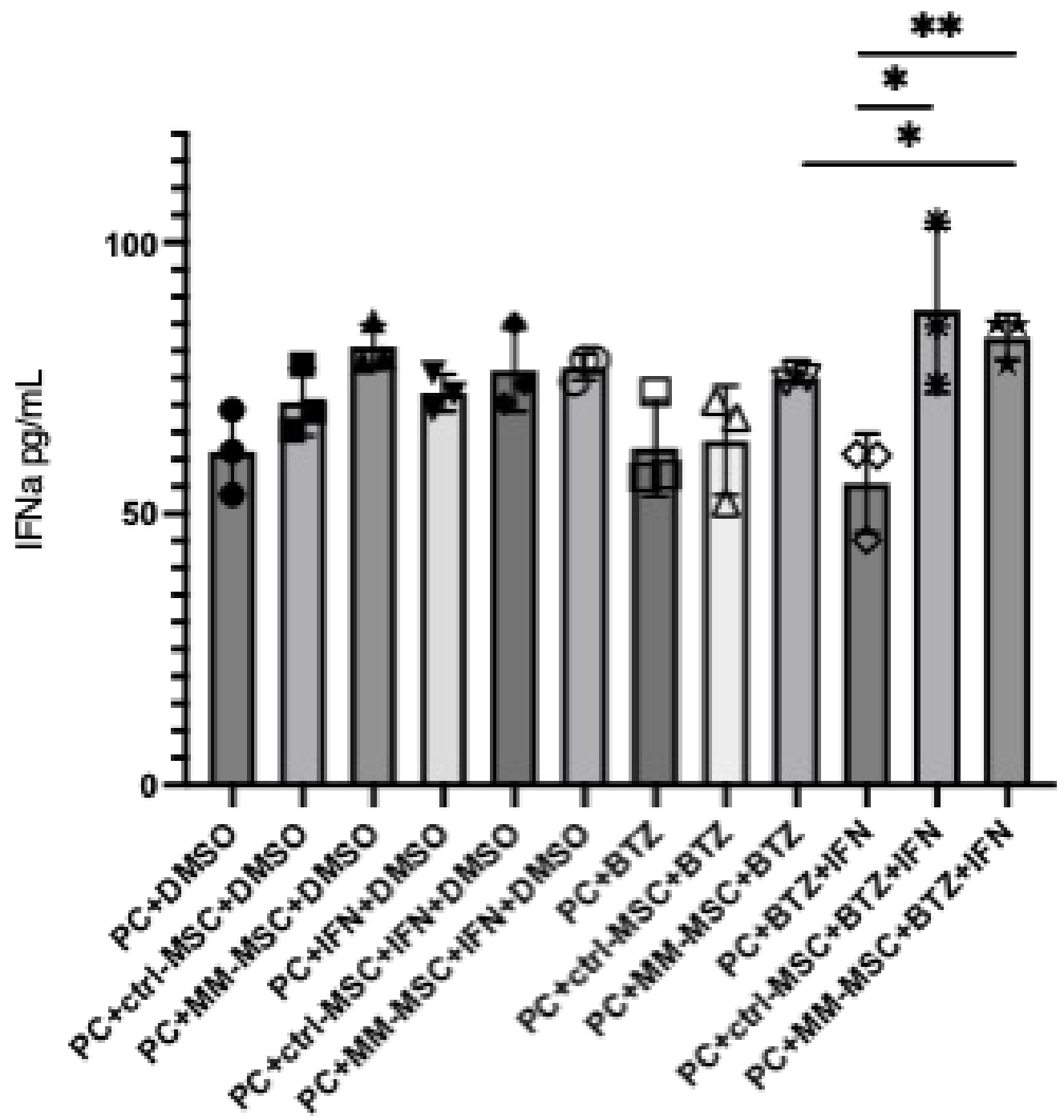

Despite the relevance of these findings, we have not incorporated these experiments into the main manuscript because we believe additional biological replicates are required to ensure robustness. Nevertheless, we would be pleased to include these analyses in the main manuscript if the reviewers consider them of particular interest.

3. Do the new findings correlate with tumor load? For example, do mice at the myeloma stage with lower plasma cell levels show the same changes as those with high tumor burden?

We thank the reviewer for this comment. However, within pooled samples, we cannot distinguish individual mice. Nevertheless, we can assess and analyze the contribution of sex to each disease state (see Supplementary Fig. 3). The MM-BIcγ1 mouse model included in the MM pool exhibited PC percentages ranging from 6.8% to 13%.

4. Please clarify how comparisons between stages were performed. If I understand correctly, the authors compared pooled samples from multiple mice without hashing or barcoding. In that case, cell-level comparisons may not be appropriate for drawing conclusions at the mouse level, even if “cells within the MM2 cluster originated from both male and female mice.” Separately analyzed biological replicates are essential.

We thank the reviewer for this important methodological point. Indeed, samples were pooled prior to scRNA-seq without hashing or barcoding. Consequently, our analyses focused on stage-associated

transcriptional signatures and gene expression changes rather than on statistical comparisons at the individual mouse level.

As we described in the supplementary methods, differential expression analyses were performed between cell populations defined by disease stage using MAST, restricting comparisons to genes expressed in at least 30% of cells and applying Bonferroni-adjusted p-values. Thus, comparisons reflect differences in transcriptional profiles between stage-associated cell states, not statistical comparisons between individual mice.

We agree that separately analyzed biological replicates are ideal for mouse-level inference. However, given the intrinsic rarity of non-hematopoietic niche populations and the requirement for stringent FACS enrichment, pooling was necessary to obtain sufficient cell numbers for transcriptional profiling. To mitigate this limitation, we applied conservative statistical thresholds, performed sex-inference analyses, and validated key findings across an independent murine model and human datasets.

To ensure that identified cell states were not driven by a single animal, we performed sex-inference analyses using sex-specific gene expression patterns (Supplementary Fig. 3). These analyses confirmed that both male- and female-derived cells contributed to all EC and MSC states, including the MM2 cluster, supporting that the observed transcriptional programs represent pooled biological contributions rather than dominance by a single individual.

We have clarified in the revised manuscript that conclusions are restricted to transcriptional remodeling of niche cell states and do not represent statistical comparisons between individual mice. This clarification has been added to Supplementary information (lines 130–132: *As samples were pooled prior to sequencing without hashing or barcoding, analyses were restricted to stage-associated transcriptional signatures rather than statistical inference at the level of individual animals*.).

5. The differences between the two models should be more clearly described and discussed. In several parts, it is unclear whether results are consistent across both models or if significant differences exist. For instance, how do the authors explain that the interferon-associated MM signature was detected in ECs and MSCs from the BIcγ1 model but absent in the more aggressive MIcγ1 model?

We acknowledge the reviewer's observation.

As described in the Introduction, we used two recently established mouse models of MM, which recapitulate the clinical, genetic, molecular, and immunological characteristics of MM patients. These models have been previously described and published in *Nature Medicine*, where details of their generation and characterization are provided[8]. We focused in MM BIcγ1 mouse model in the main manuscript, and we add the corresponding results from the MM MIcγ1 mouse model in Supplementary Figures 4, 6 and 8.

We therefore considered it unnecessary to repeat this information in the current manuscript. However, if the reviewer believes it important, we would be happy to include more details.

Regarding the question of why the MM–IFN signature was detected only in the BIcγ1 MM mouse model and not in the MIcγ1 model, we cannot draw a conclusion. Several factors may contribute to this difference. For instance, the longer time to disease progression in the BIcγ1 model, which in humans corresponds to the many years required for MGUS cells to undergo MM transformation, may allow the non-immune BM microenvironment to adapt to the malignant PC state. In contrast, the MIcγ1 model exhibits a rapid MGUS-to-MM transition, which may not allow sufficient time for EC and MSC to undergo comparable adaptive changes. But, as I mentioned, we need further investigation on it.

6. The IF stainings shown in Figures 6 and 7 are difficult to interpret. There appears to be considerable background signal, and it is not clear whether the arrows indeed point to IFN-response–positive ECs or MSCs.

We appreciate the reviewer´s valuable suggestion and agree that the images could be improved.

To address this point, we quantified the immunofluorescence data (mouse and human) and, where possible, increased the number of samples analyzed (control mice, MM $BIc_{\gamma 1}$ and MM $MIc_{\gamma 1,}$ and healthy human samples). The resulting data have been incorporated into the revised manuscript (Figs. 2I, J and 6B, and Supplementary Figs. 4F–I for the mouse data; and Figs. 7G and 7I for the human data).
In addition, in Figs. 7G and 7I we separately present high and low expression patient groups, highlighting the differences between these two patient subsets. These changes are reflected in supplementary material in lines 451-452: *MM samples were stratified into 'IFN-high' and 'IFN-low' groups using the 80th percentile as the discriminatory threshold.*

We believe that these quantitative analyses complement the immunofluorescence images, facilitate their interpretation, and strengthen the robustness of the results.

FIGURE 2

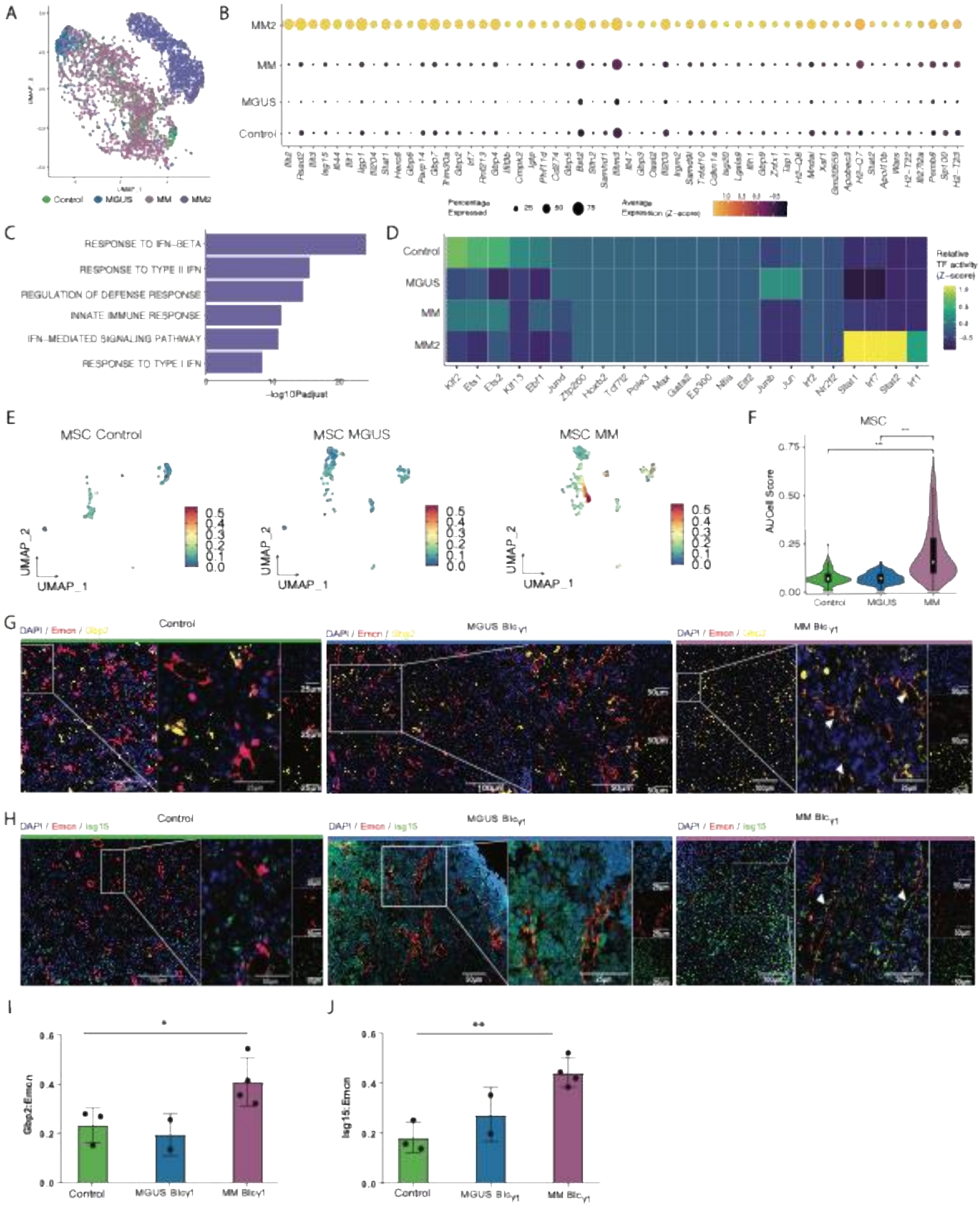

Fig. 2. Identification of a MM-specific IFN signature in the BME.
(A) UMAP dimension reduction of EC revealing a transcriptionally distinct population in MM. (B) Dot plot visualization of the markers defining EC MM2 cluster. Average gene expression is shown by color intensity, and the percentage of cells expressing each gene is shown by size. (C) Bar charts of enriched GO terms from ORA (adjusted p. value) computed using MM2 cluster markers. The horizontal axis represents the negative of the base 10 logarithm of the adjusted p. value. (D) Heatmap representing z-scaled mean regulon activity score of inferred top specific regulons for each stage. (E) UMAP plot illustrating the distribution of the IFN-related signature in MSC across control, MGUS, and MM. (F) Violin plots showing AUC-score for IFN-

related signature within MSC in control, MGUS and MM cells. (G-H) Immunofluorescence (IF) staining of EC (Emcn, red), IFN response genes markers of the MM2 cluster (Gbp2, yellow (G); Isg15, green (H)), and nucleus (DAPI) (blue) in Formalin-Fixed Paraffin-Embedded (FFPE) femurs from Control ($\text{YFP}_{c\gamma1}$), MGUS $\text{BI}_{c\gamma1}$ and MM $\text{BI}_{c\gamma1}$. White arrows point EC Ecmn^{+} IFN^{+} (Gbp2 (G)/Isg15 (H)) in MM $\text{BI}_{c\gamma1}$. (I-J) Quantification of the percentage of endothelial IFN-enriched cells, based on the coexpression of Emcn with IFN+ cells (Gbp2, (I); Isg15, (J)) in femurs from Control ($\text{YFP}_{c\gamma1}$), MGUS $\text{BI}_{c\gamma1}$ and MM $\text{BI}_{c\gamma1}$ mice.

FIGURE 6

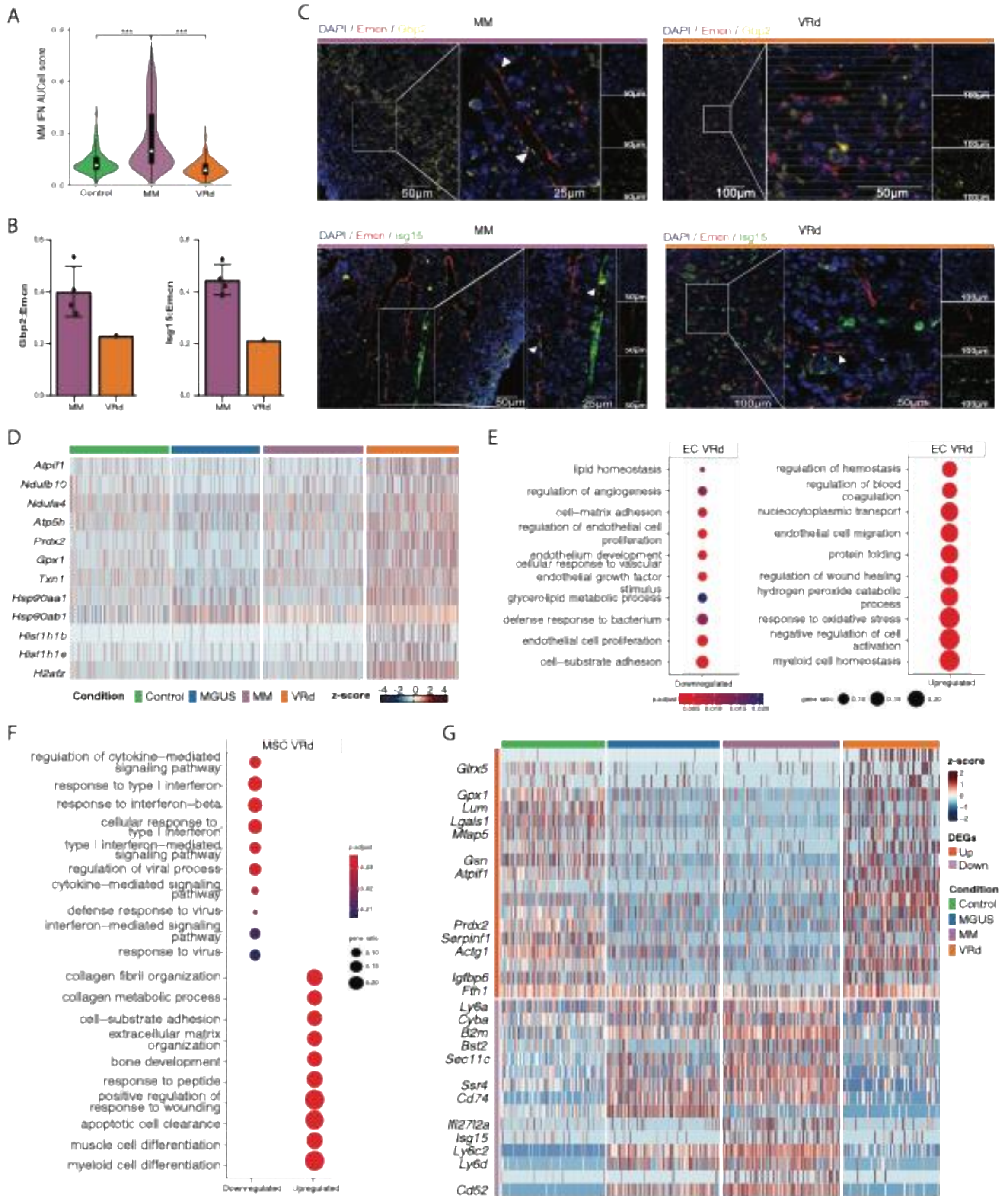

Fig. 6. VRd induces transcriptional remodeling of EC and MSC in MM.
(A) Violin plot showing AUC-score for IFN-related signature in EC from Control ($\text{YFP}_{c\gamma1}$), MM $\text{BIc}_{\gamma1}$ (MM) and MM $\text{BI}_{c\gamma1}$-*Crbn*$^{\text{I391V}}$ VRd-treated (VRd) mice. (B) Quantification of the percentage of endothelial IFN^{+} cells, determined by the coexpression of Emcn with IFN response genes (Gbp2 (left plot) and Isg15 (right

plot) in femurs from MM BI$c_{\gamma 1}$ (MM) and MM BI$_{c\gamma 1}$-*Crbn*^{I391V} VRd-treated (VRd) mice. (C) IF staining of EC (Emcn, red), IFN response genes (Gbp2 (yellow, (upper plot)); Isg15 (green, (bottom plot)), and nucleus (DAPI) (blue) in FFPE femurs from MM BI$_{c\gamma 1}$ mice (MM) and VRd-treated mice (VRd). White arrows point EC (Ecmn^{+}) IFN^{+} (Gbp2/Isg15). (D) Heatmap showing the expression of stress-related DEGs identified in the comparison between treated and non-treated non-IFN MM EC at different stages of the disease. Color represents the z-score scaled expression values. (E) Significant terms derived from ORA between VRd-treated and untreated non-IFN MM EC. (F) Significant terms derived from ORA of MSC from MM mice before and after VRd treatment. (G) Heatmap projecting the expression of DEGs identified in the comparison between non-treated and treated MSC at different stages of the disease. Color represents the z-score scaled expression values.

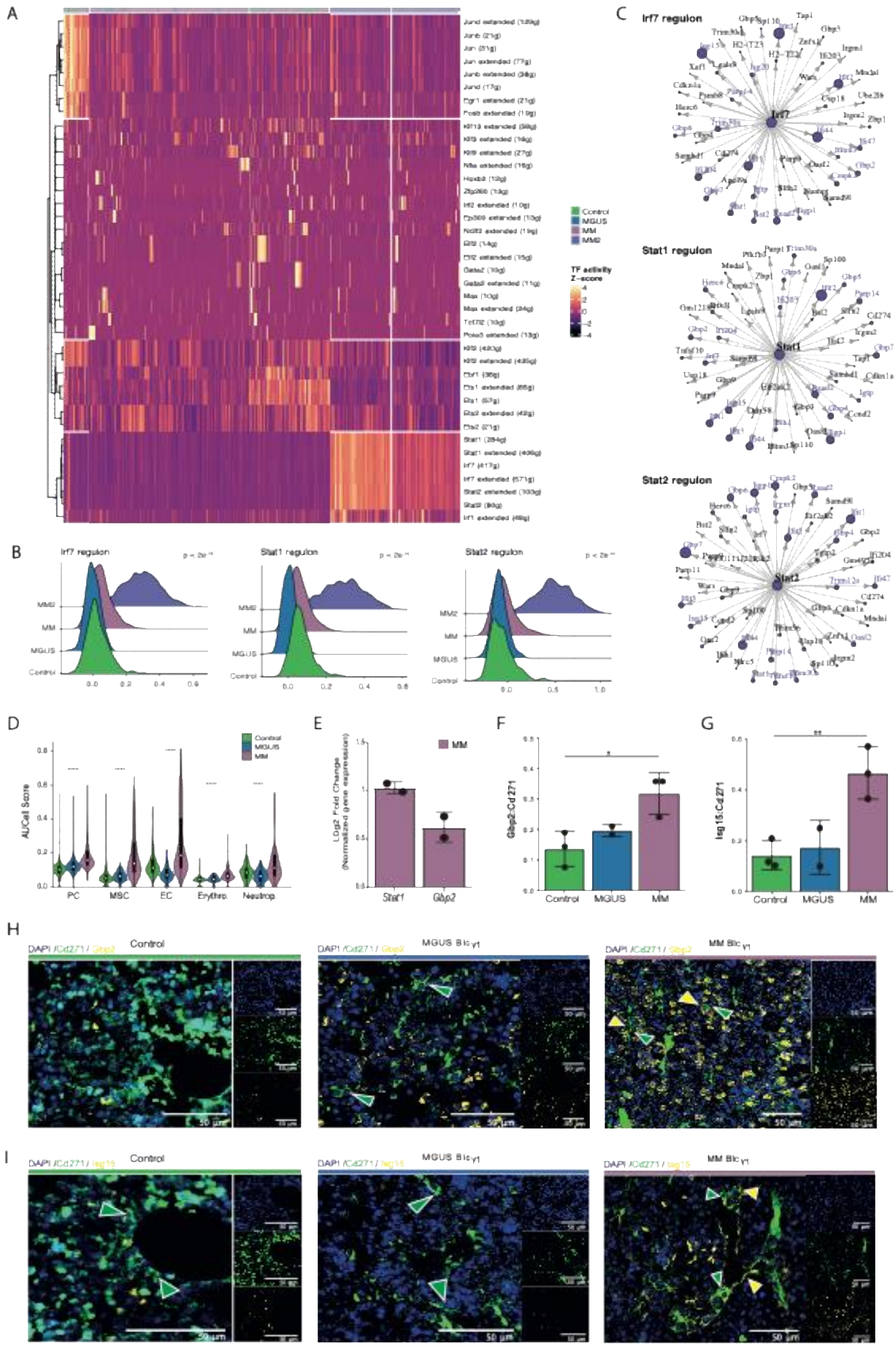

A
B
C
D
E
F
G
H
I
Irf7 regulon
Stat1 regulon
Stat2 regulon
Control
MGUS
MM
MM2
TF activity Z-score
AUCell Score
PC
MSC
EC
Erythro.
Neutrop.
Log2 Fold Change (Normalized gene expression)
Stat1
Gbp2
Gbp2:Cd271
Isg15:Cd271
DAPI /Cd271 / Gbp2
DAPI /Cd271 / Isg15
MGUS Blc γ1
MM Blc γ1
50 μm

Supplemental Figure 4: Additional information on the MM IFN-related signature in the $BI_{c\gamma1}$ model. (A) Heatmap showing the activities of the enriched regulons in EC (Z-score of regulon activity). (B) Density plots showing the score of Irf7, Stat1, and Stat2 regulons in EC (p. = adjusted p-value). (C) Regulon network of Irf7, Stat1, and Stat2 representing the top 50 target genes. (D) Violin plots showing AUC-score for MM IFN-related signature within BM populations in the $BI_{c\gamma1}$ model. (E) Quantitative PCR (qPCR) analysis of the IFN regulon (*Stat1)* and the associated gene (*Gbp2)* in MSC derived from mesenspheres of MM BIcγ1 mice. Gene expression is presented as log_2 fold change relative to MSC derived from mesenspheres of control mice. Data were normalized to the reference gene *Gapdh*. N=2 independent biological replicates per group. (F-G) Quantification of the percentage of mesenchymal stromal cells IFN-enriched cells, based on the coexpression of $Cd271^+$ with IFN+ cells (Gbp2, (F); Isg15, (G)) in femurs from Control, MGUS $BI_{c\gamma1}$ and MM $BI_{c\gamma1}$ mice. (H-I) IF staining of MSC (Cd271, green), and IFN response genes markers (Gbp2 (yellow, (H)); Isg15 (yellow, (I))), and nucleus (DAPI) (blue) in FFPE femurs from Control, MGUS $BI_{c\gamma1}$, and MM $BI_{c\gamma1}$ mice. Green arrows point to MSC ($Cd271^+$), and yellow arrows point to IFN^+ (Gbp2/Isg15).

FIGURE 7

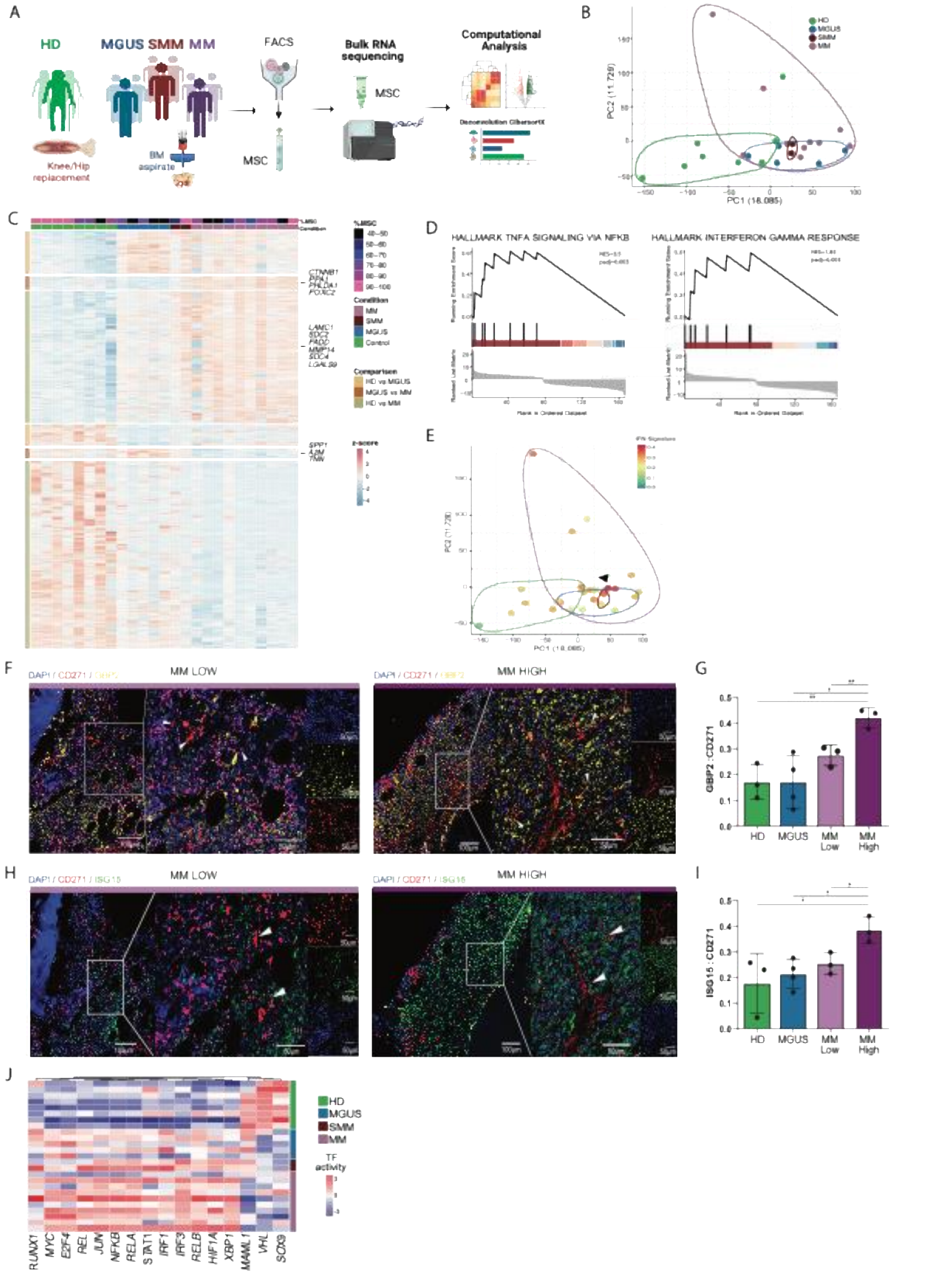

Fig. 7. Transcriptional dynamics of human MSC during MM progression.
(A) Experimental workflow used for the isolation and bulk RNA-seq analysis of human BM MSC. (B) PCA of MSC expression profiles shows clustering by disease stage. The color of the dots represents disease stages.

(C) Heatmap showing the expression profiles of DEGs identified in each pairwise comparison across disease progression. (D) GSEA plots for the gene sets 'TNFα signaling via NFκB' (left panel) and 'Interferon gamma response' (right panel) in the comparison between healthy donors (HD)- MSC and MM-MSC. (E) PCA plot showing the MM-IFN signature score in each human sample, previously identified murine BM MM cells. (F) IF staining of MSC (CD271, red), IFN response genes (*GBP2*, yellow), and nucleus (DAPI) (blue) in archived paraffin-embedded human BM biopsies of MM patients with low (MM LOW) and high IFN signal (MM HIGH) in stromal cells (see Supplementary Table 2). White arrows point to MSC ($CD271^{+}$) in MM LOW IFN samples, and MSC ($CD271^{+}$) IFN^{+} (GBP2) in MM HIGH IFN samples. (G) Quantification of the percentage of mesenchymal IFN^{+} cells, determined by the coexpression of $CD271^{+}$ with IFN response genes (*GBP2*) in FFPE biopsies from HD (three donors) and archived paraffined human BM biopsies of MGUS (four patients) and MM patients (six patients with low (three) and high (three) IFN signal in stromal cells). (H) IF staining of MSC (CD271, red), IFN response genes (*ISG15*, green), and nucleus (DAPI) (blue) in archived paraffin-embedded human BM biopsies of MM patients with low (MM LOW) and high IFN signal (MM HIGH) in stromal cells (see Supplementary Table 2). White arrows point to MSC ($CD271^{+}$) in MM LOW IFN samples, and MSC ($CD271^{+}$) IFN^{+} (ISG15) in MM HIGH IFN samples. (I) Quantification of the percentage of mesenchymal IFN^{+} cells, determined by the coexpression of $CD271^{+}$ with IFN response genes (*ISG15*) in FFPE biopsies from HD (three donors) and archived paraffined human BM biopsies of MGUS (four patients) and MM patients (six patients with low (three) and high (three) IFN signal in stromal cells). (J) Heatmap representing the variation of TF activity score across samples.

Moreover, to further confirm our interesting IFN results in MM-MSC by scRNA-seq, we performed additional IF staining for IFN-signature (Isg15 and Gbp2) in MSC ($Cd271^{+}$ cells) from Ctrl, MGUS $BI_{c\gamma1}$, MM $BI_{c\gamma1}$, MM $MI_{c\gamma1}$, and MM $BI_{c\gamma1}$-*Crbn*^{I391V} VRd-treated (VRd) mice and quantified the staining across all groups.

These new IF results also show the same tendency as we observed in EC previously, an increase of IFN signaling in MM-BIc cells in comparison to control, MGUS and MM $MI_{c\gamma1}$, as well as a reduction in MM $BI_{c\gamma1}$-*Crbn*^{I391V} VRd-treated (VRd) mice.

The corresponding results have been incorporated into the Results section (lines 184-185: *We confirm these scRNA-seq results by IF staining of EC and MSC IFN^{+} in BM biopsies from MM $MI_{c\gamma1}$ mice (**Supplementary Fig. 5B-I**)*, and Supplementary Figures 4F–I, and new Supplementary 5 B-I (see previously), and 13I and K.

The experimental details have been added to the Methods section (lines 206-208: *Antigen retrieval was carried out at 95°C for 30 minutes in either 10 mM citrate buffer (pH 6.0) or, specifically for CD271 staining combinations, in 10 mM Tris-1 mM EDTA buffer (pH 9.0)).*

Lines 209-212:

For immunofluorescence (IF) staining, the Formalin-Fixed Paraffin-Embedded (FFPE) sections were blocked with BSA 5% for 30 min at RT. The following primary antibodies were used: rat anti-Endomucin antibody (V.7C7) (Santa Cruz Technology 1: 50 dilution), mouse anti-CD271 monoclonal antibody (NGF Receptor) (ME20.4) (Invitrogen, 1: 50 dilution) …

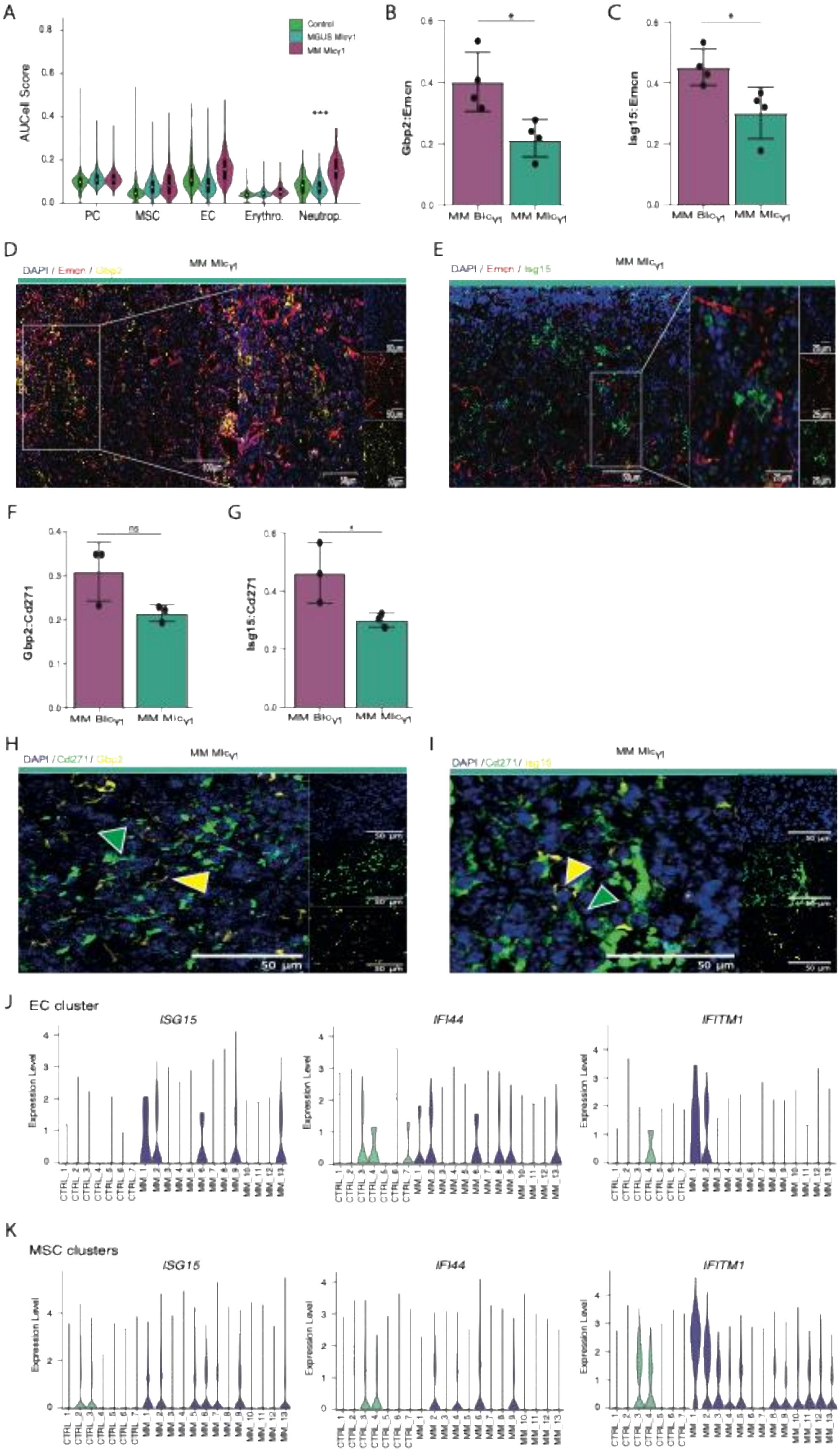

A
AUCell Score
Control
MGUS Mlcγ1
MM Mlcγ1
***
PC
MSC
EC
Erythro.
Neutrop.
B
Gbp2:Emcn
*
MM Blcγ1
MM Mlcγ1
C
Isg15:Emcn
*
MM Blcγ1
MM Mlcγ1
D
DAPI / Emcn / Gbp2
MM Mlcγ1
E
DAPI / Emcn / Isg15
MM Mlcγ1
F
Gbp2:Cd271
ns
MM Blcγ1
MM Mlcγ1
G
Isg15:Cd271
*
MM Blcγ1
MM Mlcγ1
H
DAPI / Cd271 / Gbp2
MM Mlcγ1
50 µm
I
DAPI / Cd271 / Isg15
MM Mlcγ1
50 µm
J
EC cluster
ISG15
IFI44
IFITM1
Expression Level
K
MSC clusters
ISG15
IFI44
IFITM1
Expression Level

Supplemental Figure 5: Validation in the $MI_{c\gamma1}$ model and scRNA-seq human data. (A) Violin plots showing AUC-score for MM IFN-related signature within BM populations in the $MI_{c\gamma1}$ model. (B-C) Quantification of the percentage of endothelial IFN-enriched cells, based on the coexpression of Emcn with IFN+ cells (Gbp2, (B); Isg15, (C)) in femurs from MM $BI_{c\gamma1}$ and MM $MI_{c\gamma1}$ mice. (D-E) IF staining of EC (Emcn, red), and IFN response genes markers (Gbp2 (yellow, (D)); Isg15 (green, (E))), and nucleus (DAPI) (blue) in FFPE femurs from MM $MI_{c\gamma1}$ mice. (F-G) Quantification of the percentage of mesenchymal stromal cells IFN-enriched cells, based on the coexpression of $Cd271^{+}$ with IFN+ cells (Gbp2, (F); Isg15, (G)) in femurs from MM $BI_{c\gamma1}$ and MM $MI_{c\gamma1}$ mice. (H-I) IF staining of MSC (Cd271, green), and IFN response genes markers (Gbp2 (yellow, (H)); Isg15 (yellow, (I))), and nucleus (DAPI) (blue) in FFPE femurs from MM $MI_{c\gamma1}$ mice. Green arrows point to MSC ($Cd271^{+}$), and yellow arrows point to IFN^{+} (Gbp2/Isg15). (J) Violin plots comparing the distribution of the gene expression of *ISG15*, *IFI44*, and *IFITM1* in EC from MM patients and healthy controls from scRNA-seq published data[1]. (K) Similar to J for MSC from the same healthy controls and MM human patients from scRNA-seq published data [1].

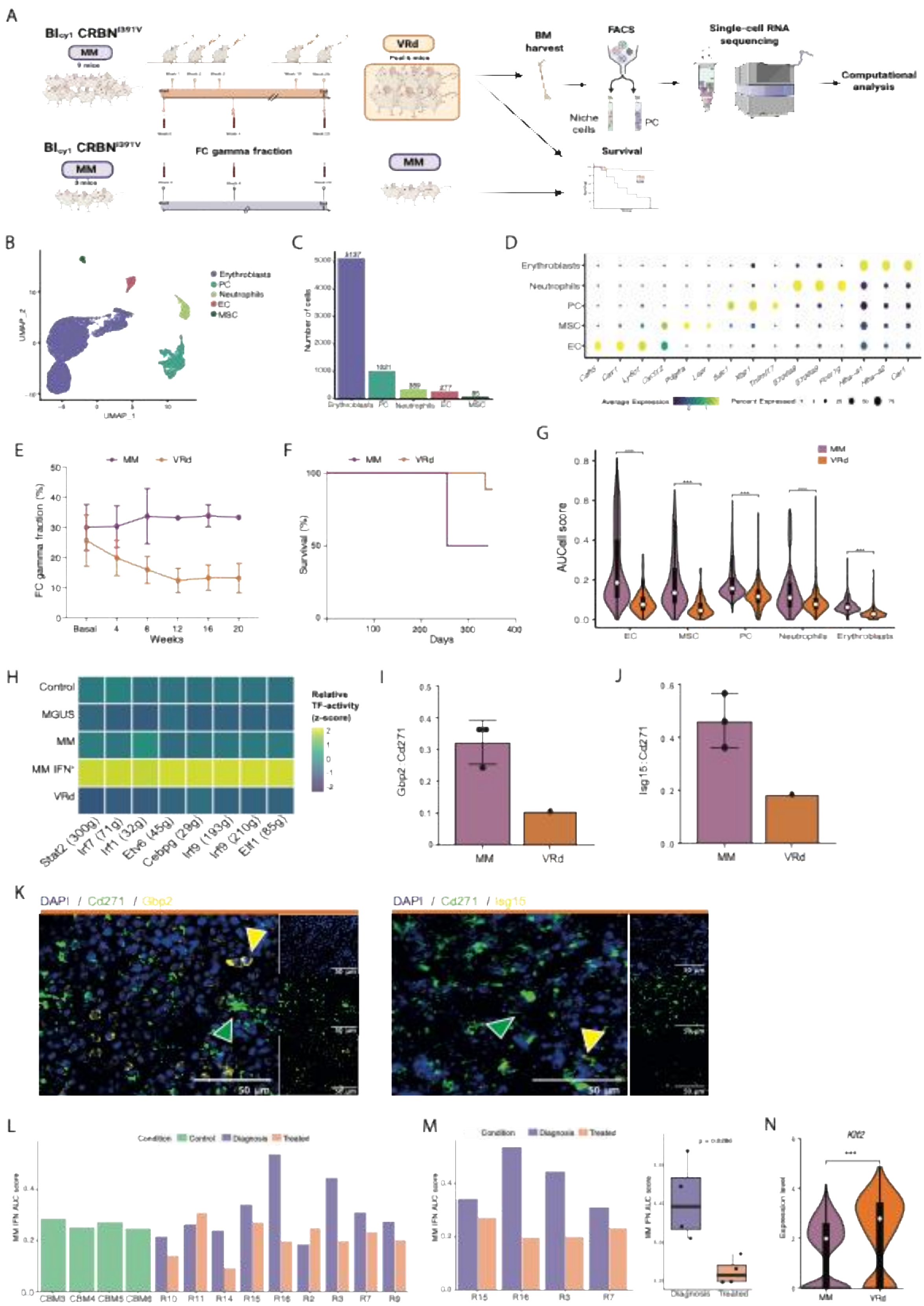

A
BI$_{cγ1}$ CRBN^{I391V}
MM
FC gamma fraction
VRd
MM
BM harvest
FACS
Niche cells
PC
Single-cell RNA sequencing
Computational analysis
Survival
B
Erythroblasts
PC
Neutrophils
EC
MSC
UMAP_1
UMAP_2
C
Number of cells
5137
1021
E
MM
VRd
FC gamma fraction (%)
Weeks
Basal
F
Survival (%)
Days
G
AUCell score
EC
MSC
PC
Neutrophils
Erythroblasts
H
Control
MGUS
MM
MM IFN⁺
VRd
Relative TF-activity (z-score)
Stat2 (300g)
Irf7 (71g)
Irf1 (32g)
Etv6 (45g)
Cebpg (29g)
Irf9 (193g)
Irf8 (210g)
Elf1 (65g)
I
Gbp2 : Cd271
J
Isg15 : Cd271
K
DAPI / Cd271 / Gbp2
DAPI / Cd271 / Isg15
50 µm
L
Condition
Control
Diagnosis
Treated
MM IFN AUC score
CBM3
CBM4
CBM5
CBM6
R10
R11
R14
R15
R16
R2
R3
R7
R9
M
Diagnosis
Treated
N
Expression level

Supplemental Figure 13: Additional analysis of VRd-treated mice and transcriptional alterations in EC and MSC from human patients. (A) VRd experiment overview representing treatment administration, serum collection for FC gamma fraction, and scRNA-seq isolation of PC, MSC, and EC from MM $BI_{c\gamma1}$ *Crbn*^{I391V} (pool of six mice). MM $BI_{c\gamma1}$ *Crbn*^{I391V} were used as controls for FC gamma fraction and survival. (B) UMAP representation of BM microenvironment cells from $BI_{c\gamma1}$-*Crbn*^{I391V} VRd-treated mice, colored by cell clustering. (C) Bar plots showing the number of cells per BM population. (D) Dot plot of canonical markers used to define PC, EC, MSC, erythroblasts, and neutrophils. The dot size reflects the percentage of cells within the cluster expressing each gene, and the color represents the average expression level. (E) Representation of the percentage of serum FC gamma fraction in VRd-treated versus the MM group. (F) Kaplan–Meier survival curves of treated and non-treated MM $BI_{c\gamma1}$ mice. (G) Violin plots showing AUC-score for MM IFN-related signature within BM populations in VRd-treated and MM $BI_{c\gamma1}$ mice. (H) Heatmap representing z-scaled mean regulon activity score of IFN-related regulons in EC across each condition. (I-J) Quantification of the percentage of mesenchymal IFN^{+} cells, determined by the coexpression of Cd271 with IFN response genes (Gbp2 (I) and Isg15 (J) in femurs from MM $BIc_{\gamma1}$ (MM) and MM $BI_{c\gamma1}$-*Crbn*^{I391V} VRd-treated (VRd) mice. (K) IF staining of MSC (Cd271, green), IFN response (Gbp2, yellow (left plot) and Isg15, yellow (right plot)), and nucleus (DAPI) (blue) in FFPE femurs from VRd-treated mice. Green arrows point to MSC (Cd271^{+}), and yellow arrows point to IFN^{+} (Gbp2/Isg15). (L) Bar plots showing the AUC-score for MM IFN-related signature in MSC from healthy controls and MM human patients before and post-induction treatment from bulk RNA-seq published data[1] . (M) Bar plots showing the AUC-score for MM IFN-related in MSC from the subset of high IFN MM human patients before and after induction treatment[1] by individuals (left panel) and grouped by disease (right panel). (N) Violin plot displaying the upregulation of *Klf2* in VRd-treated EC vs non-treated MM EC.

7. The authors should be more specific about the human data. How many patients show enrichment of the murine MM–IFN-related genes in ECs and MSCs, and how is "enrichment" defined? Do all of these patients exhibit a "shared immunomodulatory response of the BME"?

We thank the reviewer for this insightful comment. Our team carefully considered the importance of these results.

Regarding our data, in our cohort of 10 MM patients, 4 exhibited a high MM-IFN related signature (see Figure 7E, red circles and see Supplementary methods). In addition, we assessed the presence of the MMM-IFN signature by immunofluorescence (see Figure 7 F-I) and observed that, among 6 MM patients, 3 displayed high IFN levels, whereas the remaining samples showed low IFN expression. Furthermore, analysis of publicly available data from de Jong *et al*[1]. revealed that, among 13 MM patients, a subset also exhibited high expression of IFN-induced genes (see new Supplementary Fig. 5 J, K).

8. Please describe in detail how murine bone marrow non-hematopoietic cells were isolated. Is the sampling procedure comparable to that used for human bone marrow aspirates? How were samples from orthopedic surgery donors processed? Differences in sample type and processing could significantly affect EC and MSC composition and thus represent a potential confounder.

We thank the reviewer for pointing this out.
We consider that we have described all isolation methods in detail in the Supplemental methods section (see "Isolation and fluorescence-activated cell sorting of murine BM microenvironment and PC" and "Isolation and fluorescence-activated cell sorting of human BM mesenchymal-osteolineage primed cells").
We therefore considered it unnecessary to provide additional methodological details in the main text. However, if the reviewer considers further clarification necessary, we would be happy to include additional experimental information.

Regarding the reviewer´s question about differences in sample type and processing, we do not expect these factors to affect cellular composition once the populations have been isolated and purified. It is nevertheless true that BM samples from obtained from orthopedic surgeries, contain EC and higher representation of non-immune BM niche cells, as previously reported[9].

References

1. de Jong, M. M. E. *et al.* The multiple myeloma microenvironment is defined by an inflammatory stromal cell landscape. *Nature Immunology 2021 22:6* **22**, 769–780 (2021).
2. Martínez-Fleta, P. *et al.* ISG15 modulates inflammatory profiles and ability to activate CD8 + T cells in bone marrow-derived dendritic cells. *Cellular and Molecular Life Sciences 2025 82:1* **82**, 362- (2025).
3. Finak, G. *et al.* MAST: A flexible statistical framework for assessing transcriptional changes and characterizing heterogeneity in single-cell RNA sequencing data. *Genome Biol.* **16**, 1–13 (2015).
4. Zavidij, O. *et al.* Single-cell RNA sequencing reveals compromised immune microenvironment in precursor stages of multiple myeloma. *Nature Cancer 2020 1:5* **1**, 493–506 (2020).
5. Boiarsky, R. *et al.* Single cell characterization of myeloma and its precursor conditions reveals transcriptional signatures of early tumorigenesis. *Nature Communications 2022 13:1* **13**, 1–15 (2022).
6. Kellermayer, Z. *et al.* Interferon gamma-mediated prevention of tumor progression in a mouse model of multiple myeloma. *Hemasphere* **8**, (2024).
7. Ghamlouch, H. *et al.* A proinflammatory response and polarized differentiation of stromal elements characterizes the murine myeloma bone marrow niche. *Exp. Hematol. Oncol.* **14**, (2025).
8. Larrayoz, M. *et al.* Preclinical models for prediction of immunotherapy outcomes and immune evasion mechanisms in genetically heterogeneous multiple myeloma. *Nat. Med.* **29**, 632–645 (2023).
9. Bandyopadhyay, S. *et al.* Mapping the cellular biogeography of human bone marrow niches using single-cell transcriptomics and proteomic imaging. *Cell* **187**, 3120-3140.e29 (2024).
10. Forte, D. *et al.* Bone Marrow Mesenchymal Stem Cells Support Acute Myeloid Leukemia Bioenergetics and Enhance Antioxidant Defense and Escape from Chemotherapy. *Cell Metab.* **32**, 829-843.e9 (2020).
11. Pajtler, K. *et al.* Production of Chick Embryo Extract for the Cultivation of Murine Neural Crest Stem Cells. *J. Vis. Exp.* e2380 (2010) doi:10.3791/2380.
12. Yang, J. *et al.* The interaction between XBP1 and eNOS contributes to endothelial cell migration. *Exp. Cell Res.* **363**, 262–270 (2018).
13. Aibar, S. *et al.* SCENIC: Single-cell regulatory network inference and clustering. *Nat. Methods* **14**, 1083 (2017).
